# Selective Labeling of Peptides with *o*‐Carboranes via Manganese(I)‐Catalyzed C−H Activation

**DOI:** 10.1002/chem.202200811

**Published:** 2022-05-23

**Authors:** Becky Bongsuiru Jei, Long Yang, Lutz Ackermann

**Affiliations:** ^1^ Institut für Organische und Biomolekulare Chemie Georg-August-Universität Göttingen Tamannstraße 2 37077 Göttingen Germany; ^2^ Woehler Research Institute for Sustainable Chemistry (WISCh) Georg-August-Universität Göttingen Tammannstraße 2 37077 Göttingen Germany

**Keywords:** carboranes, C−H alkenylation, chemoselectivity, manganese catalysis, peptide-diversification

## Abstract

A robust method for the selective labeling of peptides via manganese(I) catalysis was devised to achieve the C‐2 alkenylation of tryptophan containing peptides with 1‐ethynyl‐*o*‐carboranes. The manganese‐catalyzed C−H activation was accomplished with high catalytic efficiency, and featured low toxicity, high functional group tolerance and excellent *E‐*stereoselectivity. This approach unravels a promising tool for the assembly of *o*‐carborane with structurally complex peptides of relevance to applications in boron neutron capture therapy.

## Graphical abstract

**The direct incorporation** of a carborane moiety into tryptophan‐containing peptides is presented as a sustainable method to access novel potential anti‐cancer and BNCT agents via non‐toxic manganese(I) catalysis.

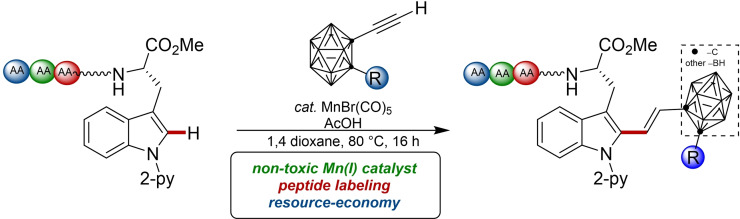

The application of indole alkaloids^[1]^ and tryptophan containing peptides^[2]^ in medicine, such as cancer therapy, has attracted interest towards the sustainable assembly of diverse indole decorated structural motifs.^[3]^ Transition metal catalyzed C−H activation has evolved as an enabling tool for the site‐selective functionalization of indoles^[4]^ and late‐stage modification of peptides.^[5]^ Thus during recent years, increasing attention has been channeled towards the late‐stage diversification of tryptophan containing peptides via transition metal catalyzed C−H activation of the indole moiety.^[6]^ While manganese(I) catalysis has evolved as a powerful tool for molecular catalysis^[7]^ and despite indisputable progress attained with 3d metal complexes in C−H activation, their application towards the modification of carborane derivatives has proven elusive.

Carboranes are cage boron clusters containing one or more carbons with a three‐dimensional electronic delocalization.^[8]^ Due to the similarity in bond length between the carbon‐carbon, carbon‐boron and boron‐boron bonds, these clusters exhibit similar reactivity to that of benzene.^[9]^ During the last decade, the application of carborane clusters in materials science,^[10]^ coordination chemistry,^[11]^ and boron neutron capture therapy (BNCT)^[12]^ has attracted major attention. This has brought about magnificent advances in regioselective cage B−H functionalization,^[13]^ cage C−H functionalization^[14]^ and sustainable protocols^[15]^ for the modification of these boron rich clusters. Noble transition metal complexes have enabled recent achievements in the cage B‐alkenylation of *o*‐carboranes.^[16]^ Also, the synthesis of cage C‐alkenyl‐*o*‐carboranes have been accomplished by the condensation of decaborane with alkenyl acetylenes,^[17]^ Ullmann coupling^[18]^ and Wittig reaction^[19]^ (Scheme 1b). Xie and co‐workers reported the nickel‐mediated cross‐coupling of *o*‐carboranes with styrenes via a *o*‐carborynyl intermediate^[20]^ (Scheme 1a) and organophosphine‐catalyzed alkenylation of *o*‐carboranes with electron deficient alkynes.^[21]^ However, these protocols suffer challenges such as, the use of stoichiometric amounts of nickel and organolithium reagents as well as long reaction time required for optimum yields with limited scope to activated alkynes. These limitations jeopardize the efficiency of the pre‐existing methods and makes the protocols largely impractical. In spite of the recently unveiled light assisted alkenylation of 1‐iodo‐*o*‐carborane (Scheme 1c),^[22]^ sustainable access to 1‐alkenylcarboranes is still open to innovative improvements.

**Scheme 1:**
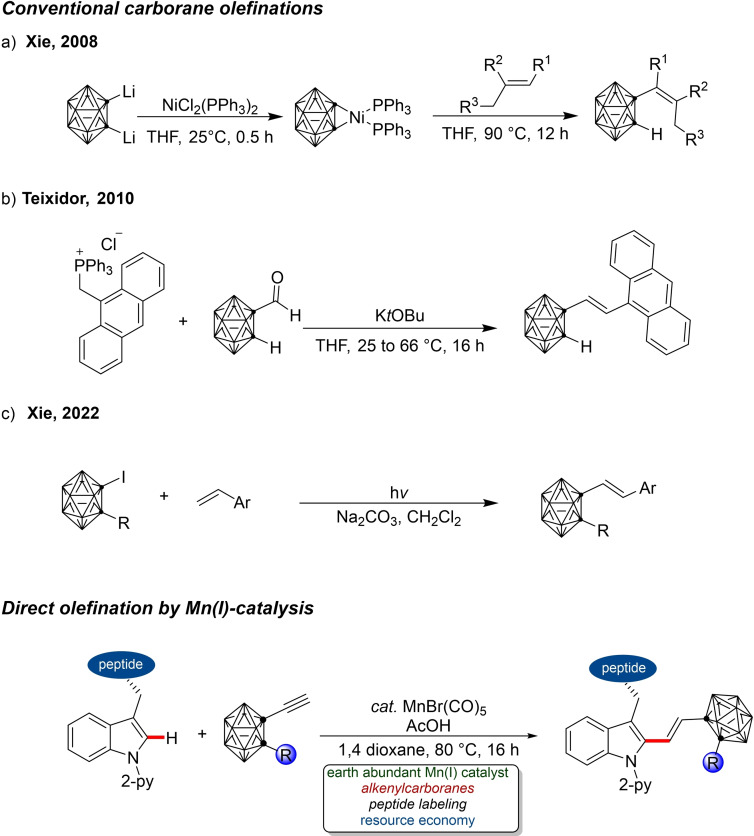
Manganese(I) catalyzed C−H alkenylation of tryptophan.

The increasing demand for more sustainable methods to access organic molecules and development of novel compounds has fueled the use of earth‐abundant 3d transition metal complexes as the future of organometallic catalysis. Their comparatively low toxicity, cost efficiency and unique chemo‐selectivity gives them high preference over their 4d and 5d counterparts.^[23]^ The versatility of a manganese(I) regime in the hydroarylation of tryptophan containing peptides has been recently achieved with terminal alkynes.^[24]^ Within our program on sustainable synthesis, we report the first earth‐abundant manganese(I) site and stereo selective C−H activation for the hydroarylation of decorated peptides to access carborane‐labeled peptides.

We initiated our studies with a representative set of reaction conditions consisting of tryptophan **1 a** with *o*‐carboranyl acetylene **2 a** in the presence of a MnBr(CO)_5_, DIPEA in Et_2_O at 80 °C for 24 h, successfully furnishing 74 % yield of the desired product **3 aa**. An improvement in yield of the alkene **3** to 81 % was observed with AcOH as the additive (entry 2). In addition, a switch in solvent from Et_2_O to 1,4‐dioxane resulted in an appreciable increase in the catalytic efficiency (entry 3). Comparatively, NaOAc was shown to be less effective than AcOH (entry 4). Additionally, we investigated the efficacy of other transition metal catalysts such as ReBr(CO)_5_, [Cp*RhCl_2_]_2_, and Mn_2_(CO)_10_ in the C‐2 alkenylation. Interestingly, the formation of product **3** was only enabled by MnBr(CO)_5_ (entries 5–7). An increase in the reaction temperature to 100 °C diminished the efficacy of our manganese(I) catalyst (entry 8). Control experiments revealed the importance of each component of the system. First, the principal role of the manganese(I) catalyst was indisputably reflected by the inability to achieve the transformation in the absence of the metal catalyst (entry 9). Second, the unique role of the additive in the manganese(I) regime was expressed in a drastic drop in the yield in its absence (entry 10). Next, the robust and user‐friendly nature of manganese(I) catalysis allowed the catalysis to proceed efficiently under air (entry 11). Moreover, the catalysis was effective in the absence of DIPEA (96 %, entry 12). Overall optimal yields were obtained with an equimolar amount of **1 a** and **2 a** with MnBr(CO)_5_ (10 mol %) as catalyst_,_ along with AcOH (20 mol %) in 1,4 dioxane at 80 °C for 16 h (Table 1).

**Table 1:** Optimization of direct alkenylation.^[a]^

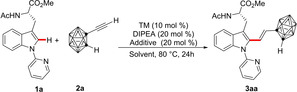				
Entry	TM	Additive	Solvent	yield [%]
**1**	MnBr(CO)_5_	PhCO_2_H	Et_2_O	74
**2**	MnBr(CO)_5_	AcOH	Et_2_O	81
**3**	MnBr(CO)_5_	AcOH	1,4‐dioxane	94
**4**	MnBr(CO)_5_	NaOAc	1,4‐dioxane	85
**5**	Mn_2_(CO)_10_	AcOH	1,4‐dioxane	—
**6**	ReBr(CO)_5_	AcOH	1,4‐dioxane	—
**7**	[Cp*RhCl_2_]_2_	AcOH	1,4‐dioxane	—
**8**	MnBr(CO)_5_	AcOH	1,4‐dioxane	80^[b]^
**9**	—	AcOH	1,4‐dioxane	—
**10**	MnBr(CO)_5_	—	1,4‐dioxane	28
**11**	MnBr(CO)_5_	AcOH	1,4‐dioxane	94^[c]^
**12**	MnBr(CO)_5_	AcOH	1,4‐dioxane	96^[d]^

[a] Reaction conditions: **1 a** (0.10 mmol), **2 a** (0.10 mmol), MnBr(CO)_5_ (10 mol %), AcOH (20 mol %), DIPEA (20 mol %) solvent (1.0 mL), under N_2_, 80 °C, 24 h. Isolated yields are reported. [b] At 100 °C. [c] Under air. [d] No DIPEA for 16 h.

With the optimized conditions in hand, we were keen to explore the robustness of our manganese(I) catalyst (Scheme 2). Generally, excellent *E*‐stereoselectivity of >20/1 were observed for all products. *N*‐protected tryptophans featuring the Boc, acetyl and the phthalimide groups **3 aa**, **3 ba** and **3 ca**, respectively, were transformed to the desired product in excellent yields. The detachable benzyl ester tryptophan was also transformed into the desired product **3 da** in excellent yield. It is worthy of note that a variety of dipeptides furnished the desired products without jeopardizing the tolerance of sensitive functional groups on the amino acid side chains. The robustness of the manganese(I) catalyst was hence highlighted with the successful transformation of dipeptides with alkyl side chains (**3 fa**–**3 ia**), oxidation sensitive groups found in methionine (**3 ja**) and modified cysteine (**3 ka**). Furthermore, electron donating (Me: **3 ma**) and withdrawing (CF_3_: **3 na**) substituents on the pyridine directing group successfully furnished the desired products in high yields (85 % and 55 %).

**Scheme 2:**
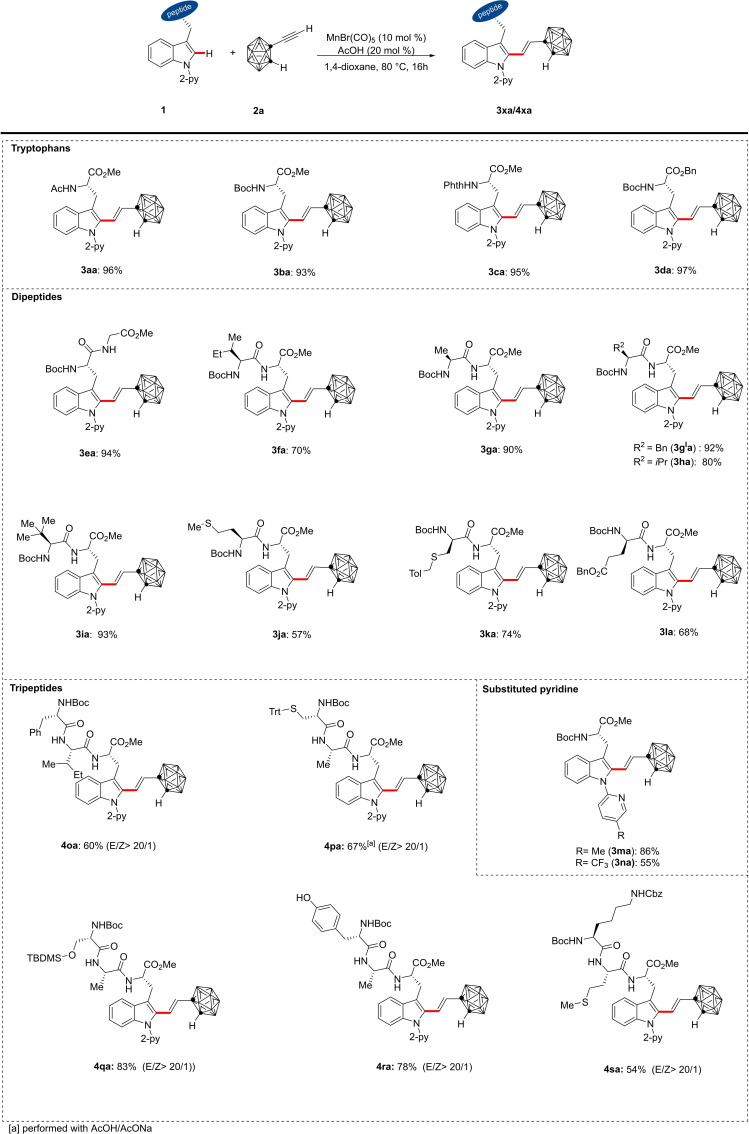
Manganese(I)‐catalyzed C−H alkenylations of tryptophans, dipeptides and tripeptides **1**.

To ascertain the validity of our manganese(I) catalysis to structurally complex substrates we extended our scope to tripeptides. Interestingly, tripeptides anchoring hydroxyl groups like tyrosine (**4 ra**) and *O*‐silylated serine (**4 qa**) furnished the alkenylated peptides with high chemo and *E*‐stereoselectivities. In addition, cysteine‐based tripeptide (**4 pa**) also afforded the desired product in the presence of AcOH/AcONa buffer system in good yields.

Motivated by the versatility of our manganese(I) catalysis, we demonstrated the effect of different substituents on the 2‐*o*‐carboranyl cage carbon (Scheme 3). Hence, methyl and *n*‐butyl decorated *o*‐carboranes furnished the products with the amino acid **5 ab** and **5 bc**, dipeptide **5 fb** as well as tripeptide **5 rb**, while internal alkynes gave thus far less satisfactory results.

**Scheme 3:**
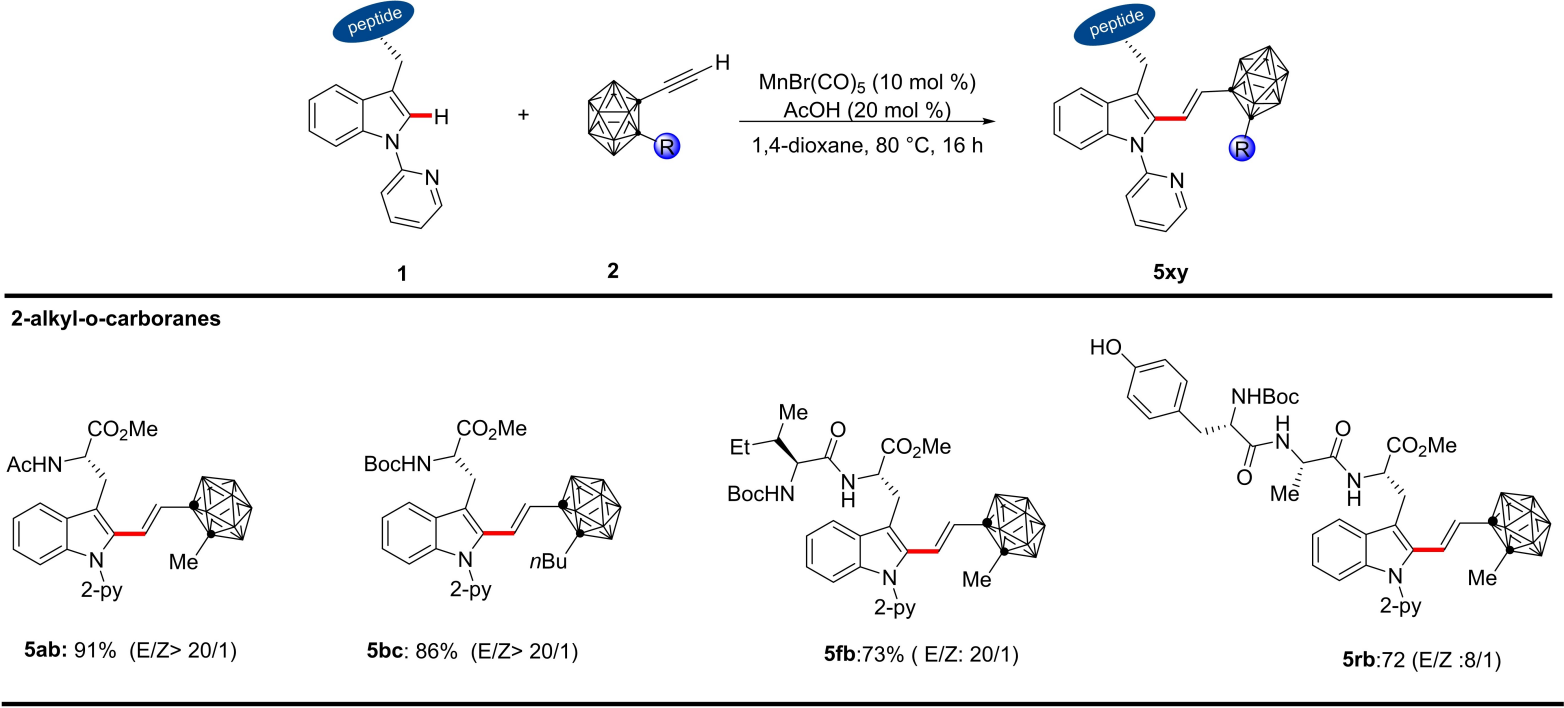
Manganese(I)‐catalyzed C−H alkenylation with substituted *o*‐carboranes **2**.

The applicability of our approach on scale was demonstrated by performing a 1.0 mmol scale with comparable yield (Scheme 4a). In a quest to further derivatize our products, subjection of the thus‐obtained amino acid **3 da** to rhodium(III) catalysis^[6a]^ resulted in the C‐7 amidation of **3 da** to afford NH_2_‐free amide **6** in good yield (Scheme 4b).

**Scheme 4:**
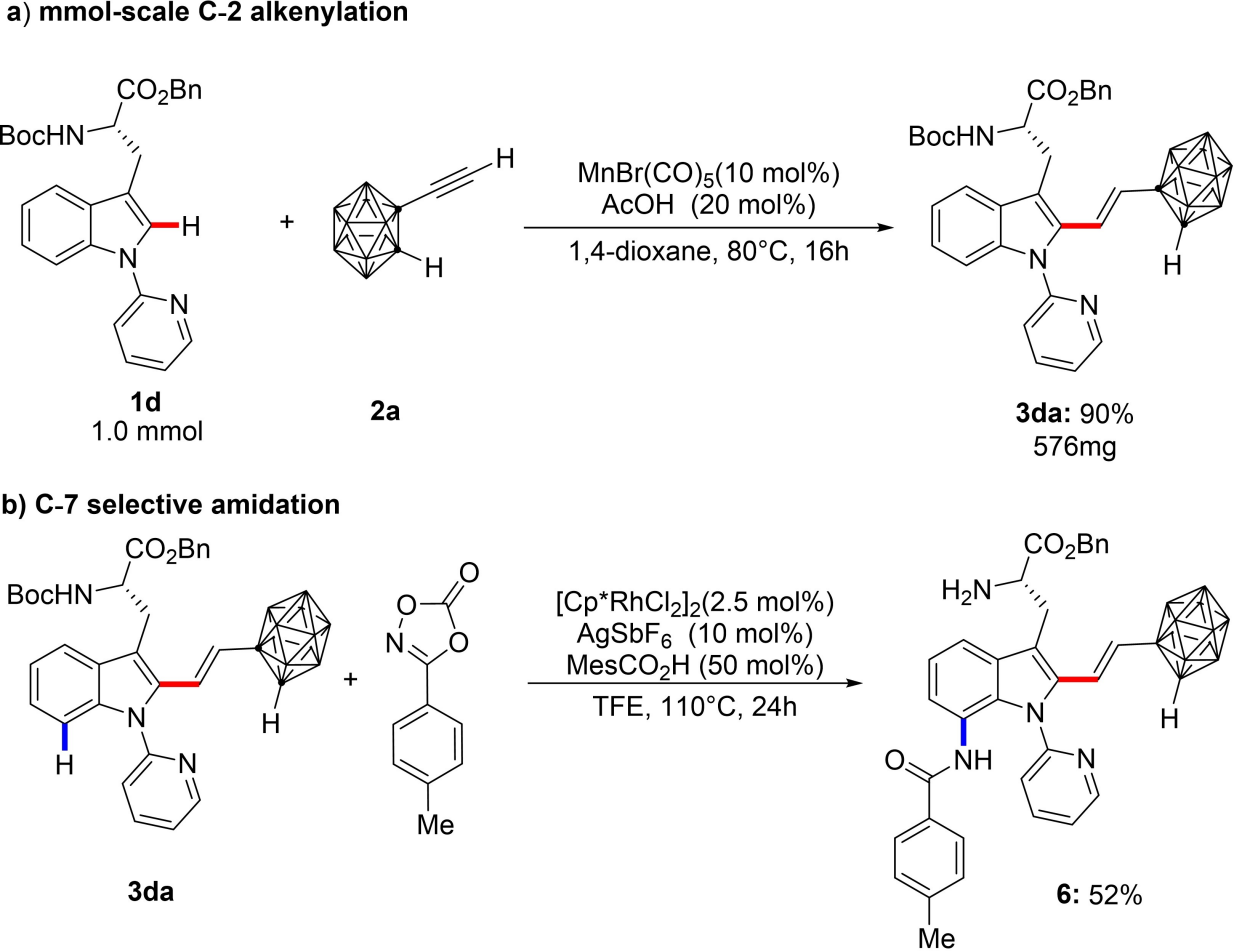
a) 1.0 mmol scale C−H alkenylation; b) Late‐stage C−H amidation of conjugated **3 da**.

Given the unique selectivity of our acid assisted manganese(I) regime, we became interested in delineating the catalyst's mode of action. A notable H/D scrambling at the C‐2 position of the indole moiety was observed in the re‐isolated [D]_n_‐**1 a** in the presence of deuterated acetic acid under standard catalytic conditions. This was indicative of a reversible C−H activation step. In addition, deuterium incorporation was also observed for the olefinic protons of product [D]_n_‐**3 aa** (Scheme 5a) supporting the existence of H/D exchange in the reaction.^[25]^ Intermolecular competition experiments revealed a preferential reactivity of electron donating substituent **3 m** over the electron withdrawing group **3 n** on the directing group (Scheme 5b).

**Scheme 5:**
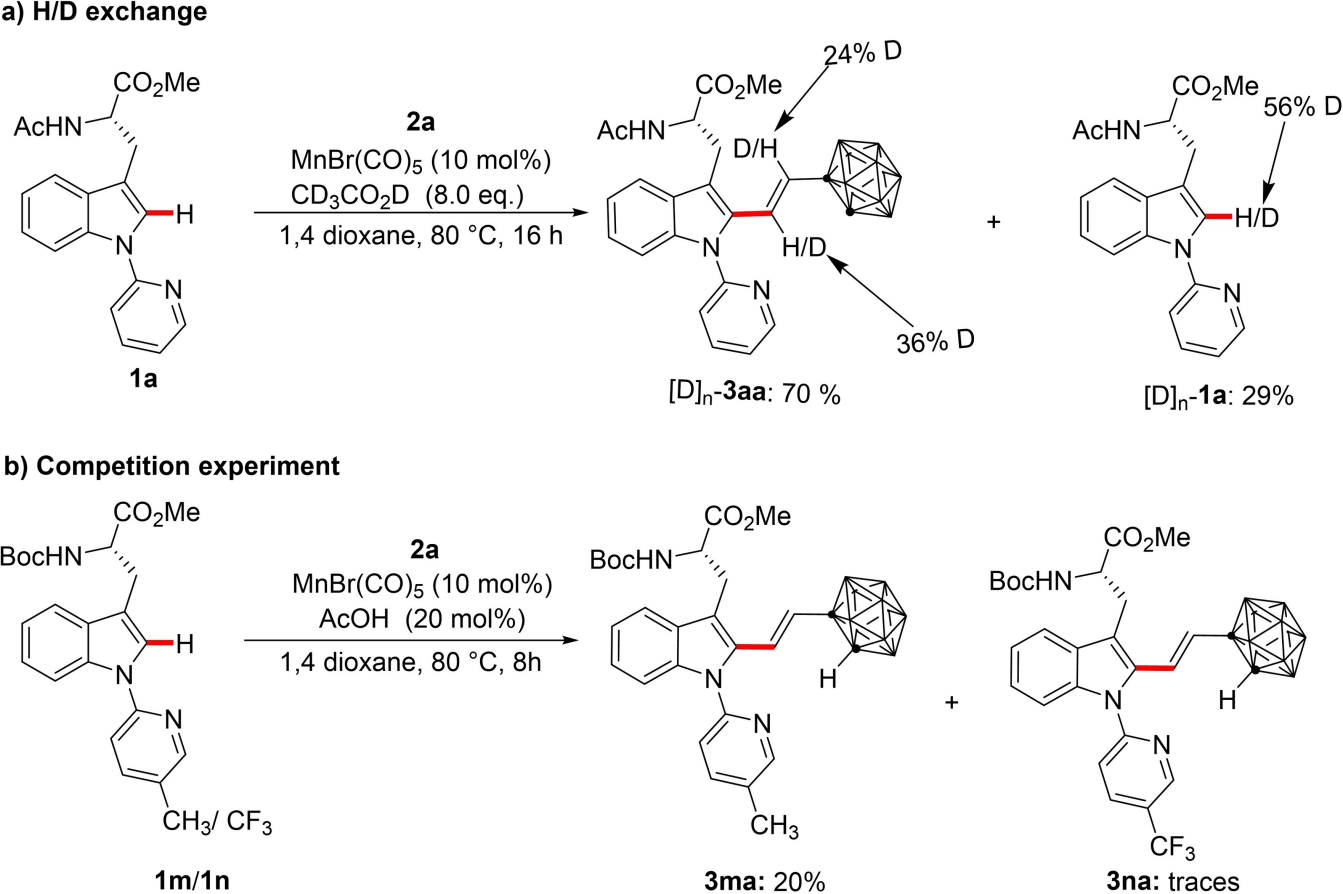
a) H/D exchange experiment; b) Intermolecular competition experiment.

Based on our studies and previous reports,^[26]^ we propose the C‐2 selective manganese(I) catalyzed alkenylation to proceed as follows.^[27]^ The reaction starts with the reversible C−H activation to give cyclometallated complex **A**. Coordination of alkyne **2** results in the formation of complex **B**, which undergoes migratory insertion of the alkyne at C‐2 to form a seven‐membered cyclometallated complex **C**. Proto‐demetallation from acetic acid furnishes the desired product **3**. At the same time, complex **D** is generated and releases the acetate which further reacts with **1** and **2** to regenerate complex **B**.

In conclusion, we have developed a manganese(I)‐catalyzed C−H alkenylation to access *o*‐carborane decorated tryptophan containing peptides for the assembly of structurally complex boron‐rich peptides. The strategy is atom economical, efficient, and demonstrates excellent chemo/stereo‐selectivity. The thus‐obtained boron‐rich compounds are available to further modifications with the prospects for future applications as potential candidates to cancer therapy, within boron neutron capture therapy.

## Experimental Section

Detailed experimental procedures and analytical data are available in the Supporting Information online.

## Conflict of interest

The authors declare no conflict of interest.

## Supporting information

As a service to our authors and readers, this journal provides supporting information supplied by the authors. Such materials are peer reviewed and may be re‐organized for online delivery, but are not copy‐edited or typeset. Technical support issues arising from supporting information (other than missing files) should be addressed to the authors.

Supporting Information

## Data Availability

The data that support the findings of this study are available in the supplementary material of this article.
