# Supplementary material for: Selective Labeling of Peptides with o‐Carboranes via Manganese(I)‐Catalyzed C−H Activation
Source: Chemistry. 2022 May 23;28(37):e202200811. doi: 10.1002/chem.202200811 (PMC9320968; doi:10.1002/chem.202200811)
Supplement: Supplementary file 1 — Supporting Information [file CHEM-28-0-s001.pdf]

Chemistry–A European Journal

Supporting Information

Selective Labeling of Peptides with *o*-Carboranes via Manganese(I)-Catalyzed C–H Activation

Becky Bongsuiru Jei, Long Yang, and Lutz Ackermann*

Table of Content

General remarks -- S-2

General procedure for manganese(I) catalyzed C-2 alkenylation of tryptophan -------------- S-3

Characterization data for products **3, 4** and **5** --- S-3

Late-stage C-7 amidation of **3da** ---S-25

Intermolecular competition experiment --S-26

H/D exchange experiment--S-26

Studies of potential racemization---S-26

Plausible mechanism---S-27

References ---S-29

^{1}H, ^{13}C, ^{11}B, ^{19}F NMR spectra --S-29

General Remarks

Catalytic reactions were carried out in oven dried 25 mL Schlenk tube, unless otherwise stated. The 1-ethynyl-*o*-carboranes[1] **2** and the peptides[2] were prepared according to literature reported procedures. Other chemicals were obtained from commercial sources and were used without further purification. Yields refer to isolated compounds estimated to be >95% pure as determined by ^{1}H NMR. Chromatographic separations were carried out on Merck Geduran SI-60 (0.040–0.063 mm, 230–400 mesh ASTM). IR spectra were recorded on a Bruker FT-IR alpha-P device. EI-MS was recorded on Jeol AccuTOF at 70 eV; ESI-MS was recorded on Bruker Daltonik micrOTOF and maXis. Melting points (M.p.) were measured on StuartTM melting point apparatus SMP3, and the values are uncorrected. NMR spectra were recorded on a Bruker Avance III 300, Avance III 400 and Avance III HD 500 in the solvent indicated. Chemical shifts (δ) are provided in ppm and spectra refer to non-deuterated solvent signal.

General Procedure for Manganese(I) Catalyzed C–2 Alkenylation of Tryptophan

Amino acid or peptide **1** (0.1 mmol, 1.0 equiv.), ethynyl-*o*-carborane **2** (0.1 mmol, 1.0 equiv.), $MnBr(CO)_5$ (2.7 mg, 10 mol %) and AcOH (1.2 µL, 20 mol %) and 1,4-dioxane (1 mL) were placed in a 25 mL oven dried Schlenk tube and stirred at 80 °C for 16 h. After cooling to room temperature, the reaction mixture was transferred to a round bottom flask with 10 mL of ethyl acetate and concentrated in vacuum. Column chromatography in *n*-hexane/EtOAc followed by gel permeation chromatography afforded the desired product **3, 4** and **5**.

Characterization Data for Products 3, 4 and 5.

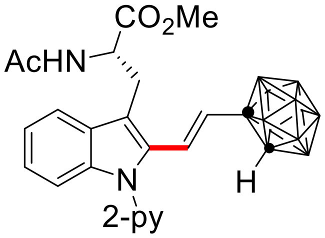

3aa

Methyl (*S*, *E*)-2-acetamido-3-[2-(*o*-carboranyl-1-en-1-yl)]-1-(pyridin-2-yl)-1*H*-indol-3-yl)propanoate (3aa)

The general procedure was followed using methyl N_α-acetyl-1-(pyridin-2-yl)-L-tryptophanate (**1a**) (33.7 mg, 0.1 mmol) and 1-ethynyl-*o*-carborane **2a** (16.8 mg, 0.1 mmol). Column chromatography on silica (*n*-hexane/EtOAc = 4/1) afforded **3aa** (48.7 mg, 96%) as yellow solid. **M.P.** = 101-103 °C. **^{1}H NMR** (300 MHz, $CDCl_3$): δ = 8.72 (dd, *J* = 4.9, 1.9 Hz, 1H), 7.94 (td, *J* = 7.7, 1.9 Hz, 1H), 7.53 – 7.45 (m, 2H), 7.41 (dd, *J* = 7.5, 4.9 Hz, 1H), 7.34 – 7.29 (m, 1H), 7.28 – 7.15 (m, 2H)., 6.85 (d, *J* = 16.1 Hz, 1H), 6.30 (d, *J* = 7.8 Hz, 1H), 6.14 (d, *J* = 16.1 Hz, 1H), 4.75 (td, *J* = 8.5, 5.0 Hz, 1H), 4.34 (s, 1H), 3.48 (s, 3H), 3.43 – 3.20 (m, 2H), 2.05 (s, 3H). **^{13}C NMR** (101 MHz, $CDCl_3$): δ = 172.5 (C_q), 169.7 (C_q), 150.9 (C_q), 149.4 (CH), 138.5 (CH), 137.4 (C_q), 131.7 (C_q), 128.6 (C_q), 125.8 (CH), 125.8 (CH), 125.7 (CH), 124.5 (CH), 122.6 (CH), 121.4 (CH), 118.9 (CH), 113.8 (C_q), 111.1 (CH), 74.2 (cage C_q), 60.7 (cage CH), 53.1 (CH), 52.6 (CH_3), 28.9 (CH_2), 23.2 (CH_3). **^{11}B NMR** (128 MHz, $CDCl_3$): δ = -2.61 (1B), -4.93 (1B), -9.06 (2B), -11.36 (3B), -12.76 (3B). **IR** (ATR): 3050, 2596, 1655, 1588, 1469, 1437, 1372, 1224, 744 cm^{-1}. **MS** (ESI) *m*/*z* (relative intensity): 507(90) $[M]^+$. **HR-MS** (ESI): *m*/*z* calcd. for $C_{23}H_{31}B_{10}N_3O_3$ $[M+H]^+$: 508.3369, found: 508.3373.

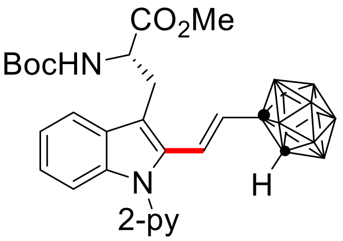

3ba

Methyl (*S*, *E*)-3-[2-(2-*o*-carboranyl-vinyl)-1-(pyridin-2-yl)-1*H*-indol-3-yl]-2-[(*tert*-butoxycarbonyl)]amino)propanoate (3ba)

The general procedure was followed using methyl N_α-(*tert*-butoxycarbonyl)-1-(pyridin-2-yl)-*L*-tryptophanate (**1b**) (39.5 mg, 0.1 mmol) and 1-ethynyl-*o*-carborane **2a** (16.8 mg, 0.1 mmol). Column chromatography on silica in (*n*-hexane/EtOAc = 4/1) afforded **3ba** (54.8 mg, 97%) as white foamy solid. **M.P.** = 102-103°C. **^{1}H NMR** (400 MHz, $CDCl_3$): δ = 8.71 – 8.65 (dd, , J = 4.9, 2.0 Hz, 1H), 7.89 (td, J = 7.8, 2.0 Hz, 1H), 7.53 – 7.41 (m, 2H), 7.36 (ddd, J = 7.5, 4.9, 1.0 Hz, 1H), 7.27 (d, J = 8.0 Hz, 1H), 7.23 – 7.11 (m, 2H), 6.85 (d, J = 16.1 Hz, 1H), 6.09 (d, J = 16.1 Hz, 1H), 5.33 (d, J = 8.3 Hz, 1H), 4.56 – 4.36 (m, 1H), 4.33 (s, 1H), 3.41 (s, 3H), 3.25 (m, J = 14.1, 7.1 Hz, 2H), 1.43 (s, 9H). **^{13}C NMR** (126 MHz): δ = 172.6 (C_q), 155.0 (C_q), 151.0 (C_q), 149.6 (CH), 138.4 (CH), 137.3 (C_q), 131.5 (C_q), 128.7 (C_q), 125.8 (CH), 125.7 (CH), 124.4 (CH), 122.4 (CH), 121.4 (CH), 121.4 (CH), 119.0 (CH), 114.15 (C_q), 111.1 (CH), 80.4 (C_q), 74.3 (cage C_q), 60.6 (cage CH), 54.5 (CH), 52.4 (CH_3), 29.7 (CH_2), 28.3 ($3CH_3$). **^{11}B NMR** (128 MHz, $CDCl_3$): δ = -2.52 (1B), -4.82 (1B), -8.97 (2B), -11.13 (6B). **IR** (ATR): 3051, 2976, 2596, 1743, 1698, 1588, 1469, 1437, 1365, 1172 cm^{-1}. **MS** (ESI) *m*/*z* (relative intensity): 565 (90) $[M]^+$. **HR-MS** (ESI): *m*/*z* calcd. for $C_{26}H_{37}B_{10}N_3O_4$ $[M+H]^+$: 566.3783, found: 566.3793.

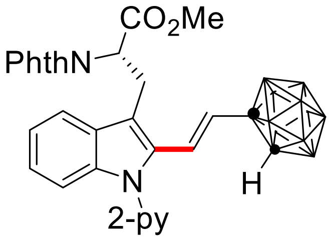

3ca

Methyl (*S*, *E*)-3-[2-(2-*o*-carboranyl-vinyl)]-1-(pyridin-2-yl)-1*H*-indol-3-yl)-2-(1,3-dioxoisoindolin-2-yl)propanoate (3ca)

The general procedure was followed using methyl (*S*)-2-(1,3-dioxoisoindolin-2-yl)-3-[1-(pyridin-2-yl)-1*H*-indol-3-yl]propanoate (**1c**) (43.9 mg, 0.1 mmol) and 1-ethynyl-*o*-carborane **2a** (16.8 mg, 0.1 mmol). Column chromatography on silica in (*n*-hexane/EtOAc = 3/2) afforded **3ca** (56.6 mg, 95%) as yellow solid. **M.P.** = 154-155 °C. **^{1}H NMR** (400 MHz, $CDCl_3$): δ = 8.59 (dd, J = 4.9, 2.0 Hz, 1H), 7.80 (ddd, J = 8.0, 7.5, 2.0 Hz, 1H), 7.75 – 7.70 (m, 2H), 7.70 – 7.63 (m, 3H), 7.39 – 7.35 (m, 1H), 7.29 (ddd, J = 7.5, 4.9, 1.0 Hz, 1H), 7.20 – 7.11 (m, 2H), 7.07 (dt, J = 7.9, 1.0 Hz, 1H), 6.75 (d, J = 16.0 Hz, 1H), 5.54 (d, J = 16.0 Hz, 1H), 5.32 – 5.25 (m, 1H), 3.95 (s, 1H), 3.86 – 3.81 (m, 2H), 3.79 (s, 3H). **^{13}C NMR** (126 MHz, $CDCl_3$): δ = 169.1 (C_q), 167.5 (C_q), 151.2 (C_q), 149.5 (CH), 138.4 (C_q), 138.1 (CH), 134.3 (C_q), 131.5 (C_q), 127.9 (CH), 126.1 (CH), 125.5 (CH), 124.8 (CH), 123.6 (CH), 122.4 (CH), 121.6 (CH), 121.5 (CH), 119.2 (CH), 115.3 (CH), 111.1 (C_q), 73.9 (cage C_q), 61.0 (cage CH), 53.1 (CH_3), 51.87 (CH), 24.6 (CH_2). **^{11}B NMR** (128 MHz, $CDCl_3$): δ = -2.04 (1B), -4.83 (1B), -9.20 (2B), -11.37 (6B). **IR** (ATR): 1746, 1712, 1586, 1467, 1434, 1385, 1185, 1069, 716 cm^{-1}. **MS** (ESI) *m*/*z* (relative intensity): 596 (30) $[M+H]^+$. **HR-MS** (ESI): *m*/*z* calcd. for $C_{29}H_{31}B_{10}N_3O_3$ $[M+H]^+$: 596.3318, found: 596.3324.

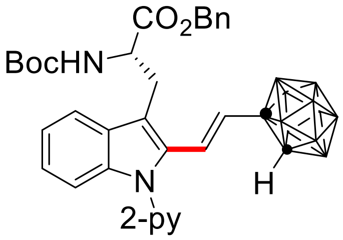

3da

Benzyl (*S*, *E*)-3-[2-(2-*o*-carboranyl-vinyl)]-1-(pyridin-2-yl)-1*H*-indol-3-yl)-2-[(*tert* butoxycarbonyl) amino]propanoate (3da)

The general procedure was followed using benzyl N_α-(*tert*-butoxycarbonyl)-1-(pyridin-2-yl)-*L*-tryptophanate (**1d**) (47.1 mg, 0.1 mmol) and 1-ethynyl-*o*-carborane **2a** (16.8 mg, 0.1 mmol). Column chromatography on silica in (*n*-hexane/EtOAc = 4/1) afforded **3da** (59.6 mg, 93%) as yellow solid. **M.P.** = 84-86 °C . **^{1}H NMR** (400 MHz, $CDCl_3$): δ = 8.68 (dd, *J* = 5.0, 1.9 Hz, 1H), 7.88 (td, *J* = 7.7, 2.0 Hz, 1H), 7.49 (dd, *J* = 10.9, 8.1 Hz, 2H), 7.35 (ddd, *J* = 7.5, 4.9, 1.0 Hz, 1H), 7.30 – 7.10 (m, 6H), 6.85 – 6.74 (m, 3H), 6.14 (d, *J* = 16.2 Hz, 1H), 5.36 (d, *J* = 8.2 Hz, 1H), 4.97 (d, *J* = 12.1 Hz, 1H), 4.61 (d, *J* = 12.1 Hz, 1H), 4.45 (td, *J* = 8.9, 5.4 Hz, 1H), 4.29 (s, 1H), 3.27 (m, 2H), 1.42 (s, 9H). **^{13}C NMR** (126 MHz, $CDCl_3$): δ = 172.2 (C_q), 155.0 (C_q), 150.9 (C_q), 149.6 (CH), 138.4 (CH), 137.3 (C_q), 134.4 (C_q), 131.6 (C_q), 128.7 (C_q), 128.4 (CH), 128.3 (CH), 128.3 (CH), 125.8 (CH), 125.7 (CH), 124.4 (CH), 122.4 (CH), 121.5 (CH), 121.3 (CH), 118.9 (CH), 113.9 (C_q), 111.1 (CH), 80.4 (C_q), 74.3 (cage C_q), 67.7 (CH_2), 60.6 (cage CH), 54.6 (CH), 29.8 (CH_2), 28.3 (CH_3), 27.4 (CH). **^{11}B NMR** (128 MHz, $CDCl_3$): δ = -2.77 (1B), -5.09 (1B), -9.16 (2B), -11.76 (3B), -13.24 (3B). **IR** (ATR): 3053, 2924, 2596, 1697, 1588, 1469, 1454, 1352, 1172, 742 cm^{-1}. $[\alpha]_D^{20}$: 36.0 (c= 1.00, CH_2Cl_2). **MS** (ESI) *m*/*z* (relative intensity): 642 (100) $[M+H]^+$. **HR-MS** (ESI): *m*/*z* calcd. for $C_{32}H_{41}B_{10}N_3O_4$ $[M+H]^+$: 642.4100, found: 642.4107.

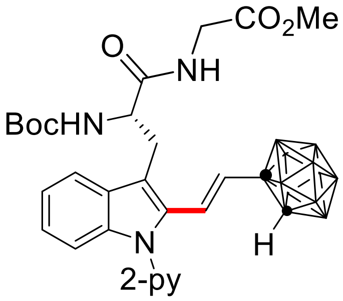

3ea

Methyl (*S*, *E*)-{3-[2-(2-*o*-carboranyl-vinyl]-1-(pyridin-2-yl)-1*H*-indol-3-yl)-2-[(*tert*-butoxycarbonyl)amino]propanoyl)glycinate (3ea).

The general procedure was followed using methyl N_α-(*tert*-butoxycarbonyl)-1-(pyridin-2-yl)-*L*-tryptophylglycinate (**1e**) (40.9 mg, 0.1 mmol) and 1-ethynyl-*o*-carborane **2a** (16.8 mg, 0.1 mmol). Column chromatography on silica in (*n*-hexane/ EtOAc = 3/2) afforded **3ea** (58.4 mg, 94%) as a white solid. **M.P.** = 115-117 °C. **^{1}H NMR** (400 MHz, $CDCl_3$): δ = 8.70 – 8.62 (dd, J = 6.9, 0.9 Hz, 1H), 7.92 (td, J = 7.8, 1.9 Hz, 1H), 7.47 (d, J = 7.8 Hz, 1H), 7.45 – 7.32 (m, 3H), 7.22 – 7.08 (m, 2H), 6.82 (d, J = 16.2 Hz, 1H), 6.33 (d, J = 16.2 Hz, 1H), 5.80 – 5.54 (m, 2H), 4.56 (s, 1H), 4.20 (s, 1H), 3.94 – 3.85 (m, 1H), 3.54 (s, 3H), 3.41 – 3.14 (m, 2H), 1.43 (s, 9H) . **^{13}C NMR** (126 MHz, $CDCl_3$): δ = 170.7 (C_q), 169.0 (C_q), 155.2 (C_q), 150.8 (C_q), 149.7 (CH), 138.6 (CH), 137.0 (C_q), 131.8 (C_q), 128.8 (C_q), 126.1 (CH), 125.3 (CH), 124.4 (CH), 122.6 (CH), 121.5 (CH), 121.4 (CH), 119.1 (CH), 113.6 (C_q), 110.9(CH), 80.3 (C_q), 74.4 (cage C_q), 60.5 (cage CH), 55.6 (CH), 52.3 (CH_3), 41.2 (CH_2), 29.8 (CH_2), 28.3 (CH_3).**^{11}B NMR** (128 MHz, $CDCl_3$): δ = -2.83 (1B), -4.85 (1B), -8.97 (2B), -11.26 (6B). **IR** (ATR): 2360, 2167, 2056, 1596, 1560, 1428, 534 cm^{-1}. **MS** (ESI) *m*/*z* (relative intensity): 622 (100) $[M]^+$. **HR-MS** (ESI): *m*/*z* calcd. for $C_{28}H_{40}B_{10}N_4O_5$ $[M+H]^+$: 622.3929, found: 622.4055.

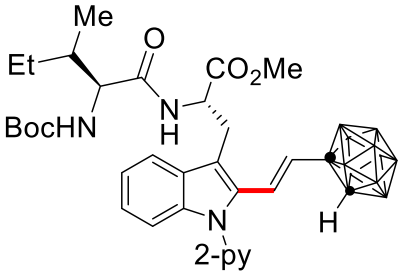

3fa

Methyl (*S*)-3-{2-[(*E*)-2-*o*-carboranyl-vinyl)]-1-(pyridin-2-yl)-1*H*-indol-3-yl)-2-{(2*S*, 3*S*)-2-[(*tert*-butoxycarbonyl) amino]-3-methylpentanamido}propanoate (3fa)

The general procedure was followed using methyl N_α-[(*tert*-butoxycarbonyl)-*L*-isoleucyl]-1-(pyridin-2-yl)-*L*-tryptophanate (**1f**) (50.8 mg, 0.1 mmol) and 1-ethynyl-*o*-carborane **2a** (16.8 mg, 0.1 mmol). Column chromatography on silica in (*n*-hexane/EtOAc = 3/2) afforded **3fa** (47.3 mg, 70%) as yellow solid. **M.P.** = 108-110 °C. **^{1}H NMR** (400 MHz, $CDCl_3$): δ = 8.67 (dd, *J* = 5.7, 2.0 Hz, 1H), 7.90 (td, *J* = 7.7, 2.0 Hz, 1H), 7.52 – 7.42 (m, 2H), 7.35 (ddd, *J* = 7.5, 4.9, 1.0 Hz, 1H), 7.29 (dt, *J* = 8.0, 1.0 Hz, 1H), 7.23 – 7.14 (m, 2H), 6.81 (d, *J* = 16.1 Hz, 1H), 6.77 (s, 1H), 6.25 (d, *J* = 16.1 Hz, 1H), 4.89 (s, 1H), 4.67 (q, *J* = 7.6 Hz, 1H), 4.49 (s, 1H), 3.97 (s, 1H), 3.41 (s, 3H), 3.41 – 3.22 (m, 2H), 1.94 – 1.83 (m, 2H), 1.45 (s, 9H), 1.33 – 1.19 (m, 1H), 0.97 – 0.85 (m, 6H). **^{13}C NMR** (126 MHz, $CDCl_3$): δ = 172.3 (C_q), 171.4 (C_q), 150.8 (C_q), 149.6 (CH), 138.5 (CH), 137.1 (C_q), 131.7 (C_q), 128.6 (C_q), 126.1 (CH), 125.7 (CH), 124.4 (CH), 122.4 (CH), 121.5 (CH), 121.3 (CH), 118.8 (CH), 113.5 (C_q), 111.2 (CH), 80.3 (C_q), 74.3 (cage C_q), 60.7 (cage CH), 59.3 (CH), 53.2 (CH), 52.5 (CH_3), 37.0 (CH), 29.4 (CH_2), 28.2 (CH_3), 24.7 (CH_2), 15.6 (CH_3), 11.5 (CH_3). **^{11}B NMR** (128 MHz, $CDCl_3$): δ = -2.74 (1B), -4.85 (1B), -8.94 (2B), -11.25 (6B). **IR** (ATR): 2963, 2565, 1713, 1652, 1588, 1519, 1469, 1436, 1366, 1173 cm^{-1}. **MS** (ESI) *m*/*z* (relative intensity): 678 (100) $[M]^+$. **HR-MS** (ESI): *m*/*z* calcd. for $C_{32}H_{48}B_{10}N_4O_5$ $[M+H]^+$: 679.4628, found: 679.4634.

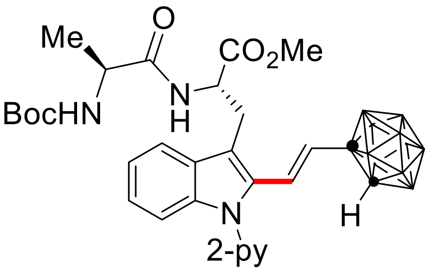

3ga

Methyl (*S*)-3-{2-((*E*)-2-*o*-carboranyl-vinyl)-1-(pyridin-2-yl)-1*H*-indol-3-yl)-2-((S)-2-[(*tert*-butoxycarbonyl) amino)]propanamido}propanoate (3ga)

The general procedure was followed using methyl N_{α}-[(*tert*-butoxycarbonyl)-*L*-alanyl)]-1-(pyridin-2-yl)-*L*-tryptophanate (**1g**) (46.6 mg, 0.1 mmol) and 1-ethynyl-*o*-carborane **2a** (16.8 mg, 0.1 mmol). Column chromatography on silica in (*n*-hexane/EtOAc = 4/1) afforded **3ga** (57.0 mg, 90%) as white solid. **M.P.** = 105-107 °C. **^{1}H NMR** (400 MHz, $CDCl_3$): δ = 8.67 (dd, *J* = 4.9, 2.0 Hz, 1H), 7.90 (td, *J* = 7.8, 2.0 Hz, 1H), 7.50 – 7.41 (m, 2H), 7.36 (ddd, *J* = 7.5, 4.9, 1.0 Hz, 1H), 7.32 – 7.26 (m, 1H), 7.25 – 7.10 (m, 2H), 6.88 (d, *J* = 8.0 Hz, 1H), 6.80 (d, *J* = 16.1 Hz, 1H), 6.14 (d, *J* = 16.1 Hz, 1H), 4.89 (s, 1H), 4.68 (q, *J* = 7.4 Hz, 1H), 4.38 (s, 1H), 4.25 – 4.01 (m, 1H), 3.44 (s, 3H), 3.24 (d, *J* = 7.3 Hz, 2H), 1.43 (s, 9H), 1.29 (dd, *J* = 7.0, 3.2 Hz, 3H). **^{13}C NMR** (126 MHz, $CDCl_3$): δ = 172.4 (C_q), 172.3 (C_q), 150.9 (C_q), 149.6 (CH), 138.5 (CH), 137.2 (C_q), 131.8 (C_q), 128.6 (C_q), 126.0 (CH), 125.9 (CH), 125.8 (CH), 124.5 (CH), 122.5 (CH), 121.4 (CH), 121.3 (CH), 118.9 (CH), 113.7 (C_q), 111.1 (CH), 80.4 (C_q), 74.2 (cage C_q), 60.8 (cage CH), 53.2 (CH), 52.6 (CH_3), 29.2 (CH_2), 28.2 (CH_3), 18.0 (CH_3).**^{11}B NMR** (128 MHz, $CDCl_3$): δ = -2.73 (1B), -5.01 (1B), -9.06 (2B), -11.45 (6B). **IR** (ATR): 2977, 2594, 1713, 1665, 1588, 1496, 1437, 1366, 1223, 1167 cm^{-1}. **MS** (ESI) *m*/*z* (relative intensity): 636 (100) $[M]^+$. **HR-MS** (ESI): *m*/*z* calcd. for $C_{29}H_{43}B_{10}N_4O_5$ $[M+H]^+$: 637.4159, found: 637.4164.

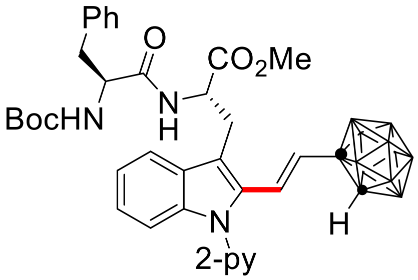

3g'a

Methyl (*S*)-3-[2-(*E*)-(2-*o*-carboranyl)-vinyl]-1-(pyridin-2-yl)-1*H*-indol-3-yl)-2-{(*S*)-2-[(*tert*-butoxycarbonyl) amino)-3-phenylpropanamido)propanoate (3g'a)

The general procedure was followed using methyl N_α-[(*tert*-butoxycarbonyl)-*L*-phenylalanyl)]-1-(pyridin-2-yl)-*L*-tryptophanate (**1g'**) (54.1 mg, 0.1 mmol) and 1-ethynyl-*o*-carborane **2a** (16.8 mg, 0.1 mmol). Column chromatography on silica in (*n*-hexane/EtOAc = 4/1) afforded **3g'a** (65.2 mg, 92%) as yellow solid. **M.P.** = 104-106 °C. **^{1}H NMR** (300 MHz, $CDCl_3$): δ = 8.72 (dd, J = 5.2, 2.0 Hz, 1H), 7.95 (td, J = 7.7, 2.0 Hz, 1H), 7.50 (d, J = 8.2 Hz, 1H), 7.40 (dd, J = 7.5, 4.5 Hz, 2H), 7.32 (dd, J = 7.7, 5.9 Hz, 3H), 7.27 – 7.14 (m, 5H), 6.86 (d, J = 16.1 Hz, 1H), 6.64 (d, J = 7.8 Hz, 1H), 6.21 (d, J = 16.1 Hz, 1H), 4.93 (s, 1H), 4.72 – 4.58 (m, 1H), 4.43 (s, 1H), 4.36 (d, J = 9.9 Hz, 1H), 3.43 (s, 3H), 3.23 (qd, J = 14.2, 7.5 Hz, 2H), 3.04 (d, J = 6.8 Hz, 2H), 1.44 (s, 9H). **^{13}C NMR** (101 MHz, $CDCl_3$): δ = 171.9 (C_q), 171.0 (C_q), 150.9 (C_q), 149.7 (CH), 138.5 (CH), 137.2 (C_q), 136.3 (C_q), 131.7 (C_q), 129.2 (CH), 128.7 (CH), 128.6 (C_q), 127.1 (CH), 125.8 (CH), 125.8 (CH), 124.5 (CH), 122.5 (CH), 121.5 (CH), 121.3 (CH), 118.81 (CH), 113.6 (C_q), 111.1 (CH), 80.5 (C_q), 74.3 (cage C_q), 60.7 (cage CH), 55.8 (CH), 53.2 (CH), 52.5 (CH_3), 38.2 (CH_2), 29.1 (CH_2), 28.2 (CH_3). **^{11}B NMR** (128 MHz, $CDCl_3$): δ = -2.67 (1B), -4.90 (1B), -8.98 (2B), -11.21 (3B), -13.04 (3B). **IR** (ATR): 2924, 2596, 2166, 1966, 1703, 1519, 1469, 1438, 1367, 744 cm^{-1}. **MS** (ESI) *m*/*z* (relative intensity): 712 (100) $[M]^+$. **HR-MS** (ESI): *m*/*z* calcd. for $C_{35}H_{47}B_{10}N_4O_5$ $[M+H]^+$: 713.4472, found: 713.4478.

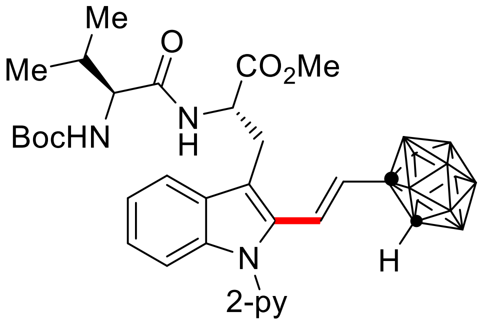

3ha

Methyl (*S*)-3-(2-((*E*)-2-*o*-carboranyl-vinyl)-1-(pyridin-2-yl)-1*H*-indol-3-yl)-2-((*S*)-2-((*tert*-butoxycarbonyl) amino)-3-methylbutanamido)propanoate (3ha)

The general procedure was followed using methyl N_{α}-[(*tert*-butoxycarbonyl)-*L*-valyl)]-1-(pyridin-2-yl)-*L*-tryptophanate (**1h**) (49.3 mg, 0.1 mmol) and 1-ethynyl-*o*-carborane **2a** (16.8 mg, 0.1 mmol). Column chromatography on silica in (*n*-hexane/EtOAc = 4/1) afforded **3ha** (52.9 mg, 80%) as white solid. **M.P.** = 115-117 °C. **^{1}H NMR** (400 MHz, $CDCl_3$): δ = 8.67 (dd, J = 6.8, 0.8 Hz, 1H), 7.90 (ddd, J = 8.0, 7.5, 2.0 Hz, 1H), 7.49 – 7.43 (m, 2H), 7.35 (ddd, J = 7.5, 4.9, 1.0 Hz, 1H), 7.29 (dt, J = 8.0, 1.0 Hz, 1H), 7.25 – 7.11 (m, 2H), 6.81 (d, J = 16.1 Hz, 1H), 6.20 (d, J = 16.1 Hz, 1H), 4.93 (d, J = 8.6 Hz, 1H), 4.68 (q, J = 7.6 Hz, 1H), 4.45 (s, 1H), 3.97 – 3.87 (m, 1H), 3.42 (s, 3H), 3.23 (d, J = 7.5 Hz, 2H), 2.14 (dt, J = 7.0, 5.9 Hz, 2H), 1.44 (s, 9H), 0.93 (d, J = 6.8 Hz, 3H), 0.89 – 0.80 (d, J = 7.1 Hz, 3H). **^{13}C NMR** (126 MHz, $CDCl_3$): δ = 172.3 (C_q), 171.4 (C_q), 155.8 (C_q), 150.9 (C_q), 149.6 (CH), 138.4 (CH), 137.1 (C_q), 131.7 (C_q), 128.6 (C_q), 126.0 (CH), 125.8 (CH), 124.4 (CH), 122.4 (CH), 121.5 (CH), 121.3 (CH), 118.8 (CH), 113.5 (C_q), 111.2 (CH), 80.3 (C_q), 74.3 (cage C_q), 60.7 (cage CH), 59.9 (CH), 53.2 (CH), 52.6 (CH_3), 30.5 (CH), 29.3 (CH_2), 28.2 (CH_3), 19.2 (CH_3), 17.5 (CH_3). **^{11}B NMR** (128 MHz, $CDCl_3$): δ = -2.67 (1B), -4.71 (1B), -8.92 (2B), -11.18 (6B). **IR** (ATR): 2961, 2594, 1656, 1499, 1469, 1437, 1366, 1214, 1174, 1018 cm^{-1}. **MS** (ESI) *m*/*z* (relative intensity): 664 (100) $[M]^{+}$. **HR-MS** (ESI): *m*/*z* calcd. for $C_{31}H_{47}B_{10}N_4O_5$ $[M+H]^{+}$: 665.4472, found: 665.4478.

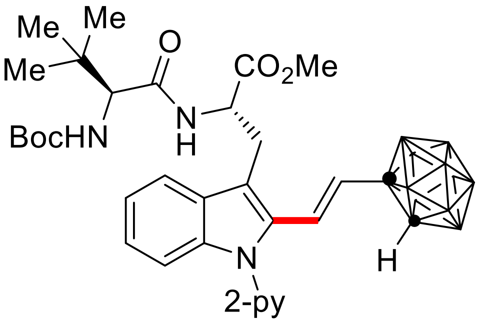

3ia

Methyl (*S*)-3-(2-((*E*)-2-*o*-carboranyl-vinyl)-1-(pyridin-2-yl)-1*H*-indol-3-yl)-2-((*S*)-2-((*tert*-butoxycarbonyl) amino)-3,3-dimethylbutanamido)propanoate (3ia)

The general procedure was followed using methyl N_α-((*S*)-2-[(tert-butoxycarbonyl)amino)]-3,3-dimethylbutanoyl)-1-(pyridin-2-yl)-*L*-tryptophanate (**1i**) (50.8 mg, 0.1 mmol) and 1-ethynyl-*o*-carborane **2a** (16.8 mg, 0.1 mmol). Column chromatography on silica in (*n*-hexane/ EtOAc = 4/1) afforded **3ia** (62.9 mg, 93%) as white solid. **M.P.** = 121-123 °C. ^{1}**H NMR** (400 MHz, $CDCl_3$): δ = 8.67 (dd, *J* = 5.0, 1.9 Hz, 1H), 7.89 (td, *J* = 7.8, 1.9 Hz, 1H), 7.46 (td, *J* = 8.3, 1.0 Hz, 2H), 7.35 (ddd, *J* = 7.5, 5.0, 1.0 Hz, 1H), 7.28 (d, *J* = 8.0 Hz, 1H), 7.22 – 7.13 (m, 2H), 6.83 (d, *J* = 16.1 Hz, 1H), 6.55 (d, *J* = 7.8 Hz, 1H), 6.16 (d, *J* = 16.1 Hz, 1H), 5.11 (d, *J* = 8.7 Hz, 1H), 4.70 – 4.62 (m, 1H), 4.49 (s, 1H), 3.82 (d, *J* = 8.9 Hz, 1H), 3.40 (s, 3H), 3.24 (dd, *J* = 7.5, 4.9 Hz, 2H), 1.43 (s, 9H), 0.98 (s, 9H). ^{13}**C NMR** (126 MHz, $CDCl_3$): δ = 172.3 (C_q), 170.8 (C_q), 155.7 (C_q), 150.9 (C_q), 149.6 (CH), 138.4 (CH), 137.2 (C_q), 131.6 (C_q), 128.6 (C_q), 126.0 (CH), 125.6 (CH), 124.5 (CH), 122.4 (CH), 121.5 (CH), 121.3 (CH), 118.8 (CH), 113.6 (C_q), 111.1 (CH), 80.1 (C_q), 74.3 (cage C_q), 62.6 (CH), 60.7 (cage CH), 53.2 (CH), 52.5 (CH_3), 34.4 (CH_2), 29.2 (C_q), 28.3 (CH_3), 26.6 (CH_3). ^{11}**B NMR** (128 MHz, $CDCl_3$): δ = -2.65 (1B), -4.79 (1B), -8.97 (2B), -10.99 (6B). **IR** (ATR): 3051, 2976, 2588, 1656, 1588, 1505, 1469, 1367, 1223, 1174 cm^{-1}. **MS** (ESI) *m*/*z* (relative intensity): 678 (100) $[M]^+$. **HR-MS** (ESI): *m*/*z* calcd. for $C_{32}H_{49}B_{10}N_4O_5$ $[M+H]^+$: 679.4628, found: 679.4634.

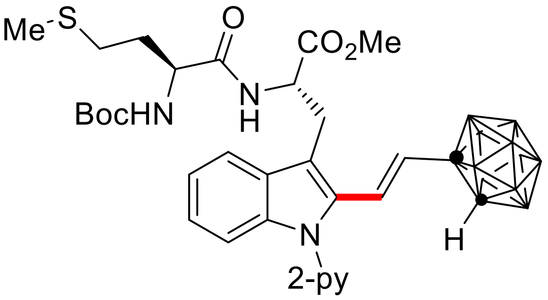

3ja

Methyl (*S*)-3-(2-((*E*)-2-*o*-carboranyl-vinyl)-1-(pyridin-2-yl)-1*H*-indol-3-yl)-2-((*S*)-2-((*tert*-butoxycarbonyl) amino)-4-(methylthio) butanamido) propanoate (3ja)

The general procedure was followed using methyl N_{α}-[(*tert*-butoxycarbonyl)-*L*-methionyl)]-1-(pyridin-2-yl)-*L*-tryptophanate (**1j**) (52.6 mg, 0.1 mmol) and 1-ethynyl-*o*-carborane **2a** (16.8 mg, 0.1 mmol). Column chromatography on silica in (*n*-hexane/EtOAc = 4/1) afforded **3ja** (39.6 mg, 57% yield) as pale-yellow solid. **M.P.** = 89-91 °C. **^{1}H NMR** (300 MHz, $CDCl_3$): δ = 8.71 (dd, *J* = 5.0, 2.0 Hz, 1H), 7.95 (td, *J* = 7.8, 2.0 Hz, 1H), 7.50 (d, *J* = 7.8 Hz, 2H), 7.41 (dd, *J* = 7.5, 5.0 Hz, 1H), 7.34 (d, *J* = 8.0 Hz, 1H), 7.23 (t, *J* = 8.4 Hz, 2H), 6.96 (d, *J* = 8.2 Hz, 1H), 6.86 (d, *J* = 16.1 Hz, 1H), 6.13 (d, *J* = 16.1 Hz, 1H), 5.14 (s, 1H), 4.75 (m, 1H), 4.38 (s, 1H), 4.27 (d, *J* = 7.0 Hz, 1H), 3.52 (s, 3H), 3.31 (d, *J* = 7.2 Hz, 2H), 3.00 – 2.88 (m, 1H), 2.56 (t, *J* = 7.1 Hz, 3H), 2.09 (s, 3H), 1.48 (s, 9H).**^{13}C NMR** (101 MHz, $CDCl_3$): δ = 172.1 (C_q), 171.3 (C_q), 155.4 (C_q), 150.9 (C_q), 149.7 (CH), 138.5 (CH), 137.3 (C_q), 131.8 (C_q), 128.5 (C_q), 125.9 (CH), 125.9 (CH), 124.5 (CH), 122.5 (CH), 121.5 (CH), 121.4 (CH), 118.9 (CH), 113.7 (C_q), 111.2 (CH), 80.5 (C_q), 74.2 (cage C_q), 60.8 (cage CH), 53.4 (CH), 53.1 (CH), 52.61 (CH_3), 31.3 (CH_2), 30.1 (CH_2), 28.9 (CH_2), 28.3 (CH_3), 15.2 (CH_3). **^{11}B NMR** (128 MHz, $CDCl_3$): δ = -2.61 (1B), -4.91 (1B), -9.00 (2B), -11.13 (6B). **IR** (ATR): 3049, 2976, 2565, 1663, 1558, 1507, 1469, 1366, 1226, 1167 cm^{-1}. **MS** (ESI) *m*/*z* (relative intensity): 696 (90) $[M]^+$. **HR-MS** (ESI): *m*/*z* calcd. for $C_{31}H_{47}B_{10}N_4O_5S$ $[M+H]^+$: 697.4192, found: 697.4199.

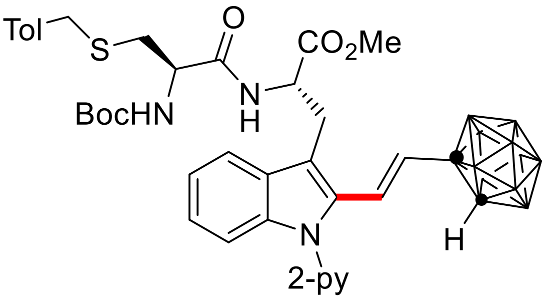

3ka

Methyl (*S*)-3-(2-((*E*)-2-*o*-carboranyl-vinyl)-1-(pyridin-2-yl)-1*H*-indol-3-yl)-2-((*R*)-2-((*tert*-butoxycarbonyl) amino)-3-((4-methylbenzyl)thio) propanamido)propanoate (3ka)

The general procedure was followed using methyl N_α-[*N*-(*tert*-butoxycarbonyl)-*S*-(4-methylbenzyl)-*L*-cysteinyl]-1-(pyridin-2-yl)-*L*-tryptophanate (**1k**) (51.1 mg, 0.1 mmol) and 1-ethynyl-*o*-carborane **2a** (16.8 mg, 0.1 mmol). Column chromatography on silica in (*n*-hexane/ EtOAc = 4/1) afforded **3ka** (57.0 mg, 74%) as white solid. **M.P.** = 115-117 °C. **^{1}H NMR** (300 MHz, $CDCl_3$): δ = 8.72 (dd, *J* = 4.9, 2.0 Hz, 1H), 7.94 (td, *J* = 7.7, 2.0 Hz, 1H), 7.54 – 7.47 (m, 2H), 7.40 (ddd, *J* = 7.5, 4.9, 1.0 Hz, 1H), 7.33 (d, *J* = 8.0 Hz, 1H), 7.27 – 7.09 (m, 7H), 6.86 (d, *J* = 16.1 Hz, 1H), 6.18 (d, *J* = 16.1 Hz, 1H), 5.31 – 5.14 (m, 1H), 4.76 – 4.65 (m, 1H), 4.38 (s, 1H), 4.30 – 4.18 (m, 1H), 3.71 (s, 2H), 3.47 (s, 3H), 3.33 – 3.27 (m, 2H), 2.87 – 2.66 (m, 2H), 2.35 (s, 3H), 1.49 (s, 9H). **^{13}C NMR** (101 MHz, $CDCl_3$): δ = 172.4 (C_q), 170.4 (C_q), 155.3 (C_q), 150.9 (C_q), 149.7 (CH), 138.4 (CH), 137.2 (C_q), 137.0 (C_q), 134.5 (C_q), 131.7 (C_q), 129.4 (CH), 128.8 (CH), 128.6 (C_q), 125.9 (CH), 125.8 (CH), 124.5 (CH), 122.4 (CH), 121.5 (CH), 121.3 (CH), 118.9 (CH), 113.6 (C_q), 111.2 (CH), 80.7 (C_q), 74.3 (cage C_q), 60.7 (cage CH), 53.6 (CH), 52.6 (CH), 52.6 (CH_3), 36.2 (CH_2), 33.4 (CH_2), 29.1 (CH_2), 28.2 (CH_3), 21.1 (CH_3).**^{11}B NMR** (128 MHz, $CDCl_3$): δ = -2.61 (1B), -4.82 (1B), -8.98 (2B), -11.13 (6B). **IR** (ATR): 3049, 2924, 2596, 1664, 1588, 1513, 1469, 1366, 1210, 1167 cm^{-1}. **MS** (ESI) *m*/*z* (relative intensity): 772 (100) $[M]^+$. **HR-MS** (ESI): *m*/*z* calcd. for $C_{37}H_{51}B_{10}N_4O_5S$ $[M+H]^+$: 773.4505, found: 773.4548.

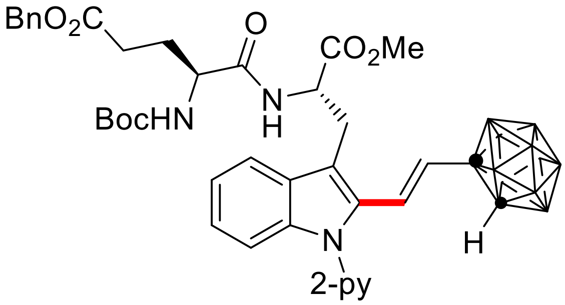

3la

Benzyl (*S*)-5-{((*S*)-3-(2-((*E)*-2-*o*-carboranyl-vinyl)-1-(pyridin-2-yl)-1*H*-indol-3-yl)-1-methoxy-1-oxopropan-2-yl) amino}-4-[(*tert*-butoxycarbonyl) amino]-5-oxopentanoate (3la)

The general procedure was followed using benzyl (*S*)-4-[(tert-butoxycarbonyl)amino]-5-{[(*S*)-1-methoxy-1-oxo-3-(1-(pyridin-2-yl)]-1H-indol-3-yl)propan-2-yl)}amino)-5-oxopentanoate (**1l**) (61.4 mg, 0.1 mmol) and 1-ethynyl-*o*-carborane **2a** (16.8 mg, 0.1 mmol). Column chromatography on silica in (*n*-hexane/EtOAc = 4/1) afforded **3la** (53.2 mg, 68%) as pale-yellow solid. **M.P.** = 84-86 °C. **^{1}H NMR** (400 MHz, $CDCl_3$): δ = 8.66 (dd, J = 5.8, 2.0 Hz, 1H), 7.89 (td, J = 7.7, 2.0 Hz, 1H), 7.49 – 7.42 (m, 2H), 7.37 – 7.28 (m, 7H), 7.22 – 7.12 (m, 2H), 6.99 (d, J = 6.1 Hz, 1H), 6.82 (d, J = 16.1 Hz, 1H), 6.09 (d, J = 16.1 Hz, 1H), 5.20 (d, J = 7.9 Hz, 1H), 4.75 – 4.64 (m, 1H), 4.34 (s, 1H), 4.23 – 4.08 (m, 1H), 3.46 (s, 3H), 3.25 (d, J = 7.3 Hz, 2H), 2.45 (m, 4H), 2.12 – 2.03 (m, 2H), 1.93 – 1.84 (m, 2H), 1.42 (s, 9H). **^{13}C NMR** (126 MHz, $CDCl_3$): δ = 173.1 (C_q), 172.1 (C_q), 171.3 (C_q), 155.6 (C_q), 150.9 (C_q), 149.6 (CH), 138.5 (CH), 137.2 (C_q), 135.6 (C_q), 131.7 (C_q), 128.6 (CH), 128.5 (C_q), 128.3 (CH), 128.2 (CH), 125.9 (CH), 125.9 (CH), 124.5 (CH), 122.5 (CH), 121.5 (CH), 121.4 (CH), 118.9 (CH), 113.7 (C_q), 111.1 (CH), 80.4 (C_q), 74.2 (cage C_q), 66.6 (CH_2), 60.8 (cage CH), 53.7 (CH), 53.1 (CH), 52.6 (CH_3), 30.4 (CH_2), 29.0 (CH_2), 28.2 (CH_3), 27.6 (CH_2). **^{11}B NMR** (128 MHz, $CDCl_3$): δ = -2.72 (1B), -5.02 (1B), -9.18 (2B), -12.62 (6B). **IR** (ATR): 2924, 2850, 2361, 1735, 1260, 1095, 794, 601 cm^{-1}. **MS** (ESI) *m*/*z* (relative intensity): 784 (90) $[M]^+$. **HR-MS** (ESI): *m*/*z* calcd. for $C_{38}H_{50}B_{10}N_4O_7$ $[M+H]^+$: 785.4683, found: 785.4750.

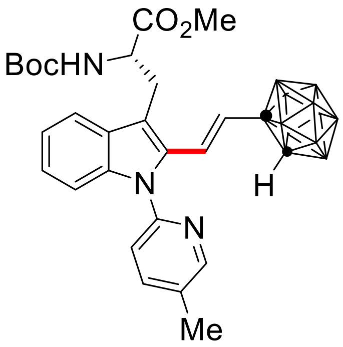

3ma

Methyl (*S*, *E*)-3-(2-(2-*o*-carboranyl-vinyl)-1-(5-methylpyridin-2-yl)-1*H*-indol-3-yl)-2-((*tert*-butoxycarbonyl) amino)propanoate (3ma)

The general procedure was followed using methyl N_α-(*tert*-butoxycarbonyl)-1-(5-methylpyridin-2-yl)-*L*-tryptophanate (**1m**) (40.9 mg, 0.1 mmol) and 1-ethynyl-*o*-carborane **2a** (16.8 mg, 0.1 mmol). Column chromatography on silica in (*n*-hexane/EtOAc = 4/1) afforded **3ma** (49.7 mg, 86 %) as a white solid. **M.P.** = 76-78 °C. **^{1}H NMR** (400 MHz, $CDCl_3$): δ = 8.49 (s, 1H), 7.69 (d, J = 8.2 Hz, 1H), 7.41 (m, 2H), 7.23 – 7.08 (m, 3H), 6.83 (d, J = 16.1 Hz, 1H), 6.12 (d, J = 16.1 Hz, 1H), 5.33 (d, J = 8.3 Hz, 1H), 4.48 – 4.37 (m, 1H), 4.34 (s, 1H), 3.40 (s, 3H), 3.33 – 3.15 (m, 2H), 2.45 (s, 3H), 1.43 (s, 9H). **^{13}C NMR** (126 MHz, $CDCl_3$): δ = 172.7 (C_q), 155.1 (C_q), 149.8 (CH), 148.6 (C_q), 139.0 (CH), 137.4 (C_q), 132.4 (C_q), 131.6 (C_q), 128.6 (C_q), 125.8 (CH), 125.5 (CH), 124.3 (CH), 121.2 (CH), 120.9 (CH), 118.9 (CH), 113.6 (C_q), 111.0 (CH), 80.4 (C_q), 74.5 (cage C_q), 60.7 (cage CH), 54.5 (CH), 52.4 (CH_3), 29.7 (CH_2), 28.3 (CH_3), 18.1 (CH_3). **^{11}B NMR** (128 MHz, $CDCl_3$): δ = -2.65 (1B), -5.00 (1B), -9.04 (2B), -11.08 (3B), -13.01 (3B). **IR** (ATR): 2586, 2147, 2027, 1984, 1691, 1482, 1453, 1364, 1151, 739 cm^{-1}. **MS** (ESI) *m/z* (relative intensity): 579 (80) $[M]^+$. **HR-MS** (ESI): *m/z* calcd. for $C_{27}H_{40}B_{10}N_3O_4$ $[M+H]^+$: 580.3944, found: 580.3949.

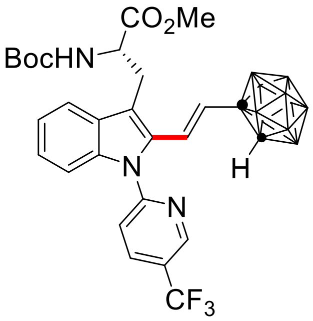

3na

Methyl (*S*, *E*)-3-[2-(2-*o*-carboranyl-vinyl)]-1-[5-(trifluoromethyl) pyridin-2-yl]-1*H*-indol-3-yl)-2-((tert-butoxycarbonyl) amino) propanoate (3na)

The general procedure was followed using methyl N_α-(*tert*-butoxycarbonyl)-1-(5-(trifluoromethyl)pyridin-2-yl)-*L*-tryptophanate (**1n**) (46.3 mg, 0.1 mmol) and 1-ethynyl-*o*-carborane **2a** (16.8 mg, 0.1 mmol). Column chromatography on silica in (*n*-hexane/EtOAc = 4/1) afforded **3na** (34.7 mg, 55 %) as a white solid. **M.P.** = 107-109 °C. **^{1}H NMR** (300 MHz, $CDCl_3$): δ = 8.97 (s, 1H), 8.15 (d, J = 10.9 Hz, 1H), 7.69 (d, J = 8.2 Hz, 1H), 7.46 (t, J = 8.2 Hz, 2H), 7.35 – 7.19 (m, 2H), 6.89 (d, J = 16.0 Hz, 1H), 6.35 (d, J = 16.1 Hz, 1H), 5.42 (d, J = 8.2 Hz, 1H), 4.55 – 4.36 (m, 2H), 3.43 (s, 3H), 3.34 – 3.17 (m, 2H), 1.47 (s, 9H). **^{13}C NMR** (126 MHz, $CDCl_3$): δ = 172.5 (C_q), 155.0 (C_q), 153.7 (C_q), 146.7 (q, $^3J_{C\text{-}F}$= 4.1 MHz, CH), 136.6 (C_q), 135.6 (q, $^3J_{C\text{-}F}$= 3.3 MHz, CH), 131.4 (C_q) , 129.2 (C_q), 126.6 (CH), 125.9 (CH), 125.0 (CH), 124.5 (q, $^2J_{C\text{-}F}$ = 32.0 Hz, C_q), 123.2 (q, $^1J_{C\text{-}F}$ = 271.3 Hz, C_q), 122.2 (CH), 120.1 (CH), 119.1 (CH), 115.4 (C_q), 111.5 (CH), 80.5 (C_q), 74.0 (cage C_q), 60.6 (cage CH), 54.6 (CH), 52.5 (CH_3), 30.9 (CH), 29.9 (CH_2), 28.3 (CH_3).**^{11}B NMR** (128 MHz, $CDCl_3$): δ = -2.61 (1B), -4.75 (1B), -8.93 (2B), -11.45 (3B), -12.57 (3B). **^{19}F NMR** (376 MHz, $CDCl_3$): δ = -61.93. **IR** (ATR): 2586, 2147, 2027, 1984, 1691, 1482, 1453, 1364, 1151, 739 cm^{-1}. **MS** (ESI) *m*/*z* (relative intensity): 633 (80) $[M]^+$. **HR-MS** (ESI): *m*/*z* calcd. for $C_{27}H_{37}B_{10}F_3N_3O_4$ $[M+H]^+$: 634.3661, found: 634.3667.

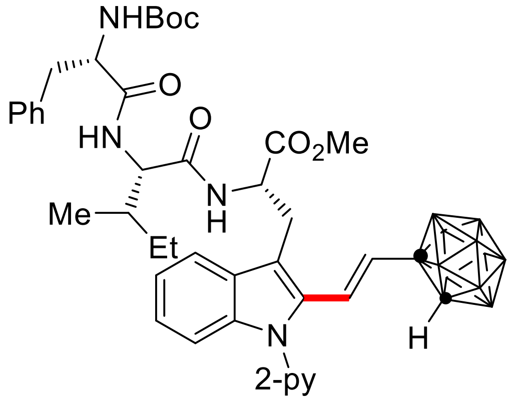

4oa

Methyl (6*S*, 9*S*, 12*S*)-6-benzyl-12-((2-((*E*)-2-*o*-carboranyl-vinyl)-1-(pyridin-2-yl)-1H-indol-3-yl) methyl)-9-((*R*)-sec-butyl)-2,2-dimethyl-4,7,10-trioxo-3-oxa-5,8,11-triazatridecan-13-oate (4oa)

The general procedure was followed using methyl N_α-(*tert*-butoxycarbonyl)-*L*-phenylalanyl-*L*-alloisoleucyl-1-(pyridin-2-yl)-*L*-tryptophanate (**1o**) (65.4 mg, 0.1 mmol) and 1-ethynyl-*o*-carborane **2a** (16.8 mg, 0.1 mmol). Column chromatography on silica in (*n*-hexane/EtOAc = 4/1) afforded **4oa** (49.3 mg, 60%) as white solid. **M.P.** = 127-129 °C. **^{1}H NMR** (400 MHz,

$CDCl_3$): δ = 8.65 (dd, J = 5.3, 2.4 Hz, 1H), 7.89 (td, J = 7.7, 1.9 Hz, 1H), 7.52 – 7.41 (m, 2H), 7.37 – 7.33 (m, 1H), 7.29 – 7.22 (m, 3H), 7.21 – 7.14 (m, 5H), 6.88 (s, 1H), 6.88 (d, J = 16.1 Hz, 1H), 6.43 (s, 1H), 6.07 (d, J = 16.1 Hz, 1H), 5.17 (d, J = 7.4 Hz, 1H), 4.68 – 4.58 (m, 1H), 4.37 (s, 1H), 4.33 – 4.21 (m, 2H), 3.46 (s, 3H), 3.23 (d, J = 7.5 Hz, 2H), 3.02 (d, J = 7.1 Hz, 2H), 1.90 – 1.80 (m, 1H), 1.40 (s, 9H), 1.37 – 1.28 (m, 1H), 1.03 – 0.91 (m, 1H), 0.86 – 0.80 (m, 6H). **^{13}C NMR** (126 MHz, $CDCl_3$): δ = 172.0 (C_q), 171.5 (C_q), 170.6 (C_q), 155.7 (C_q), 150.9 (C_q), 149.6 (CH), 138.5 (CH), 137.3 (C_q), 136.6 (C_q), 131.7 (C_q), 129.2 (CH), 128.8 (CH), 128.4 (C_q), 127.0 (CH), 126.0 (CH), 125.8 (CH), 124.5 (CH), 122.5 (CH), 121.5 (CH), 121.4 (CH), 119.0 (CH), 113.8 (C_q), 111.1 (CH), 80.5 (C_q), 74.2 (cage C_q), 60.8 (cage CH), 57.8 (CH), 53.2 (CH), 52.5 (CH_3), 37.5 (CH_2), 36.6 (CH), 28.6 (CH_2), 28.2 (CH_3), 24.5 (CH_2), 15.4 (CH_3), 11.4 (CH_3). **^{11}B NMR** (126 MHz, $CDCl_3$): δ = -2.61 (1B), -4.80 (1B), -9.10 (2B), -11.64 (6B). **IR** (ATR): 2361, 1760, 1643, 1559, 1429, 1377, 1342, 1097, 668 cm^{-1}. **MS** (ESI) m/z (relative intensity): 825 (100) $[M]^+$. **HR-MS** (ESI): m/z calcd. for $C_{41}H_{58}B_{10}N_5O_6$ $[M+H]^+$: 826,5312, found: 826,5379.

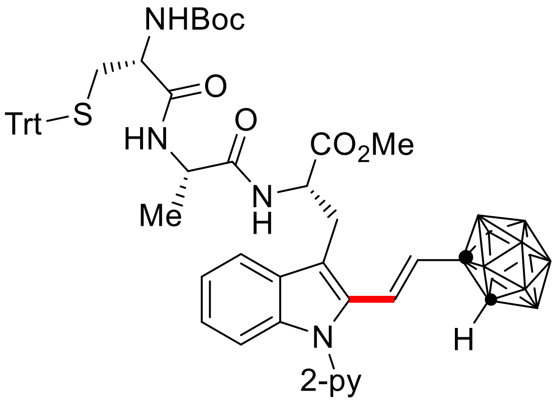

4pa

Methyl (6*R*, 9*S*, 12*S*)-12-((2-((*E*)-2-*o*-carboranyl-vinyl)-1-(pyridin-2-yl)-*1H*-indol-3-yl) methyl)-2,2,9-trimethyl-4,7,10-trioxo-6-((tritylthio)methyl)-3-oxa-5,8,11-triazatridecan-13-oate (4pa)

The general procedure was followed using methyl N_α-*N*-(*tert*-butoxycarbonyl)-*S*-trityl-*L*-cysteinyl-*L*-alanyl-1-(pyridin-2-yl)-*L*-tryptophanate (**1p**) (66.6 mg, 0.1 mmol) and 1-ethynyl-*o*-carborane **2a** (16.8 mg, 0.1 mmol). Column chromatography on silica in (*n*-hexane/EtOAc = 1/1) afforded **4pa** (65.7 mg, 67%) as white solid. **M.P.** = 185-187 °C. **^{1}H NMR** (400 MHz, $CDCl_3$): δ = 8.69 (dd, J = 5.2, 1.9 Hz, 1H), 7.90 (td, J = 7.8, 1.9 Hz, 1H), 7.51 – 7.44 (m, 2H), 7.43 – 7.34 (m, 7H), 7.31 – 7.14 (m, 12H), 7.02 (d, J = 7.8 Hz, 1H), 6.81 (d, J =

16.1 Hz, 1H), 6.28 (s, 1H), 6.03 (d, *J* = 16.1 Hz, 1H), 4.89 (s, 1H), 4.65 – 4.54 (m, 2H), 3.66 (s, 1H), 3.45 (s, 3H), 3.25 – 3.09 (m, 2H), 2.66 – 2.42 (m, 3H), 1.41 (s, 9H), 1.26 (d, *J* = 7.1 Hz, 3H). **^{13}C NMR** (126 MHz, $CDCl_3$): δ = 171.9 (C_q), 171.4 (C_q), 170.7 (C_q), 155.5 (C_q), 150.9 (C_q), 149.6 (CH), 144.3 (C_q), 138.5 (CH), 137.3 (C_q), 131.7 (C_q), 129.5 (CH), 128.5 (C_q), 128.1 (CH), 126.9 (CH), 125.9 (CH), 125.8 (CH), 124.5 (CH), 122.5 (CH), 122.4 (CH), 121.4 (CH), 119.1 (CH), 113.9 (C_q), 111.1 (CH), 80.5 (C_q), 74.2 (cage C_q), 67.3 (C_q), 60.8 (cage CH), 53.6 (CH), 53.2 (CH), 52.5 (CH_3), 48.8 (CH), 33.2 (CH_2), 28.6 (CH_2), 28.2 (CH_3), 17.2 (CH_3). **^{11}B NMR** (128 MHz, $CDCl_3$): δ = -2.45 (1B), -4.68 (1B), -8.98 (2B), -11.05 (6B). **IR** (ATR): 2361, 2184, 2043, 1649, 1488, 1446, 1156, 740, 699 cm^{-1}. **MS** (ESI) *m*/*z* (relative intensity): 981 (100) $[M]^+$. **HR-MS** (ESI): *m*/*z* calcd. for $C_{51}H_{62}B_{10}N_5O_6S$ $[M+H]^+$: 982.5346, found: 982.5414.

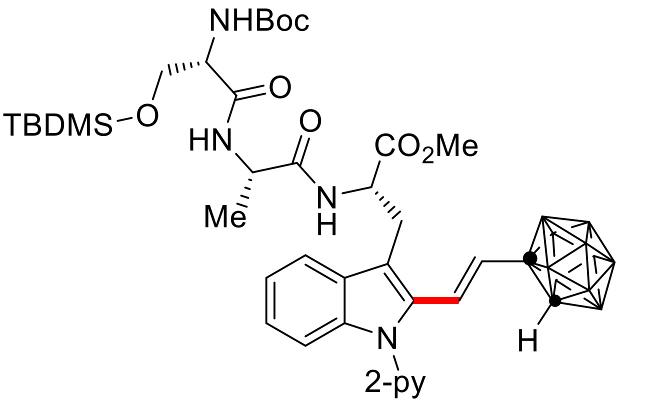

4qa

Methyl (6*S*, 9*S*, 12*S*)-12-((2-((*E*)-2-*o*-carboranyl-vinyl)-1-(pyridin-2-yl)-*1H*-indol-3-yl) methyl)-6-((*tert*-butoxycarbonyl) amino)-2,2,3,3,9-pentamethyl-7,10-dioxo-4-oxa-8,11-diaza-3-silatridecan-13-oate (4qa)

The general procedure was followed using methyl N_α-*N*-(*tert*-butoxycarbonyl)-*o*-(*tert*-butyldimethylsilyl)-*L*-seryl-*L*-alanyl-1-(pyridin-2-yl)-*L*-tryptophanate (**1q**) (66.5 mg, 0.1 mmol) and 1-ethynyl-*o*-carborane **2a** (16.8 mg, 0.1 mmol). Column chromatography on silica in (*n*-hexane/EtOAc = 1/1) afforded **4qa** (69.1 mg, 83%) as white solid. **M.P.** = 111-113°C. **^{1}H NMR** (400 MHz, $CDCl_3$): δ = 8.69 (dd, *J* = 5.7, 1.3 Hz, 1H), 7.89 (td, *J* = 7.7, 1.9 Hz, 1H), 7.53 – 7.43 (m, 2H), 7.31 – 7.25 (m, 1H), 7.22 – 7.12 (m, 1H), 7.23 – 7.12 (m, 2H), 7.01 (s, 1H), 6.83 (d, *J* = 16.1 Hz, 2H), 6.04 (d, *J* = 16.1 Hz, 1H), 5.55 (d, *J* = 6.8 Hz, 1H), 4.60 (dd, *J* = 7.5, 1.2 Hz, 1H), 4.42 (t, *J* = 7.2 Hz, 1H), 4.35 (s, 1H), 4.10 (s, 1H), 3.90 (dd, *J* = 9.9, 4.0 Hz, 1H), 3.48 (s, 3H), 3.42 (m, 1H), 3.33 – 3.18 (m, 2H), 1.45 (s, 9H), 1.32 (d, *J* = 7.1

Hz, 3H), 0.86 (s, 9H), 0.04 (d, *J* = 7.6 Hz, 6H).**^{13}C NMR** (126 MHz, $CDCl_3$): *δ* = 171.9 (C_q), 171.7 (C_q), 170.6 (C_q), 155.7 (C_q), 151.0 (C_q), 149.6 (CH), 138.5 (CH), 138.5 (CH), 137.3 (C_q), 131.8 (C_q), 128.4 (C_q), 126.0 (CH), 125.9 (CH), 124.5 (CH), 122.5 (CH), 121.5 (CH), 119.0 (CH), 113.7 (C_q) , 111.2 (CH), 80.3 (C_q), 74.2 (cage C_q), 63.0 (C_q), 60.9 (cage CH), 53.2 (CH), 52.5 (CH), 52.5 (CH), 48.9 (CH_3), 28.4 (CH_2), 28.3 (CH_3), 25.8 (CH_3), 18.1 (CH_2), 17.6 (CH_3), -5.6 (CH_3).**^{11}B NMR** (128 MHz, $CDCl_3$): *δ* = -2.57 (1B), -4.87 (1B), -9.15 (2B), -11.74 (6B). **IR** (ATR): 2534, 2361, 2002, 1761, 1596, 1560, 1428, 1036, 823 cm^{-1}. **MS** (ESI) *m*/*z* (relative intensity): 837 (100) $[M]^+$. **HR-MS** (ESI): *m*/*z* calcd. for $C_{38}H_{62}B_{10}N_5O_7Si$ $[M+H]^+$: 838.5344, found: 838.5410.

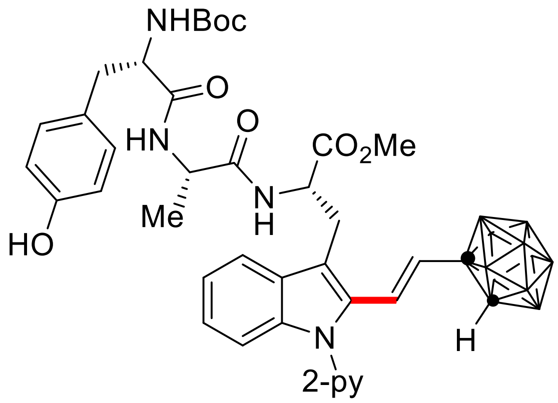

4ra

Methyl (6*S*, 9*S*, 12*S*)-12-((2-((*E*)-2-*o*-carboranyl-vinyl)-1-(pyridin-2-yl)-*1H*-indol-3-yl) methyl)-6-(4-hydroxybenzyl)-2,2,9-trimethyl-4,7,10-trioxo-3-oxa-5,8,11-triazatridecan-13-oate (4ra)

The general procedure was followed using methyl N_α-(*tert*-butoxycarbonyl)-*L*-tyrosyl-*L*-alanyl-1-(pyridin-2-yl)-*L*-tryptophanate (**1r**) (62.8 mg, 0.1 mmol) and 1-ethynyl-*o*-carborane **2a** (16.8 mg, 0.1 mmol). Column chromatography on silica in (*n*-hexane/EtOAc = 1/1) afforded **4ra** (62.1 mg, 78%) as white solid. **M.P.** = 173-175 °C. **^{1}H NMR** (300 MHz, $CDCl_3$): *δ* = 8.70 (dd, *J* = 4.9, 1.8 Hz, 1H), 7.99 (td, *J* = 7.7, 2.0 Hz, 1H), 7.58 – 7.49 (m, 1H), 7.48 – 7.36 (m, 3H), 7.24 – 6.72 (m, 2H), 7.03 (s, 1H), 6.92 – 6.72 (m, 4H), 6.55 – 6.46 (m, 2H), 6.23 (s, 1H), 5.94 (d, *J* = 16.1 Hz, 1H), 5.29 (d, *J* = 7.7 Hz, 1 H), 4.63 (d, *J* = 7.3 Hz, 1H), 4.40 – 4.29 (m, 1H), 4.22 (s, 2H), 3.62 (s, 3H), 3.37 – 3.19 (m, 2H), 2.97 – 2.85 (m, 1H), 2.82 – 2.71 (m, 1H), 1.45 (s, 9H), 1.27 (d, *J* = 7.0 Hz, 3H) .**^{13}C NMR** (126 MHz, $CDCl_3$): *δ* = 171.9 (C_q), 171.7 (C_q), 171.4 (C_q), 155.6 (C_q), 155.3 (C_q), 150.8 (C_q), 149.5 (CH), 139.0 (CH), 137.5 (C_q), 131.7 (C_q), 130.1 (CH), 128.5 (C_q), 127.5 (C_q), 126.1 (CH), 125.8 (CH),

124.7 (CH), 122.9 (CH), 121.8 (CH), 121.7 (CH), 119.1 (CH), 115.6 (CH), 114.5 (C_q), 110.9 (CH), 80.4 (C_q), 74.1 (cage C_q), 60.9 (cage CH), 56.0 (CH), 53.6 (CH), 52.7 (CH), 48.8 (CH_3), 37.5 (CH_2), 30.9 (CH_3), 28.3 (CH_3), 28.0 (CH_2). **^{11}B NMR** (128 MHz, $CDCl_3$): δ = -2.31 (1B), -4.46 (1B), -9.09 (2B), -11.25 (6B). **IR** (ATR): 3400, 3050, 2595, 1648, 1516, 1469, 1437, 1366, 1221, 1171 cm^{-1}. **MS** (ESI) *m/z* (relative intensity): 799 (100) $[M]^+$. **HR-MS** (ESI): *m/z* calcd. for $C_{38}H_{52}B_{10}N_5O_7$ $[M+H]^+$: 800.4792, found: 800.4859.

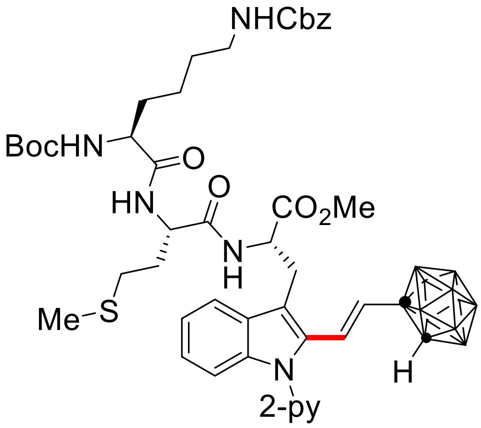

4sa

Methyl (9*S*, 12*S*, 15*S*)-15-([2-((*E*)-2-*o*-carboranyl-vinyl)-1-(pyridin-2-yl)-*1H*-indol-3-yl) methyl)-9-((*tert*-butoxycarbonyl) amino]-12-(2-(methylthio) ethyl)-3,10,13-trioxo-1-phenyl-2-oxa-4,11,14-triazahexadecan-16-oate (4sa)

The general procedure was followed using methyl N_α-N_6-[(benzyloxy)carbonyl]-N_2-(*tert*-butoxycarbonyl)-*L*-lysyl-*L*-methionyl-1-(pyridin-2-yl)-*L*-tryptophanate (**1s**) (78.7 mg, 0.1 mmol) and 1-ethynyl-*o*-carborane **2a** (16.8 mg, 0.1 mmol). Column chromatography on silica in (*n*-hexane/EtOAc = 3/2) afforded **4sa** (51.6 mg, 54%) as white solid. **M.P.** = 91-93 °C. **^{1}H NMR** (400 MHz, $CDCl_3$): δ = 8.67 (dd, *J* = 4.7, 1.3 Hz, 1H), 7.89 (td, *J* = 7.7, 1.9 Hz, 1H), 7.47 (dd, *J* = 7.5, 1.3 Hz, 1H), 7.42 (d, *J* = 8.1 Hz, 1H), 7.39 – 7.22 (m, 7H), 7.23 – 7.07 (m, 3H), 6.98 (s, 1H), 6.83 (d, *J* = 16.0 Hz, 1H), 5.95 (d, *J* = 16.0 Hz, 1H), 5.34 (d, *J* = 7.1 Hz, 1H), 5.06 (s, 1H), 4.73 – 4.65 (m, 1H), 4.59 – 4.49 (m, 1H), 4.31 (s, 1H), 3.97 (s, 1H), 3.49 (s, 3H), 3.31 – 3.22 (m, 2H), 3.09 – 3.14 (m, 2H), 2.52 (t, *J* = 7.0 Hz, 3H), 2.08 – 1.98 (m, 6H), 1.96 – 1.88 (m, 2H), 1.70 (s, 2H), 1.42 (s, 9H), 1.27 (d, *J* = 12.3 Hz, 2H). **^{13}C NMR** (126 MHz, $CDCl_3$): δ = 172.4 (C_q), 171.9 (C_q), 170.8 (C_q), 156.7 (C_q), 156.0 (C_q), 150.9 (C_q), 149.7 (CH), 138.5 (C_q), 137.5 (C_q), 136.5 (C_q), 131.8 (CH), 128.5 (C_q), 128.3 (CH), 128.1 (CH), 128.0 (CH), 126.0 (CH), 125.8 (CH), 124.5 (CH), 122.6 (CH), 121.5 (CH), 121.5 (CH), 119.0 (CH), 113.8 (C_q), 111.1 (CH), 80.3 (C_q), 74.1 (cage C_q), 66.6 (CH_2), 60.9 (cage

CH), 54.4 (CH), 53.1 (CH), 52.6 (CH_3), 52.1 (CH), 40.0 (CH_2), 31.1 (CH_2), 30.5 (CH_2), 30.0 (CH_2), 29.3 (CH_2), 28.3 (CH_3), 28.3 (CH_2), 22.3 (CH_2), 15.0 (CH_3). **^{11}B NMR** (128 MHz, $CDCl_3$): δ = -2.25 (1B), -4.55 (1B), -8.93 (2B), -10.94 (6B). **IR** (ATR): 2956, 2594, 2362, 1982, 1700, 1650, 1520, 1231, 754 cm^{-1}. **MS** (ESI) *m*/*z* (relative intensity): 958 (90) $[M]^{+}$. **HR-MS** (ESI): *m*/*z* calcd. for $C_{45}H_{65}B_{10}N_5O_8S$ $[M+H]^{+}$: 959.5510, found: 959.5577.

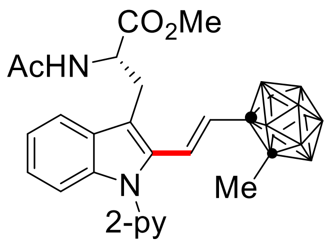

5ab

Methyl (*S*, *E*)-2-acetamido-3-{2-[2-(2-methyl-*o*-carboranyl)-vinyl]-1-[pyridin-2-yl]-*1H*-indol-3-yl} propanoate (5ab)

The general procedure was followed using methyl N_α-acetyl-1-(pyridin-2-yl)-*L*-tryptophanate (**1a**) (33.7 mg, 0.1 mmol) and 1-ethynyl-2-methyl-*o*-carborane **2b** (18.3 mg, 0.1 mmol). Column chromatography on silica in (*n*-hexane/EtOAc = 4/1) afforded **5ab** (47.3 mg, 91%) as pale-yellow solid. **M.P.** = 129-131 °C. **^{1}H NMR** (400 MHz, $CDCl_3$): δ = 8.65 (dd, *J* = 6.8, 0.8 Hz, 1H), 7.90 (ddd, *J* = 8.0, 7.5, 2.0 Hz,1H), 7.59 (dd, *J* = 1.5, 0.8 Hz, 1H), 7.41 – 7.33 (m, 2H), 7.28 (dt, *J* = 8.0, 1.0 Hz, 1H), 7.25 – 7.15 (m, 2H), 6.98 (d, *J* = 16.0 Hz, 1H), 6.06 (d, *J* = 7.9 Hz, 1H), 5.59 (d, *J* = 16.0 Hz, 1H), 4.89 (ddd, *J* = 8.0, 7.2, 5.9 Hz, 1H), 3.60 (s, 3H), 3.45 – 3.26 (m, 2H), 1.96 (s, 3H), 1.85 (s, 3H). **^{13}C NMR** (101 MHz, $CDCl_3$): δ = 172.2 (C_q), 169.6 (C_q), 151.4 (C_q), 149.8 (CH), 138.7 (CH), 138.4 (C_q), 131.8 (C_q), 129.1 (CH), 128.4 (C_q), 125.0 (CH), 123.2 (CH), 122.8 (CH), 121.9 (CH), 121.7 (CH), 119.4 (CH), 115.6 (C_q), 110.9 (CH), 78.1 (cage C_q), 75.7 (cage C_q), 52.9 (CH), 52.6 (CH_3), 28.1 (CH_2), 23.2 (CH_3), 23.2 (CH_3). **^{11}B NMR** (128 MHz, $CDCl_3$): δ = -3.78 (1B), -5.34 (1B), -10.11 (8B). **IR** (ATR): 2955, 2587, 1746, 1656, 1587, 1436, 1371, 1220, 743 cm^{-1}. **MS** (ESI) *m*/*z* (relative intensity): 521 (90) $[M]^{+}$. **HR-MS** (ESI): *m*/*z* calcd. for $C_{24}H_{34}B_{10}N_3O_3$ $[M+H]^{+}$: 522.3525, found: 522.3530.

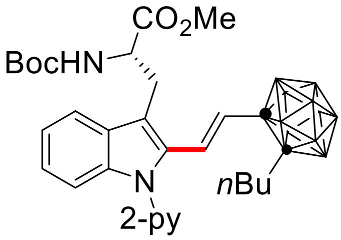

5bc

Methyl (*S,E*)-3-{2-[2-(2-*n*-butyl-*o*-carboranyl)-vinyl]-1-[(pyridin-2-yl)-1*H*-indol-3-yl]-2-[(*tert*-butoxycarbonyl)] amino}propanoate (5bc)

The general procedure was followed using methyl N_α-(*tert*-butoxycarbonyl)-1-(pyridin-2-yl)-*L*-tryptophanate (**1b**) (39.5 mg, 0.1 mmol) and 1-ethynyl-2-butyl-*o*-carborane **2c** (22.5 mg, 0.1 mmol). Column chromatography on silica in (*n*-hexane/EtOAc = 4/1) afforded **5bc** (52.5 mg, 86%) as yellow solid. **M.P.** = 103-105 °C. **^{1}H NMR** (400 MHz, $CDCl_3$): δ = 8.64 (dd, J = 4.9, 1.2 Hz, 1H), 7.89 (td, J = 7.8, 2.0 Hz, 1H), 7.60 (d, J = 7.8 Hz, 1H), 7.41 – 7.32 (m, 2H), 7.28 (dt, J = 8.0, 1.0 Hz, 1H), 7.23 – 7.14 (m, 2H), 7.00 (d, J = 16.0 Hz, 1H), 5.61 (d, J = 16.0 Hz, 1H), 5.14 (d, J = 8.6 Hz, 1H), 4.66 – 4.53 (m, 1H), 3.61 (s, 3H), 3.40 – 3.24 (m, 2H), 2.06 – 1.96 (m, 2H), 1.46 – 1.30 (m, 10H), 1.30 – 1.09 (m, 3H), 0.87 (t, J = 7.3 Hz, 3H). **^{13}C NMR** (126 MHz, $CDCl_3$): δ = 172.5 (C_q), 155.0 (C_q), 151.4 (C_q), 149.7 (CH), 138.6 (CH), 138.3 (C_q), 131.7 (C_q), 129.6 (CH), 128.4 (C_q), 124.9 (CH), 122.9 (CH), 122.7 (CH), 121.8 (CH), 121.6 (CH), 119.6 (CH), 115.8 (C_q), 110.8 (CH), 81.0 (C_q), 80.1 (cage C_q), 79.8 (cage C_q), 54.2 (CH), 52.4 (CH_3), 35.0 (CH_2), 31.7 (CH_2), 28.6 (CH_2), 28.2 (CH_3), 22.3 (CH_2), 13.7 (CH_3). **^{11}B NMR** (128 MHz, $CDCl_3$): δ = -4.37 (2B), -10.48 (8B). **IR** (ATR): 2958, 2566, 1713, 1587, 1468, 1366, 1170, 1031, 742 cm^{-1}. **MS** (ESI) *m*/*z* (relative intensity): 621 (90) $[M]^+$. **HR-MS** (ESI): *m*/*z* calcd. for $C_{30}H_{46}B_{10}N_3O_4$ $[M+H]^+$: 622.4413, found: 622.4448.

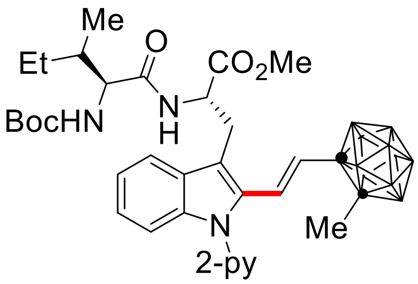

5fb

Methyl (*S*)-3-{2-[(*E*)-2(2-methyl-*o*-carboranyl-vinyl]-1-(pyridin-2-yl)-*1H*-indol-3-yl)-2-((*2S*, *3S*)-2-[(*tert*-butoxycarbonyl) amino]-3-methylpentanamido} propanoate (5fb)

The general procedure was followed using methyl N_α-[(*tert*-butoxycarbonyl)-*L*-isoleucyl]-1-(pyridin-2-yl)-*L*-tryptophanate (**1f**) (50.7 mg, 0.1 mmol) and 1-ethynyl-2-methyl-*o*-carborane **2b** (18.3 mg, 0.1 mmol). Column chromatography on silica in (*n*-hexane/EtOAc = 4/1) afforded **5fb** (50.4 mg, 73%) as white solid. **M.P.** = 114-116 °C. **^{1}H NMR** (400 MHz, $CDCl_3$): δ = 8.63 (dd, *J* = 6.8, 0.9 Hz, 1H), 7.89 (ddd, *J* = 8.0, 7.5, 1.9 Hz, 1H), 7.67 – 7.60 (m, 1H), 7.41 – 7.32 (m, 2H), 7.29 (dt, *J* = 8.0, 0.9 Hz, 1H), 7.23 – 7.17 (m, 2H), 6.99 (d, *J* = 16.0 Hz, 1H), 6.59 (d, *J* = 7.9 Hz, 1H), 5.72 (d, *J* = 16.0 Hz, 1H), 4.99 – 4.78 (m, 2H), 3.95 (s, 1H), 3.57 (s, 3H), 3.40 – 3.23 (m, 2H), 1.89 (s, 3H), 1.85 (s, 2H), 1.42 (s, 9H), 1.33 – 1.16 (m, 1H), 0.87 – 0.78 (m, 6H).**^{13}C NMR** (126 MHz, $CDCl_3$): δ = 172.0 (C_q), 171.2 (C_q), 155.6 (C_q), 151.2 (C_q), 149.7 (CH), 138.6 (CH), 138.1 (C_q), 131.6 (C_q), 129.4 (CH), 128.4 (C_q), 124.9 (CH), 123.3 (CH), 122.6 (CH), 121.7 (CH), 121.6 (CH), 119.4 (CH), 115.2 (C_q), 111.0 (CH), 80.0 (C_q), 78.2 (cage C_q), 75.8 (cage C_q), 59.2 (CH), 52.9 (CH), 52.5 (CH_3), 37.1 (CH), 28.4 (CH_2), 28.3 (CH_3), 24.4 (CH_2), 23.2 (CH_3), 15.4 (CH_3), 11.5 (CH_3).**^{11}B NMR** (128 MHz, $CDCl_3$): δ = -4.06 (1B), -5.41 (1B), -10.31 (8B). **IR** (ATR): 2962, 2584, 1764, 1684, 1650, 1469, 1366, 1175, 742 cm^{-1}. **MS** (ESI) *m*/*z* (relative intensity): 692 (90) $[M]^+$. **HR-MS** (ESI): *m*/*z* calcd. for $C_{33}H_{51}B_{10}N_4O_5$ $[M+H]^+$: 693.4785, found: 693.4791.

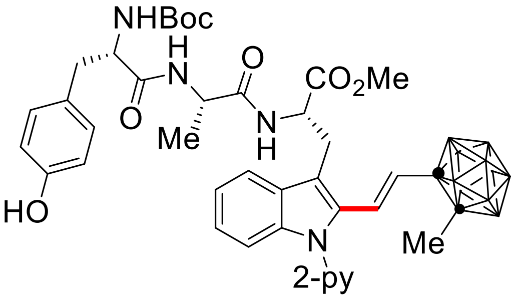

5rb

Methyl (6*S*, 9*S*, 12*S*)-12-((2-((*E*)-2(2-methyl-*o*-carboranyl-vinyl)-1-(pyridin-2-yl)-*1H*-indol-3-yl) methyl)-6-(4-hydroxybenzyl)-2,2,9-trimethyl-4,7,10-trioxo-3-oxa-5,8,11-triazatridecan-13-oate (5sb)

The general procedure was followed using methyl N_α-(*tert*-butoxycarbonyl)-*L*-tyrosyl-*L*-alanyl-1-(pyridin-2-yl)-*L*-tryptophanate (**1r**) (62.8 mg, 0.1 mmol) and 1-ethynyl-2-methyl-*o*-carborane **2b** (18.3 mg, 0.1 mmol). Column chromatography on silica in (*n*-hexane/EtOAc = 3/2) afforded **5sb** (58.4 mg, 72%) as white solid. **M.P.** = 161-163 °C. **^{1}H NMR** (400 MHz, $CDCl_3$): δ = 8.64 (dd, *J* = 6.9, 0.8 Hz, 1H), 7.94 (td, *J* = 7.7, 2.0 Hz, 1H), 7.63 – 7.56 (m, 1H), 7.50 (s, 1H), 7.43 – 7.29 (m, 3H), 7.23 – 7.16 (m, 2H), 6.99 (d, *J* = 16.0 Hz, 1H), 6.71 (d, *J* = 8.2 Hz, 2H), 6.44 – 6.35 (m, 2H), 6.20 (s, 2H), 5.68 (d, *J* = 16.0 Hz, 1H), 5.37 (s, 1H),

4.65 (q, J = 7.2 Hz, 1H), 4.36 – 4.25 (m, 1H), 4.15 (s, 2H), 3.66 (s, 3H), 3.40 – 3.25 (m, 2H), 2.80 (dd, J = 13.7, 6.3 Hz, 1H), 2.60 (dd, J = 13.7, 7.9 Hz, 2H), 2.15 (s, 3H), 1.40 (s, 9H), 1.18 (d, J = 7.0 Hz, 3H). **^{13}C NMR** (126 MHz, $CDCl_3$): δ = 171.7 (C_q), 171.3 (C_q), 155.5 (C_q), 155.5 (C_q), 155.4 (C_q), 151.0 (C_q), 149.6 (CH), 139.2 (CH), 138.0 (C_q), 131.8 (C_q), 130.0 (CH), 129.3 (CH), 128.4 (C_q), 127.4 (C_q), 125.1 (CH), 123.7 (CH), 123.0 (CH), 122.1 (CH), 121.9 (CH), 119.5 (CH), 115.7 (C_q), 115.5 (CH), 110.7 (CH), 80.3 (C_q), 78.1 (cage C_q), 76.0 (cage C_q), 56.1 (CH), 53.7 (CH), 52.7 (CH_3), 48.6 (CH), 37.7 (CH_2), 28.3 (CH_3), 27.4 (CH_2), 23.2 (CH_3), 17.8 (CH_3). **^{11}B NMR** (128 MHz, $CDCl_3$): δ = -5.29 (2B), -10.10 (8B). **IR** (ATR): 2547, 2361, 2167, 1643, 1515, 1232, 1146, 726, 546 cm^{-1}. **MS** (ESI) m/z (relative intensity): 813 (100) $[M]^+$. **HR-MS** (ESI): m/z calcd. for $C_{39}H_{54}B_{10}N_5O_7$ $[M+H]^+$: 814.4948, found: 814.4952.

Late-Stage C–7 Amidation of 3da

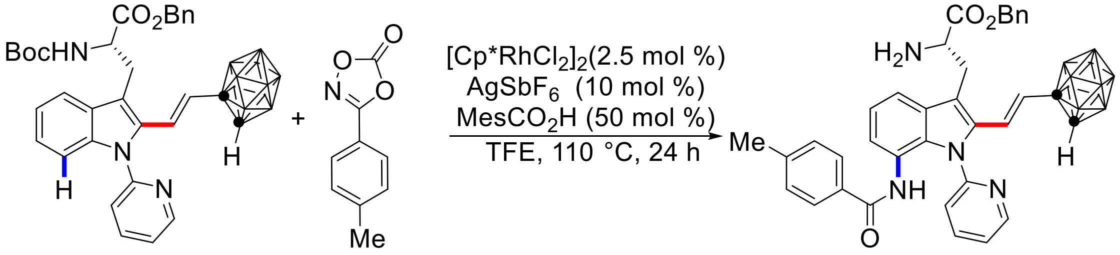

3da **6**: 52%

Benzyl (*S*,*E*)-2-amino-3-[2-(2-*o*-carboranyl-vinyl)-7-(4-methylbenzamido)]-1-(pyridin-2-yl)-*1H*-indol-3-yl)propanoate (6)

Alkenylated tryptophan **3da** (63.9 mg, 0.1 mmol**)**, 3-(*p*-tolyl)-1,4,2-dioxazol-5-one (32.6 mg, 0.2 mmol), $[Cp^*RhCl_2]_2$ (1.5 mg, 2.5 mol %), $AgSbF_6$ (3.4 mg, 10 mol %), and $MesCO_2H$ (8.3 mg, 50 mol %) in TFE (1.0 mL) was stirred at 100 °C for 24 h. After cooling to room temperature, the solvent was removed in vacuo. Column chromatography on silica gel (*n*-hexane/ EtOAc = 2/1) followed by gel permeation chromatography afforded to the isolation of product **6** (35.0 mg, 52%) as a yellow solid. **M.P.** 169-171 °C. **^{1}H NMR** (400 MHz, $CDCl_3$): δ = 8.66 (dd, J = 4.9, 2.8 Hz, 1H), 7.88 (ddd, J = 8.0, 7.5, 2.0 Hz, 1H), 7.48 – 7.42 (m, 2H), 7.37 (ddd, J = 7.5, 4.9, 1.0 Hz, 1H), 7.23 – 7.14 (m, 5H), 7.13 – 7.03 (m, 5H), 6.87 – 6.83 (m, 2H), 6.77 (d, J = 16.1 Hz, 1H), 6.66 (s, 1H), 6.10 (d, J = 16.1 Hz, 1H), 5.69 (d, J = 7.8 Hz, 1H), 4.98 (d, J = 12.1 Hz, 1H), 4.78 – 4.68 (m, 2H), 4.19 (s, 1H), 3.42 – 3.22 (m, 2H), 2.29 (s, 3H). **^{13}C NMR** (126 MHz, $CDCl_3$): δ = 172.6 (C_q), 154.9 (C_q), 150.9 (C_q), 149.6 (CH), 138.5 (CH), 137.4 (C_q), 135.2 (C_q), 134.4 (C_q), 134.1 (C_q), 131.9 (C_q), 129.9 (CH), 128.6 (C_q), 128.5 (CH), 128.4 (CH), 128.3 (CH), 126.0 (CH), 125.7 (CH), 124.5 (CH), 122.5

(CH), 121.5 (CH), 121.4 (CH), 121.3 (CH), 119.0 (CH), 113.9 (C_q), 111.1 (CH), 74.2 (cage C_q), 67.8 (CH_2), 60.8 (cage CH), 54.0 (CH), 29.3 (CH_2), 20.8 (CH_3). **^{11}B NMR** (128 MHz, $CDCl_3$): δ = -2.65 (1B), -4.88 (1B), -9.16 (2B), -11.60 (2B), -12.80 (4B). **IR** (ATR): 2922, 2595, 1734, 1650, 1598, 1543, 1469, 1438, 1196 cm^{-1}. **MS** (ESI) *m*/*z* (relative intensity): 674 (90) $[M]^+$. **HR-MS** (ESI): *m*/*z* calcd. for $C_{35}H_{41}B_{10}N_4O_3$ $[M+H]^+$: 675.4104, found: 675.4111.

Intermolecular Competition Experiment

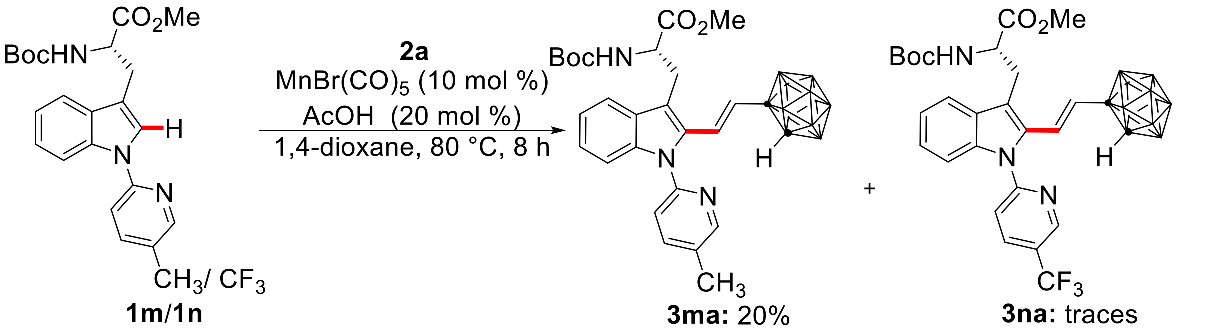

An oven dried Schlenk tube was charged with methyl N_α-(*tert*-butoxycarbonyl)-1-(5-methylpyridin-2-yl)-*L*-tryptophanate (**1m**) (0.2 mmol), methyl N_α-(*tert*-butoxycarbonyl)-1-(5-(trifluoromethyl)pyridin-2-yl)-*L*-tryptophanate (**1n**) (0.2 mmol), 1-ethynyl-*o*-carborane **2a** (0.1 mmol) and $MnBr(CO)_5$ (10 mol %), AcOH (1.2 µL, 20 mol %) and 1,4-dioxane (1 mL). After stirring at 80 °C for 8 h and cooling to ambient temperature, solvent was removed in vacuo. Purification by column chromatography on silica gel (*n*-hexane/EtOAc = 4/1) afforded **3ma** (11.6 mg, 20%) as a white solid and traces of **3na**.

H/D Exchange Experiment

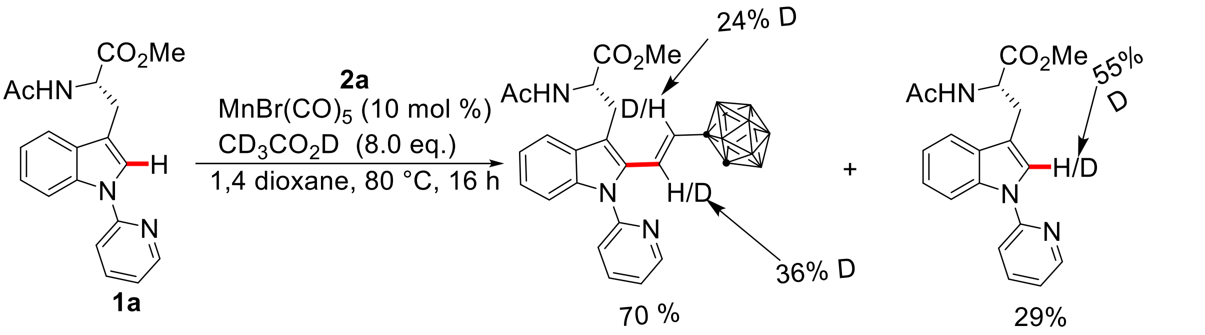

A solution of N^α-acetyl-1-(pyridin-2-yl)-*L*-tryptophanate (**1a**) (0.1 mmol), alkyne **2a** (0.1 mmol), $MnBr(CO)_5$ (10 mol %) and CD_3COD (8.0 equiv.) in 1,4-dioxane was stirred at 80 °C for 16 h. After cooling to room temperature, the solvent was removed in vacuo. Column

chromatography of the crude mixture (*n*-hexane/EtOAc = 3/2) afforded a mixture of the deuterium labeled amino acid **1a** (9.8 mg, 29%) and **3aa** (35.4 mg, 70 %).

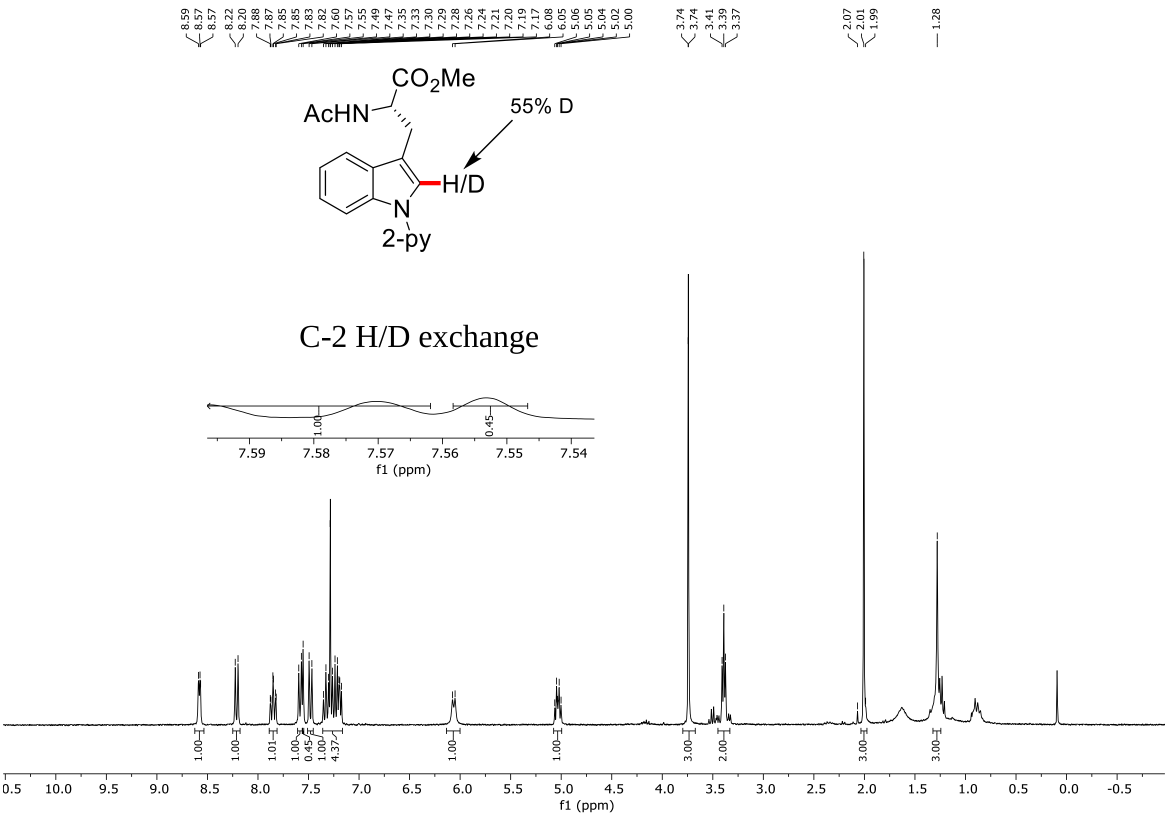

Studies on Potential Racemization

DL-tryptophan **3d** was subjected to manganese(I) catalyzed C–H alkenylation. HPLC **3da** revealed no racemization occurred during the reaction.

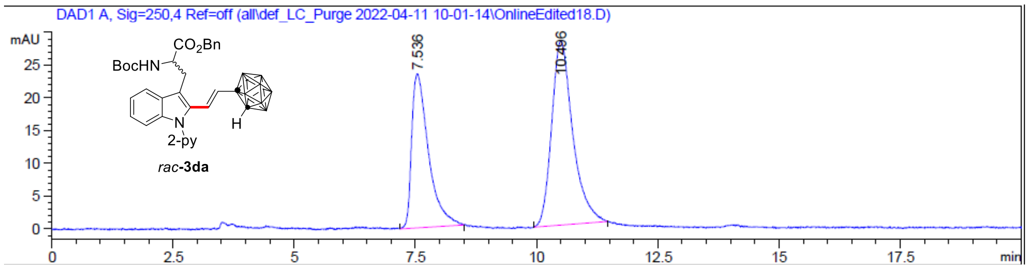

Signal 1: DAD1 A, Sig=250,4 Ref=off

Peak #	RetTime [min]	Type	Width [min]	Area [mAU*s]	Height [mAU]	Area %
1	7.536	BV R	0.2864	566.76721	23.50105	39.8986
2	10.496	BV R	0.3614	853.75244	28.18307	60.1014
Totals :				1420.51965	51.68411	

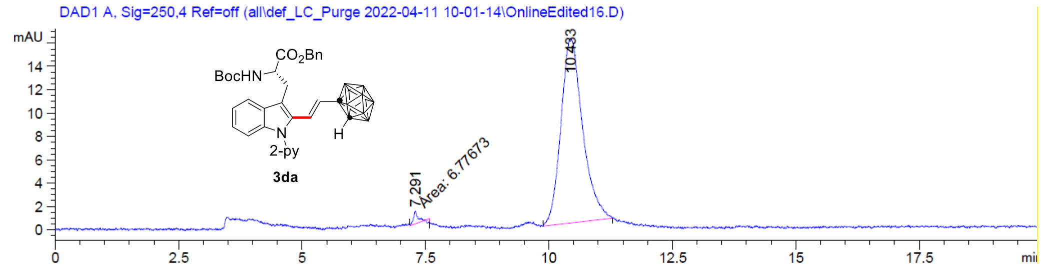

Signal 1: DAD1 A, Sig=250,4 Ref=off

Peak #	RetTime [min]	Type	Width [min]	Area [mAU*s]	Height [mAU]	Area %
1	7.291	MM	0.1044	6.77673	1.08218	1.3811
2	10.433	BB	0.3594	483.91034	15.84080	98.6189
Totals :				490.68706	16.92298	

Figure S1. HPLC-Chromatogram of **3da**. These were recorded on an Agilent 1290 Infinity using the column CHIRALPAK® ID and *n*-hexane/*i*PrOH (90:10, 1 mL/min, detection at 250 nm)

Plausible Mechanism

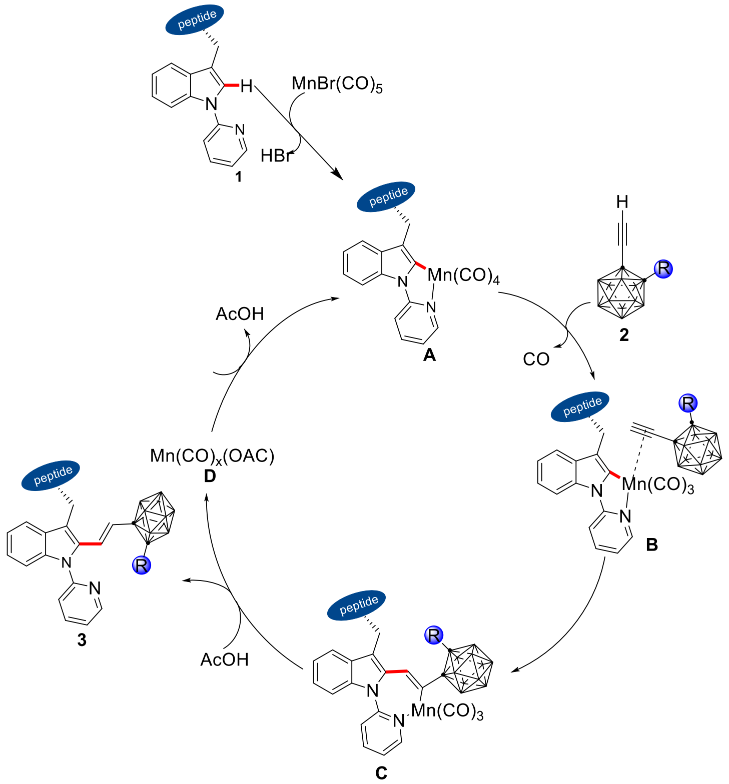

Scheme S1. Plausible Reaction

References

1. X. Wu, J. Guo, Y. Quan, W. Jia, D. Jia, Y. Chen and Z. Xie, *J. Mater. Chem. C*, 2018, **6**, 4140–4149.
2. M. M. Lorion, N. Kaplaneris, J. Son, R. Kuniyil and L. Ackermann, *Angew. Chem. Int. Ed.*, 2019, **58**, 1684-1688.

^{1}H, ^{13}C, ^{11}B, ^{19}F NMR Spectra

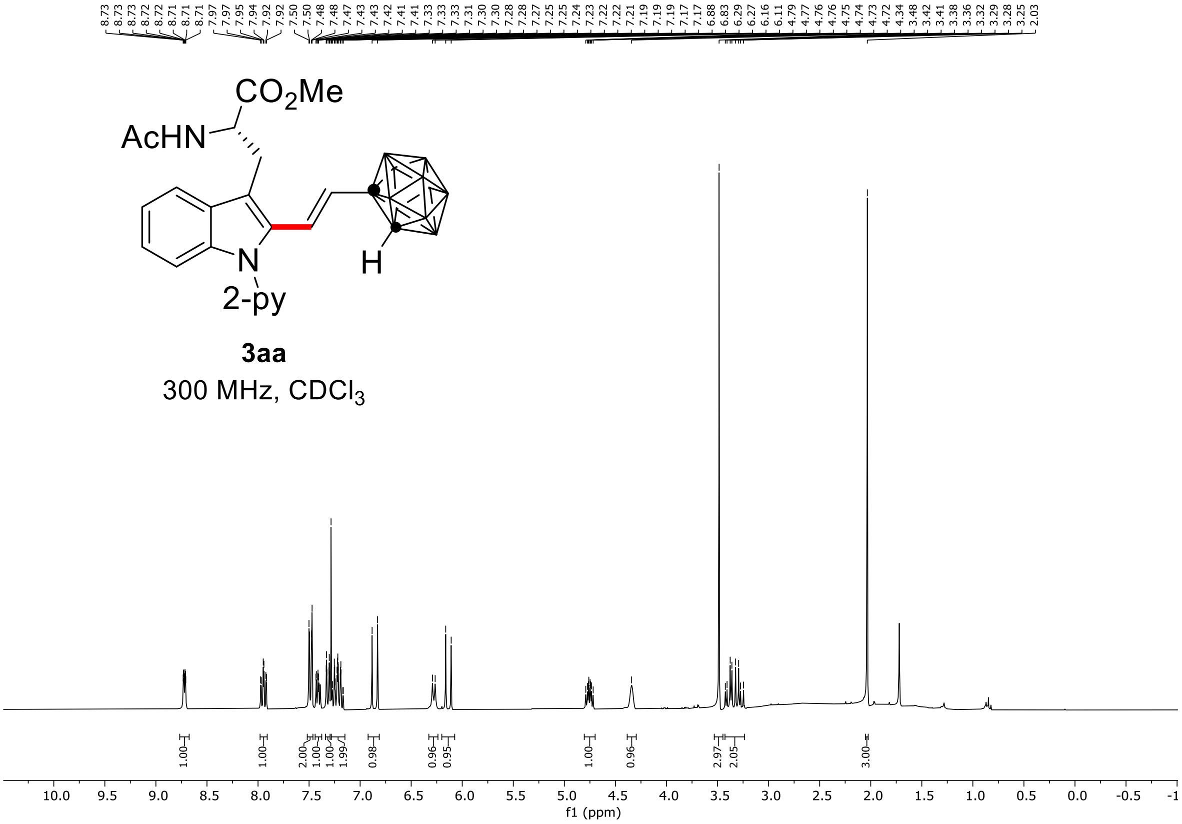

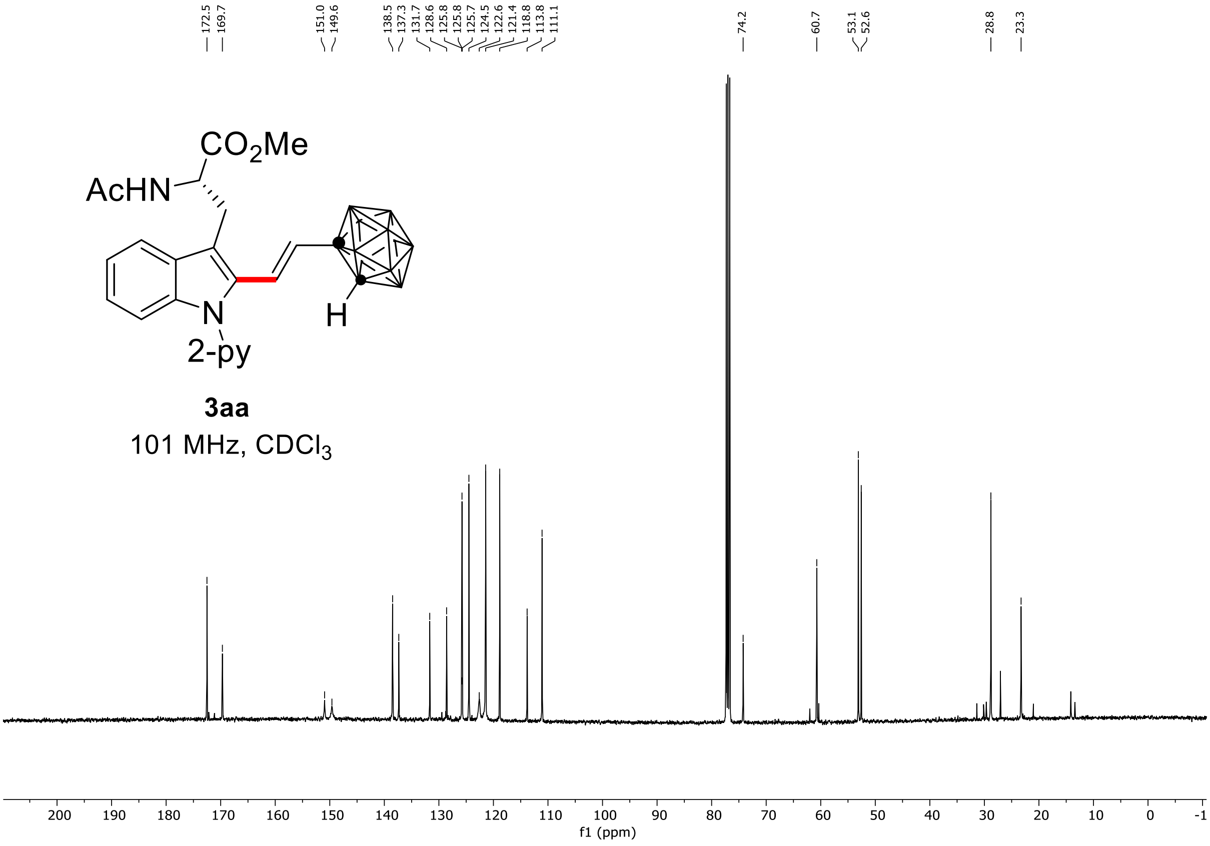

3aa
101 MHz, $CDCl_3$

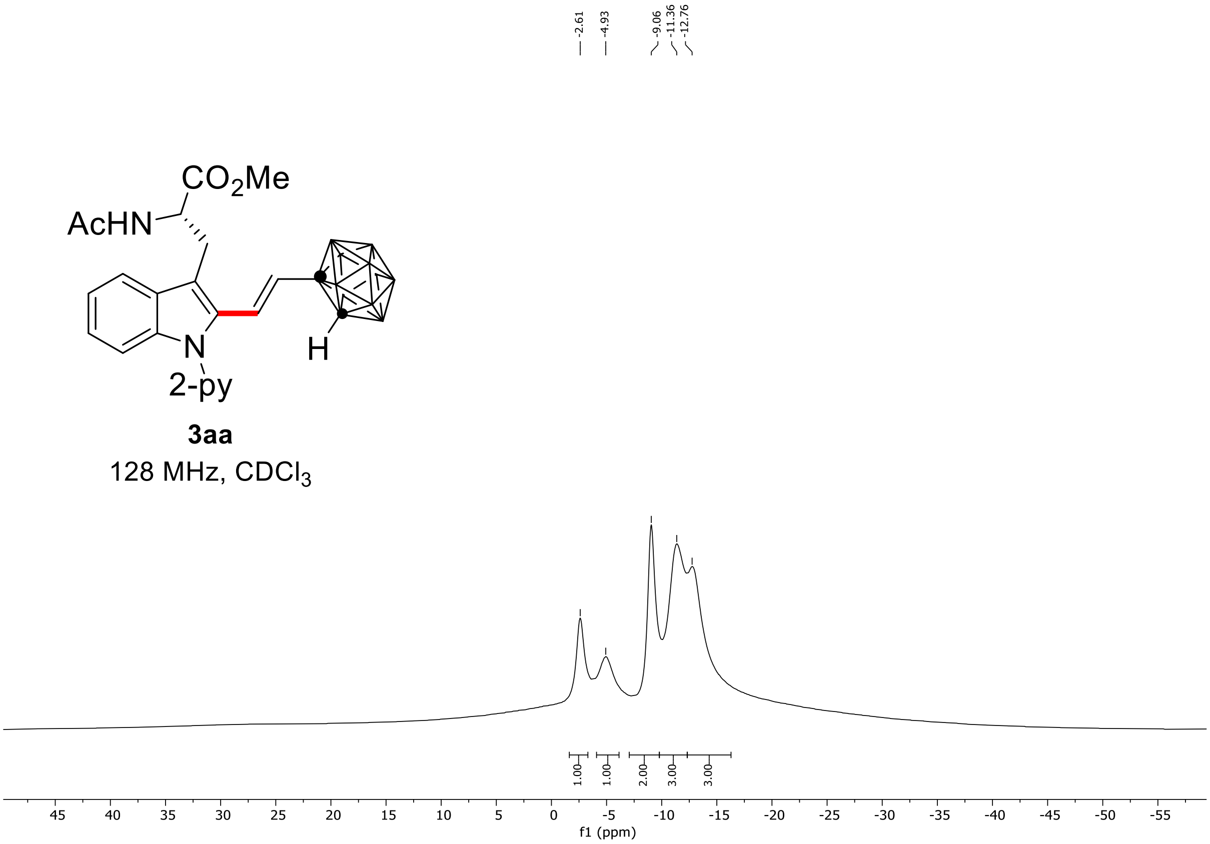

3aa
128 MHz, $CDCl_3$

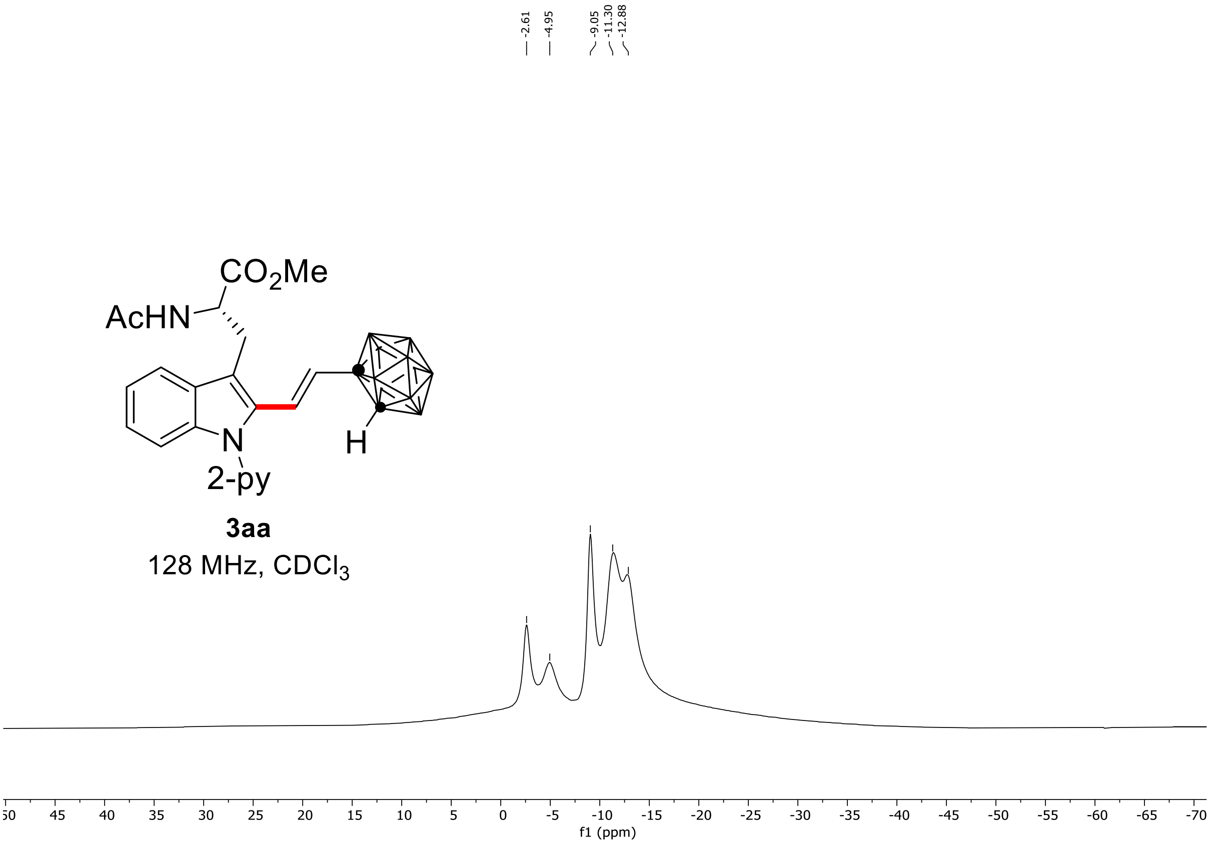
-2.61
-4.95
-9.05
-11.30
-12.88
CO2Me
AcHN
N
2-py
H
3aa
128 MHz, CDCl3
f1 (ppm)

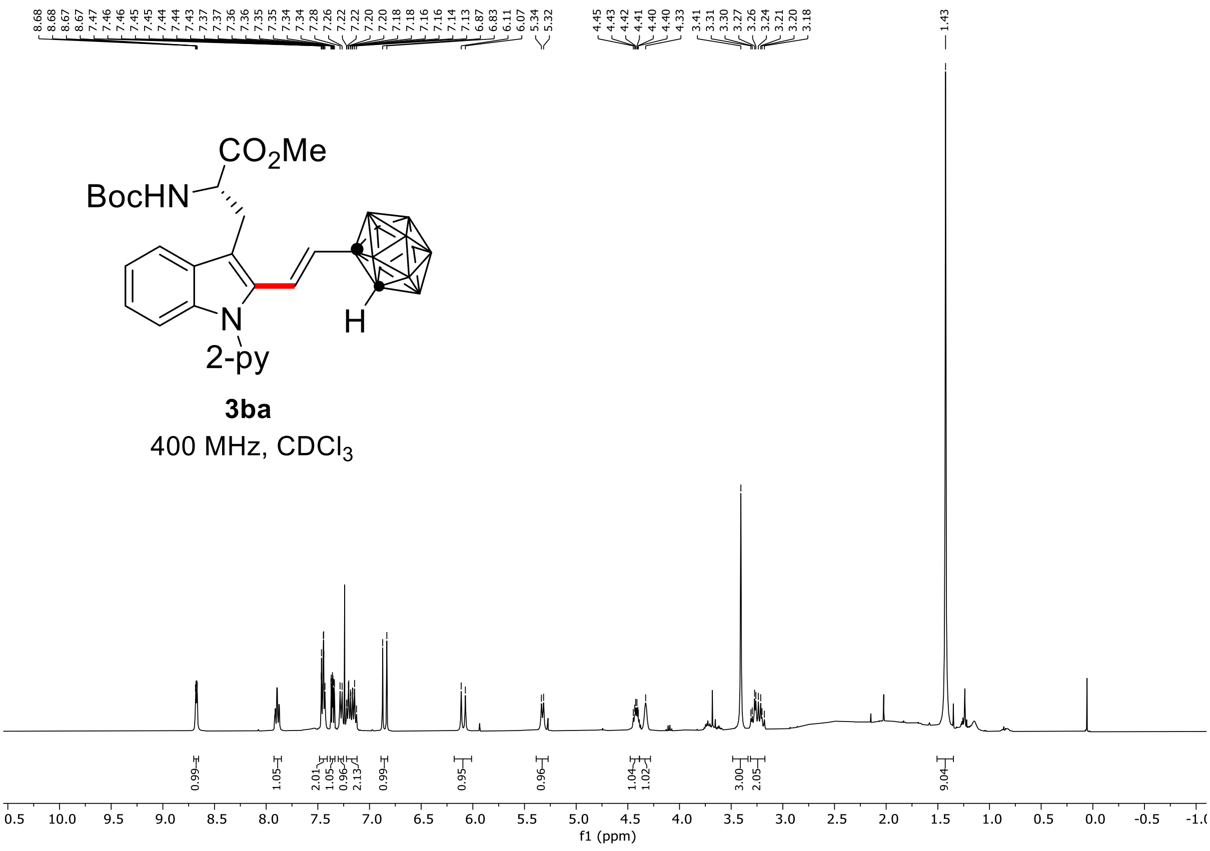
8.68 8.68 8.67 7.47 7.46 7.46 7.45 7.45 7.44 7.44 7.43 7.37 7.37 7.36 7.36 7.35 7.35 7.34 7.34 7.28 7.26 7.22 7.22 7.20 7.20 7.18 7.18 7.16 7.16 7.14 7.13 6.87 6.83 6.11 6.07
5.34 5.32
4.45 4.43 4.42 4.41 4.40 4.40 4.33 3.41 3.31 3.30 3.27 3.26 3.24 3.21 3.20 3.18
1.43
CO2Me
BocHN
N
2-py
H
3ba
400 MHz, CDCl3
0.99
1.05
2.01
1.05
0.96
2.13
0.99
0.95
0.96
1.04
1.02
3.00
2.05
9.04
f1 (ppm)

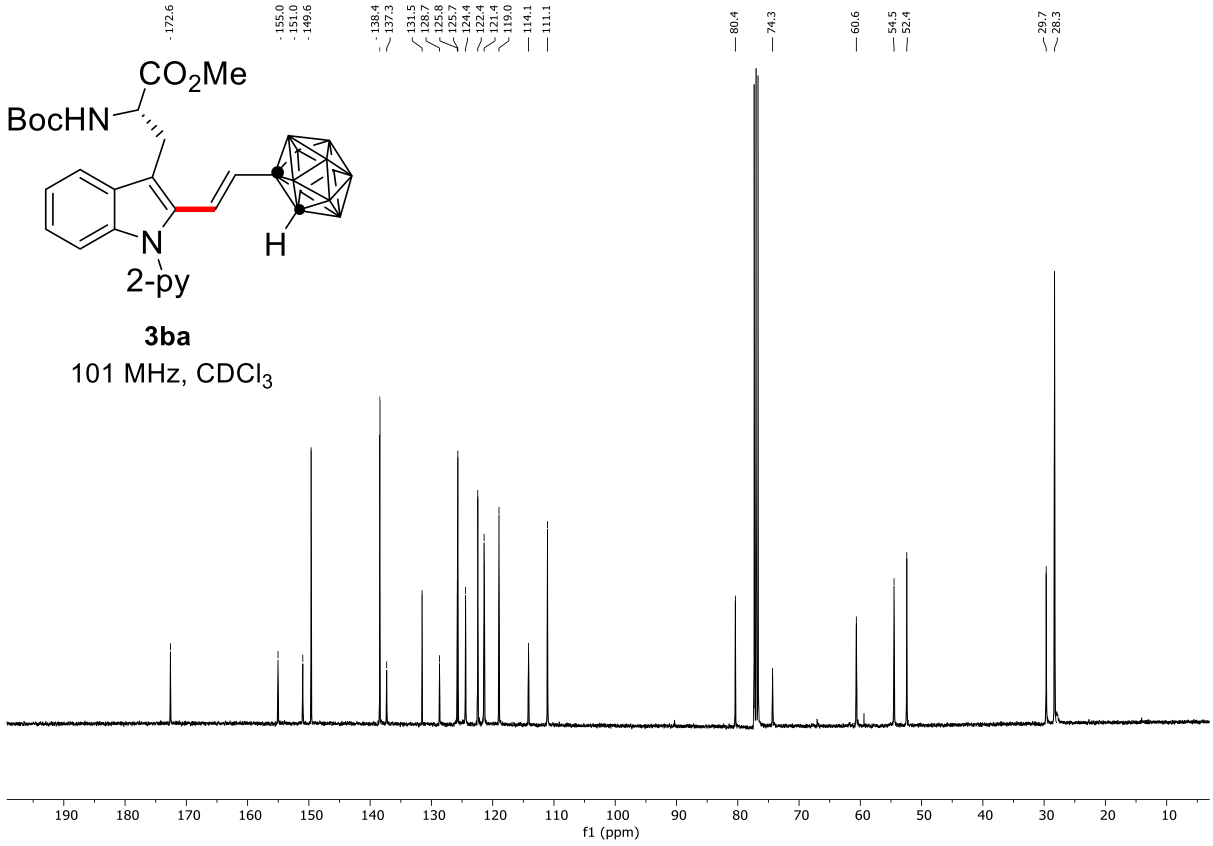
172.6
155.0
151.0
149.6
138.4
137.3
131.5
128.7
125.8
125.7
124.4
122.4
121.4
119.0
114.1
111.1
80.4
74.3
60.6
54.5
52.4
29.7
28.3
CO2Me
BocHN
N
2-py
H
3ba
101 MHz, CDCl3
f1 (ppm)

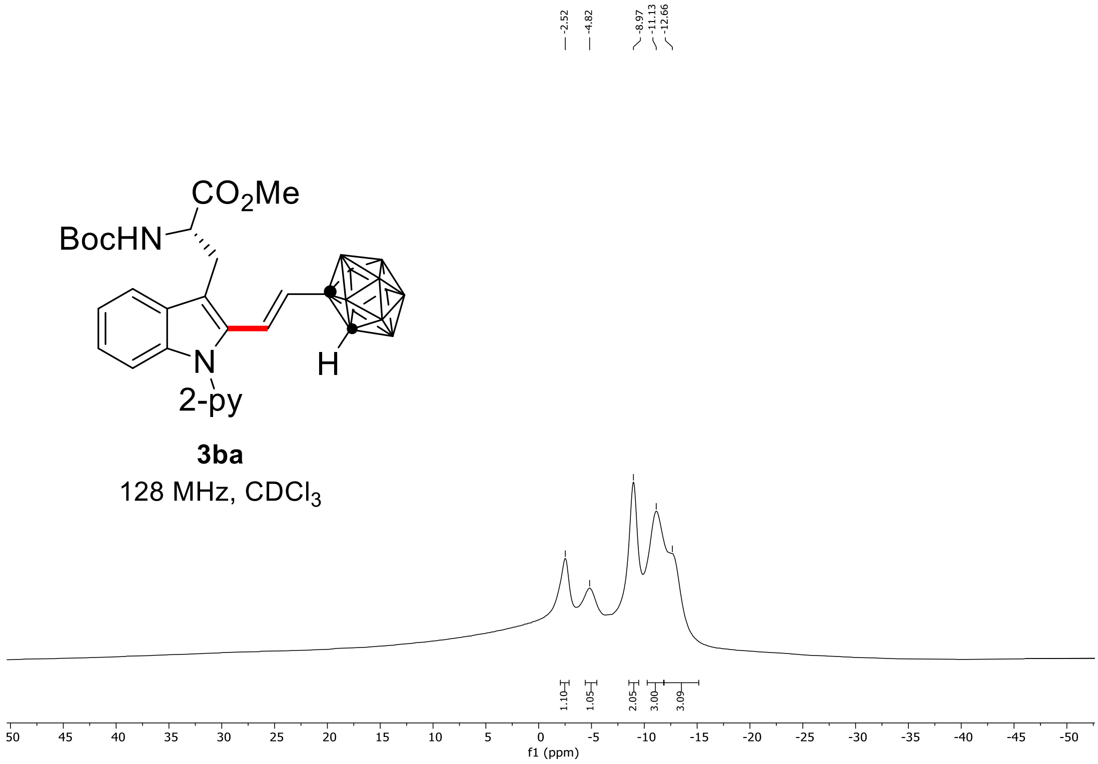
-2.52
-4.82
-8.97
-11.13
-12.66
CO2Me
BocHN
N
2-py
H
3ba
128 MHz, CDCl3
1.10
1.05
2.05
3.00
3.09
f1 (ppm)

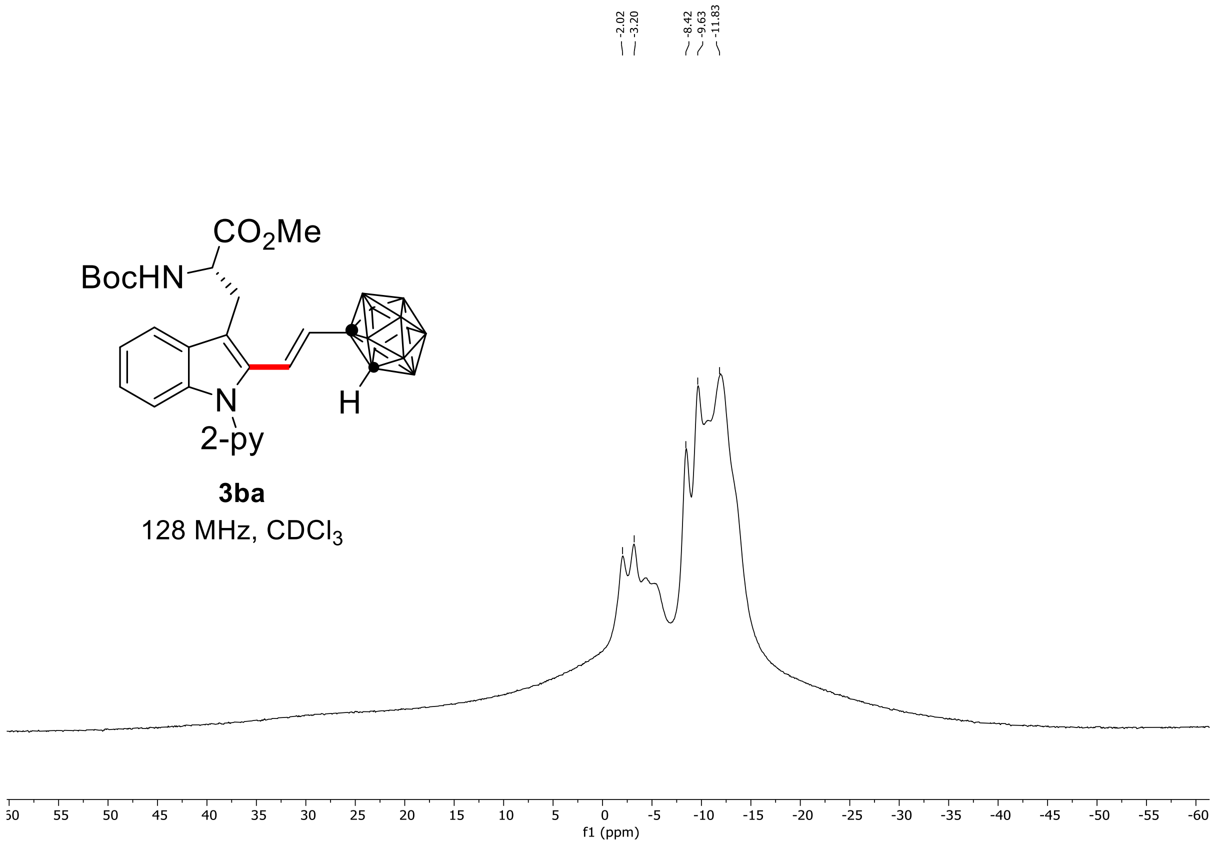
CO2Me
BocHN
N
2-py
H
3ba
128 MHz, CDCl3
f1 (ppm)

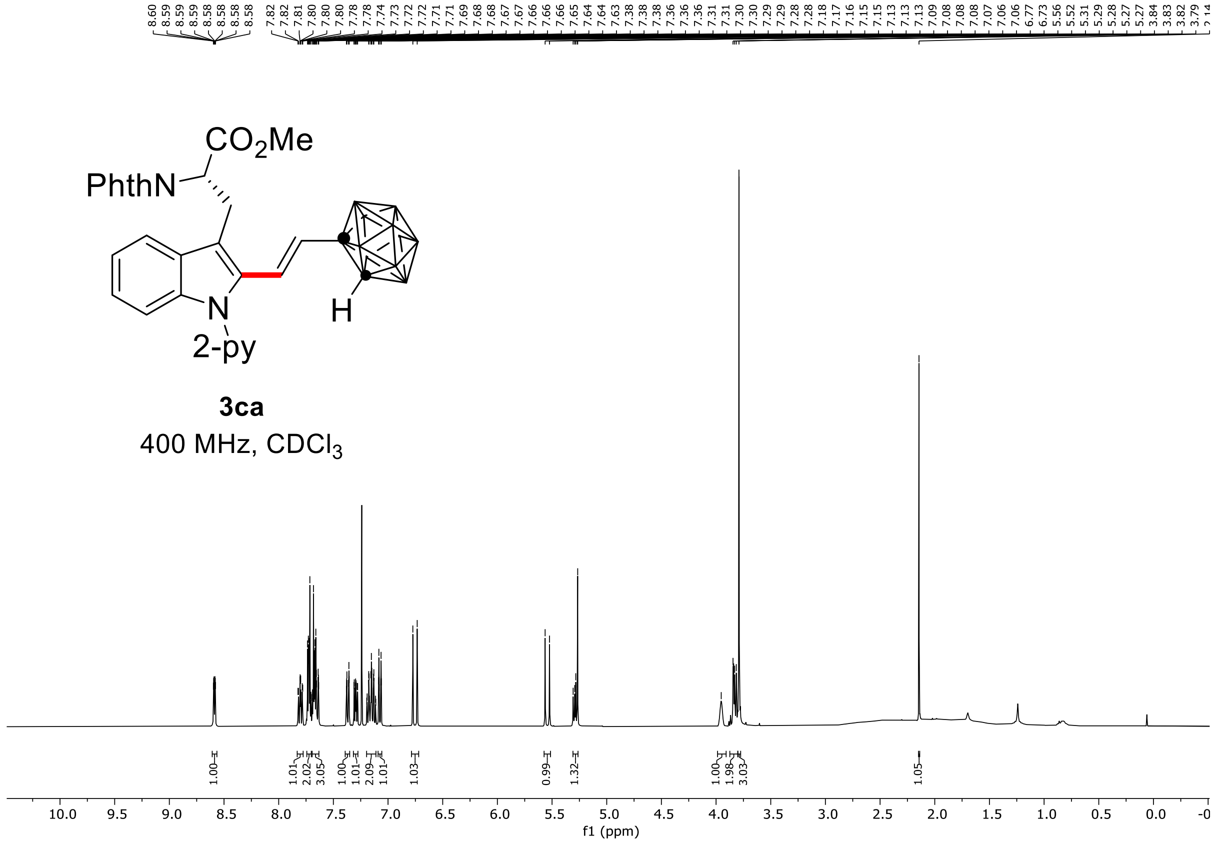
CO2Me
PhthN
N
2-py
H
3ca
400 MHz, CDCl3
f1 (ppm)

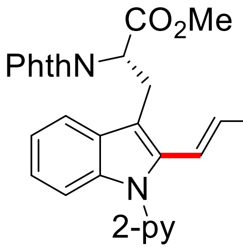

3ca

126 MHz, $CDCl_3$

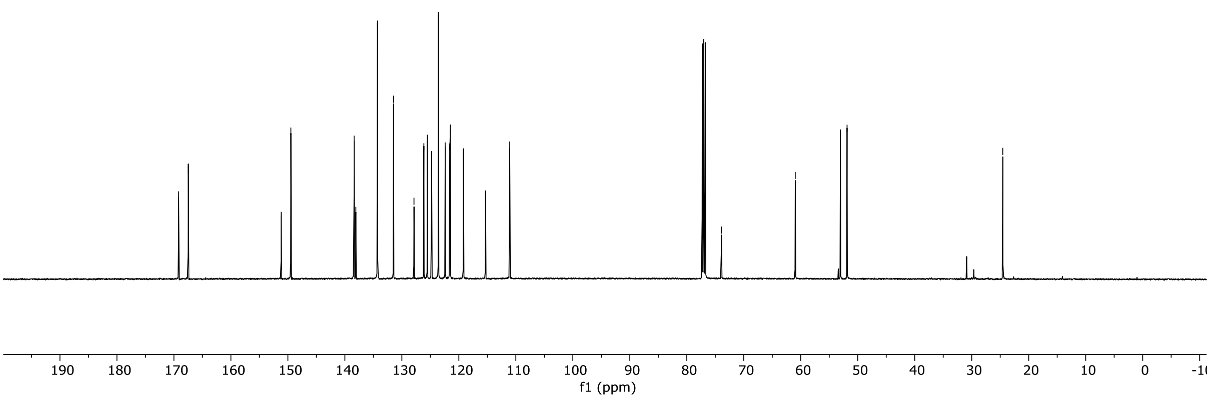

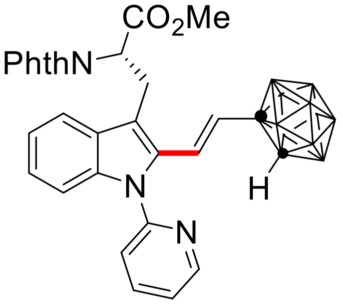

3ca

128 MHz, $CDCl_3$

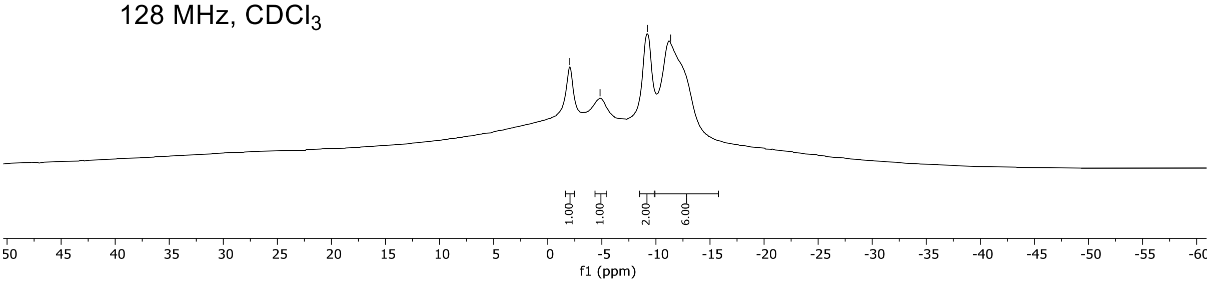

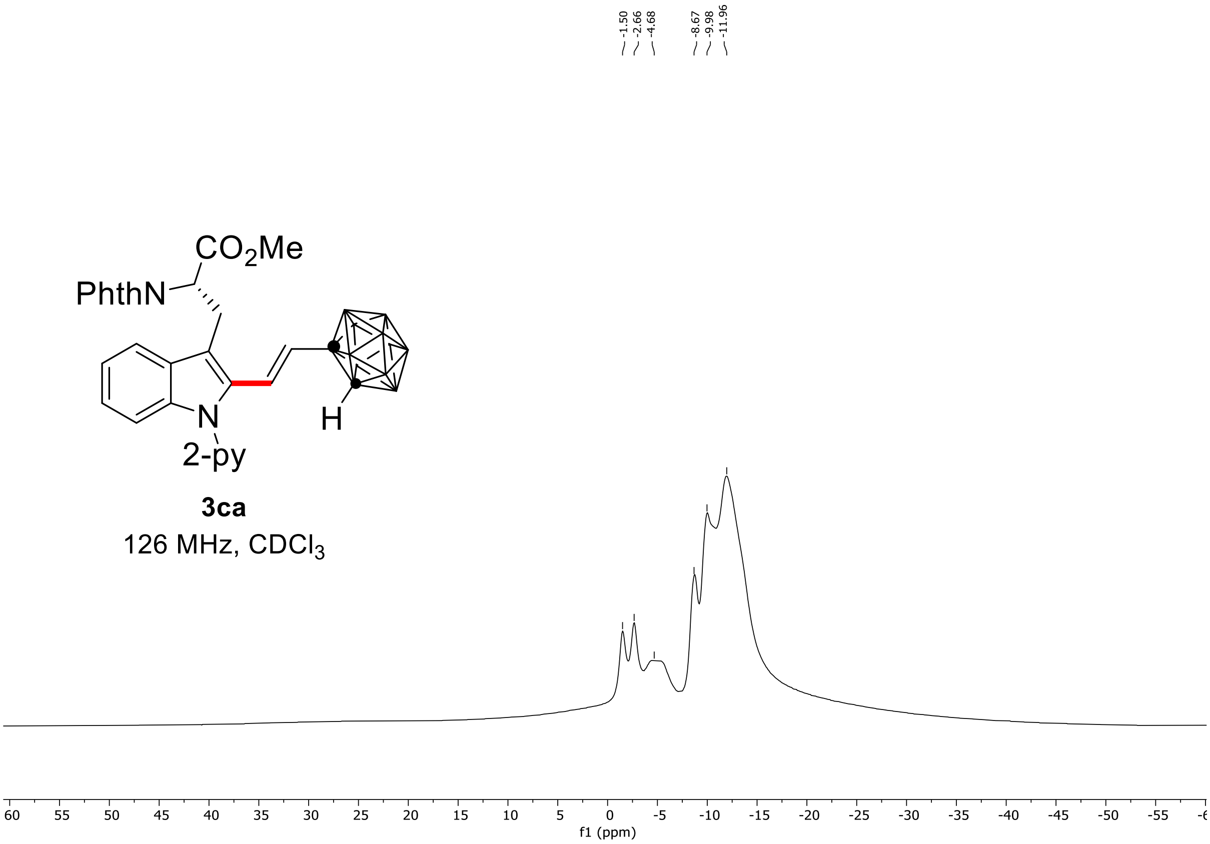

-1.50
-2.66
-4.68
-8.67
-9.98
-11.96
CO_2Me
PhthN
H
N
2-py
3ca
126 MHz, $CDCl_3$
60 55 50 45 40 35 30 25 20 15 10 5 0 -5 -10 -15 -20 -25 -30 -35 -40 -45 -50 -55
f1 (ppm)

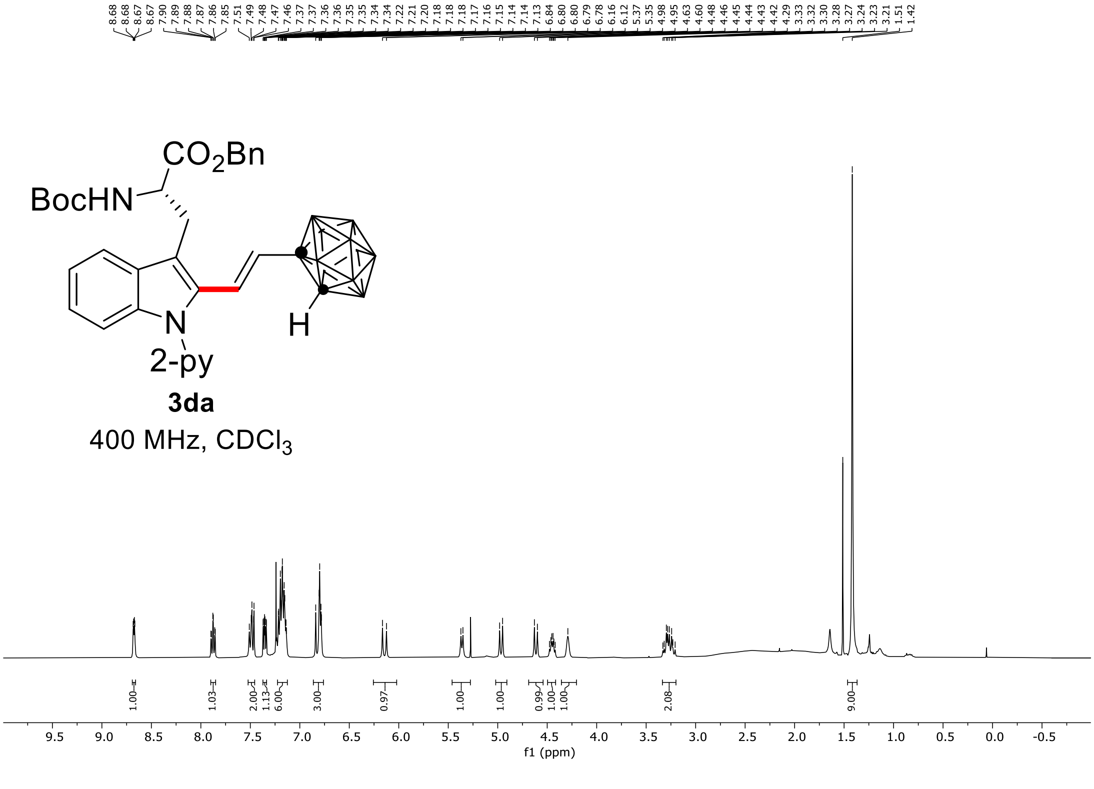

8.68 8.68 8.67 8.67 7.90 7.89 7.88 7.87 7.86 7.85 7.51 7.49 7.48 7.47 7.46 7.37 7.37 7.36 7.36 7.35 7.35 7.34 7.34 7.22 7.21 7.20 7.18 7.18 7.17 7.16 7.15 7.14 7.14 7.13 6.84 6.80 6.80 6.79 6.78 6.16 6.12 5.37 5.35 4.98 4.95 4.63 4.60 4.48 4.46 4.45 4.44 4.43 4.42 4.29 3.33 3.32 3.30 3.28 3.27 3.24 3.23 3.21 1.51 1.42
CO_2Bn
BocHN
H
N
2-py
3da
400 MHz, $CDCl_3$
1.00 1.03 2.00 1.13 6.00 3.00 0.97 1.00 1.00 0.99 1.00 1.00 2.08 9.00
9.5 9.0 8.5 8.0 7.5 7.0 6.5 6.0 5.5 5.0 4.5 4.0 3.5 3.0 2.5 2.0 1.5 1.0 0.5 0.0 -0.5
f1 (ppm)

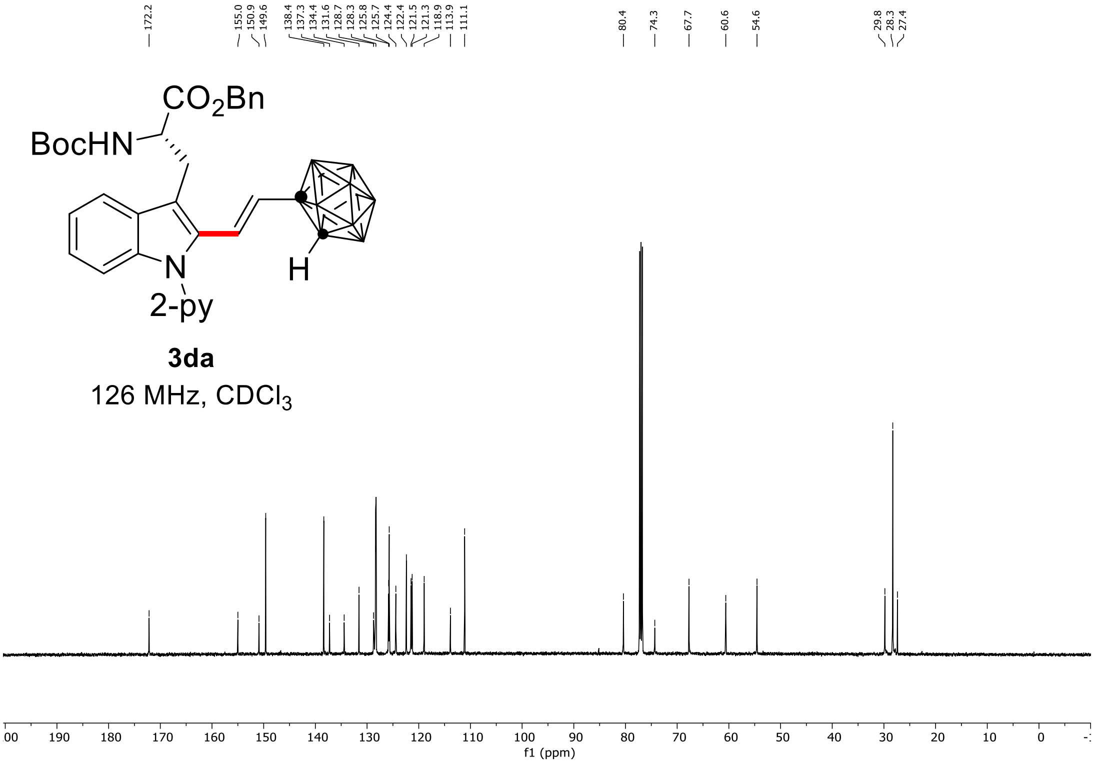

3da

126 MHz, $CDCl_3$

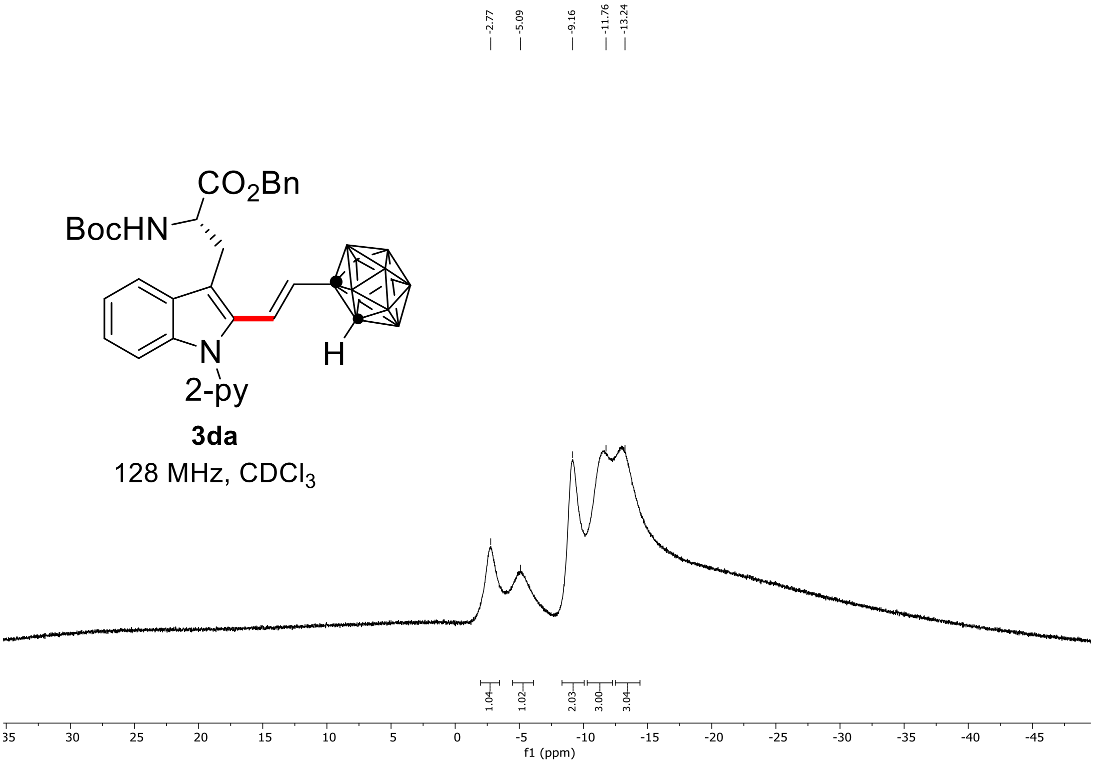

3da

128 MHz, $CDCl_3$

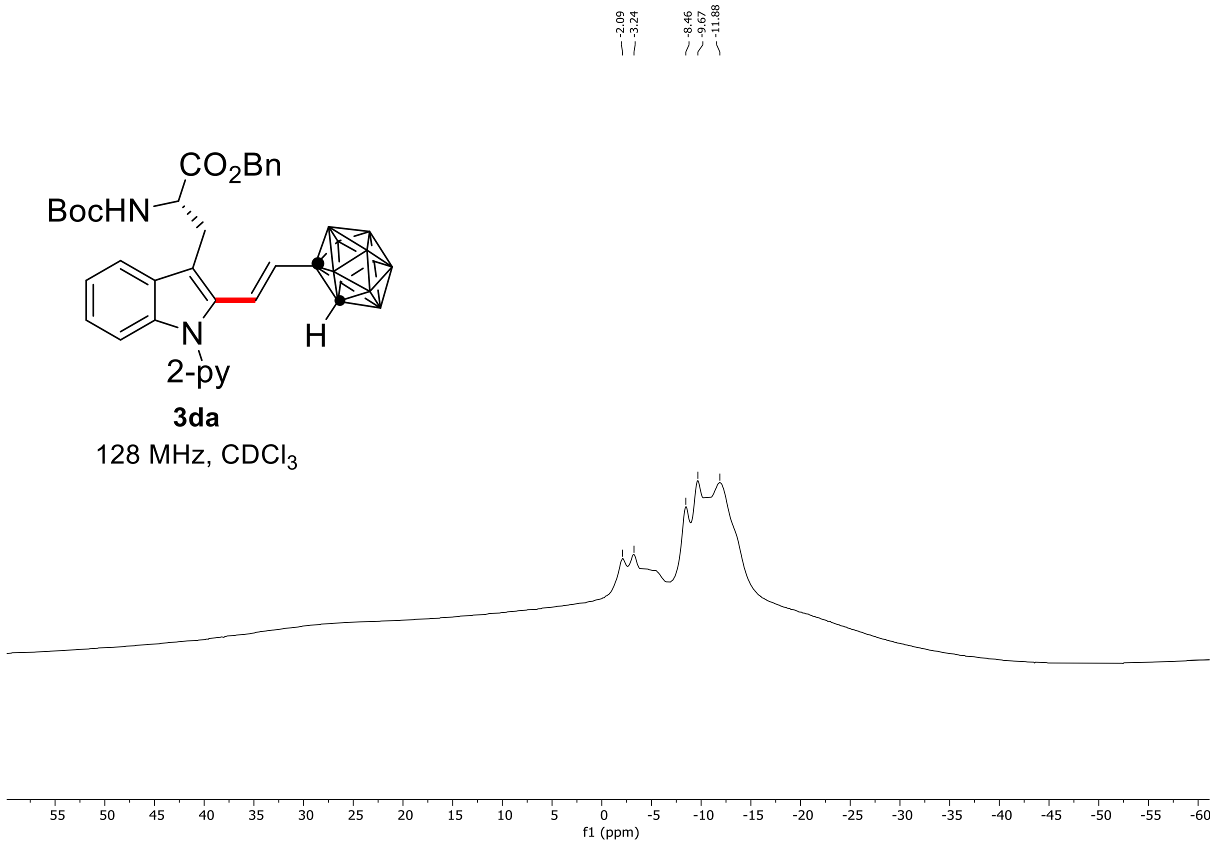

-2.09
-3.24
-8.46
-9.67
-11.88
CO2Bn
BocHN
N
2-py
H
3da
128 MHz, CDCl3
f1 (ppm)

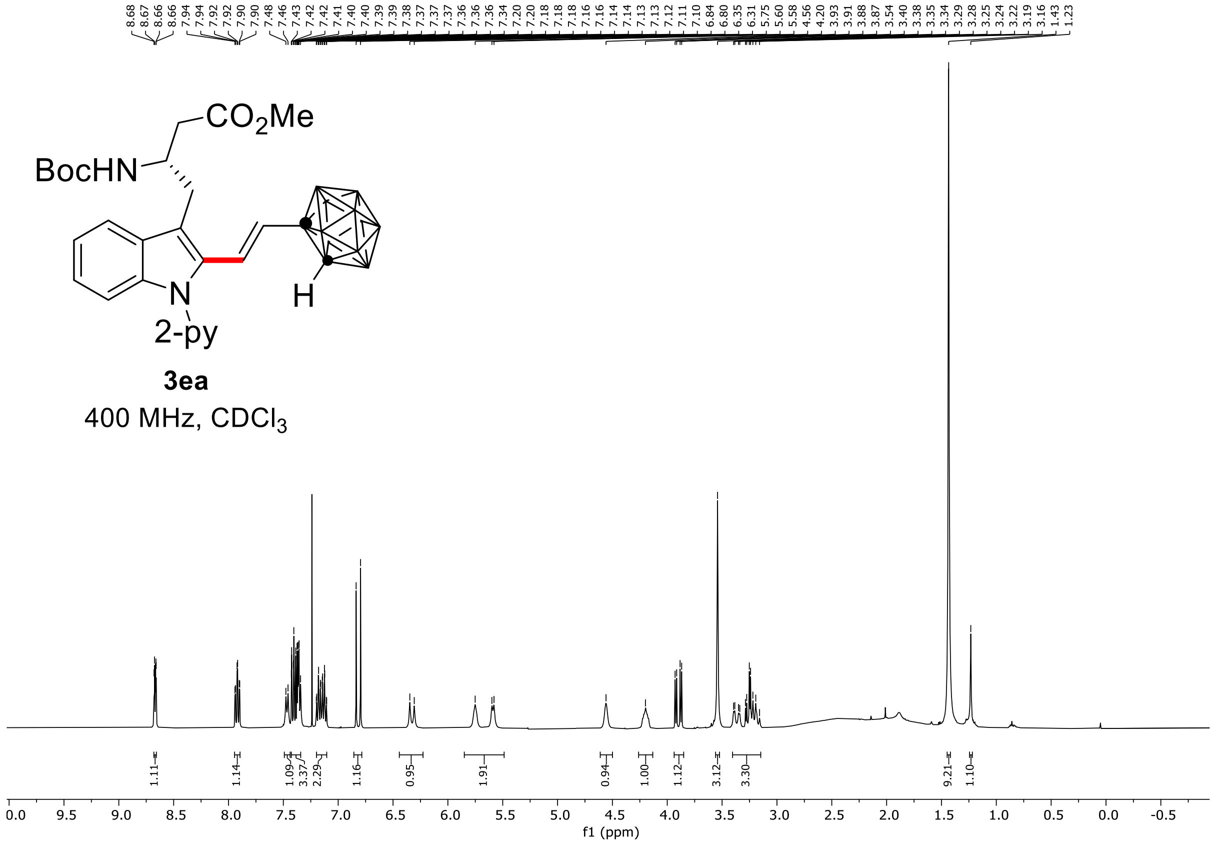

8.68 8.67 8.66 8.66 7.94 7.94 7.92 7.92 7.90 7.90 7.48 7.46 7.43 7.42 7.42 7.41 7.40 7.39 7.39 7.38 7.37 7.37 7.37 7.36 7.36 7.36 7.34 7.20 7.20 7.18 7.18 7.18 7.16 7.16 7.14 7.14 7.13 7.13 7.12 7.11 7.10 6.84 6.80 6.35 6.31 5.75 5.60 5.58 4.56 4.20 3.93 3.91 3.88 3.87 3.54 3.40 3.38 3.35 3.34 3.29 3.28 3.25 3.24 3.22 3.19 3.16 1.43 1.23
CO2Me
BocHN
N
2-py
H
3ea
400 MHz, CDCl3
1.11 1.14 1.09 3.37 2.29 1.16 0.95 1.91 0.94 1.00 1.12 3.12 3.30 9.21 1.10
f1 (ppm)

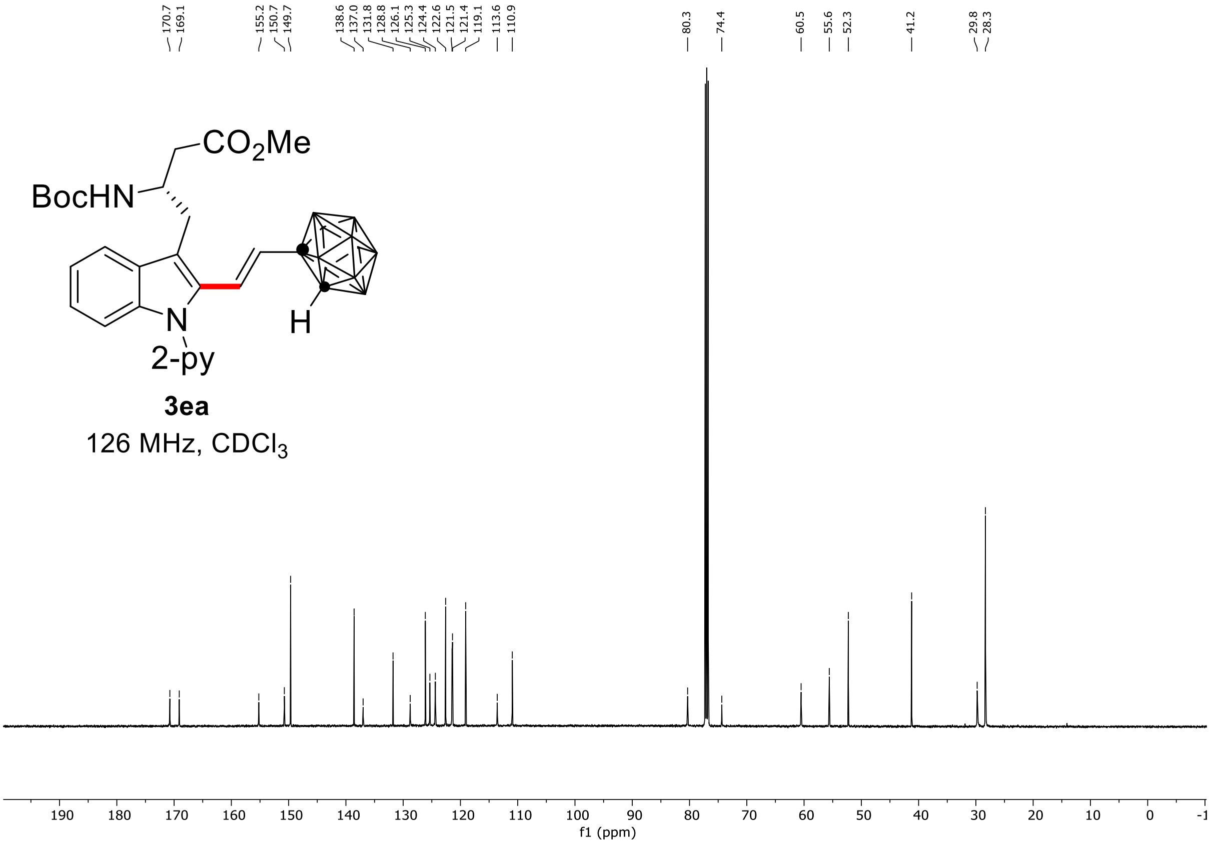
170.7
169.1
155.2
150.7
149.7
138.6
137.0
131.8
128.8
126.1
125.3
124.4
122.6
121.5
121.4
119.1
113.6
110.9
80.3
74.4
60.5
55.6
52.3
41.2
29.8
28.3
CO2Me
BocHN
N
2-py
H
3ea
126 MHz, CDCl3
f1 (ppm)

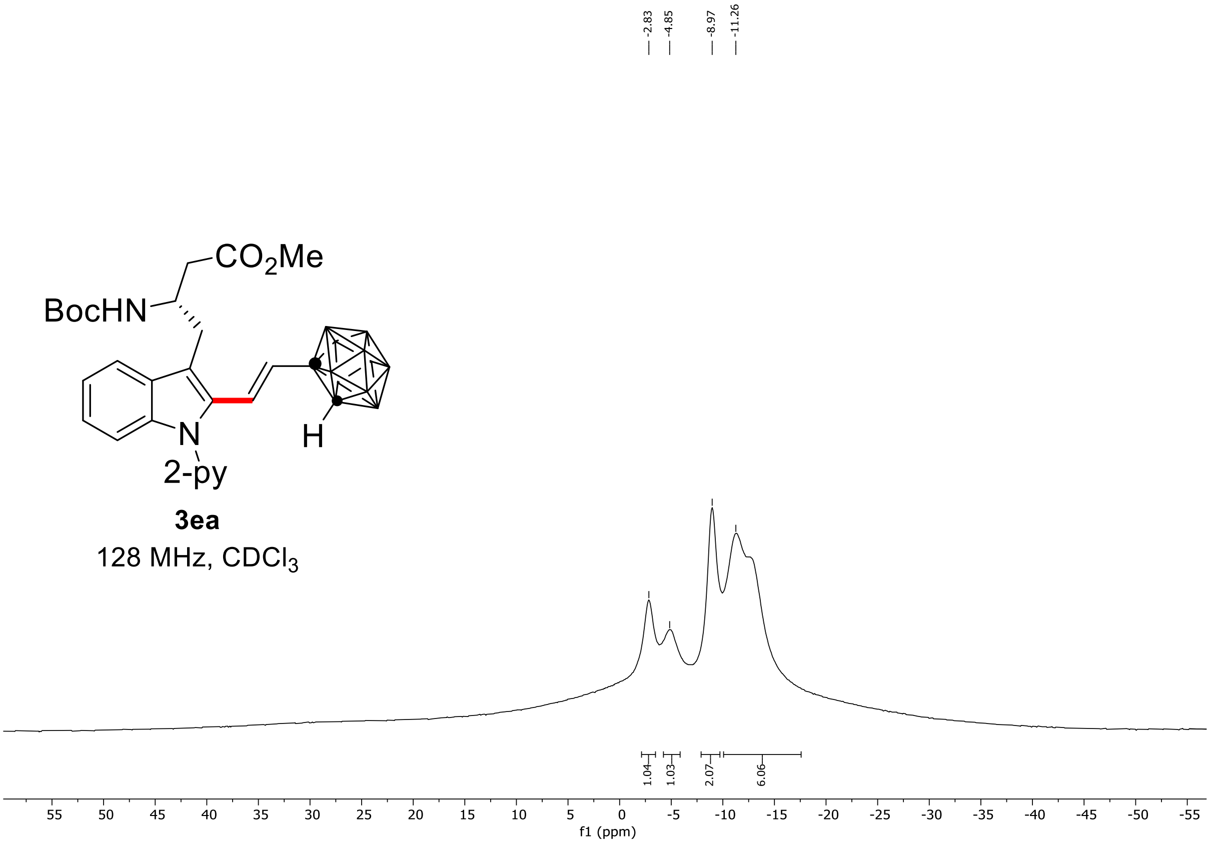
-2.83
-4.85
-8.97
-11.26
CO2Me
BocHN
N
2-py
H
3ea
128 MHz, CDCl3
1.04
1.03
2.07
6.06
f1 (ppm)

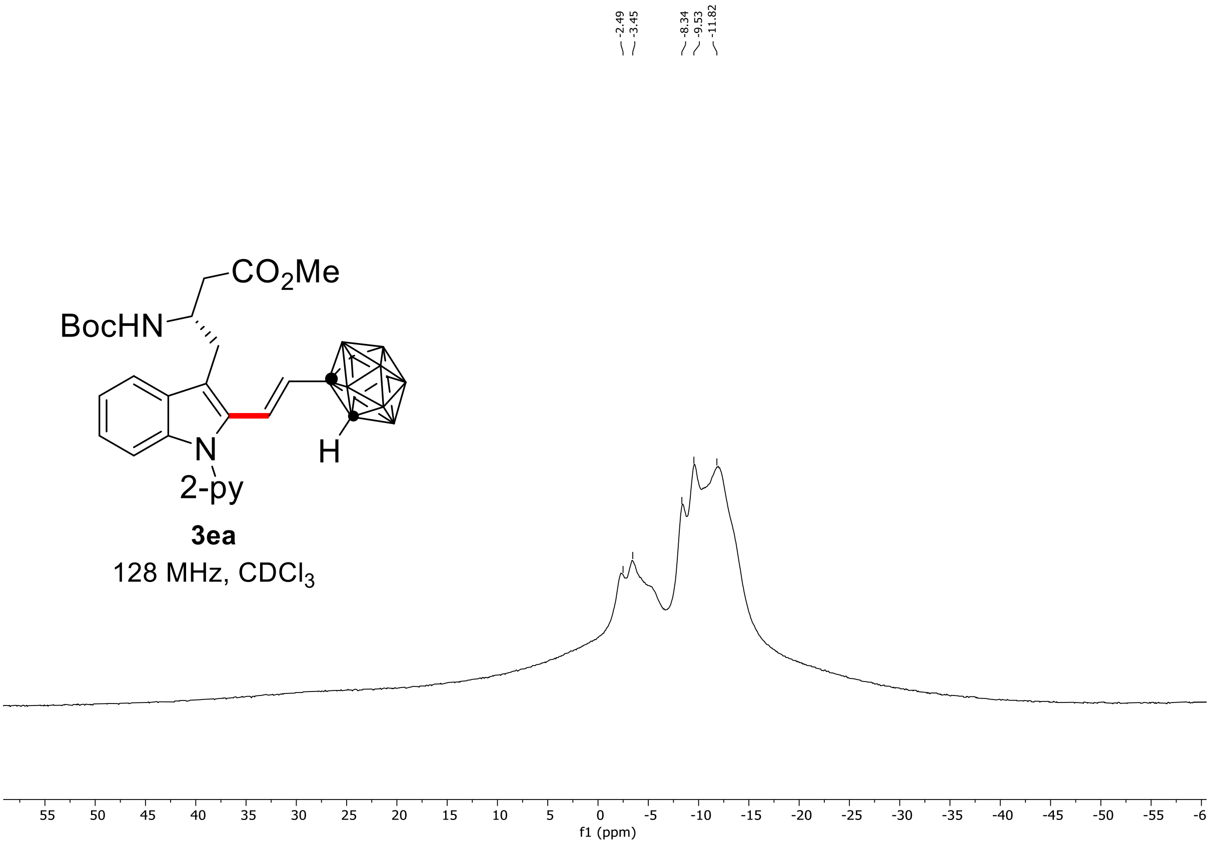

CO2Me
BocHN
N
2-py
H
3ea
128 MHz, CDCl3
-2.49
-3.45
-8.34
-9.53
-11.82
f1 (ppm)

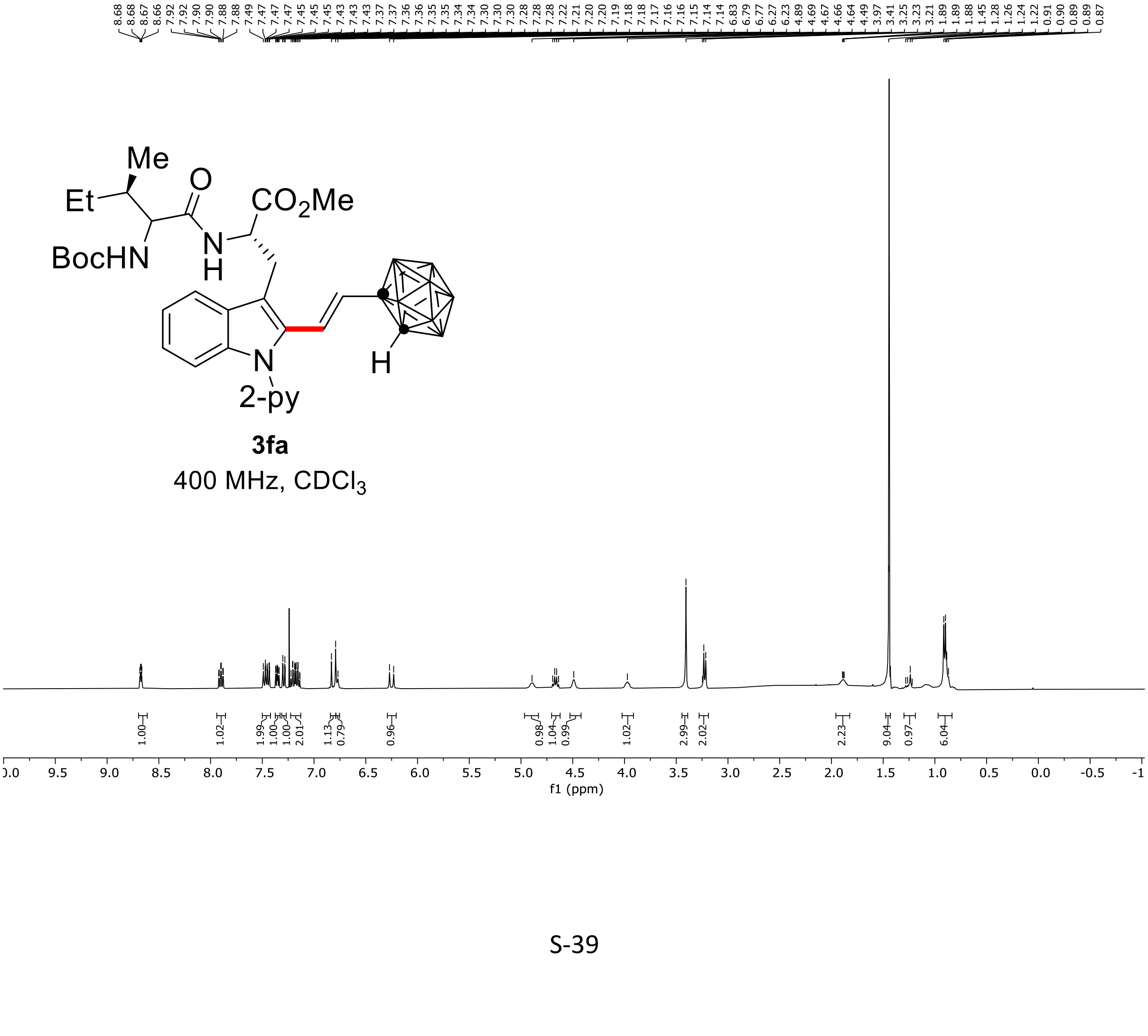

Me
O
Et
CO2Me
BocHN
N
H
N
2-py
H
3fa
400 MHz, CDCl3
f1 (ppm)

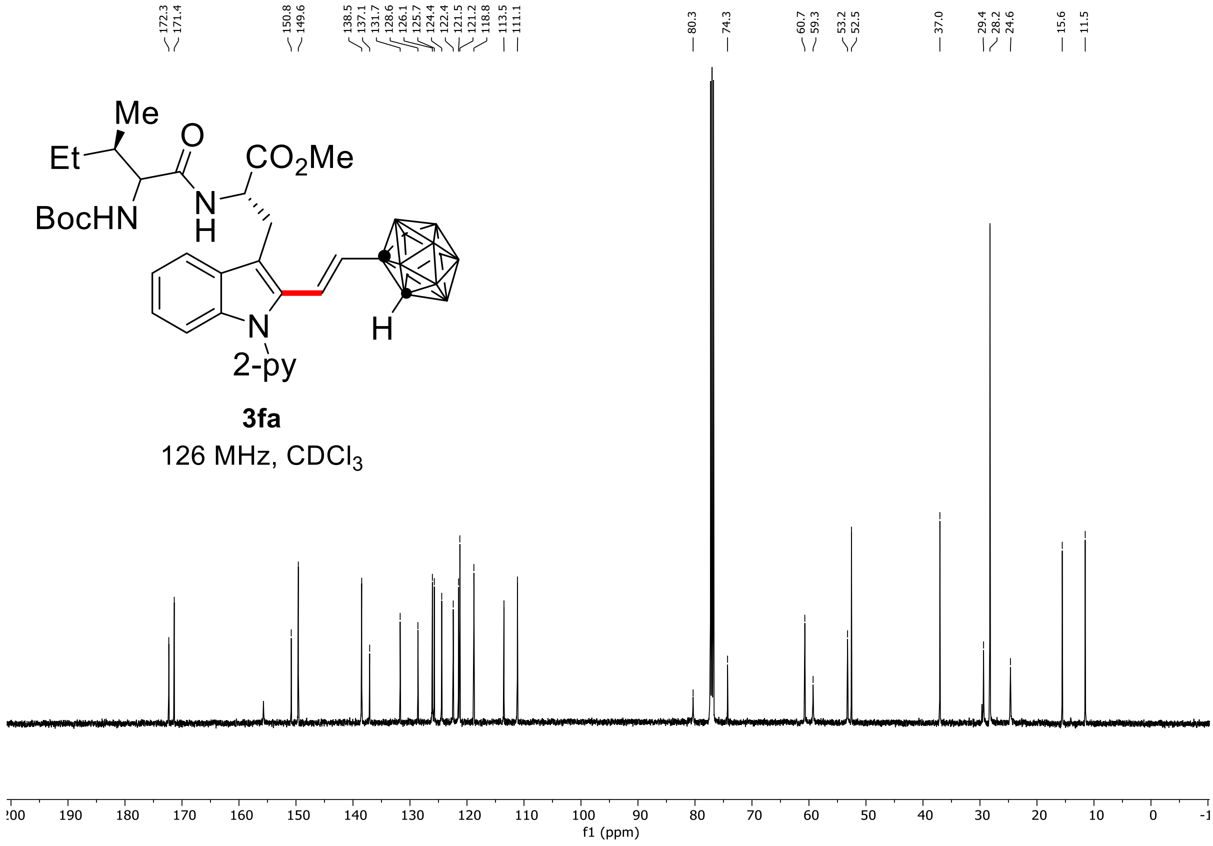
172.3
171.4
150.8
149.6
138.5
137.1
131.7
128.6
126.1
125.7
124.4
122.4
121.5
121.2
118.8
113.5
111.1
80.3
74.3
60.7
59.3
53.2
52.5
37.0
29.4
28.2
24.6
15.6
11.5
Me
Et
O
CO_2Me
BocHN
N
H
N
2-py
H
3fa
126 MHz, $CDCl_3$
f1 (ppm)

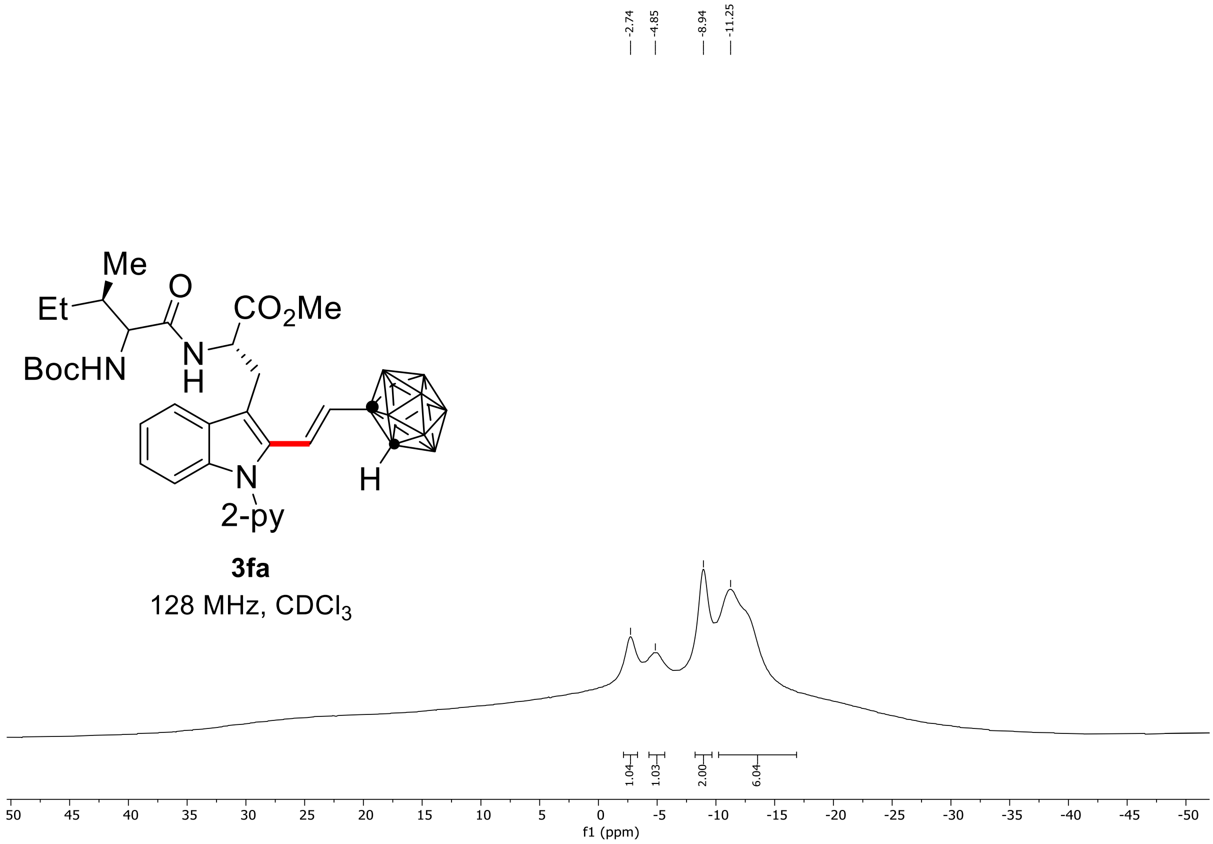
-2.74
-4.85
-8.94
-11.25
Me
Et
O
CO_2Me
BocHN
N
H
N
2-py
H
3fa
128 MHz, $CDCl_3$
1.04
1.03
2.00
6.04
f1 (ppm)

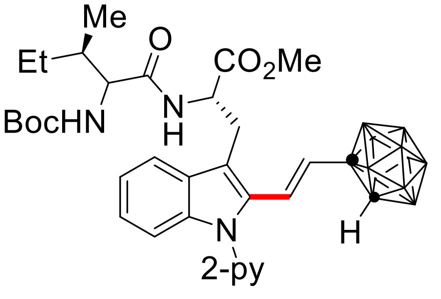

3fa

128 MHz, $CDCl_3$

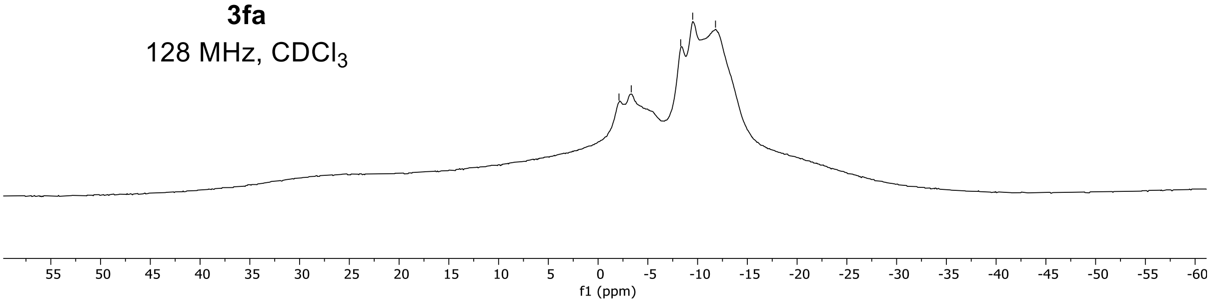

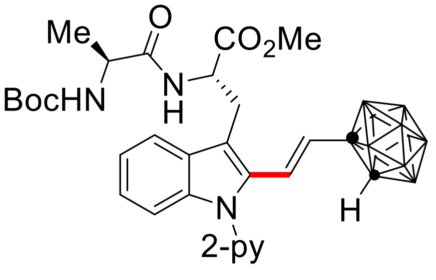

3ga

400 MHz, $CDCl_3$

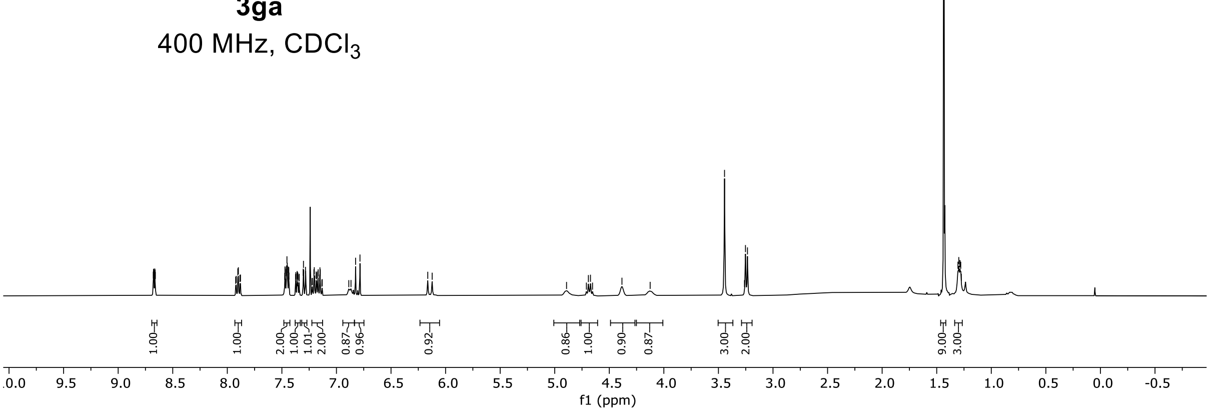

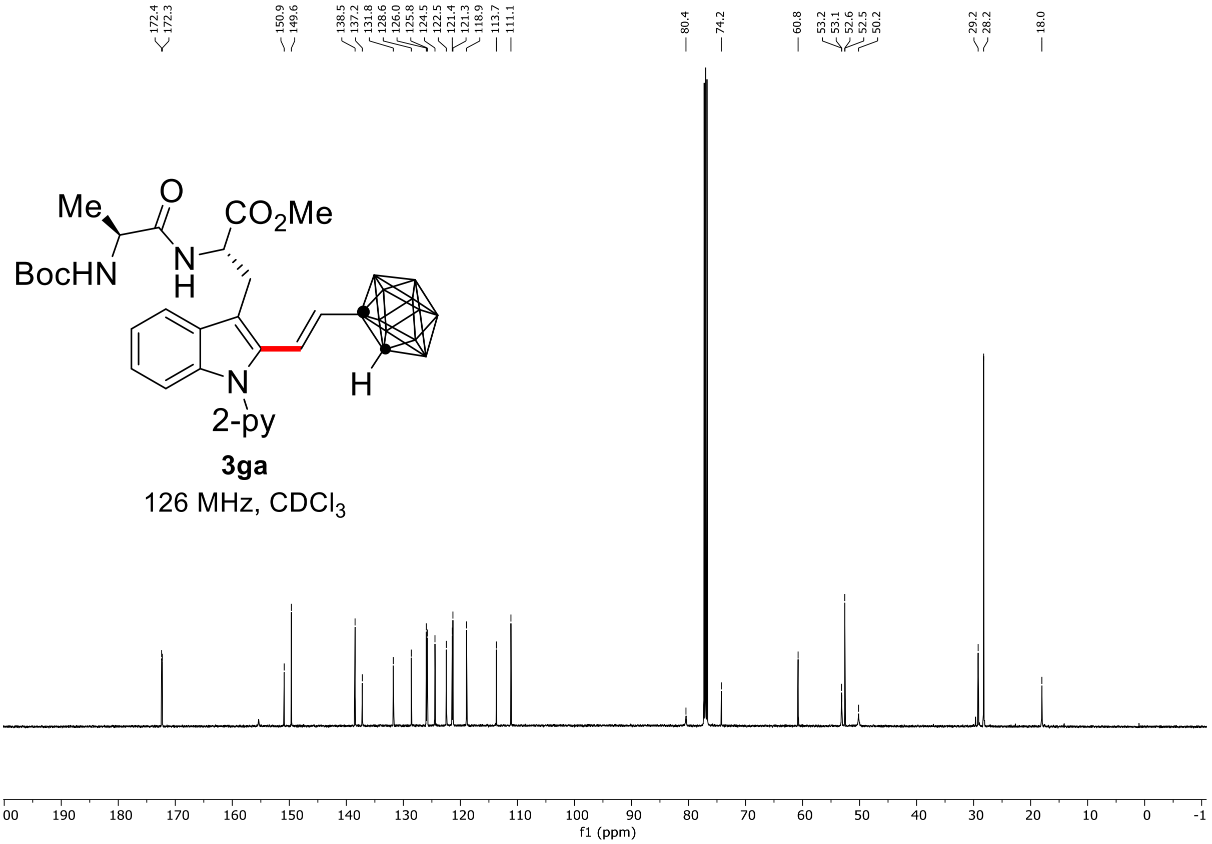
172.4
172.3
150.9
149.6
138.5
137.2
131.8
128.6
126.0
125.8
124.5
122.5
121.4
121.3
118.9
113.7
111.1
80.4
74.2
60.8
53.2
53.1
52.6
52.5
50.2
29.2
28.2
18.0
Me
O
CO2Me
BocHN
N
H
N
2-py
H
3ga
126 MHz, CDCl3
f1 (ppm)

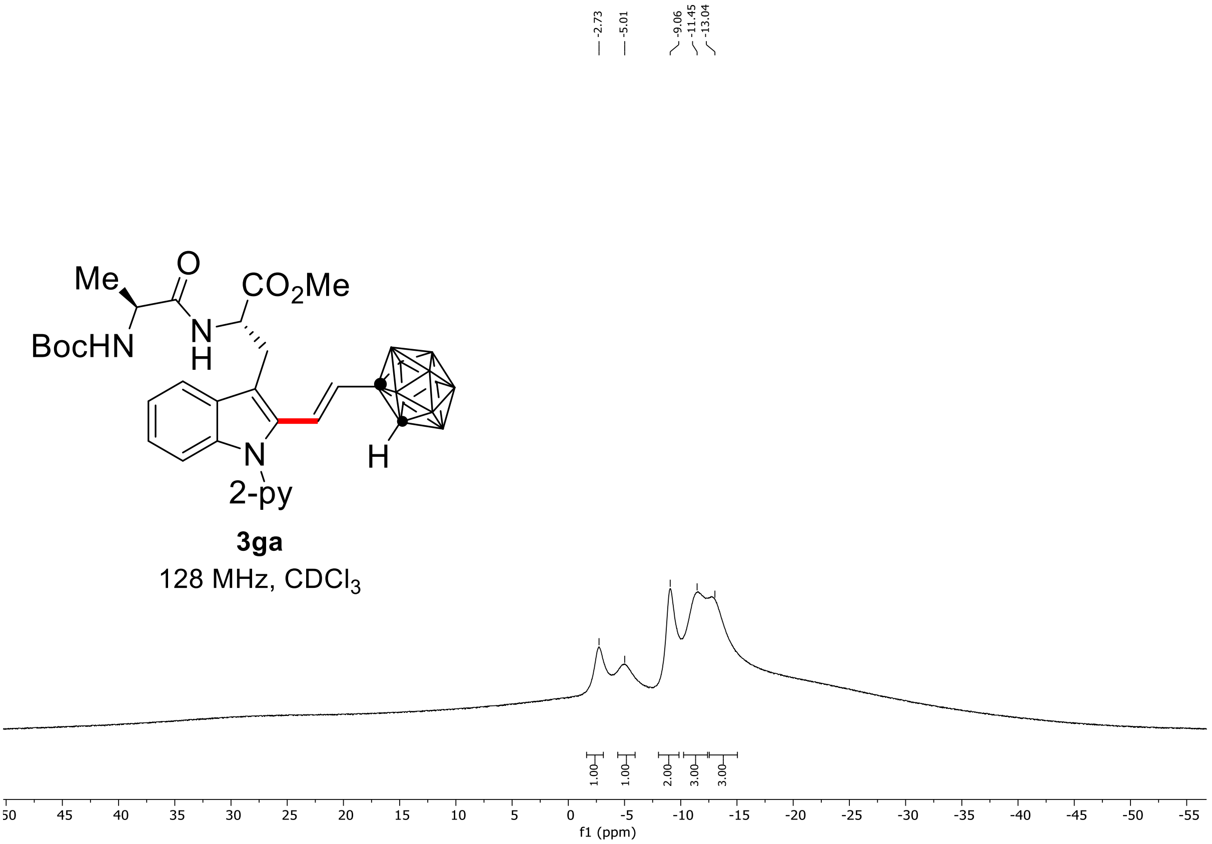
-2.73
-5.01
-9.06
-11.45
-13.04
Me
O
CO2Me
BocHN
N
H
N
2-py
H
3ga
128 MHz, CDCl3
1.00
1.00
2.00
3.00
3.00
f1 (ppm)

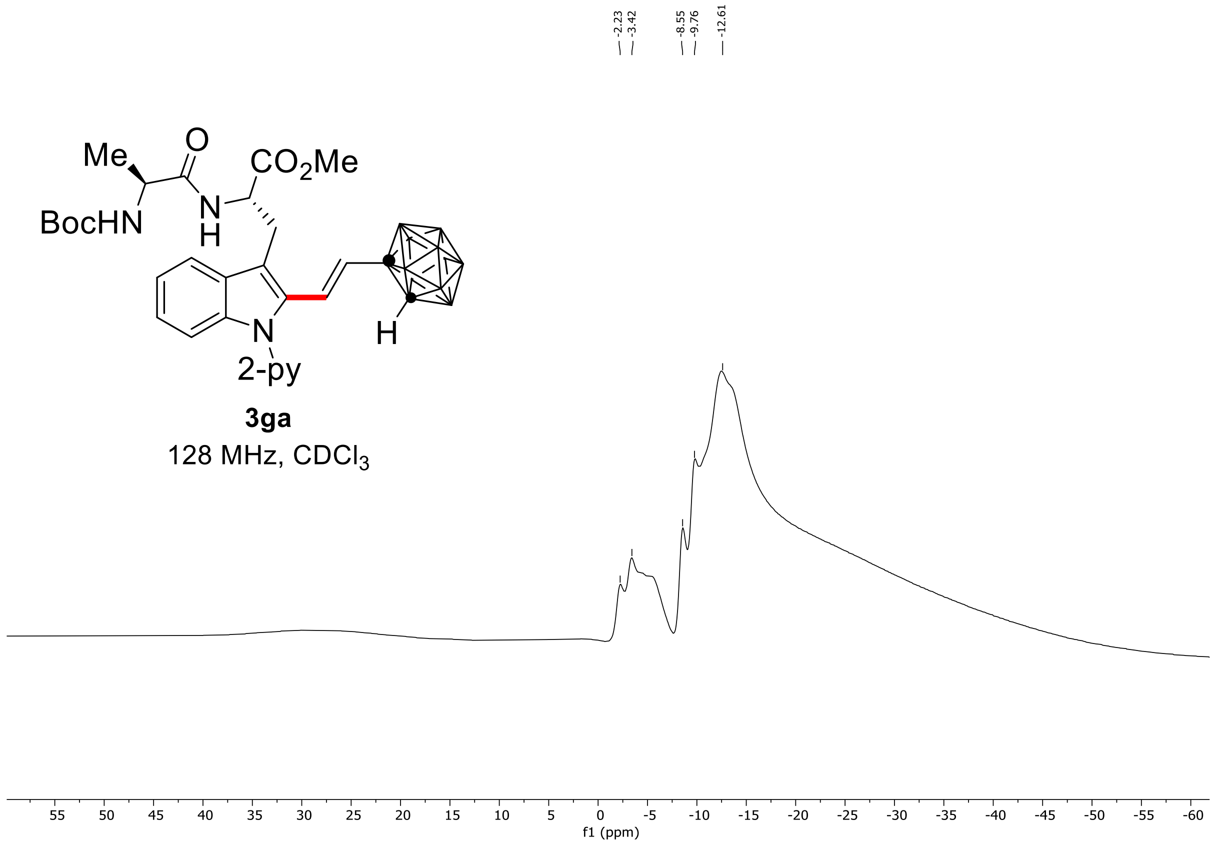

3ga
128 MHz, $CDCl_3$

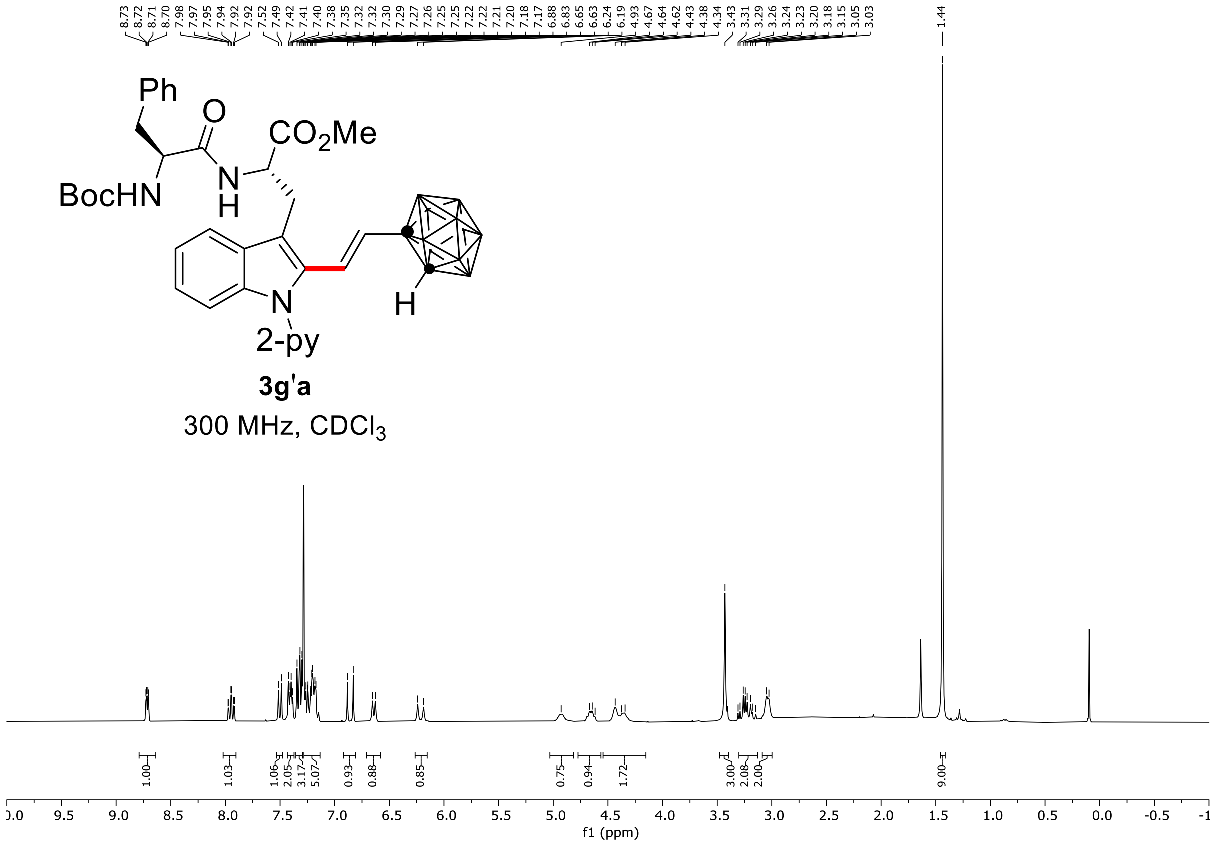

3g'a
300 MHz, $CDCl_3$

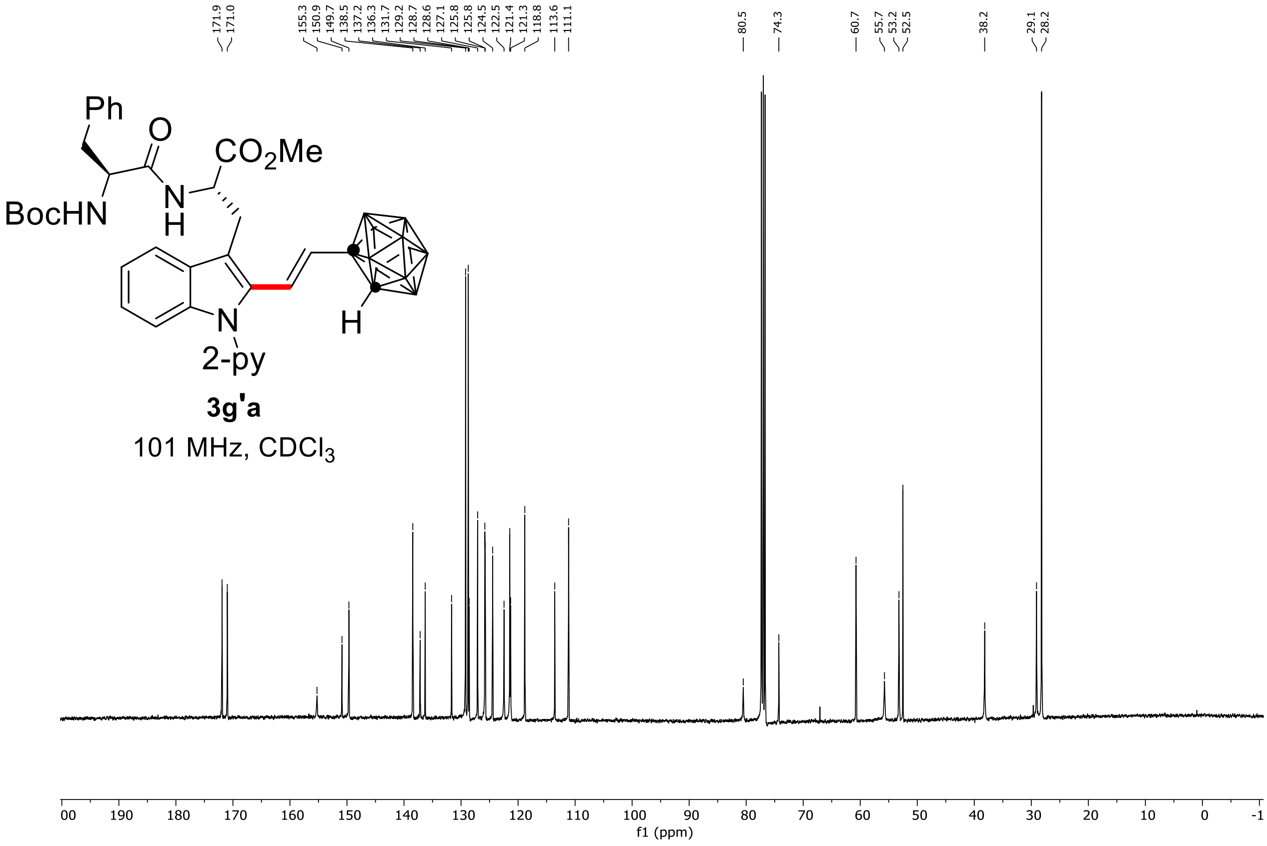
171.9
171.0
155.3
150.9
149.7
138.5
137.2
136.3
131.7
129.2
128.7
128.6
127.1
125.8
125.8
124.5
122.5
121.4
121.3
118.8
113.6
111.1
80.5
74.3
60.7
55.7
53.2
52.5
38.2
29.1
28.2
Ph
O
CO_2Me
BocHN
N
H
N
2-py
H
3g'a
101 MHz, $CDCl_3$
f1 (ppm)

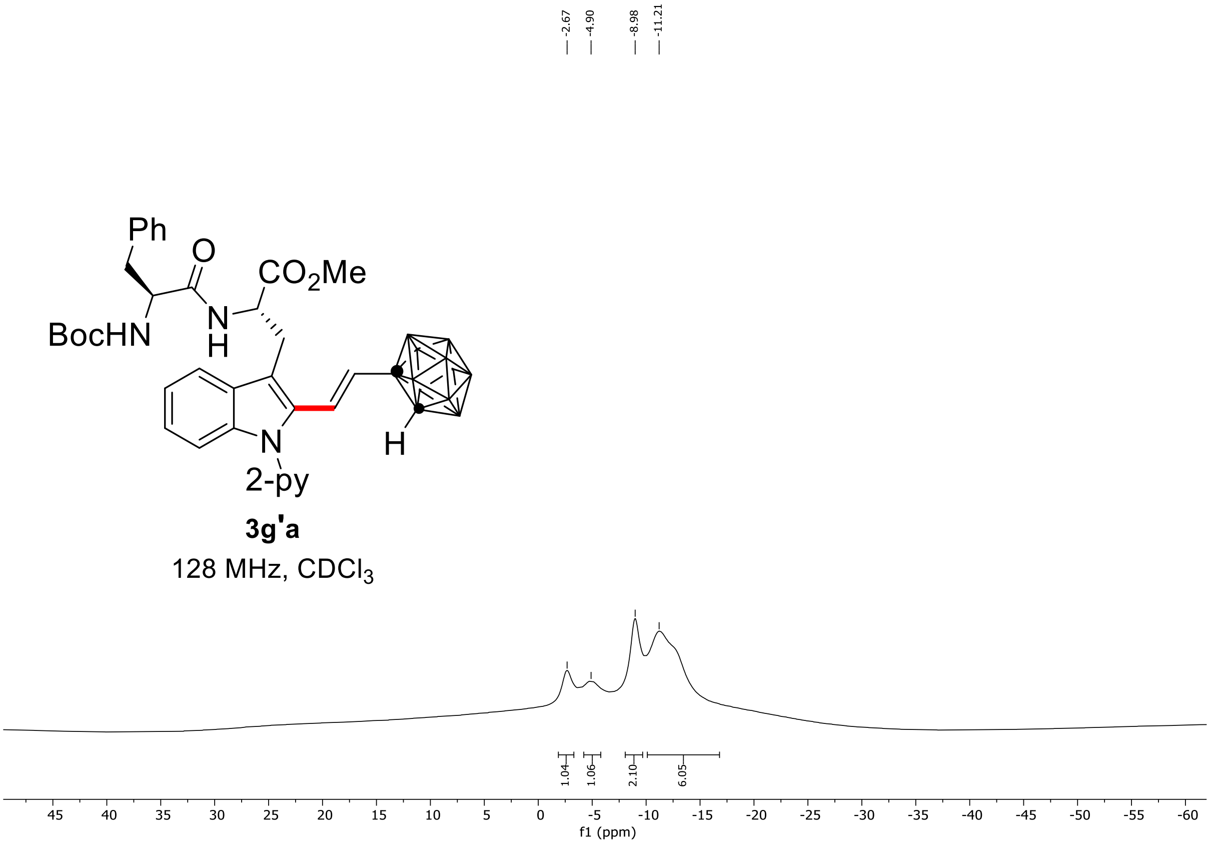
-2.67
-4.90
-8.98
-11.21
Ph
O
CO_2Me
BocHN
N
H
N
2-py
H
3g'a
128 MHz, $CDCl_3$
1.04
1.06
2.10
6.05
f1 (ppm)

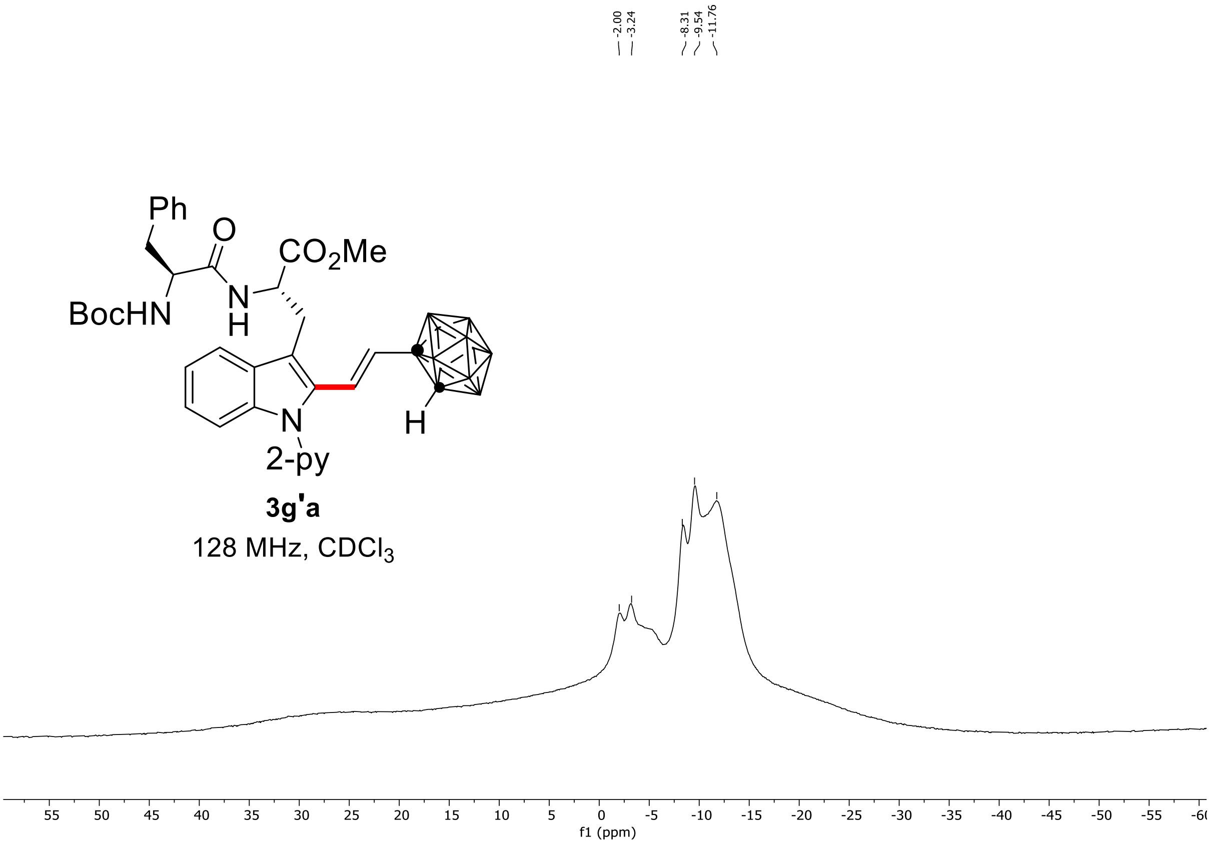
-2.00
-3.24
-8.31
-9.54
-11.76
Ph
O
CO2Me
BocHN
N
H
N
2-py
H
3g'a
128 MHz, CDCl3
f1 (ppm)

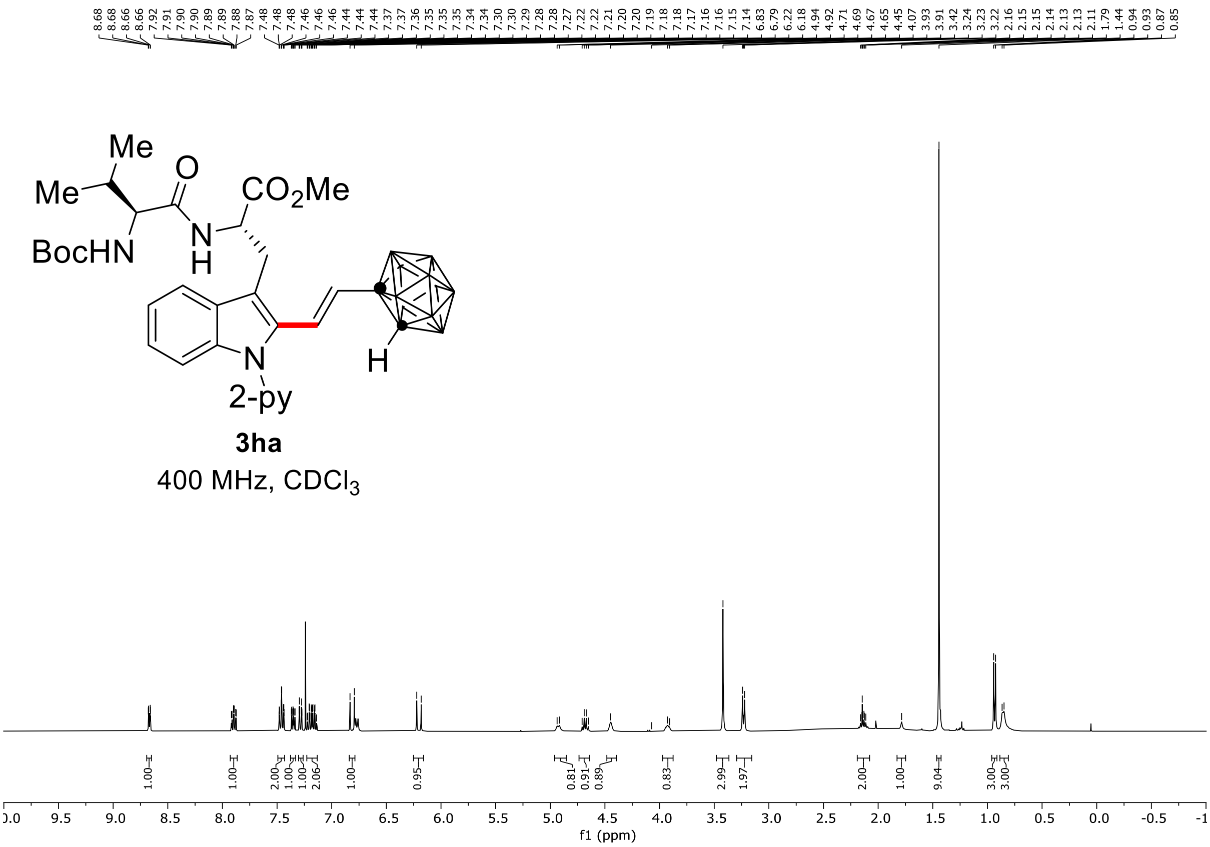
8.68
8.68
8.66
8.66
7.92
7.91
7.90
7.90
7.89
7.89
7.88
7.87
7.48
7.48
7.48
7.46
7.46
7.44
7.44
7.37
7.36
7.35
7.35
7.34
7.34
7.30
7.30
7.29
7.28
7.28
7.27
7.22
7.22
7.21
7.20
7.20
7.19
7.18
7.18
7.17
7.16
7.16
7.15
7.14
6.83
6.79
6.22
6.18
4.94
4.92
4.71
4.69
4.67
4.65
4.45
4.07
3.93
3.91
3.42
3.24
3.23
3.22
2.16
2.15
2.15
2.14
2.13
2.13
2.11
1.79
1.44
0.94
0.93
0.87
0.85
Me
Me
O
CO2Me
BocHN
N
H
N
2-py
H
3ha
400 MHz, CDCl3
1.00
1.00
2.00
1.00
1.00
2.06
1.00
0.95
0.81
0.91
0.89
0.83
2.99
1.97
2.00
1.00
9.04
3.00
3.00
f1 (ppm)

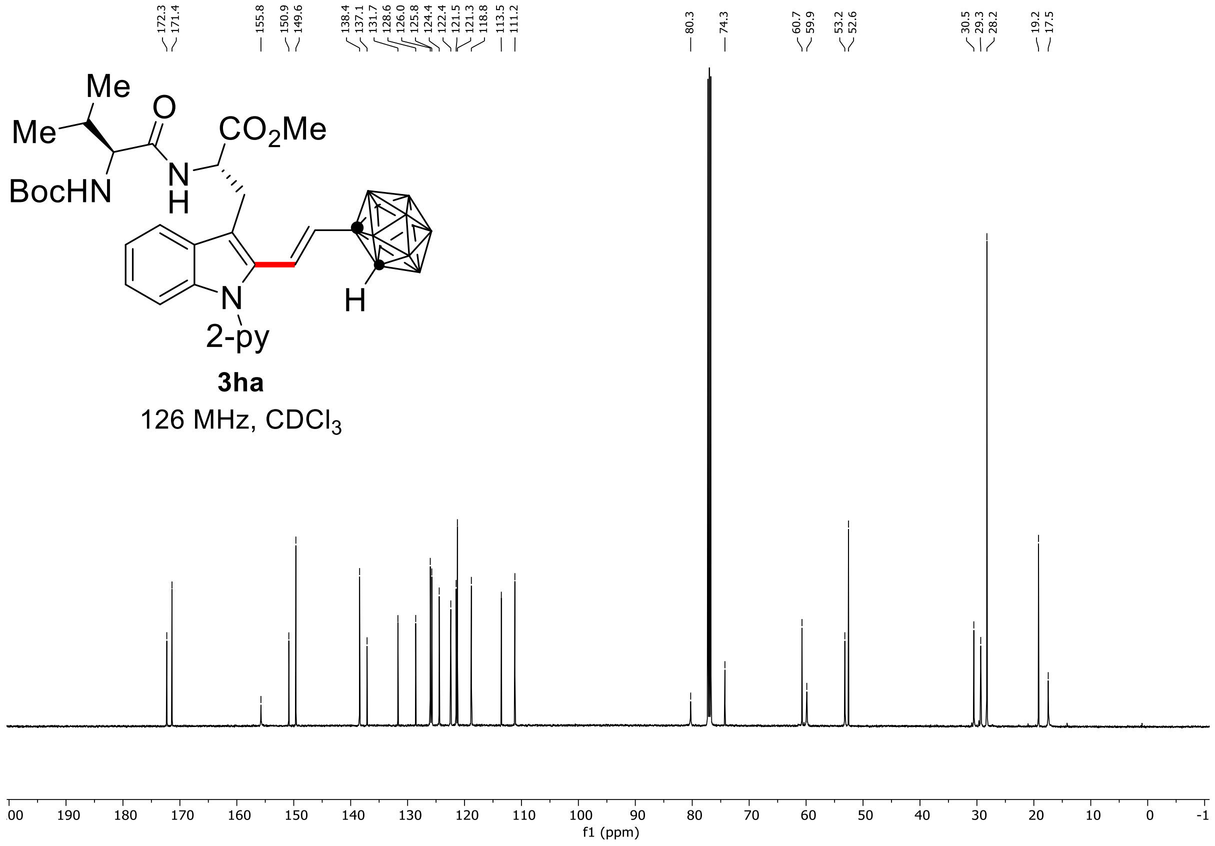
172.3
171.4
155.8
150.9
149.6
138.4
137.1
131.7
128.6
126.0
125.8
124.4
122.4
121.5
121.3
118.8
113.5
111.2
80.3
74.3
60.7
59.9
53.2
52.6
30.5
29.3
28.2
19.2
17.5
3ha
126 MHz, $CDCl_3$
f1 (ppm)

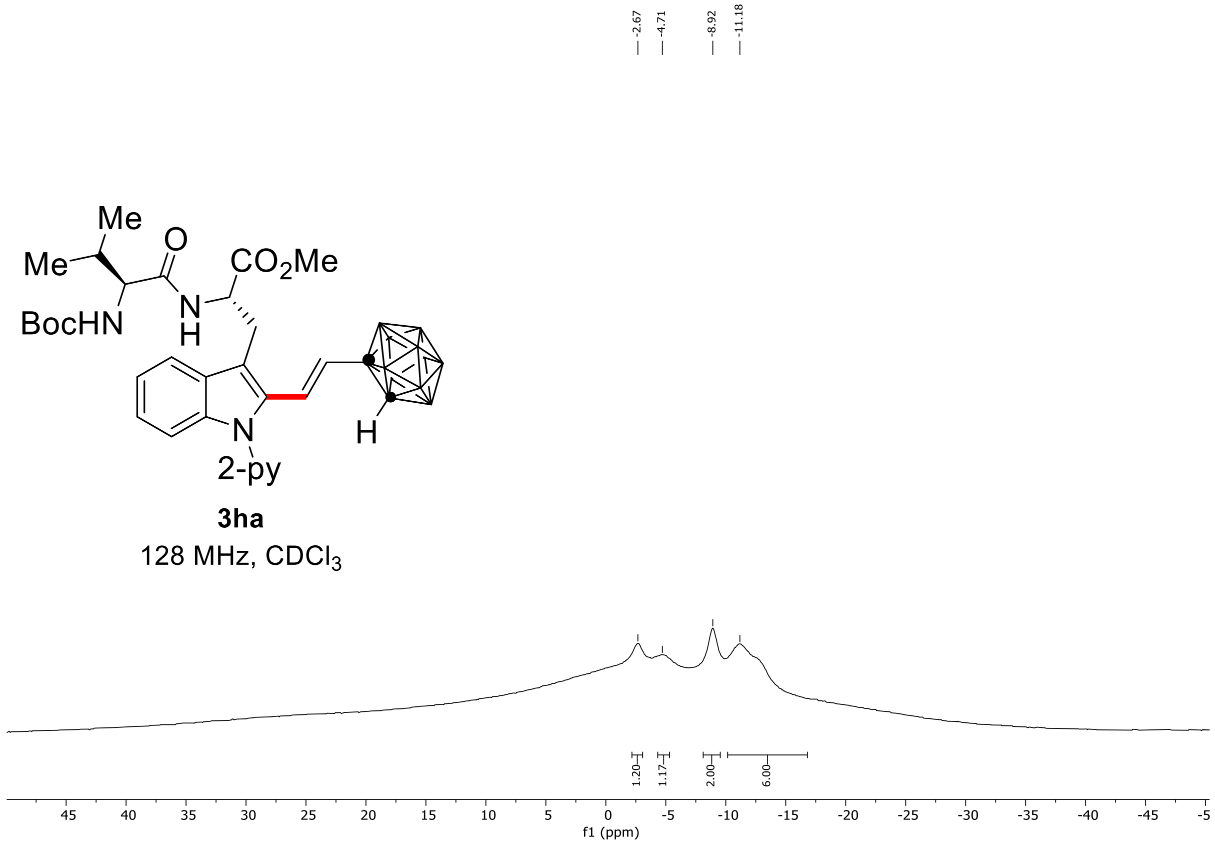
-2.67
-4.71
-8.92
-11.18
3ha
128 MHz, $CDCl_3$
1.20
1.17
2.00
6.00
f1 (ppm)

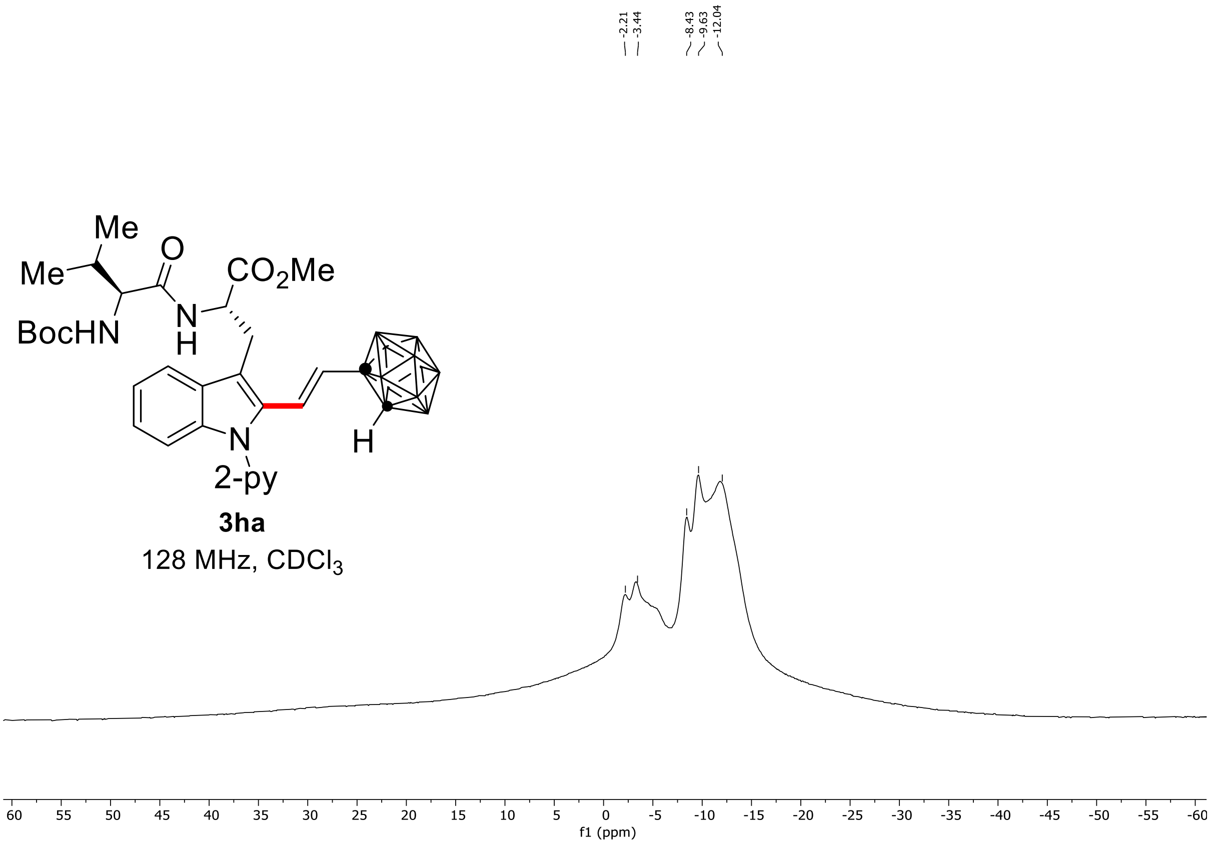
-2.21
-3.44
-8.43
-9.63
-12.04
Me
Me
O
CO2Me
BocHN
N
H
N
2-py
H
3ha
128 MHz, CDCl3
60 55 50 45 40 35 30 25 20 15 10 5 0 -5 -10 -15 -20 -25 -30 -35 -40 -45 -50 -55 -60
f1 (ppm)

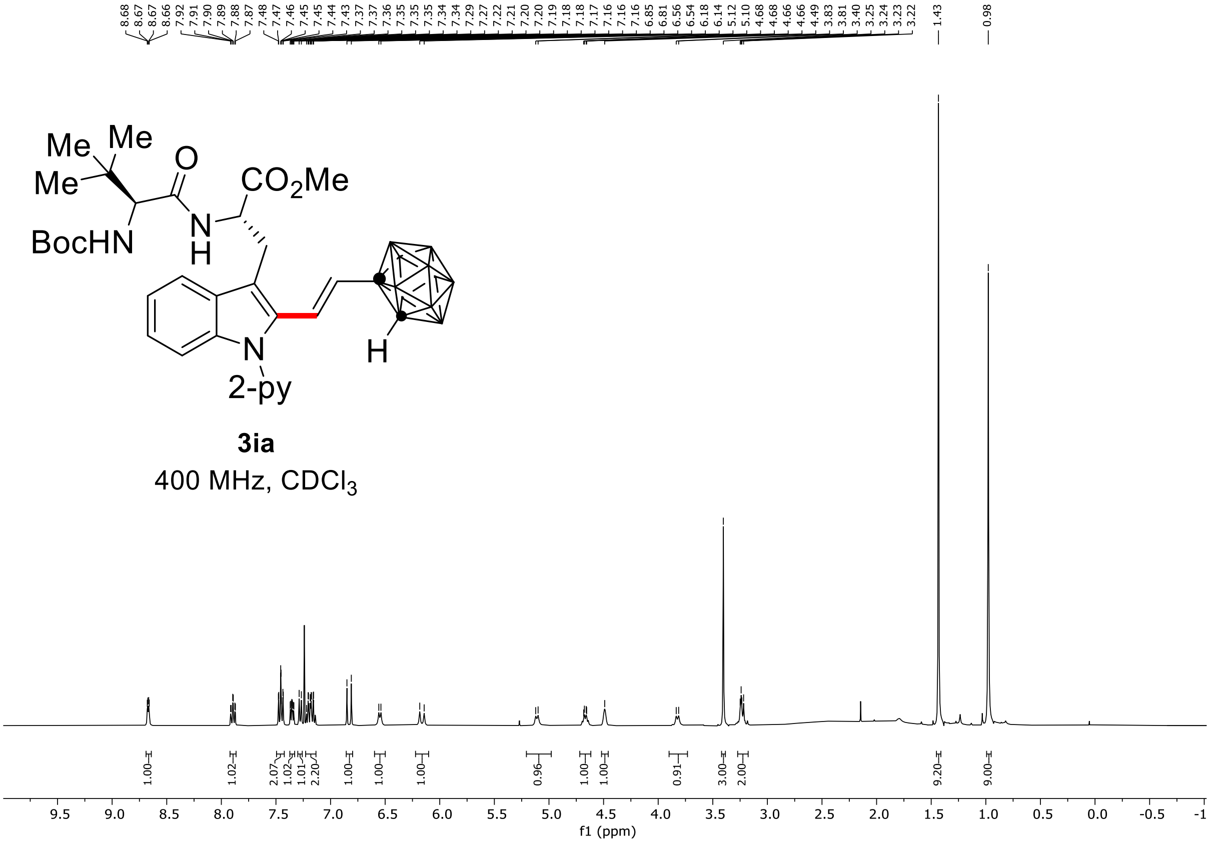
8.68 8.67 8.67 8.66 7.92 7.91 7.90 7.89 7.88 7.87 7.48 7.47 7.46 7.45 7.45 7.44 7.43 7.37 7.37 7.36 7.35 7.35 7.34 7.34 7.29 7.27 7.22 7.21 7.20 7.19 7.18 7.17 7.16 7.16 6.85 6.81 6.56 6.54 6.18 6.14 5.12 5.10 4.68 4.68 4.66 4.66 4.49 3.83 3.81 3.40 3.25 3.24 3.23 3.22
1.43
0.98
Me
Me
Me
O
CO2Me
BocHN
N
H
N
2-py
H
3ia
400 MHz, CDCl3
1.00 1.02 2.07 1.02 1.01 2.20 1.00 1.00 1.00 0.96 1.00 1.00 0.91 3.00 2.00 9.20 9.00
9.5 9.0 8.5 8.0 7.5 7.0 6.5 6.0 5.5 5.0 4.5 4.0 3.5 3.0 2.5 2.0 1.5 1.0 0.5 0.0 -0.5 -1
f1 (ppm)

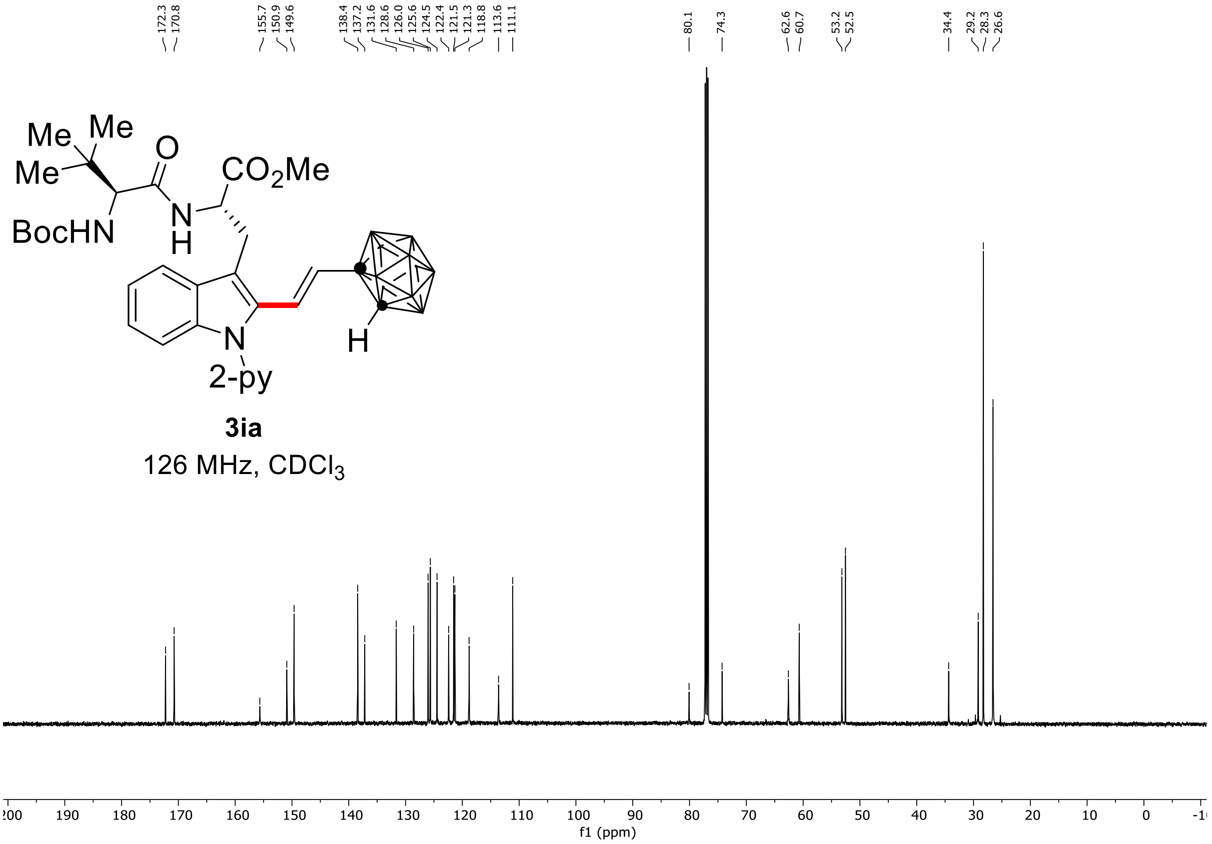

172.3
170.8
155.7
150.9
149.6
138.4
137.2
131.6
128.6
126.0
125.6
124.5
122.4
121.5
121.3
118.8
113.6
111.1
80.1
74.3
62.6
60.7
53.2
52.5
34.4
29.2
28.3
26.6
Me
Me
Me
O
CO2Me
N
H
BocHN
N
2-py
H
3ia
126 MHz, CDCl3
f1 (ppm)

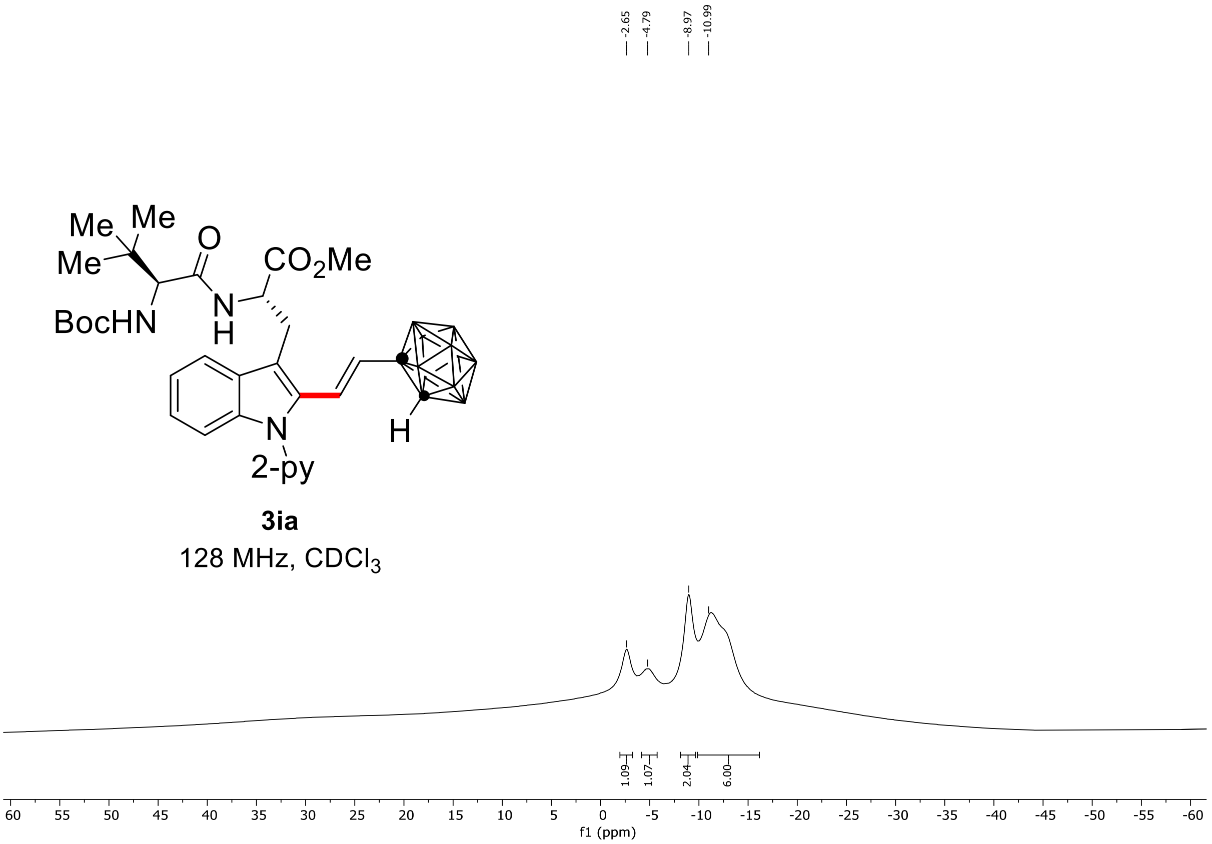

-2.65
-4.79
-8.97
-10.99
Me
Me
Me
O
CO2Me
N
H
BocHN
N
2-py
H
3ia
128 MHz, CDCl3
1.09
1.07
2.04
6.00
f1 (ppm)

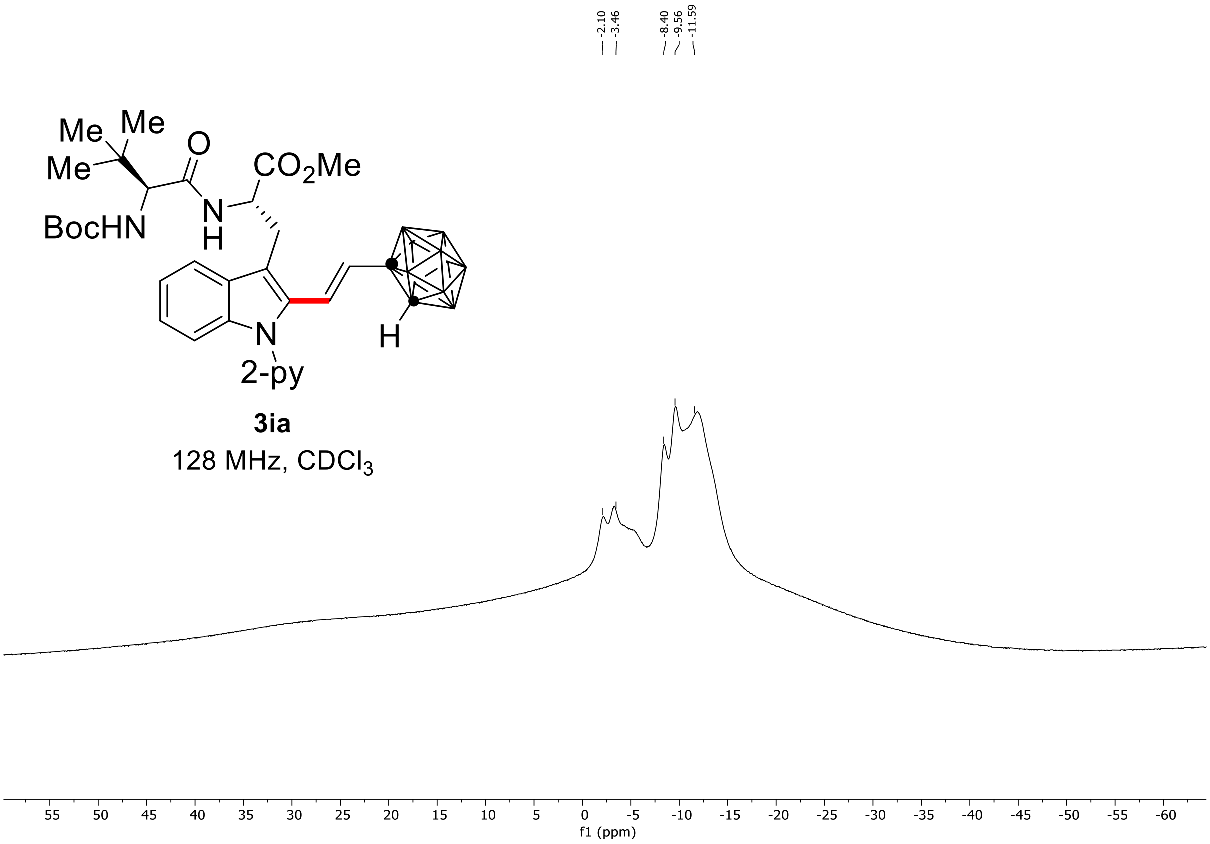
-2.10
-3.46
-8.40
-9.56
-11.59
3ia
128 MHz, CDCl3
f1 (ppm)

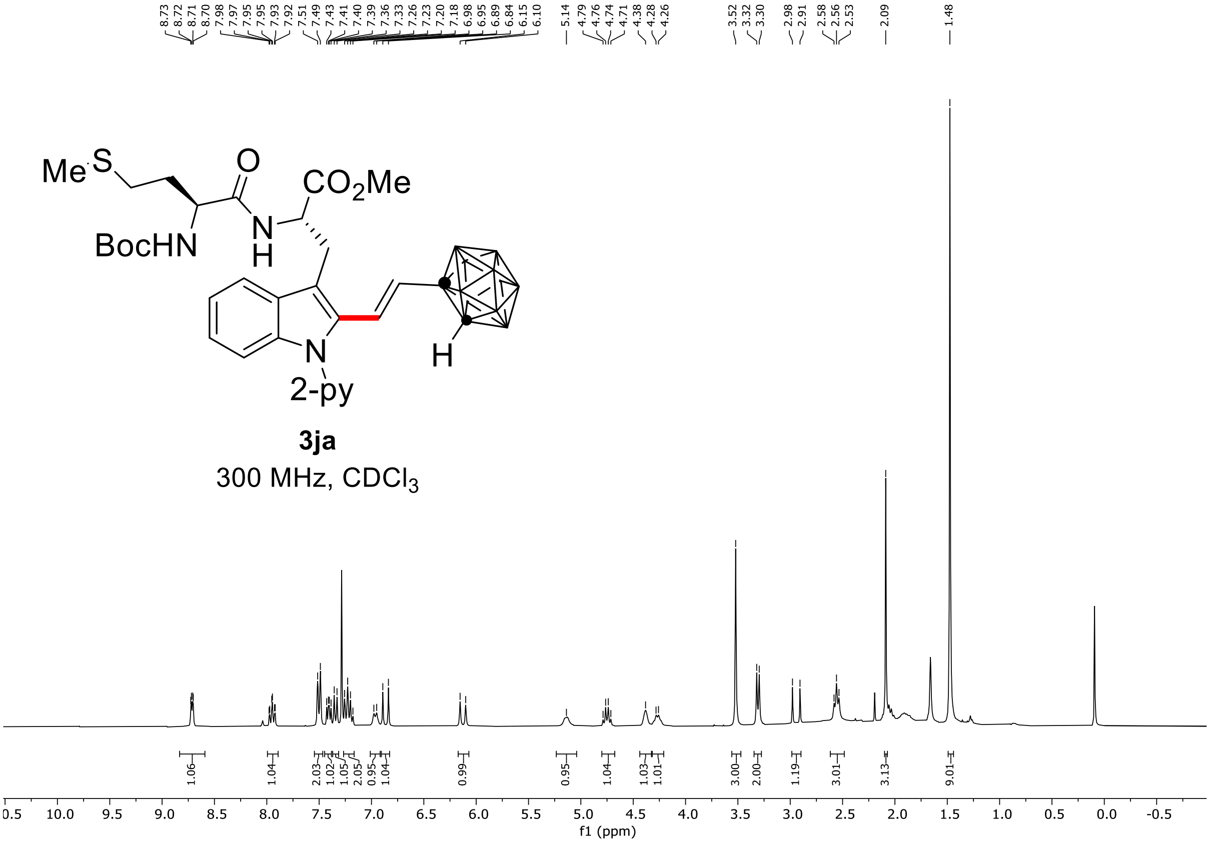
8.73
8.72
8.71
8.70
7.98
7.97
7.95
7.93
7.92
7.51
7.49
7.43
7.41
7.40
7.39
7.36
7.33
7.26
7.23
7.20
7.18
6.98
6.95
6.89
6.84
6.15
6.10
5.14
4.79
4.76
4.74
4.71
4.38
4.28
4.26
3.52
3.32
3.30
2.98
2.91
2.58
2.56
2.53
2.09
1.48
3ja
300 MHz, CDCl3
1.06
1.04
2.03
1.02
1.05
2.05
0.95
1.04
0.99
0.95
1.04
1.03
1.01
3.00
2.00
1.19
3.01
3.13
9.01
f1 (ppm)

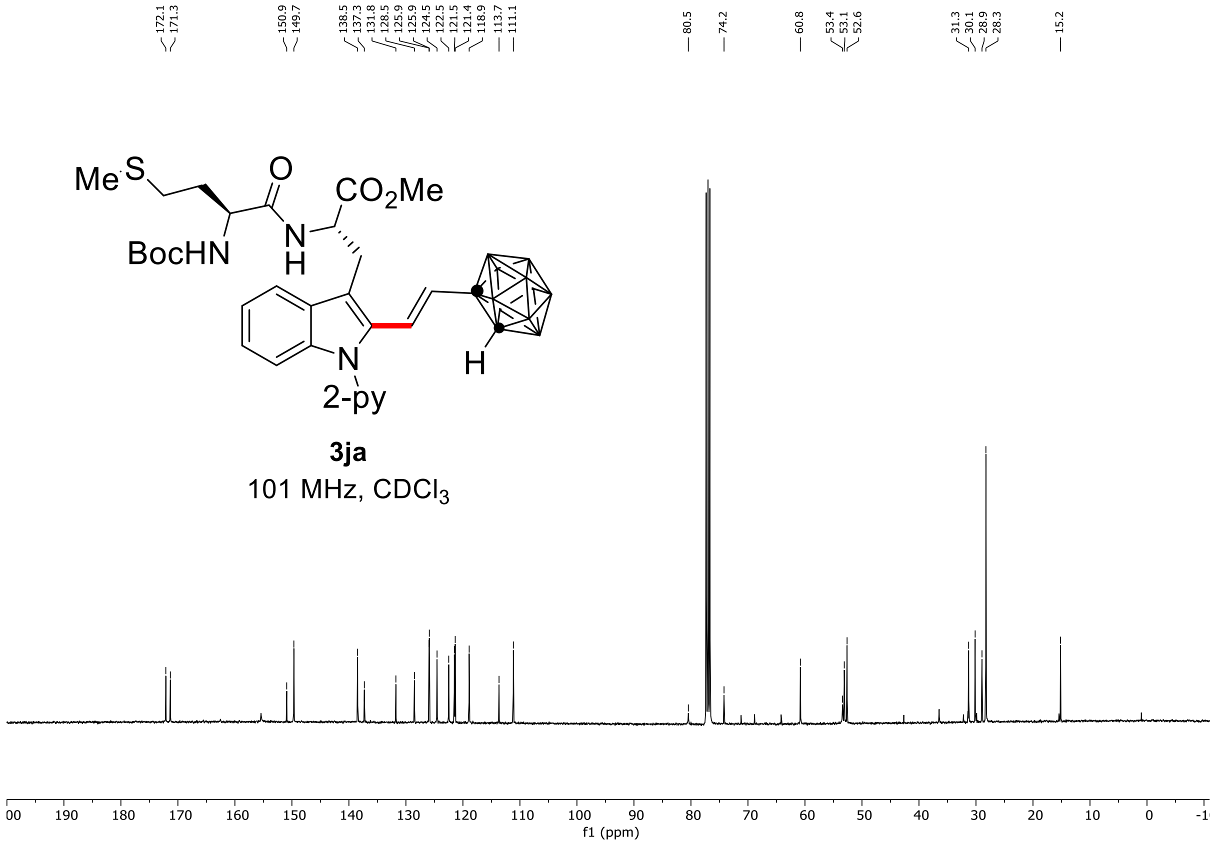
172.1
171.3
150.9
149.7
138.5
137.3
131.8
128.5
125.9
125.9
124.5
122.5
121.5
121.4
118.9
113.7
111.1
80.5
74.2
60.8
53.4
53.1
52.6
31.3
30.1
28.9
28.3
15.2
Me S
O
CO2Me
N
H
BocHN
N
2-py
H
3ja
101 MHz, CDCl3
f1 (ppm)

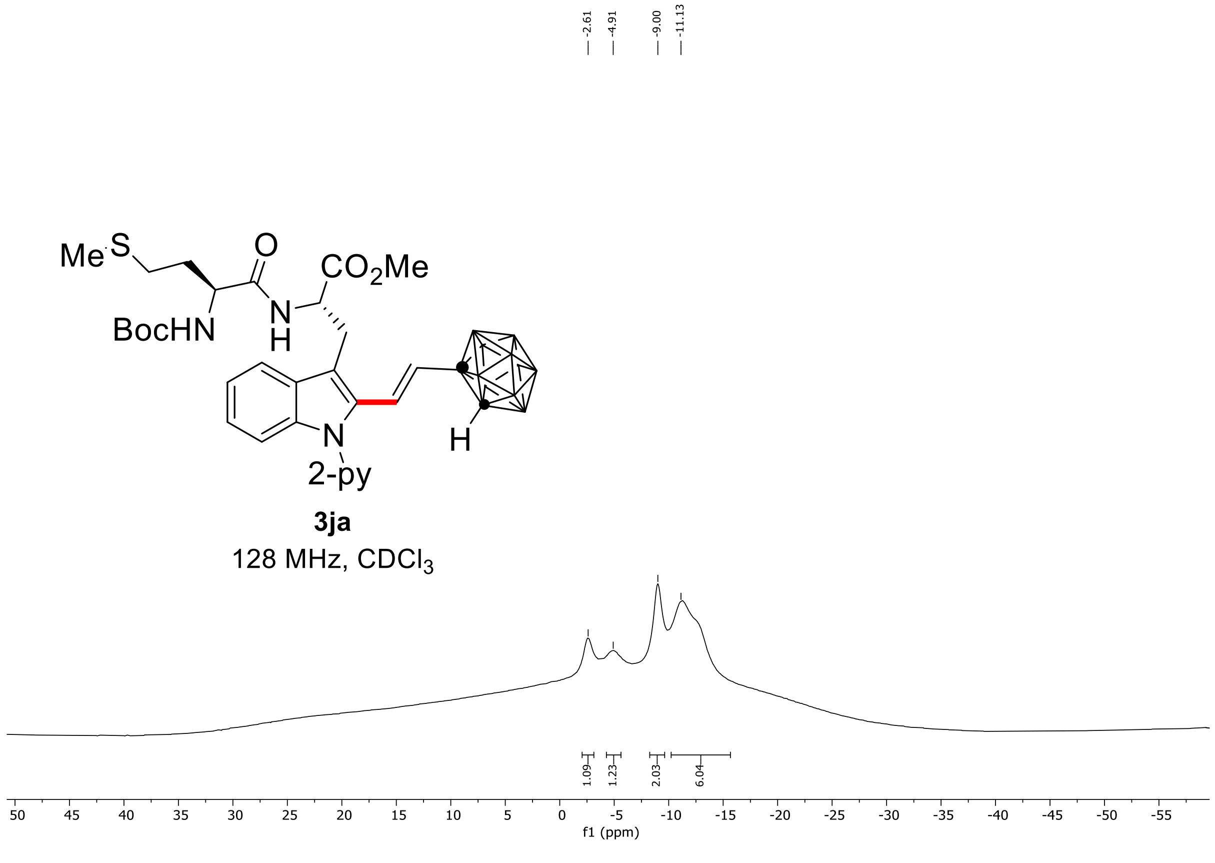
-2.61
-4.91
-9.00
-11.13
Me S
O
CO2Me
N
H
BocHN
N
2-py
H
3ja
128 MHz, CDCl3
1.09
1.23
2.03
6.04
f1 (ppm)

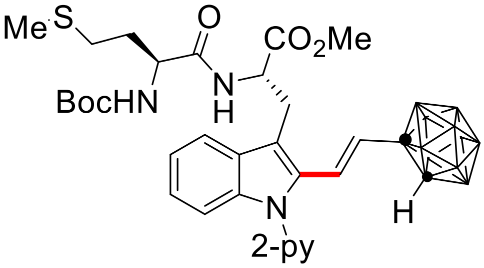

3ja

128 MHz, $CDCl_3$

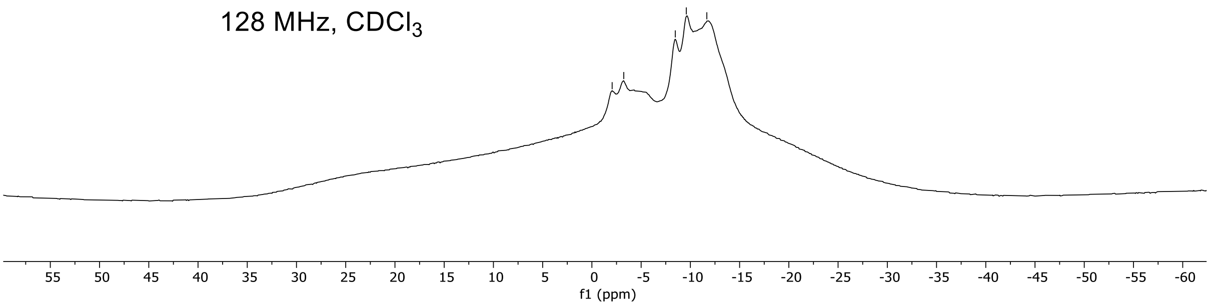

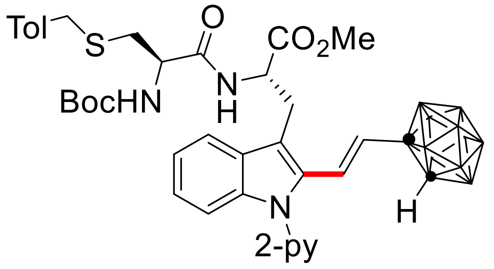

3ka

300 MHz, $CDCl_3$

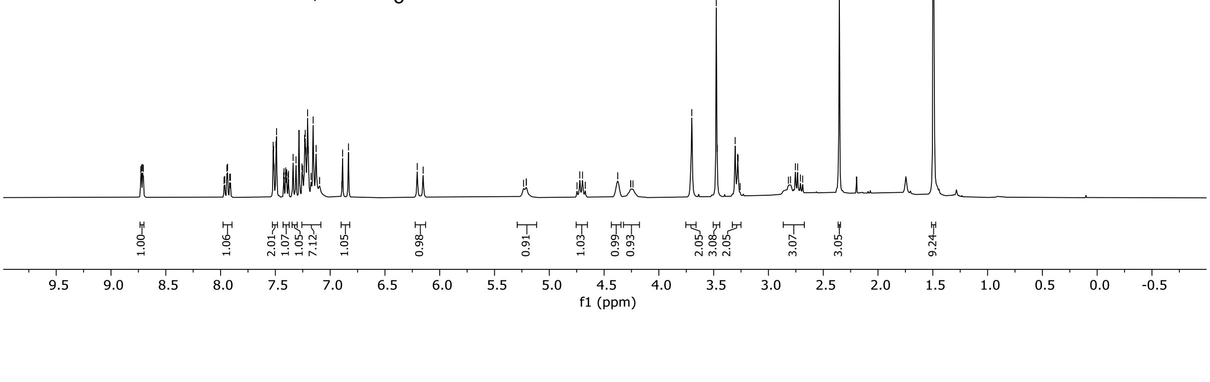

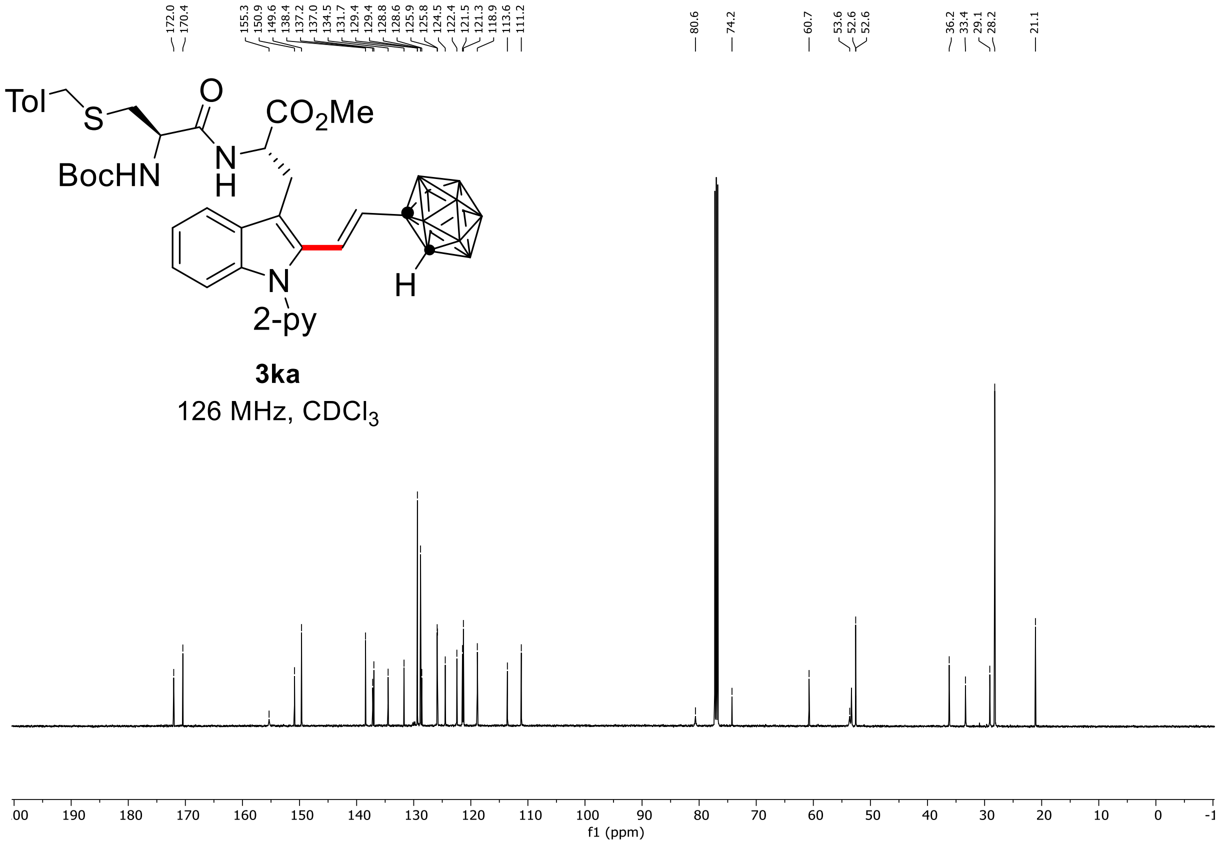
172.0
170.4
155.3
150.9
149.6
138.4
137.2
137.0
134.5
131.7
129.4
129.4
128.8
128.6
125.9
125.8
124.5
122.4
121.5
121.3
118.9
113.6
111.2
80.6
74.2
60.7
53.6
52.6
52.6
36.2
33.4
29.1
28.2
21.1
Tol
S
O
CO2Me
N
H
BocHN
N
2-py
H
3ka
126 MHz, CDCl3
f1 (ppm)

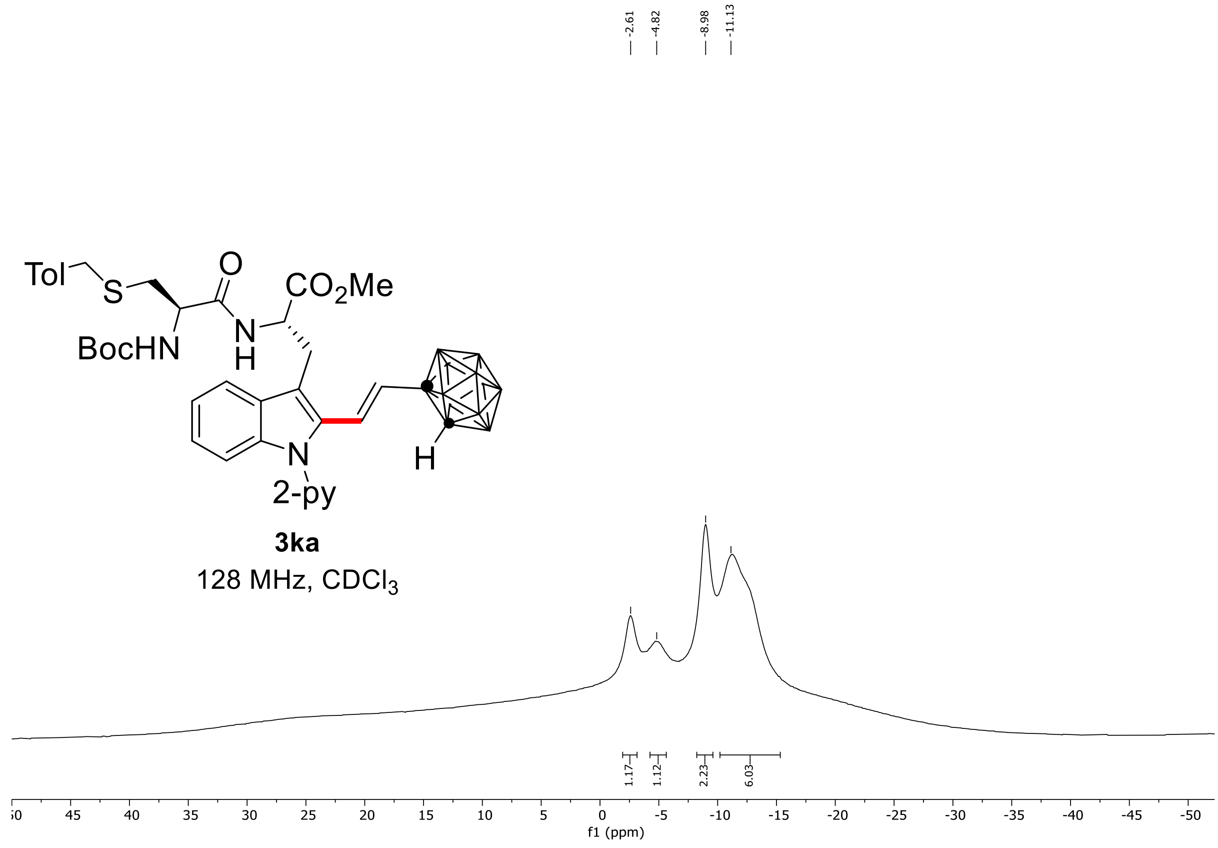
-2.61
-4.82
-8.98
-11.13
Tol
S
O
CO2Me
N
H
BocHN
N
2-py
H
3ka
128 MHz, CDCl3
1.17
1.12
2.23
6.03
f1 (ppm)

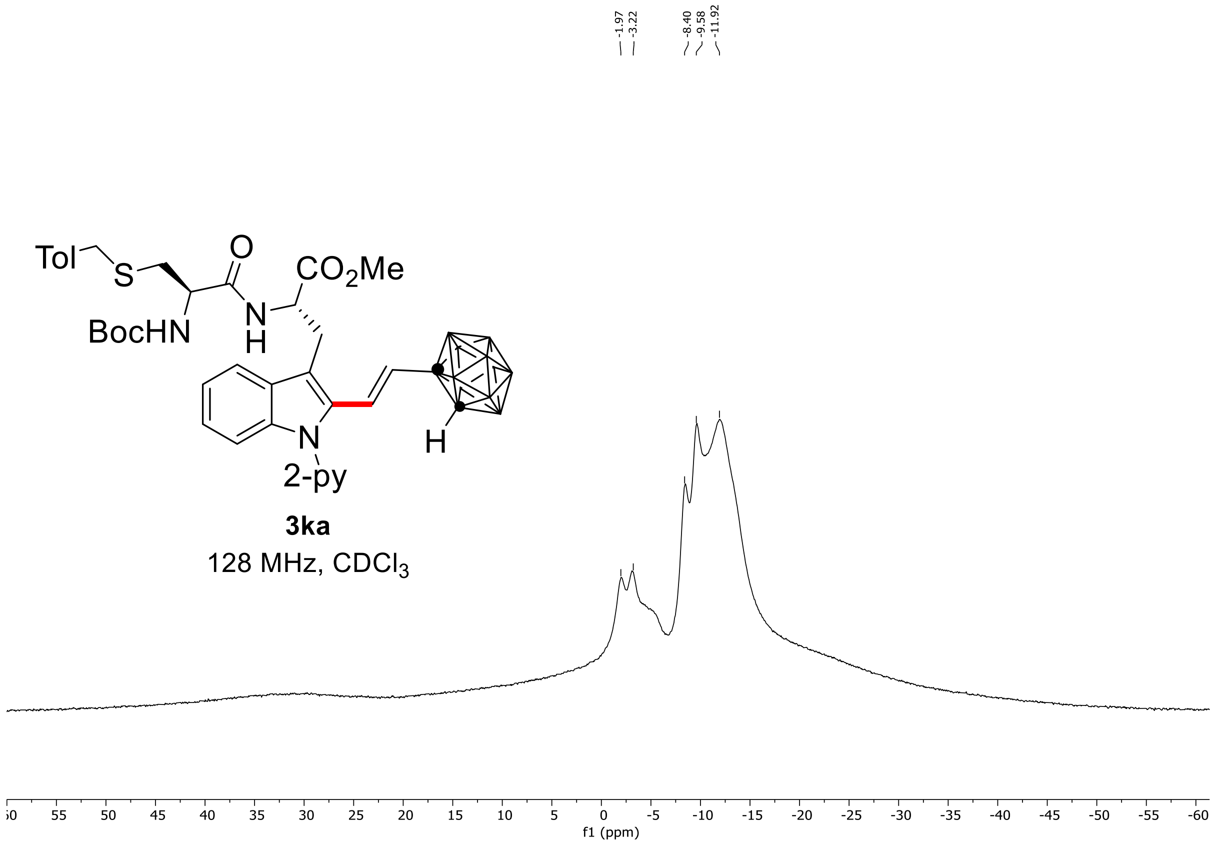

3ka
128 MHz, $CDCl_3$

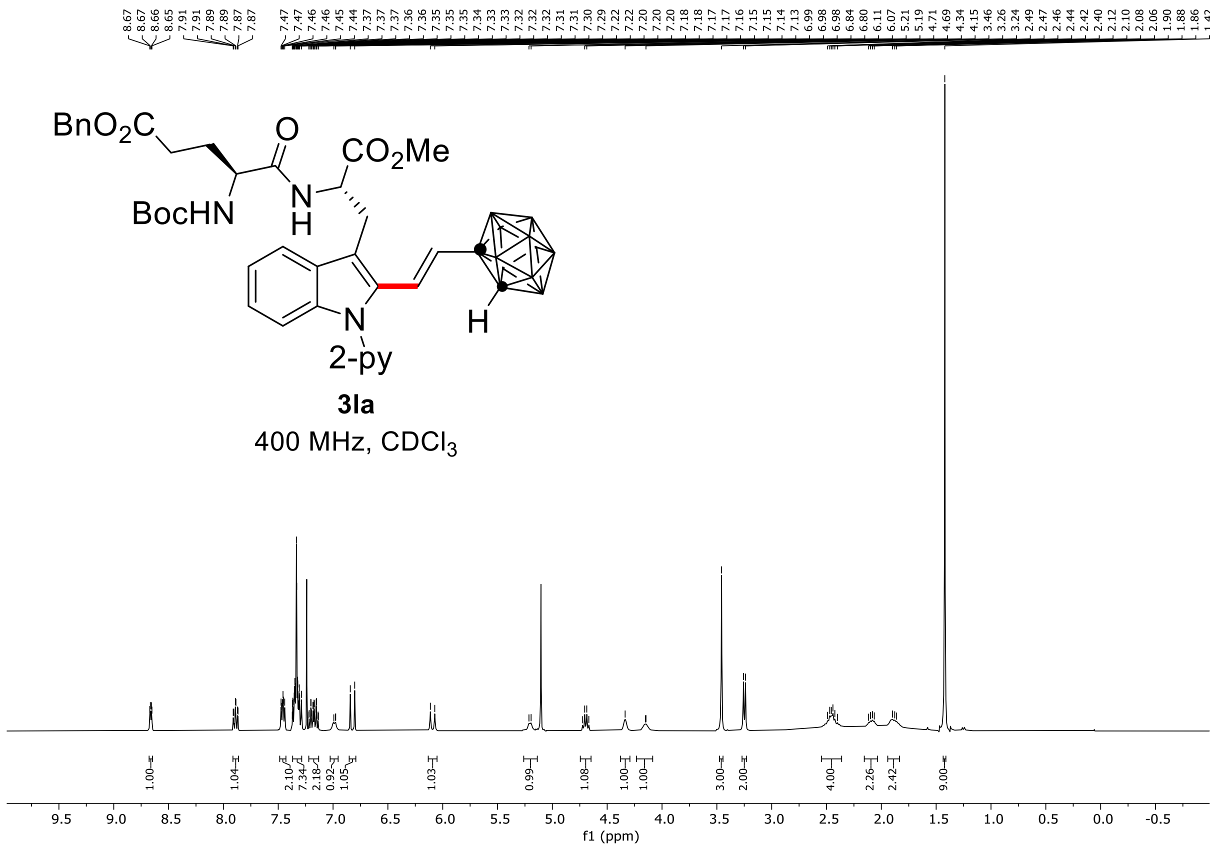

3la
400 MHz, $CDCl_3$

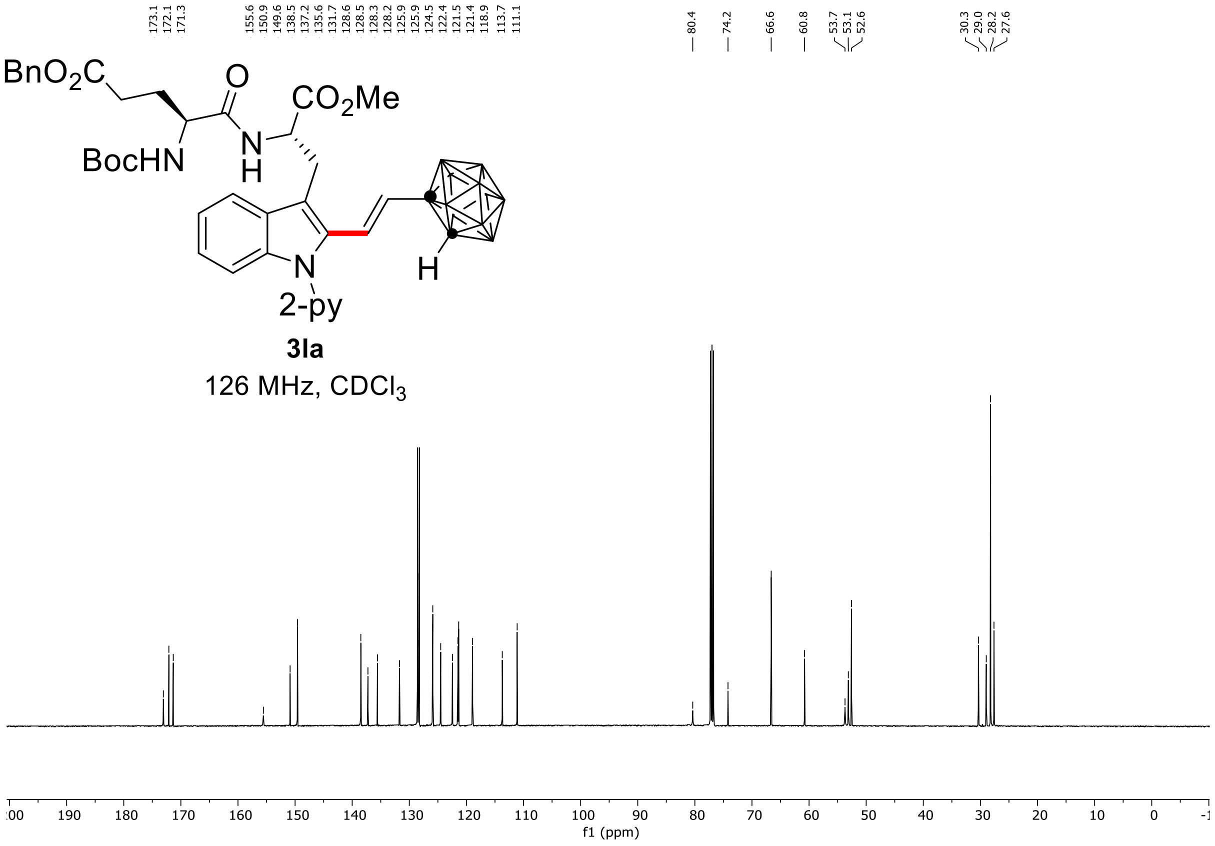
173.1
172.1
171.3
155.6
150.9
149.6
138.5
137.2
135.6
131.7
128.6
128.5
128.3
128.2
125.9
125.9
124.5
122.4
121.5
121.4
118.9
113.7
111.1
80.4
74.2
66.6
60.8
53.7
53.1
52.6
30.3
29.0
28.2
27.6
BnO2C
O
CO2Me
N
H
BocHN
N
2-py
H
3la
126 MHz, CDCl3
f1 (ppm)

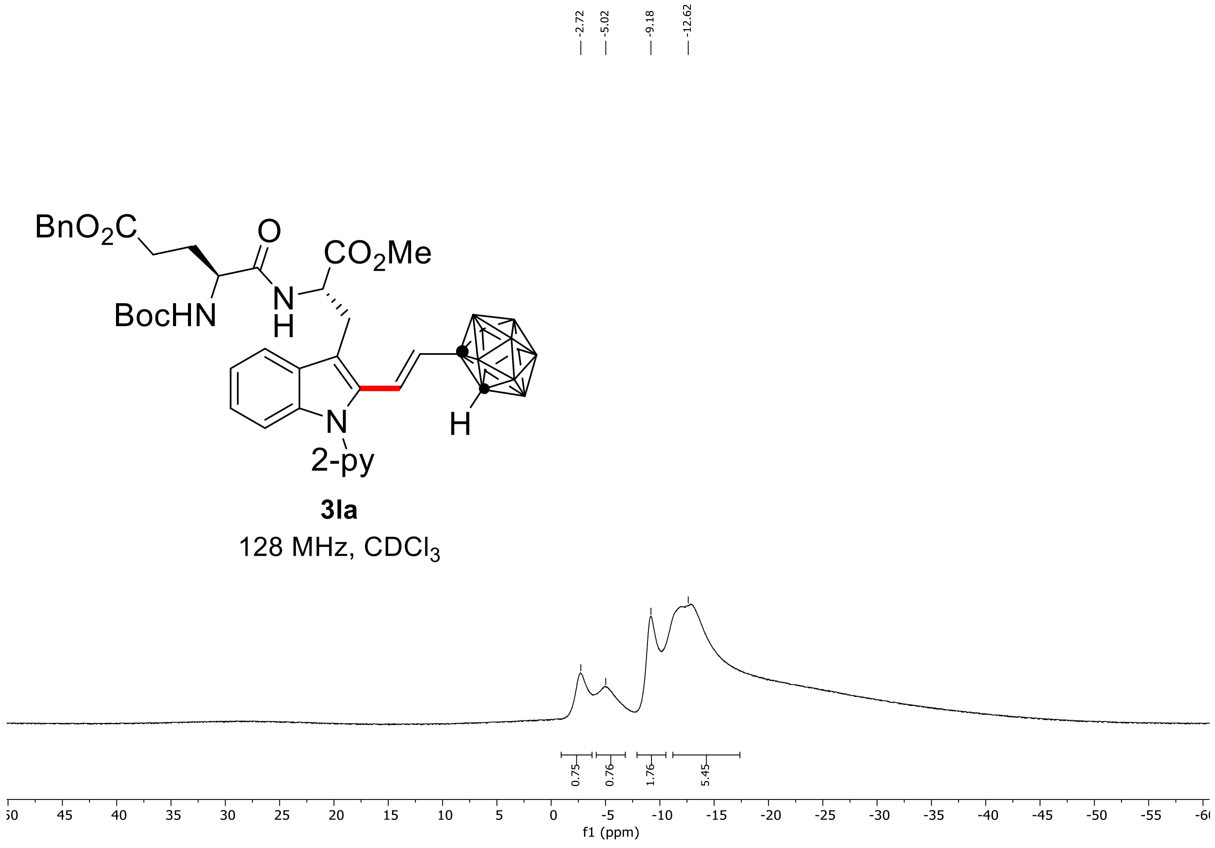
-2.72
-5.02
-9.18
-12.62
BnO2C
O
CO2Me
N
H
BocHN
N
2-py
H
3la
128 MHz, CDCl3
0.75
0.76
1.76
5.45
f1 (ppm)

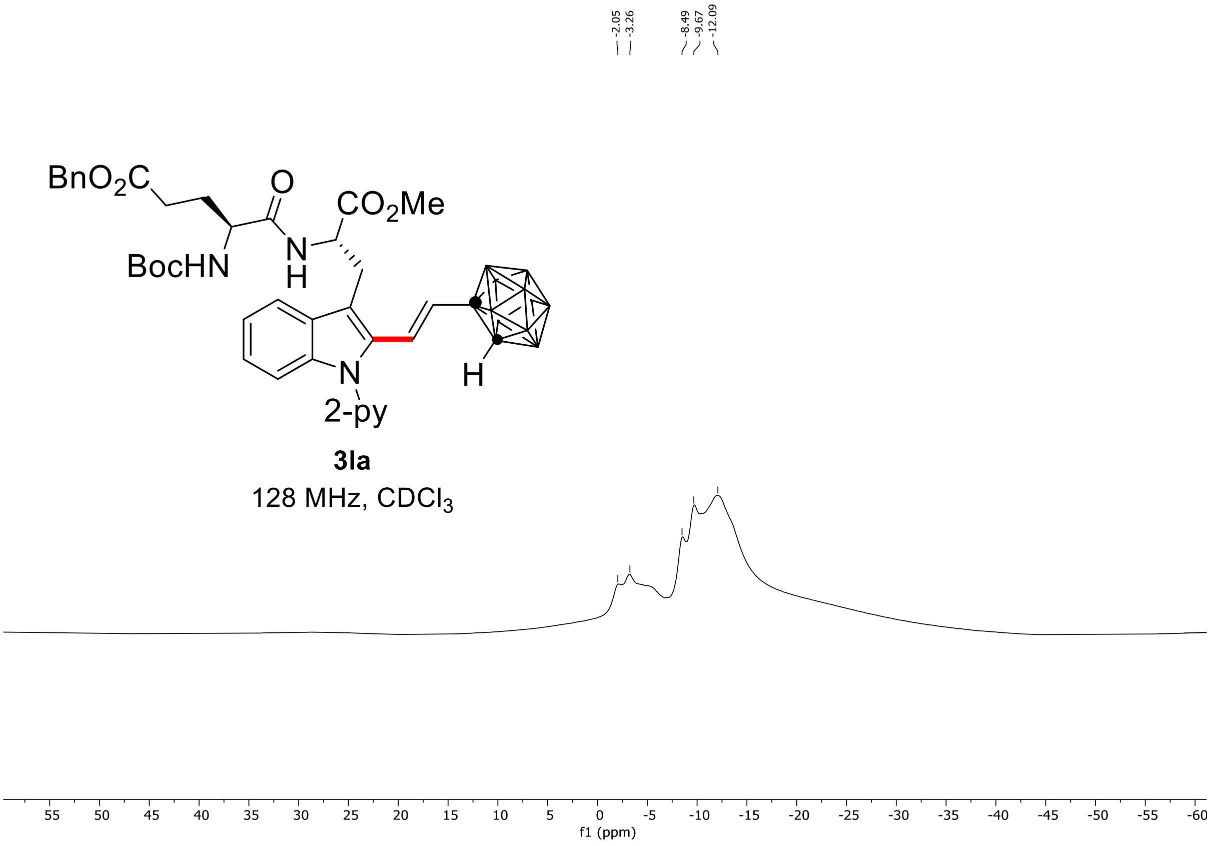
BnO2C
CO2Me
BocHN
H
2-py
3la
128 MHz, CDCl3
f1 (ppm)

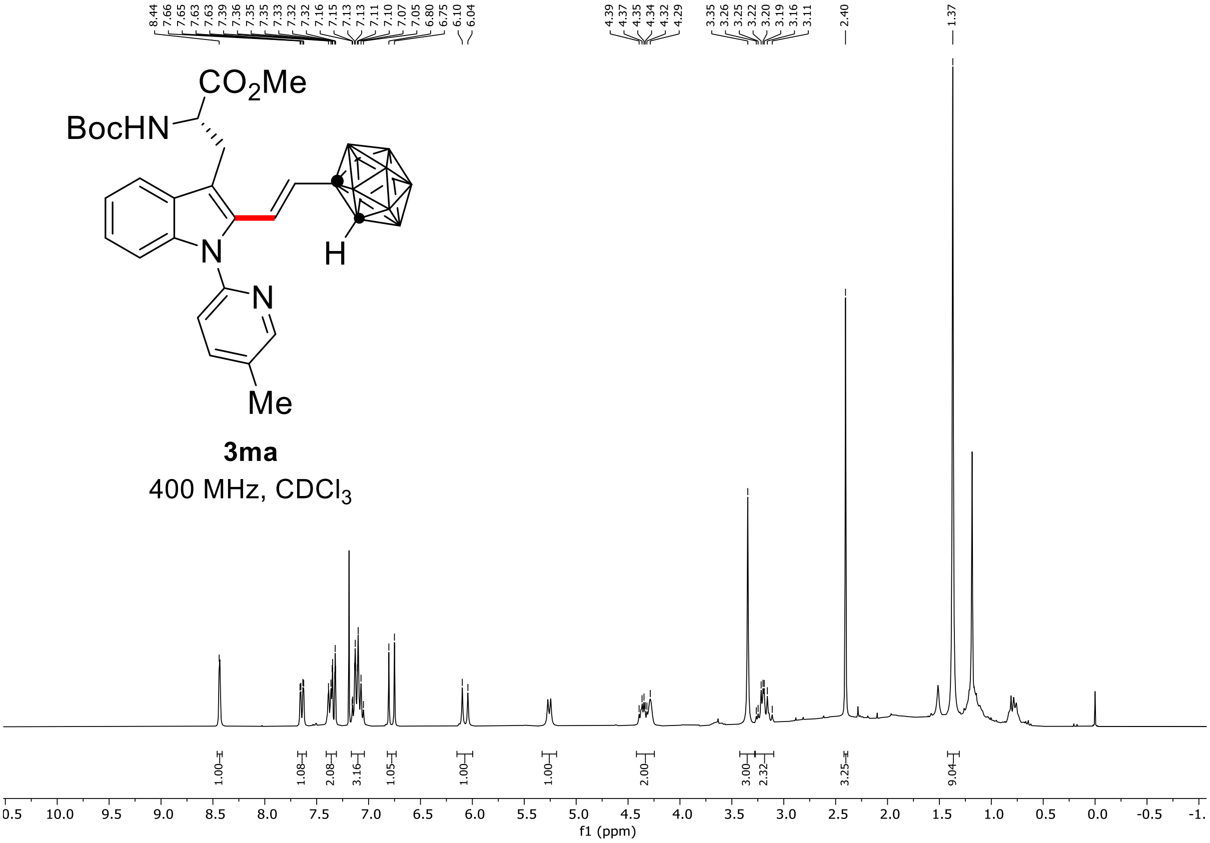
CO2Me
BocHN
Me
3ma
400 MHz, CDCl3
f1 (ppm)

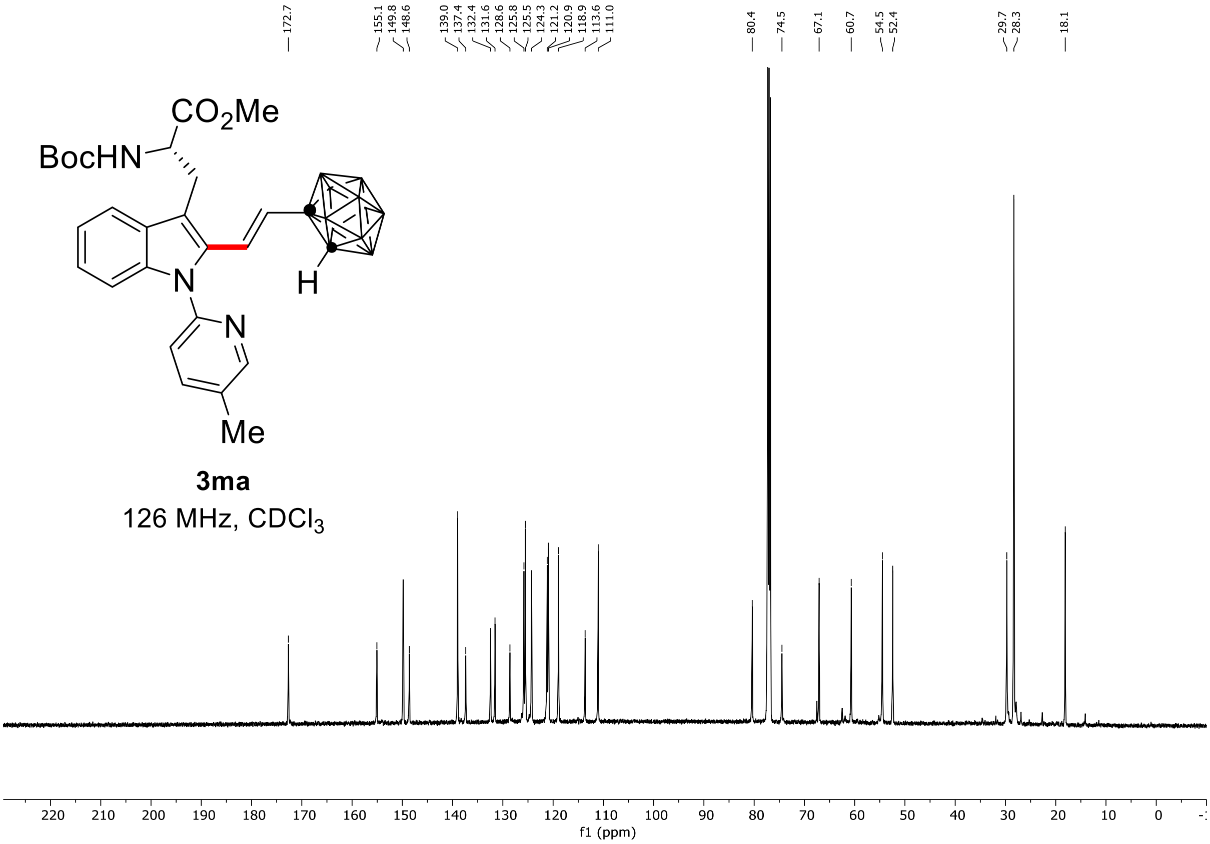

3ma

126 MHz, $CDCl_3$

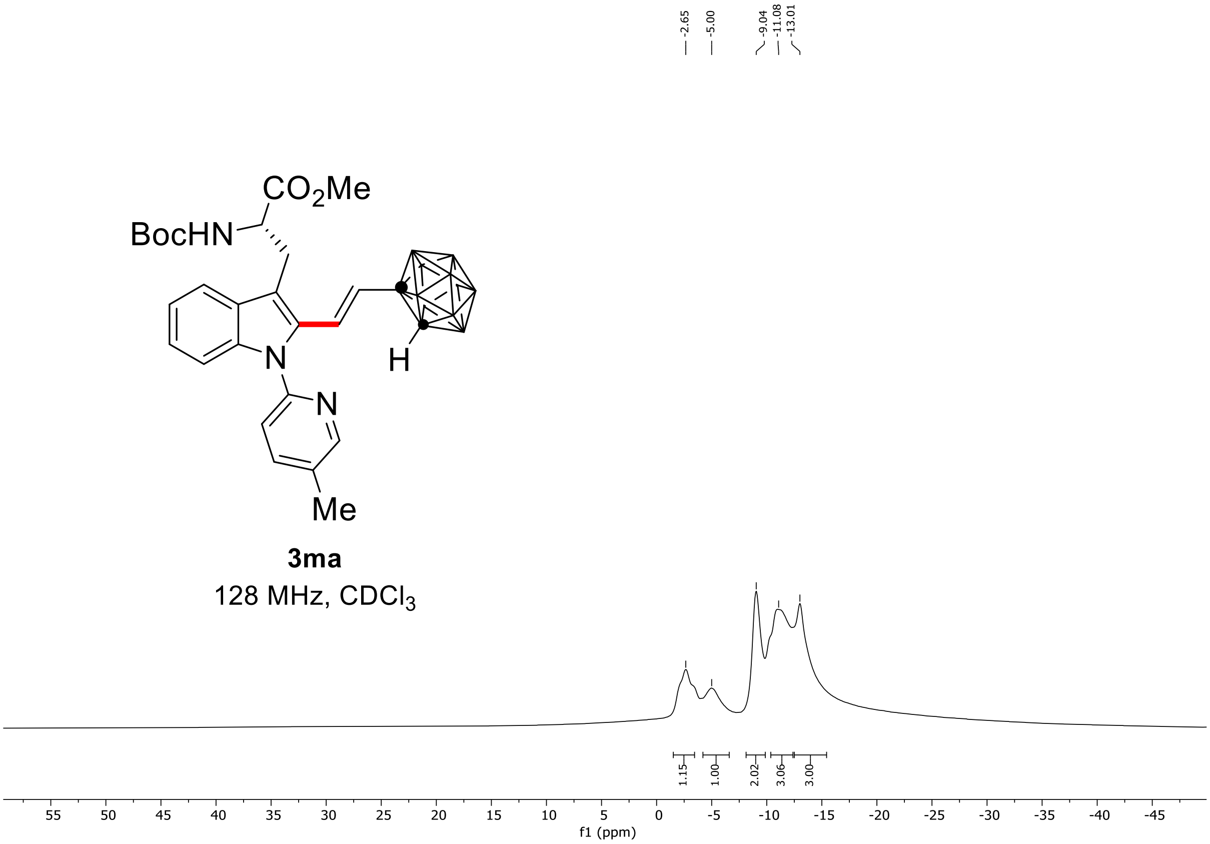

3ma

128 MHz, $CDCl_3$

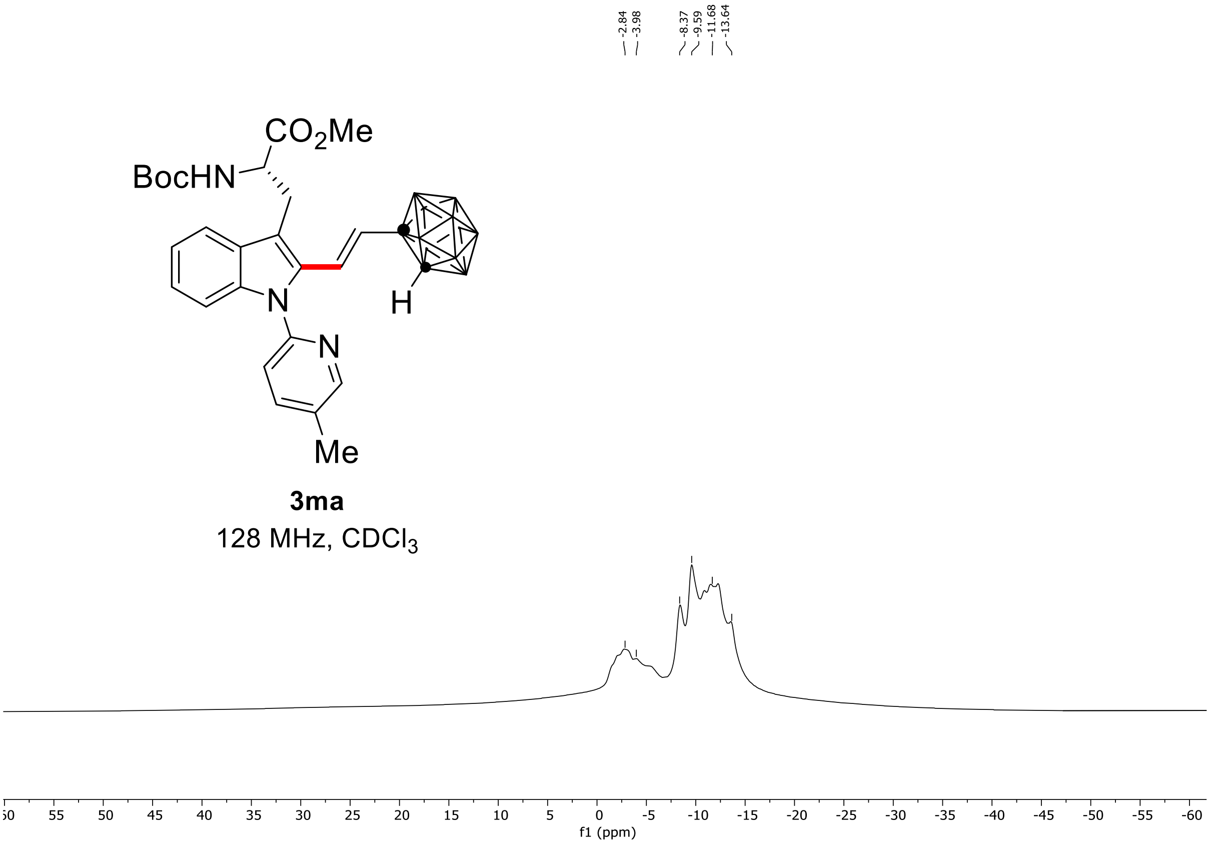

3ma
128 MHz, $CDCl_3$

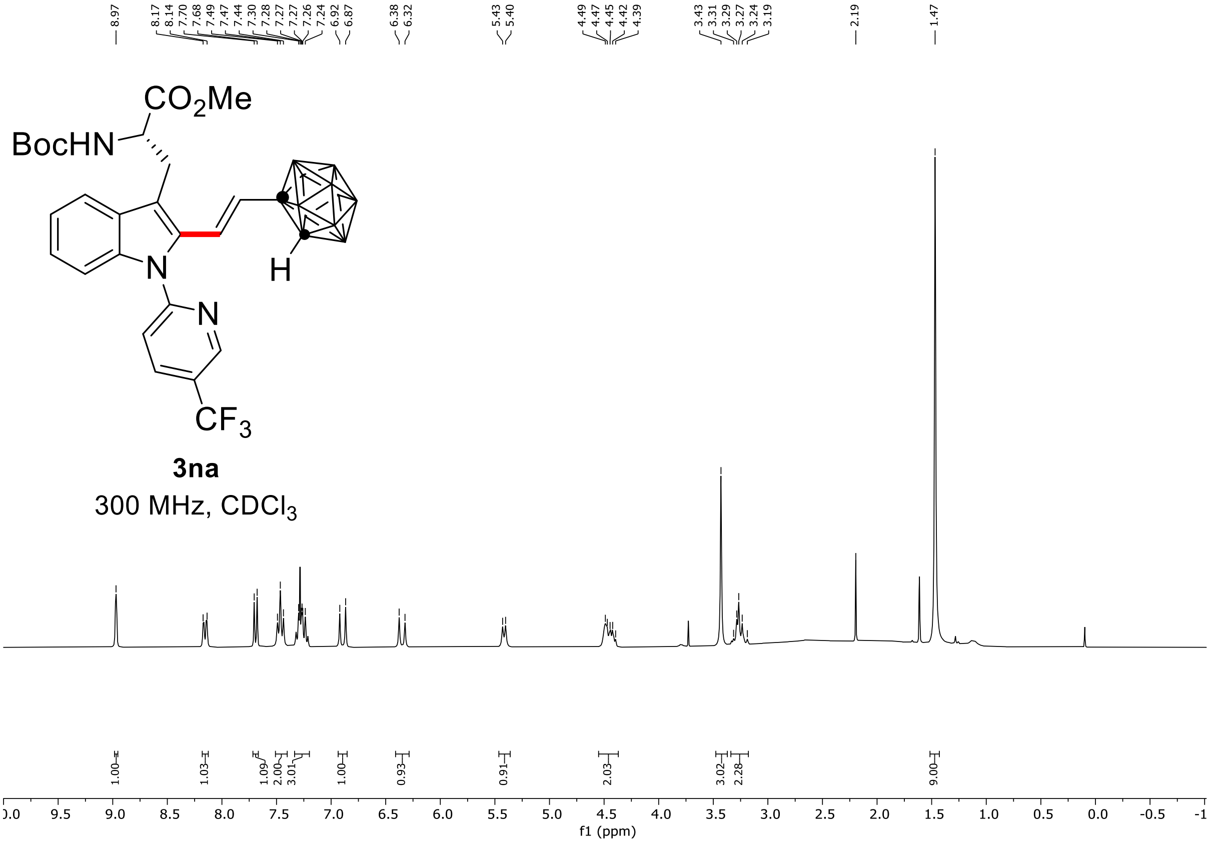

3na
300 MHz, $CDCl_3$

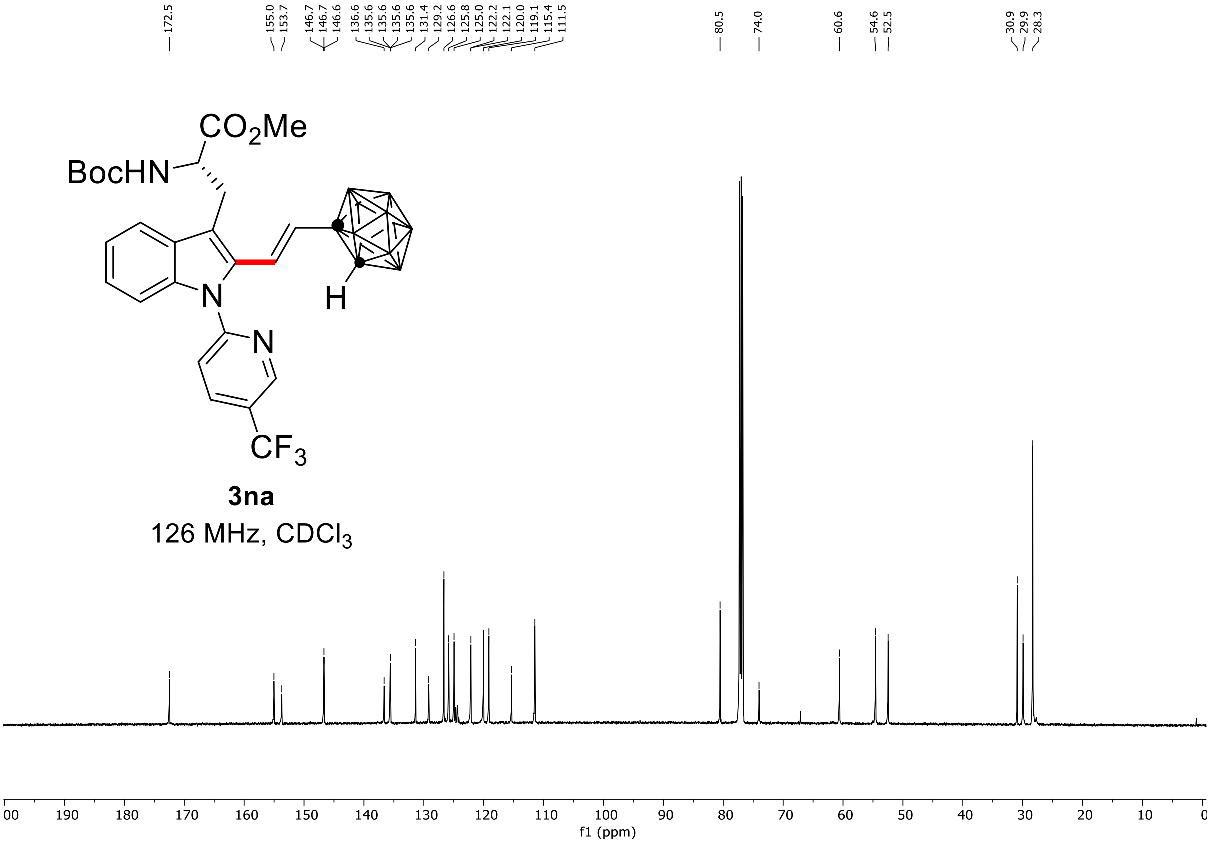
172.5
155.0
153.7
146.7
146.7
146.6
136.6
135.6
135.6
135.6
135.6
131.4
129.2
126.6
125.8
125.0
122.2
122.1
120.0
119.1
115.4
111.5
80.5
74.0
60.6
54.6
52.5
30.9
29.9
28.3
CO_2Me
BocHN
N
N
H
CF_3
3na
126 MHz, $CDCl_3$
f1 (ppm)

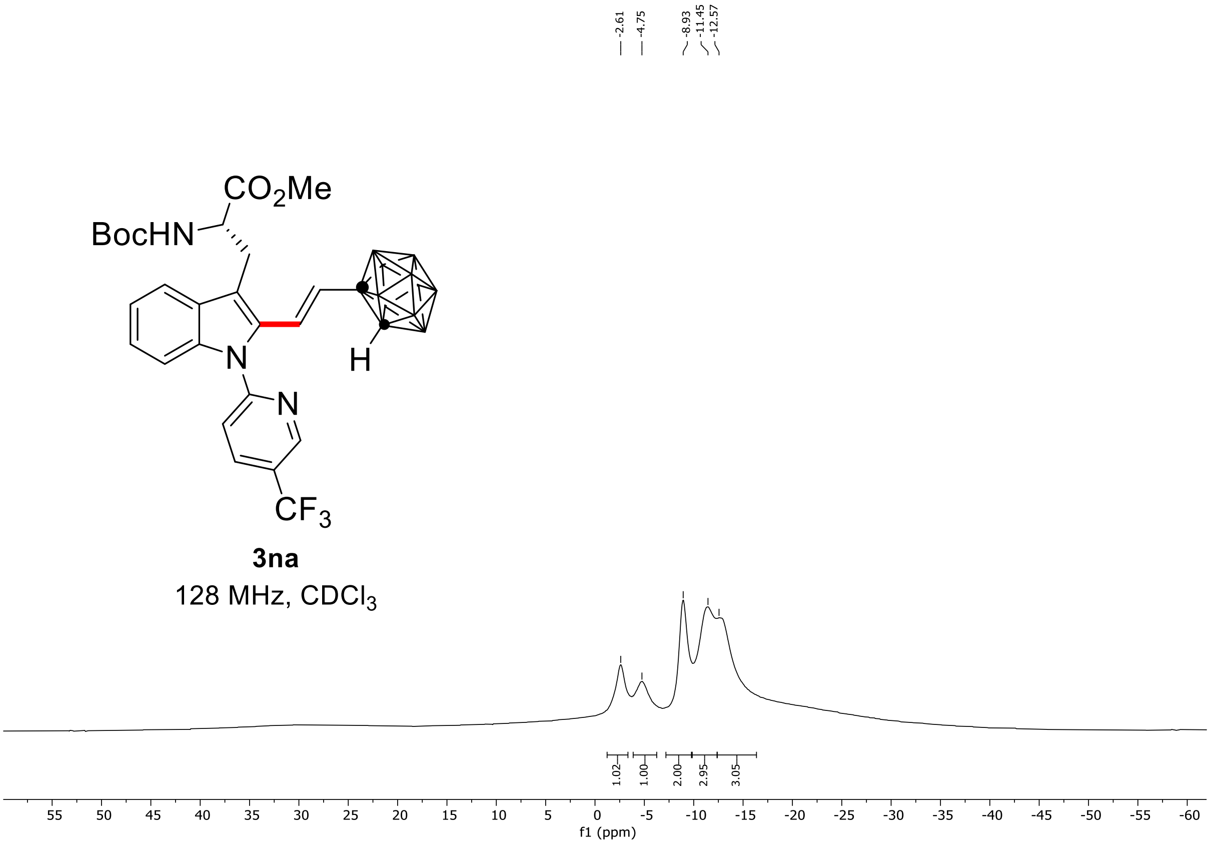
-2.61
-4.75
-8.93
-11.45
-12.57
CO_2Me
BocHN
N
N
H
CF_3
3na
128 MHz, $CDCl_3$
1.02
1.00
2.00
2.95
3.05
f1 (ppm)

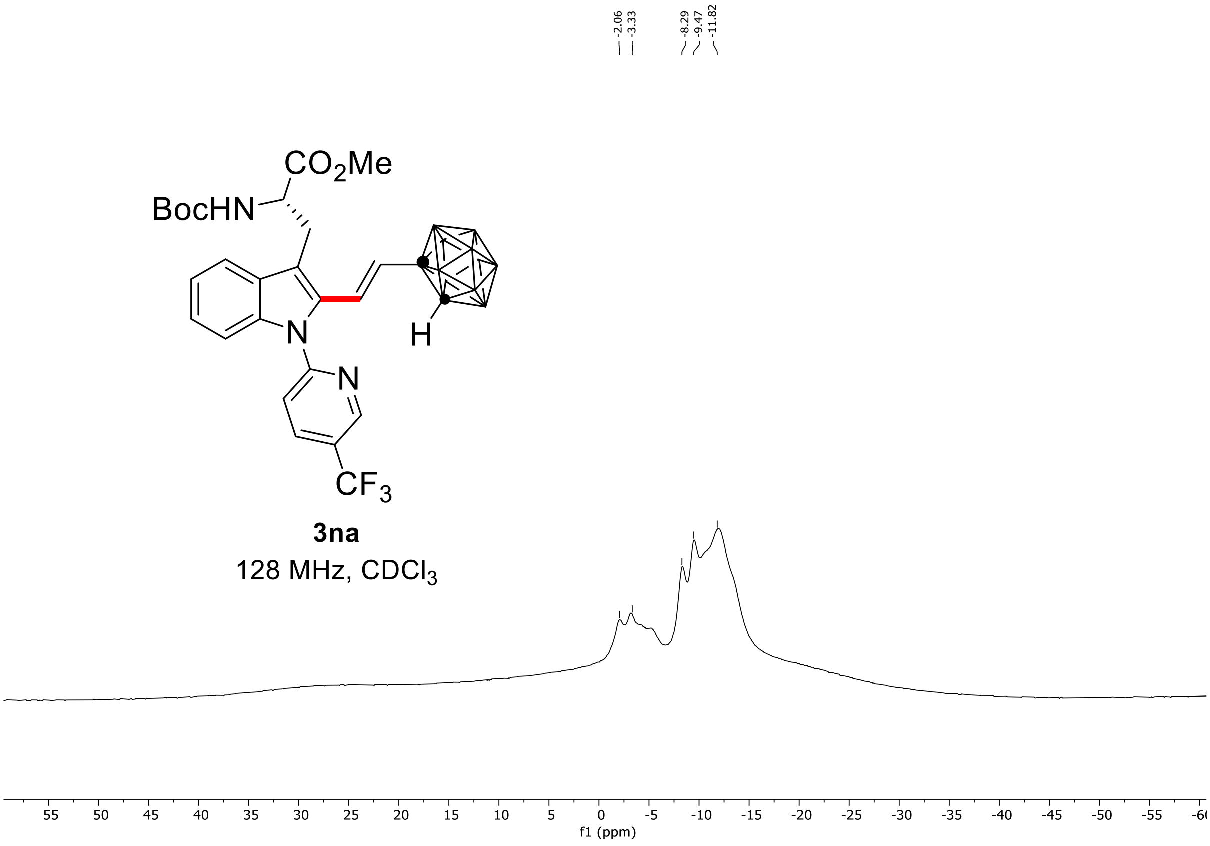
-2.06
-3.33
-8.29
-9.47
-11.82
CO2Me
BocHN
N
N
H
CF3
3na
128 MHz, CDCl3
f1 (ppm)

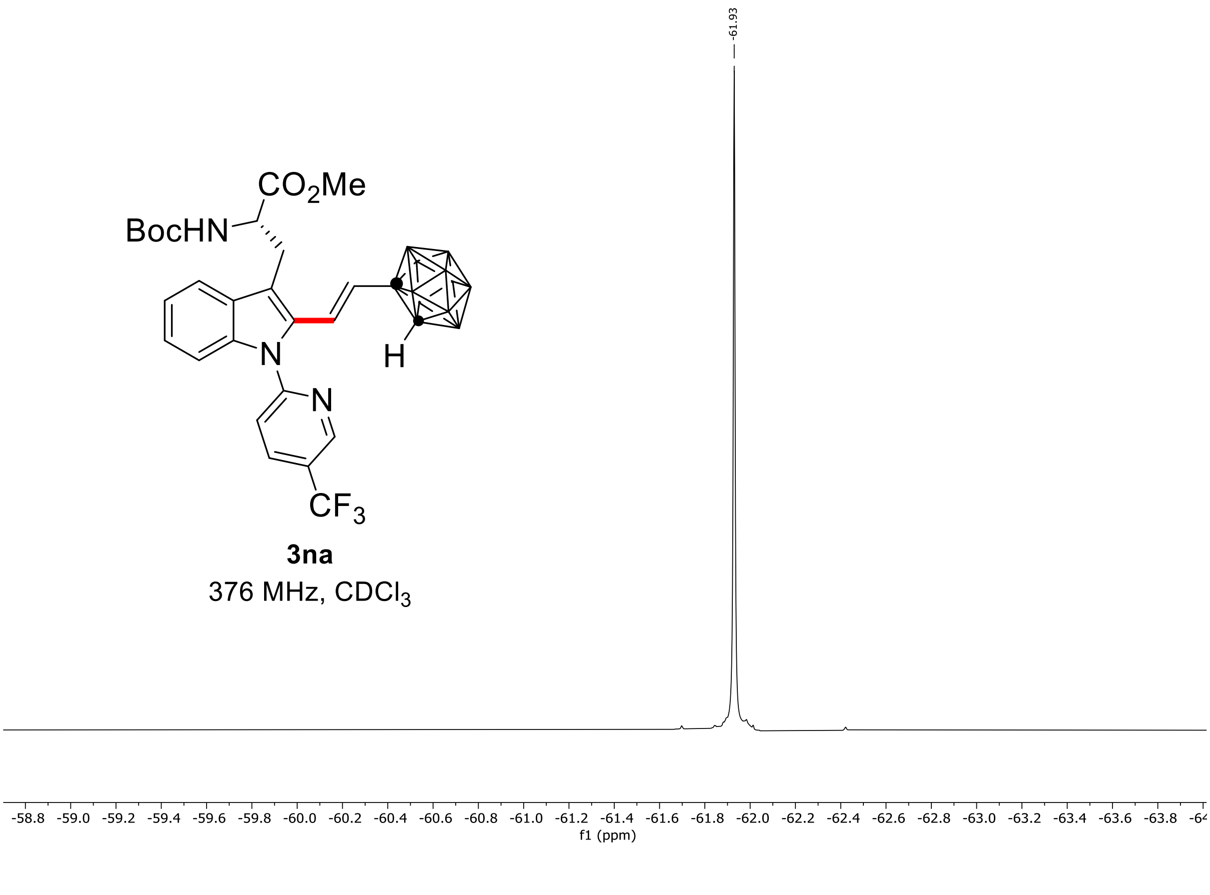
-61.93
CO2Me
BocHN
N
N
H
CF3
3na
376 MHz, CDCl3
f1 (ppm)

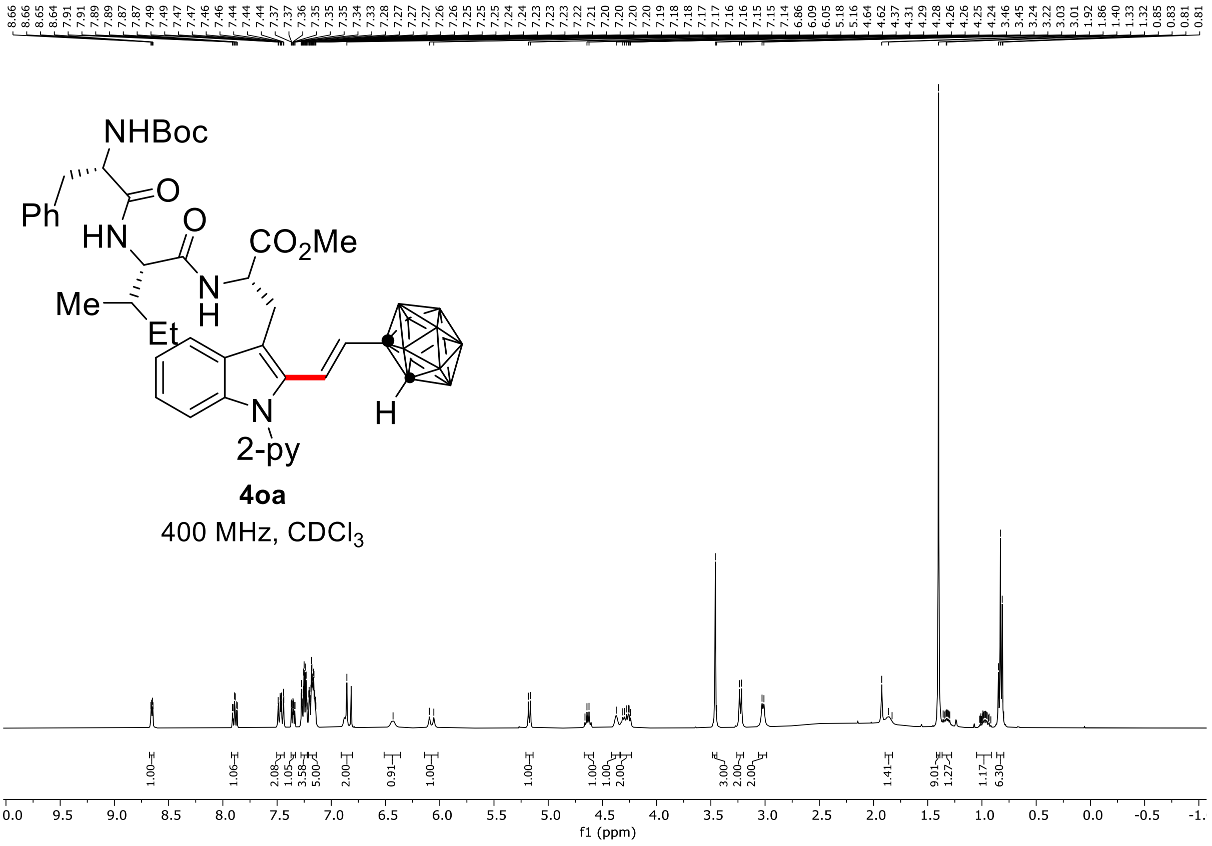
NHBoc
Ph
HN
O
O
CO2Me
Me
Et
N
H
N
2-py
H
4oa
400 MHz, CDCl3
f1 (ppm)

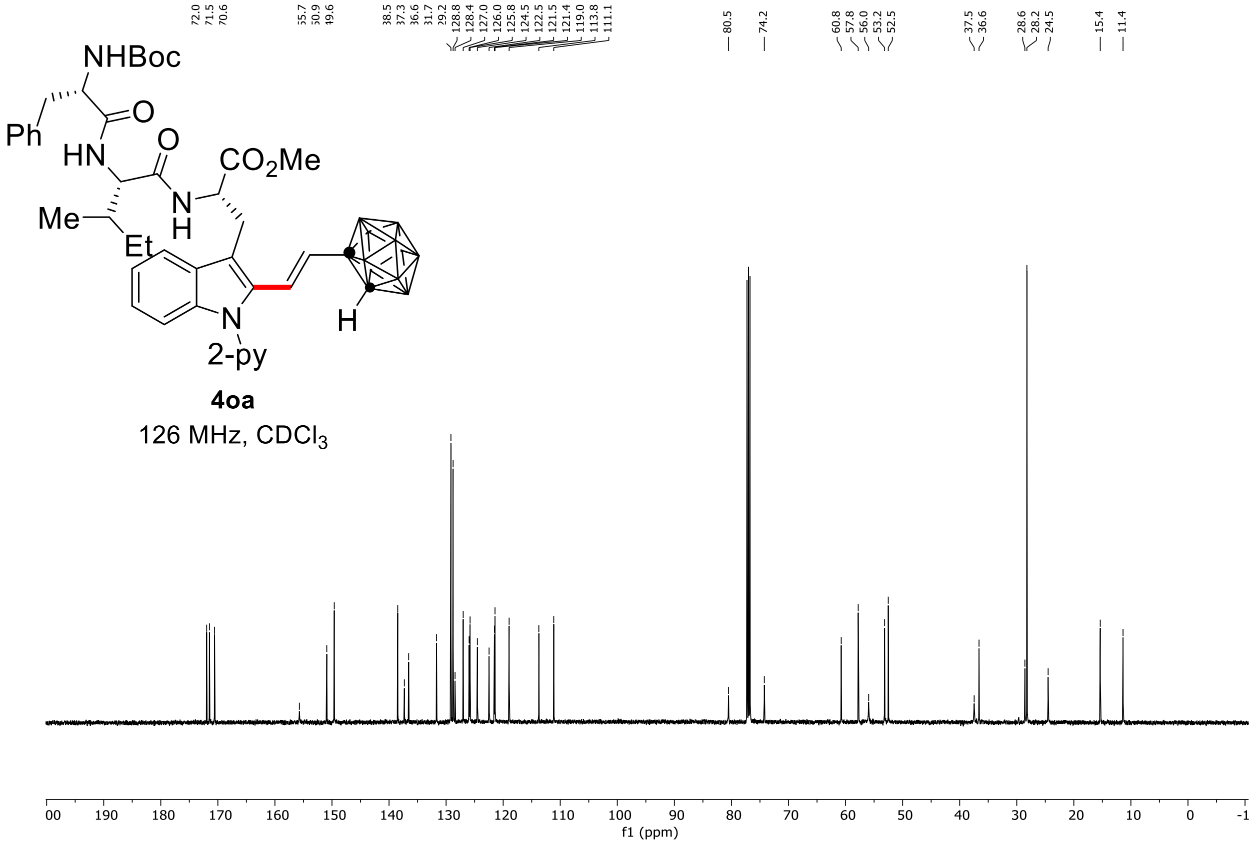
NHBoc
Ph
HN
O
O
CO2Me
Me
Et
N
H
N
2-py
H
4oa
126 MHz, CDCl3
f1 (ppm)

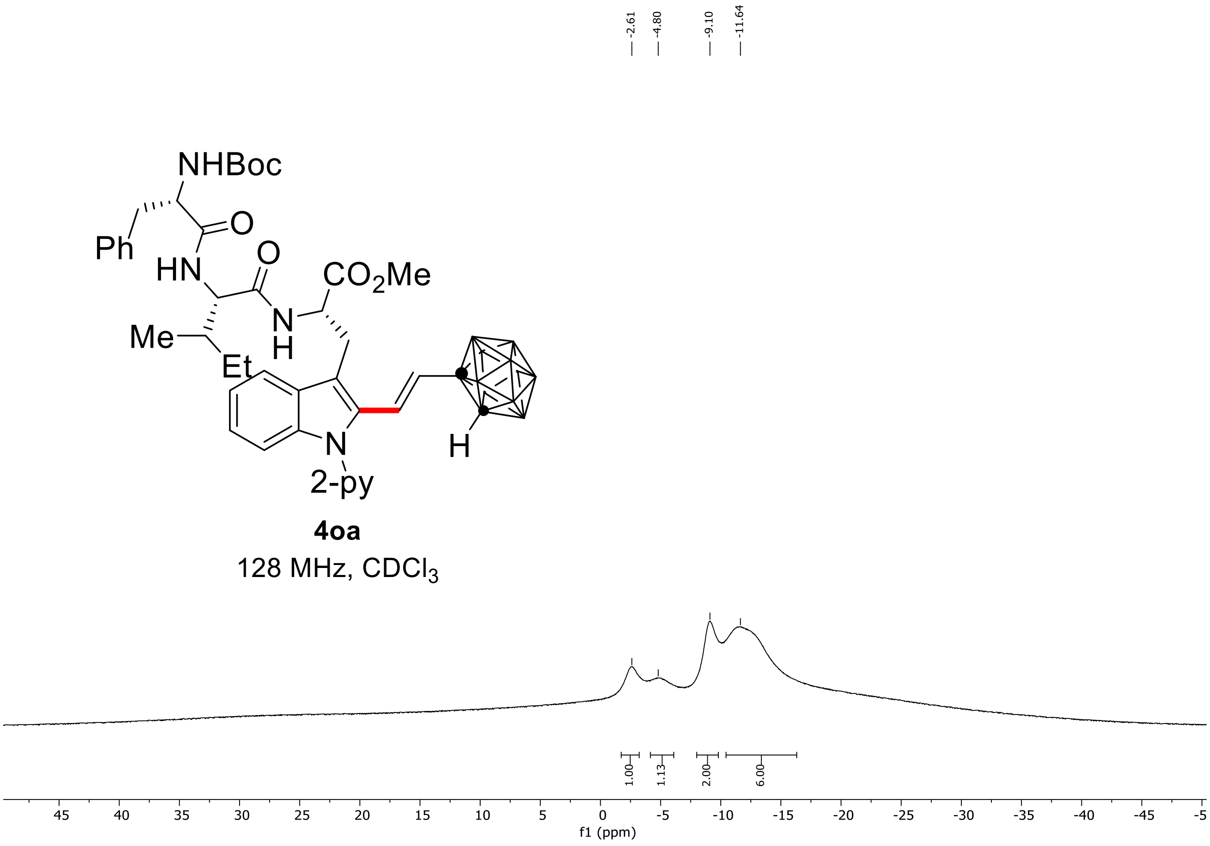
-2.61
-4.80
-9.10
-11.64
NHBoc
Ph
O
HN
O
CO2Me
N
H
Me
Et
N
2-py
H
4oa
128 MHz, CDCl3
1.00
1.13
2.00
6.00
f1 (ppm)

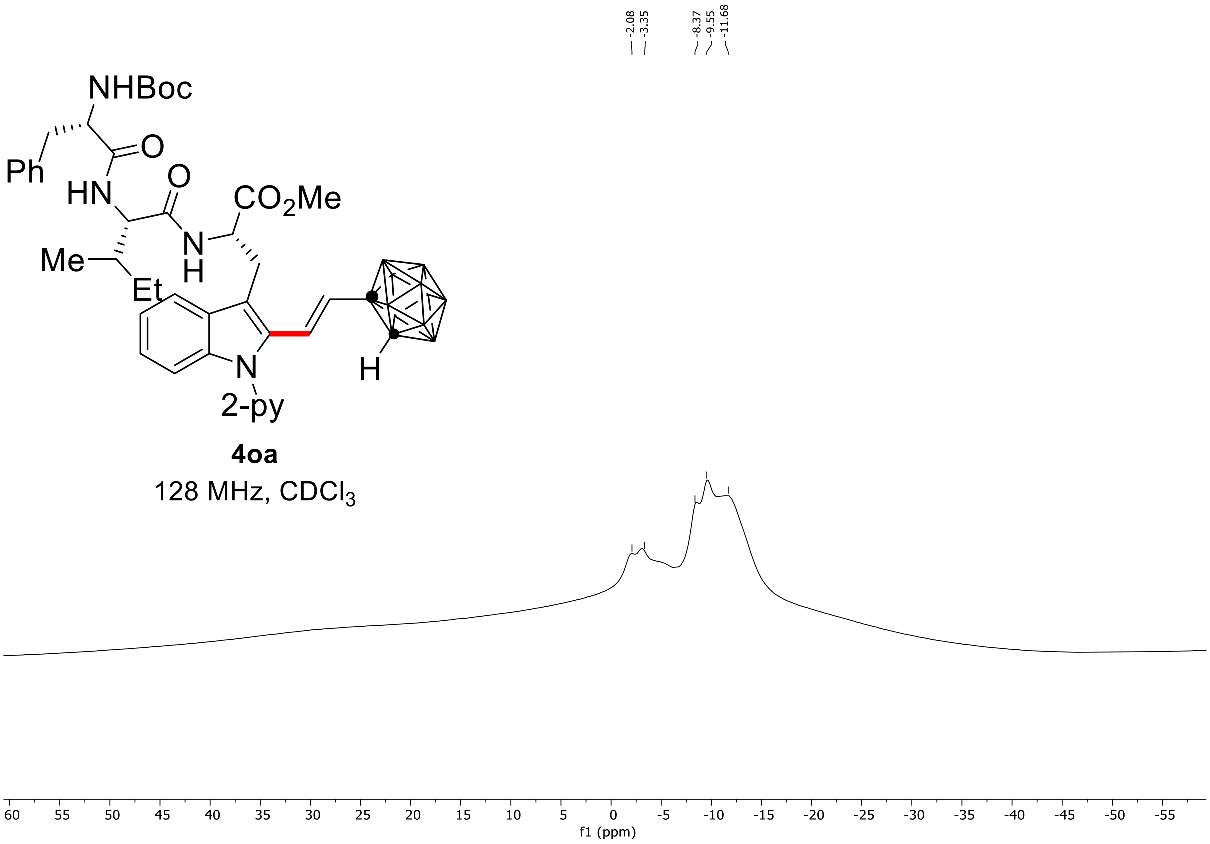
-2.08
-3.35
-8.37
-9.55
-11.68
NHBoc
Ph
O
HN
O
CO2Me
N
H
Me
Et
N
2-py
H
4oa
128 MHz, CDCl3
f1 (ppm)

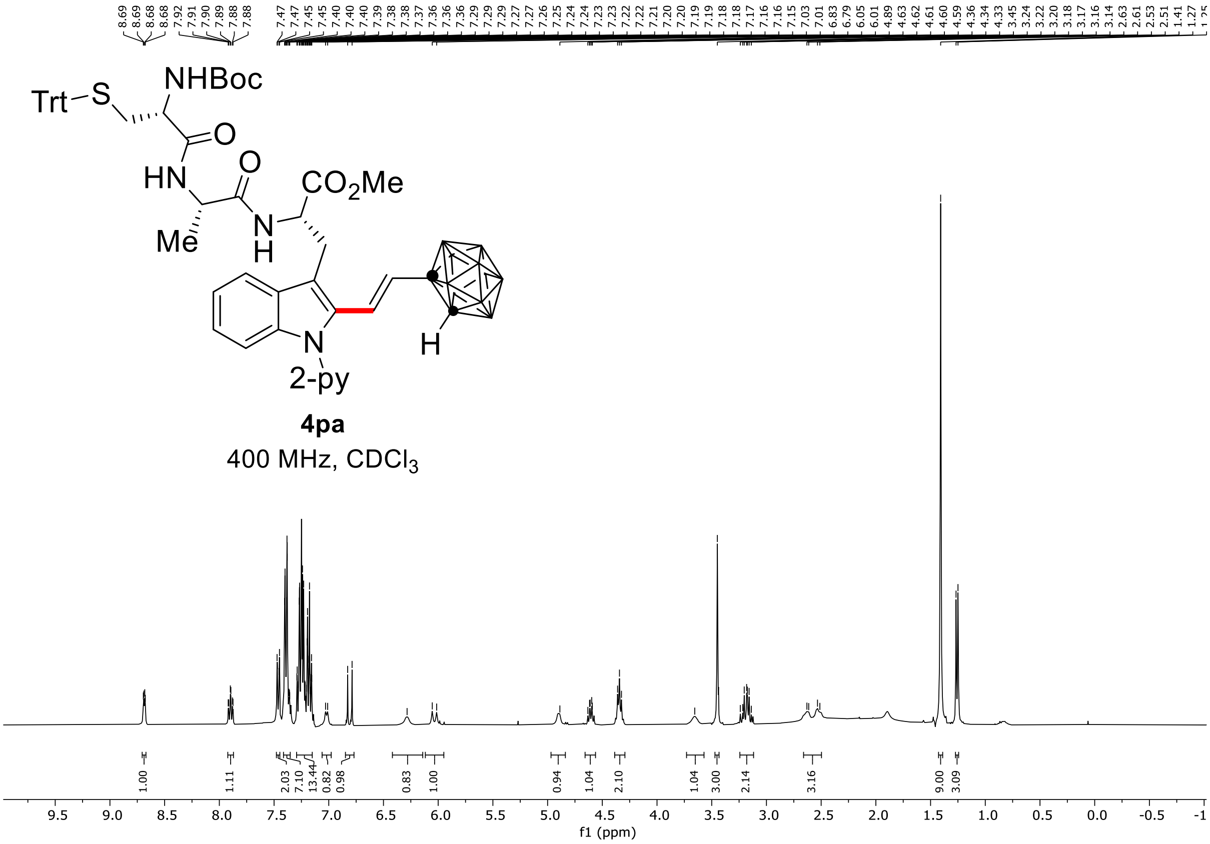
Trt
S
NHBoc
O
HN
O
CO2Me
Me
N
H
N
H
2-py
4pa
400 MHz, CDCl3
f1 (ppm)

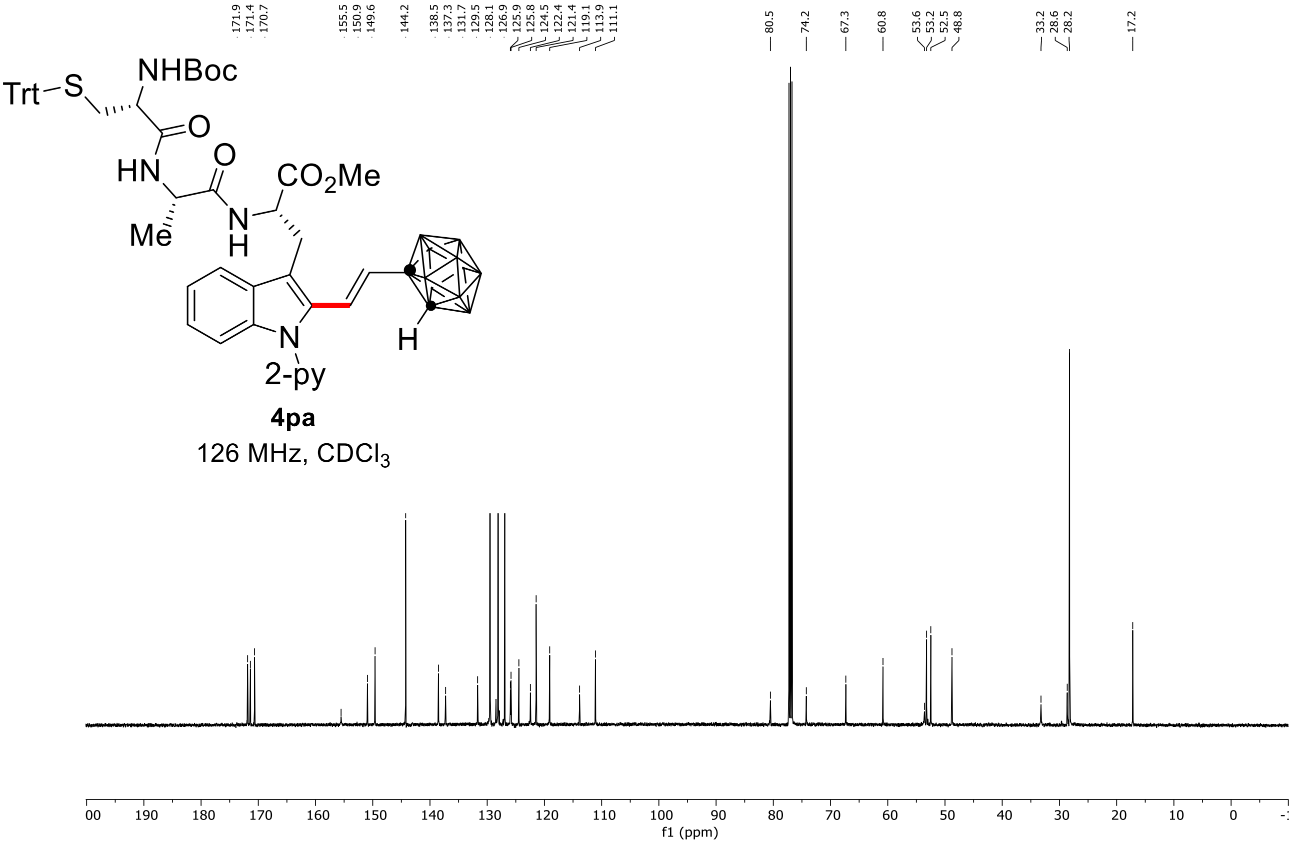
171.9
171.4
170.7
155.5
150.9
149.6
144.2
138.5
137.3
131.7
129.5
128.1
126.9
125.9
125.8
124.5
122.4
121.4
119.1
113.9
111.1
80.5
74.2
67.3
60.8
53.6
53.2
52.5
48.8
33.2
28.6
28.2
17.2
Trt
S
NHBoc
O
HN
O
CO2Me
Me
N
H
N
H
2-py
4pa
126 MHz, CDCl3
f1 (ppm)

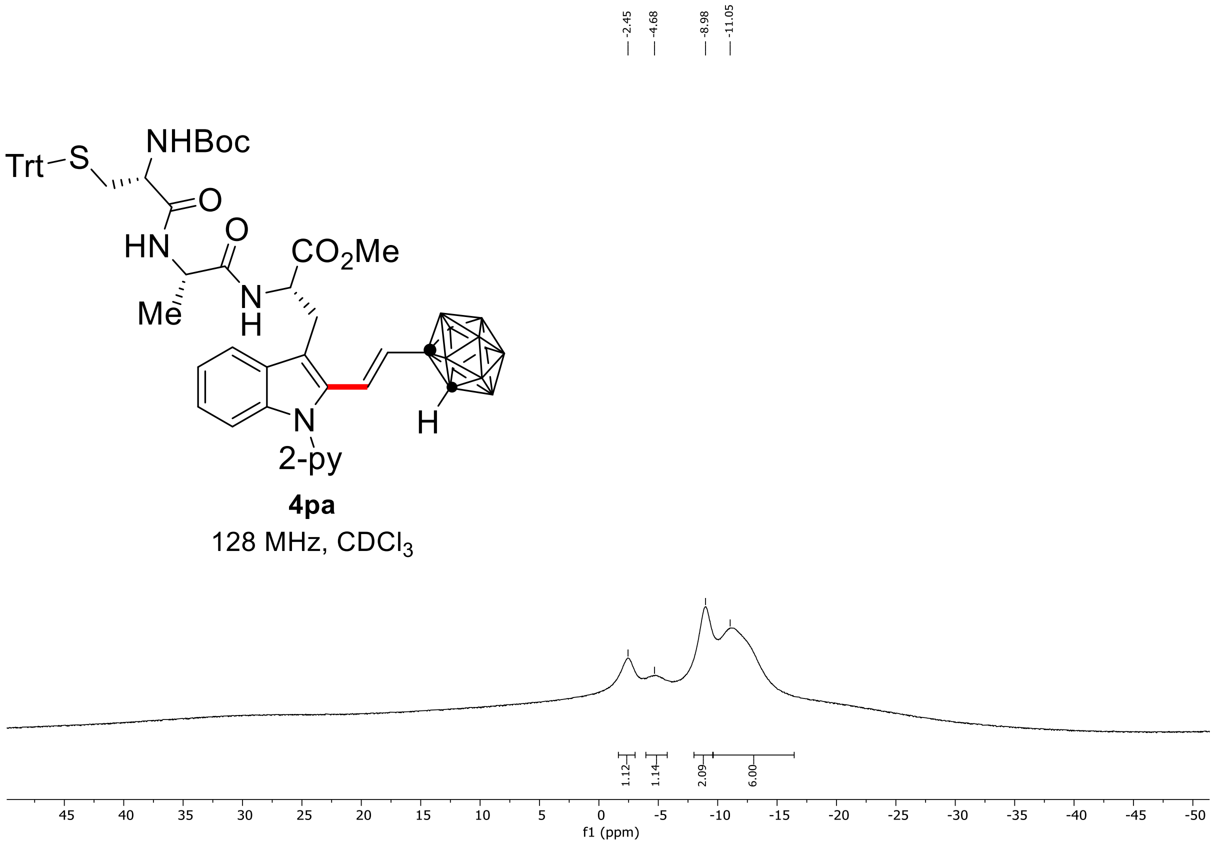

4pa

128 MHz, $CDCl_3$

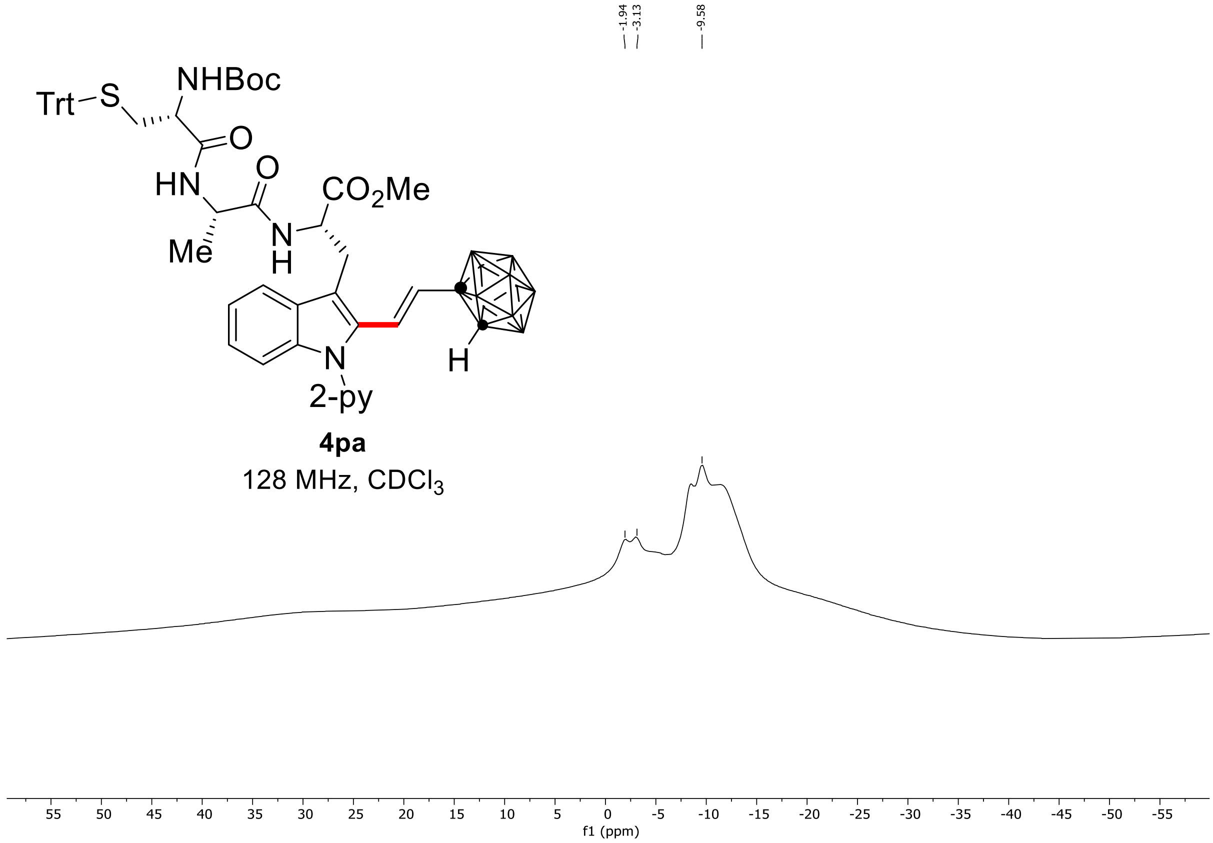

4pa

128 MHz, $CDCl_3$

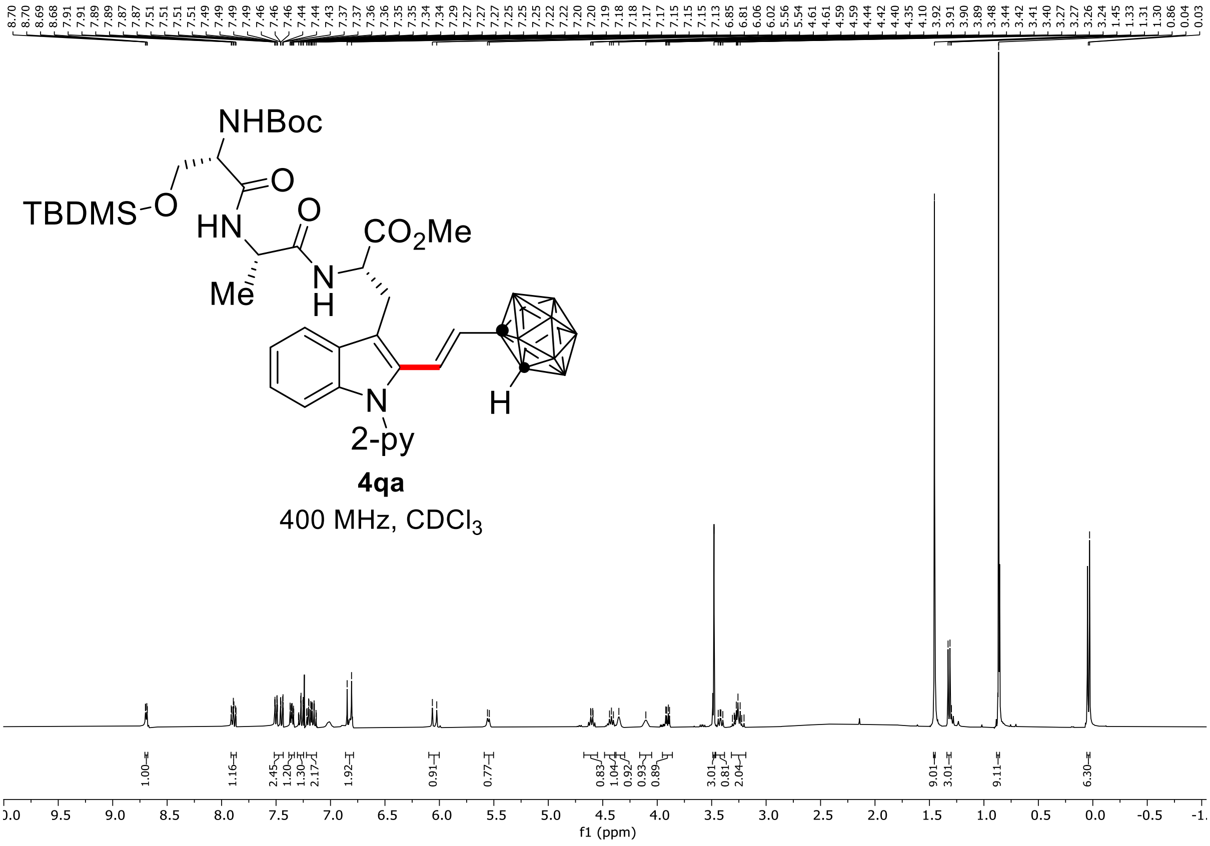

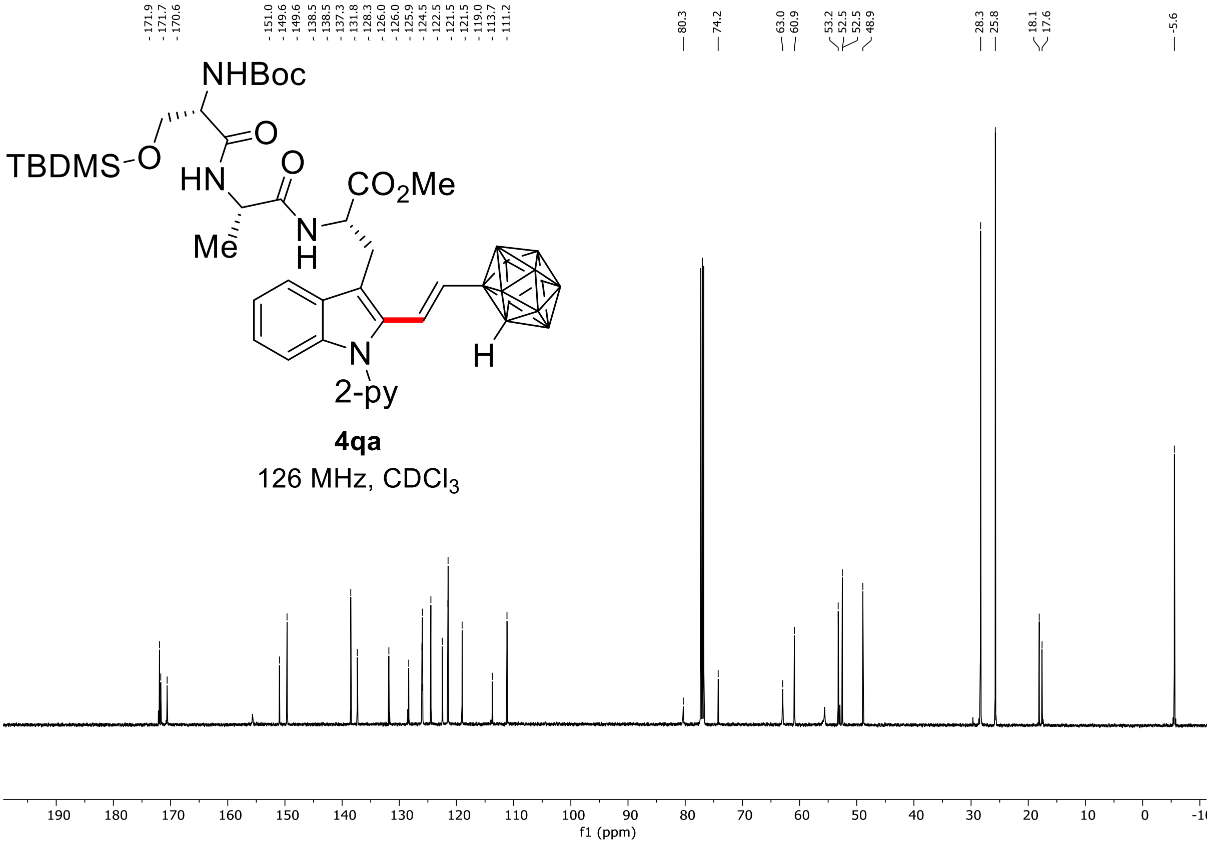

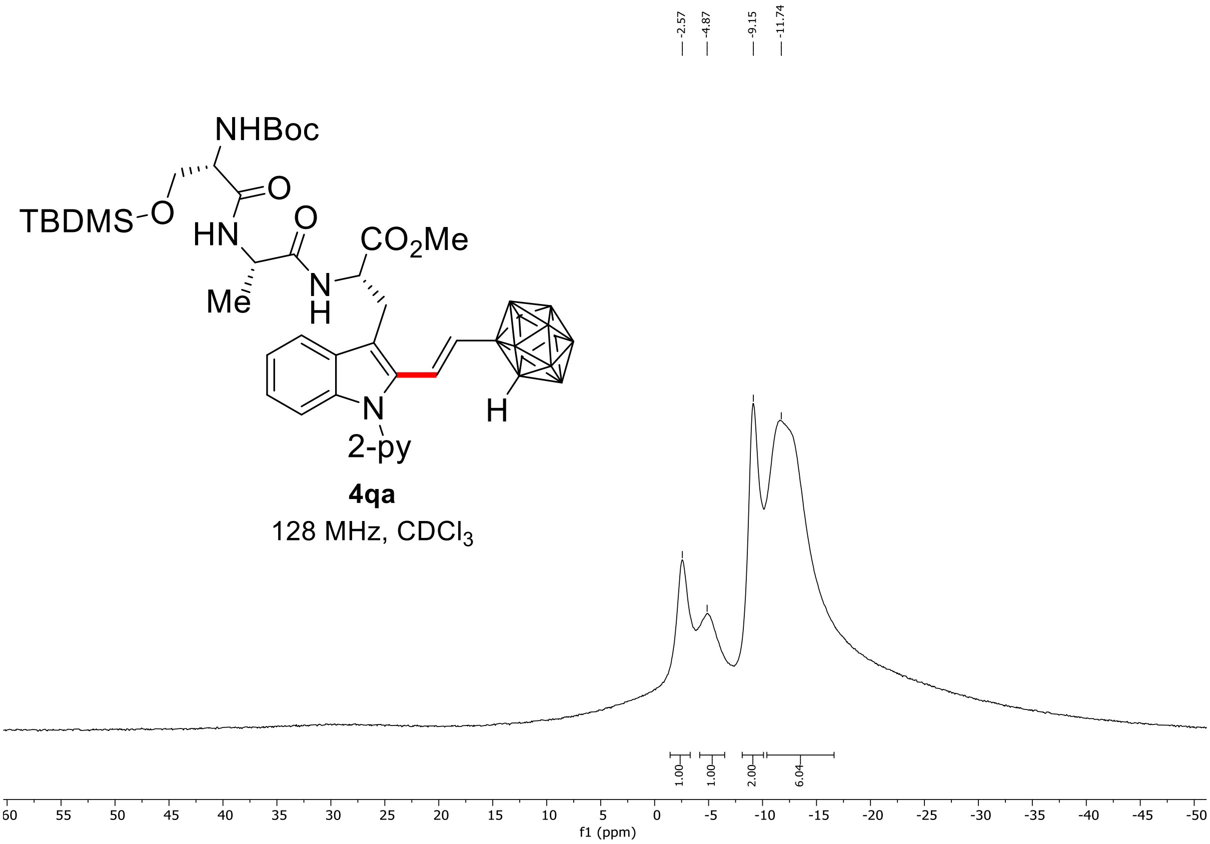

NHBoc
TBDMS
O
HN
O
O
CO2Me
N
H
Me
N
2-py
H
4qa
128 MHz, CDCl3
-2.57
-4.87
-9.15
-11.74
1.00
1.00
2.00
6.04
f1 (ppm)

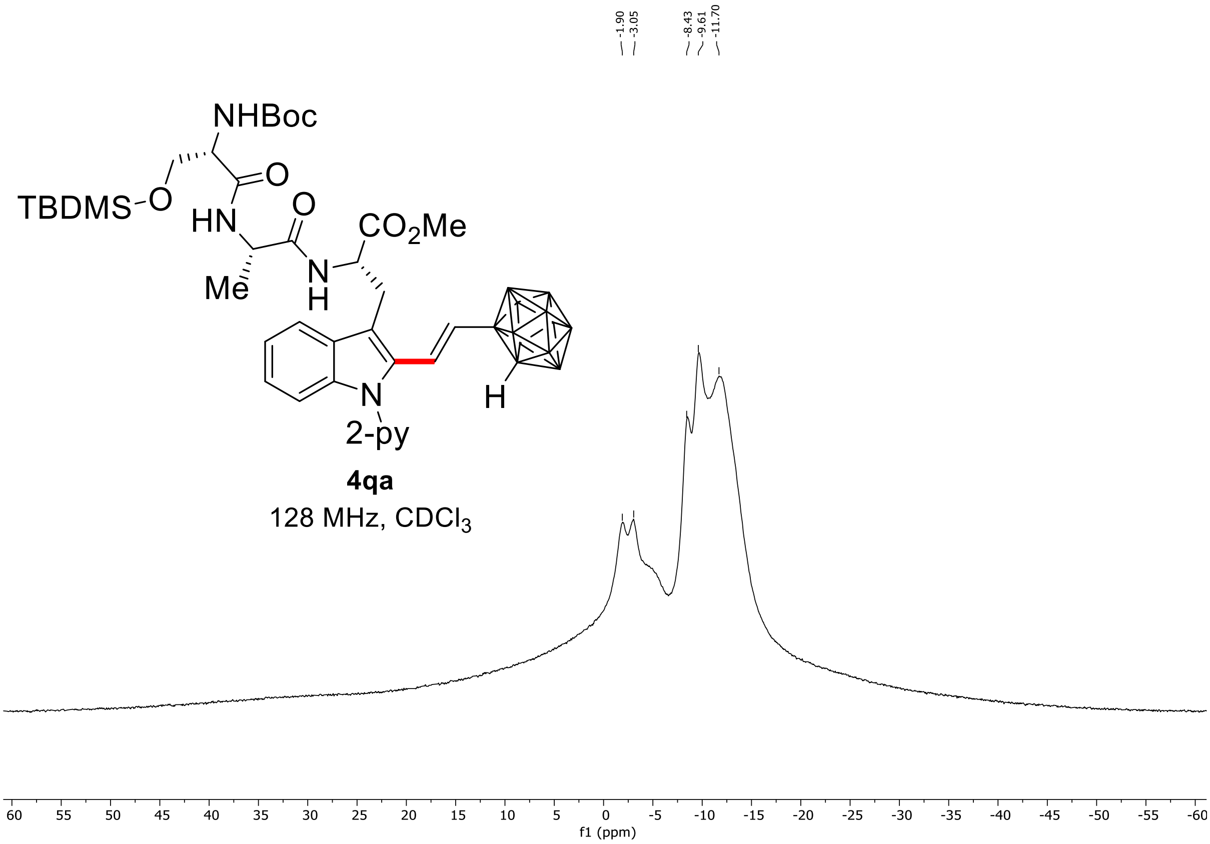

NHBoc
TBDMS
O
HN
O
O
CO2Me
N
H
Me
N
2-py
H
4qa
128 MHz, CDCl3
-1.90
-3.05
-8.43
-9.61
-11.70
f1 (ppm)

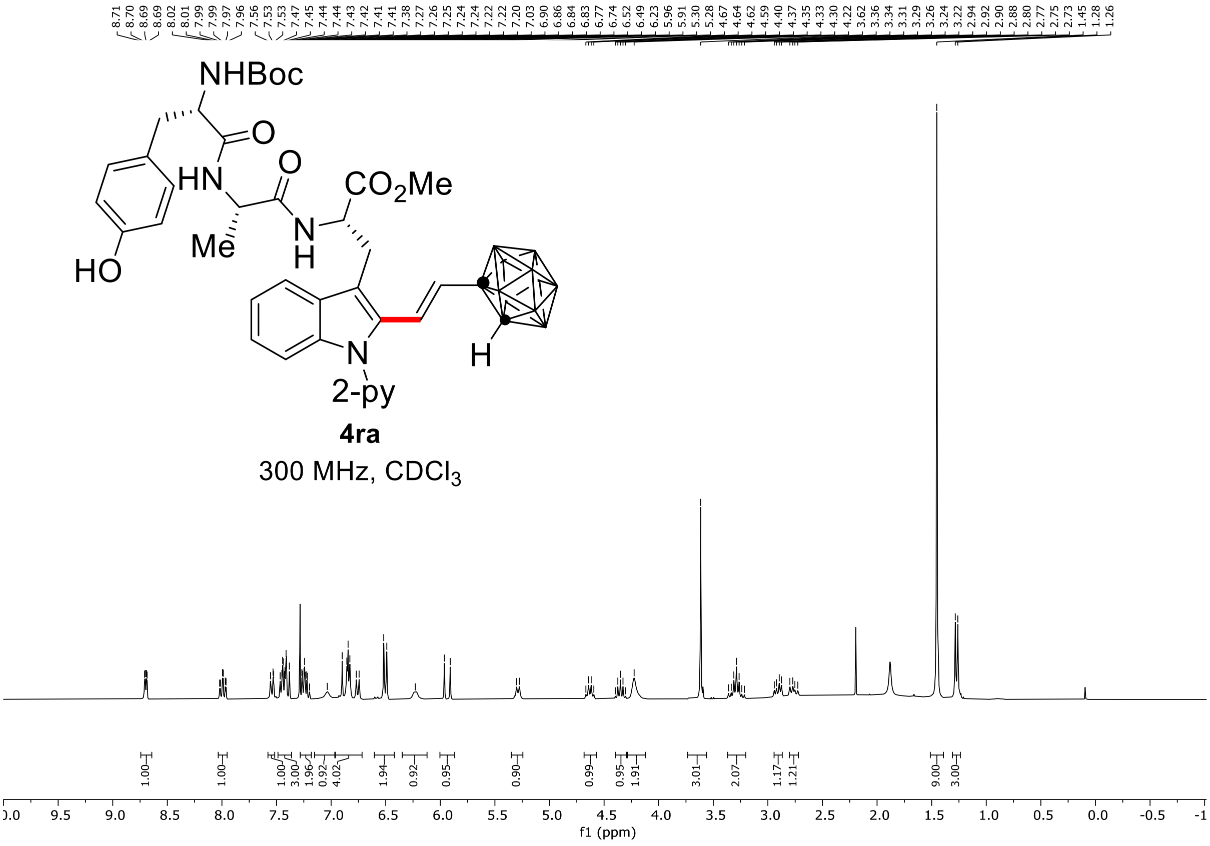
NHBoc
HN
Me
HO
CO2Me
N
H
2-py
H
4ra
300 MHz, CDCl3
f1 (ppm)

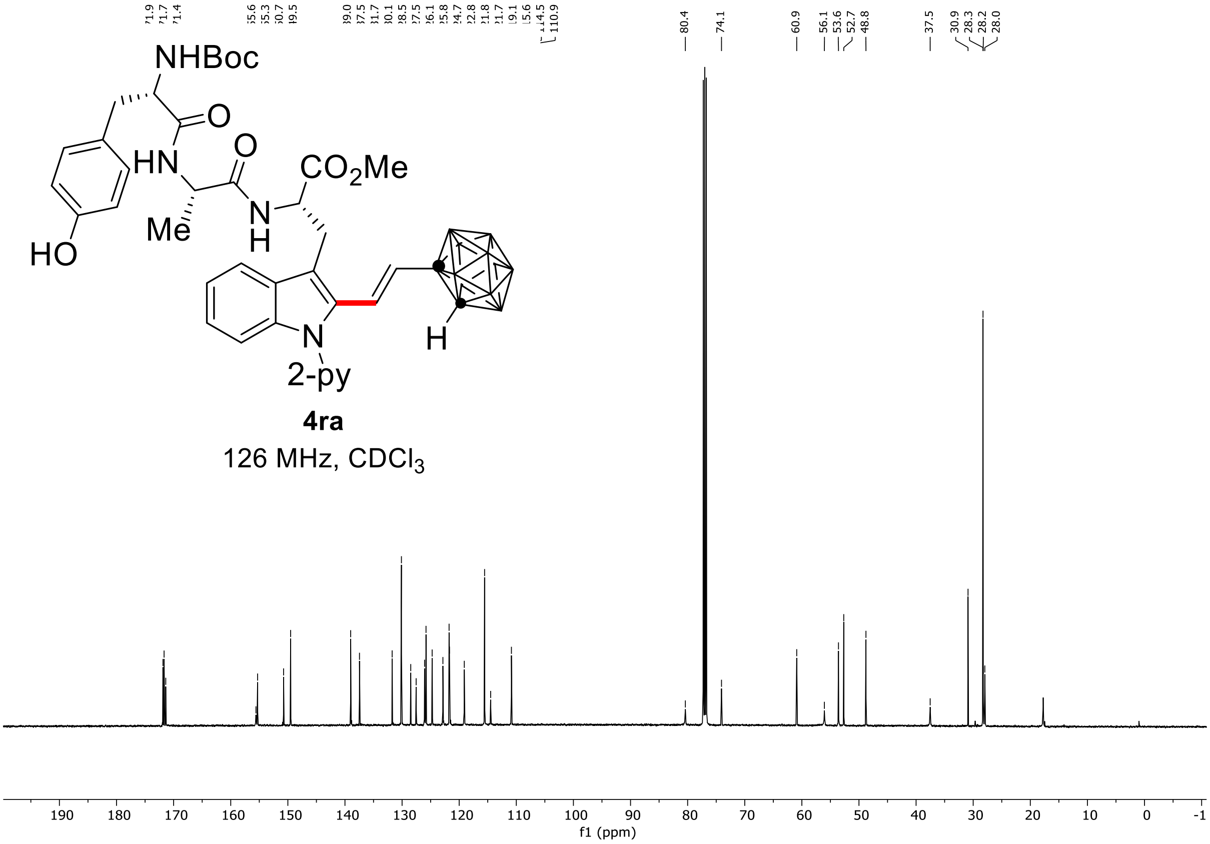
NHBoc
HN
Me
HO
CO2Me
N
H
2-py
H
4ra
126 MHz, CDCl3
f1 (ppm)

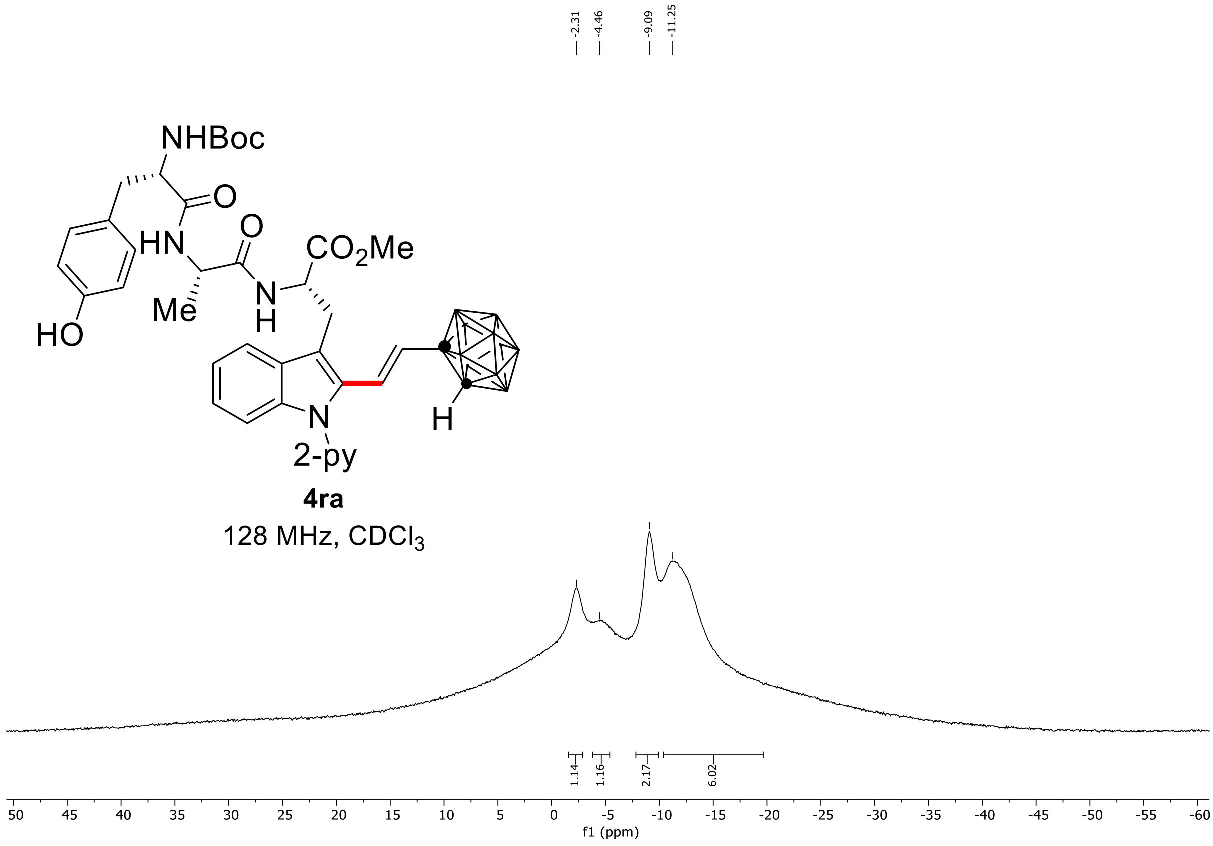
-2.31
-4.46
-9.09
-11.25
NHBoc
O
HN
O
CO2Me
N
H
Me
HO
N
2-py
H
4ra
128 MHz, CDCl3
1.14
1.16
2.17
6.02
f1 (ppm)

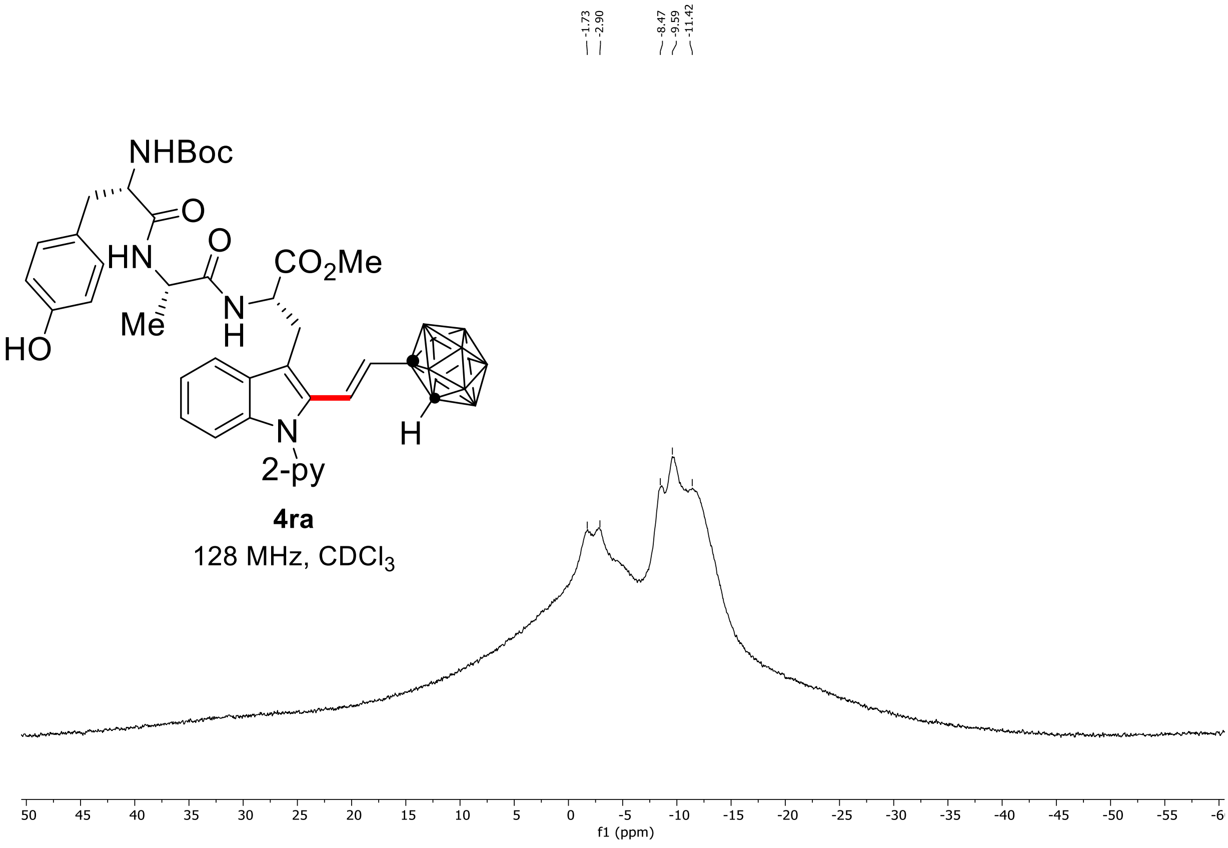
-1.73
-2.90
-8.47
-9.59
-11.42
NHBoc
O
HN
O
CO2Me
N
H
Me
HO
N
2-py
H
4ra
128 MHz, CDCl3
f1 (ppm)

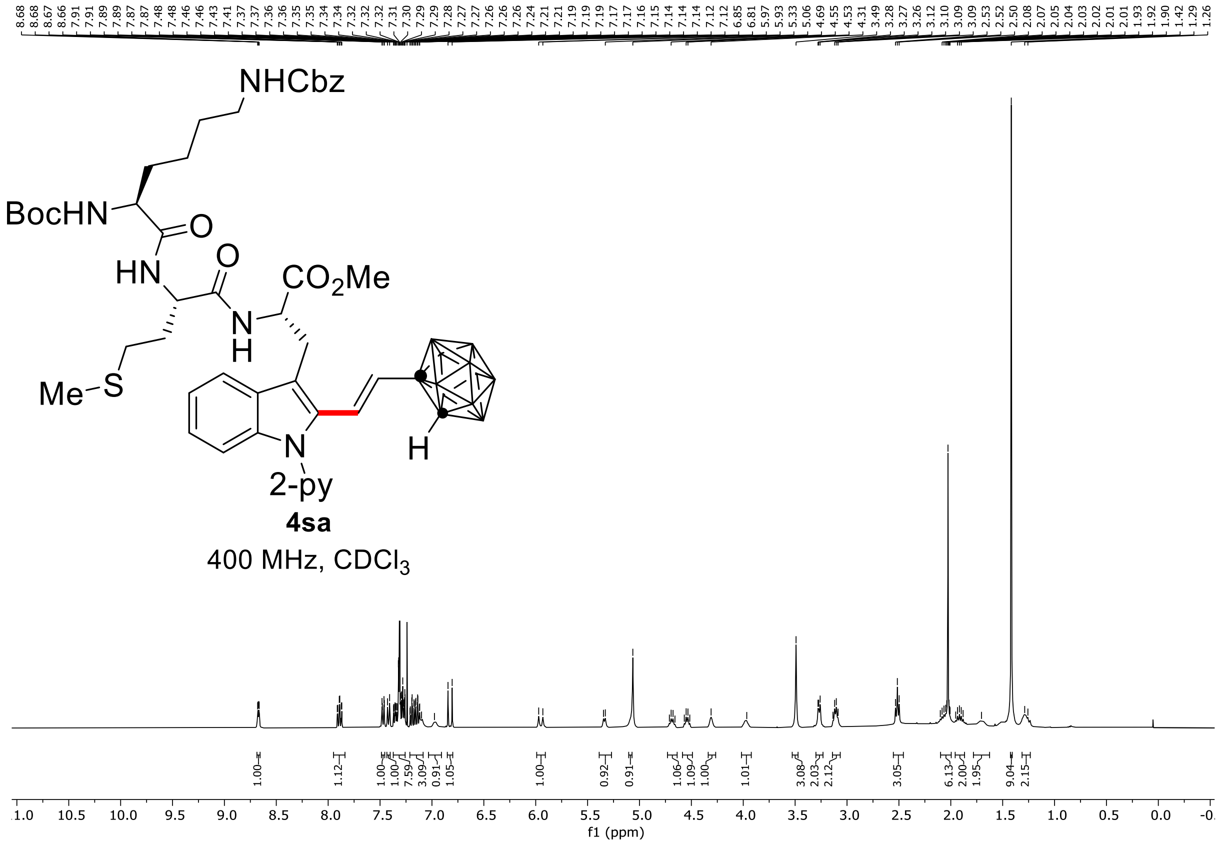
8.68 8.68 8.67 8.66 7.91 7.89 7.89 7.87 7.48 7.48 7.46 7.43 7.41 7.37 7.37 7.36 7.36 7.35 7.35 7.34 7.34 7.32 7.32 7.31 7.30 7.29 7.29 7.28 7.27 7.27 7.26 7.26 7.24 7.21 7.21 7.19 7.19 7.17 7.17 7.16 7.15 7.14 7.14 7.14 7.12 7.12 6.85 6.81 5.97 5.93 5.33 5.06 4.69 4.55 4.53 4.31 3.49 3.28 3.27 3.26 3.12 3.10 3.09 3.09 2.53 2.52 2.50 2.08 2.07 2.05 2.04 2.03 2.02 2.02 2.01 2.01 1.93 1.92 1.90 1.42 1.29 1.26
NHCbz
BocHN
O
HN
O
CO_2Me
N
H
Me–S
N
2-py
H
4sa
400 MHz, $CDCl_3$
1.00 1.12 1.00 1.00 7.59 3.09 0.91 1.05 1.00 0.92 0.91 1.06 1.09 1.00 1.01 3.08 2.03 2.12 3.05 6.13 2.00 1.95 9.04 2.15
f1 (ppm)

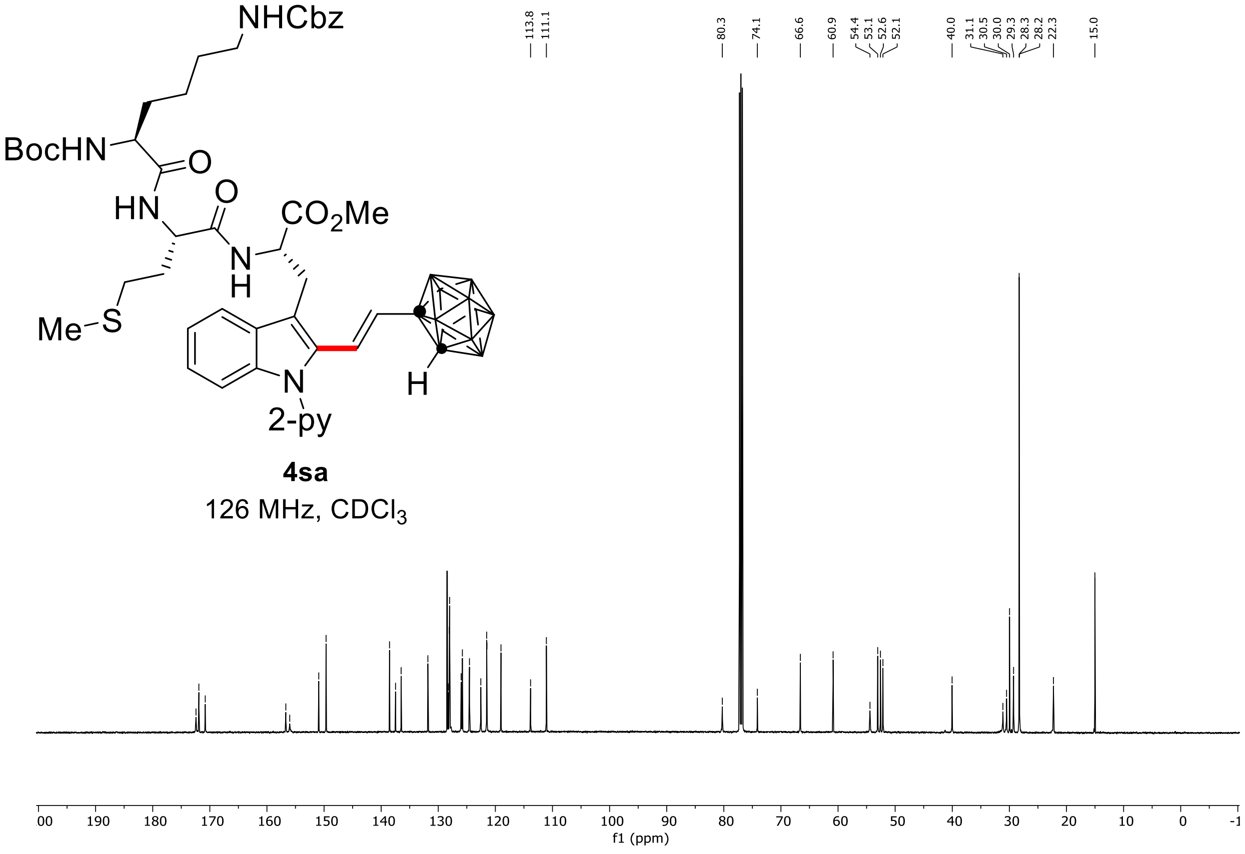
113.8 111.1 80.3 74.1 66.6 60.9 54.4 53.1 52.6 52.1 40.0 31.1 30.5 30.0 29.3 28.3 28.2 22.3 15.0
NHCbz
BocHN
O
HN
O
CO_2Me
N
H
Me–S
N
2-py
H
4sa
126 MHz, $CDCl_3$
f1 (ppm)

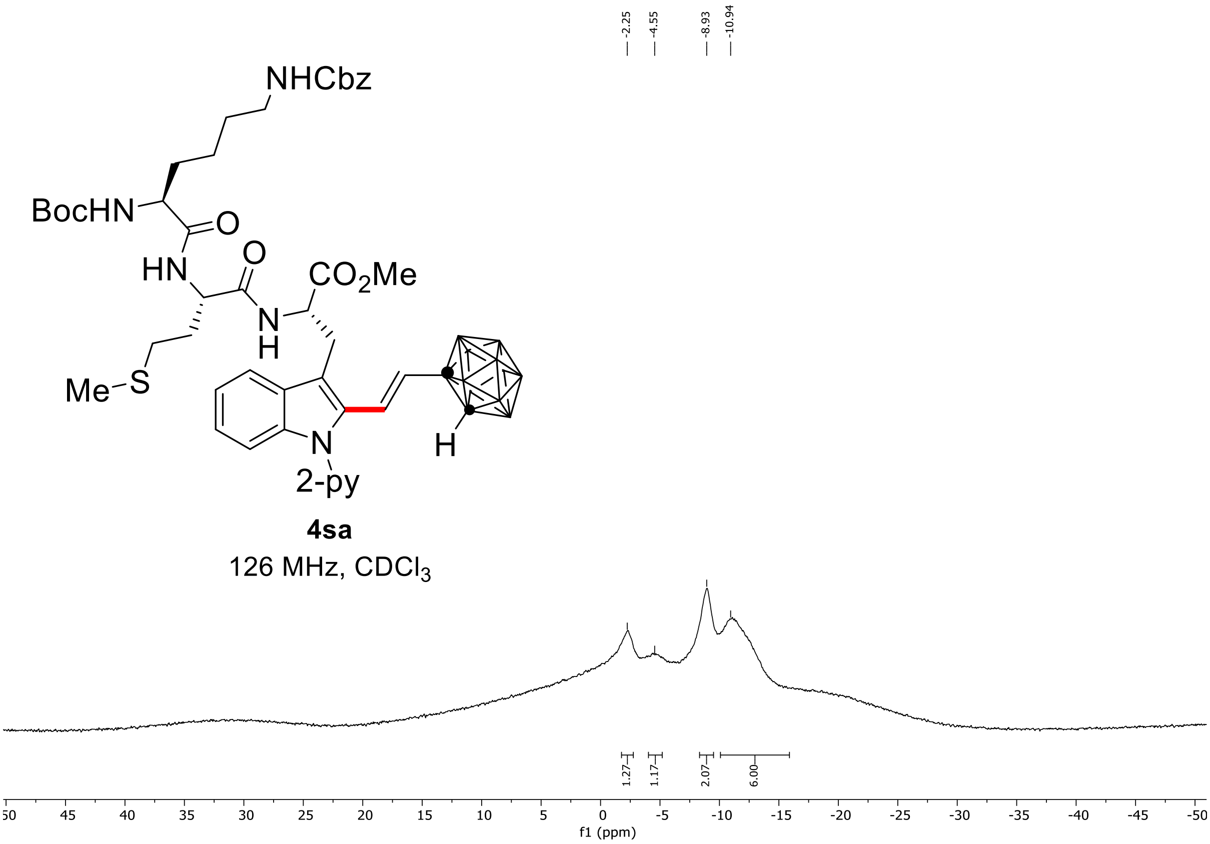
-2.25
-4.55
-8.93
-10.94
NHCbz
BocHN
O
HN
O
CO2Me
N
H
Me-S
N
2-py
H
4sa
126 MHz, CDCl3
1.27
1.17
2.07
6.00
50 45 40 35 30 25 20 15 10 5 0 -5 -10 -15 -20 -25 -30 -35 -40 -45 -50
f1 (ppm)

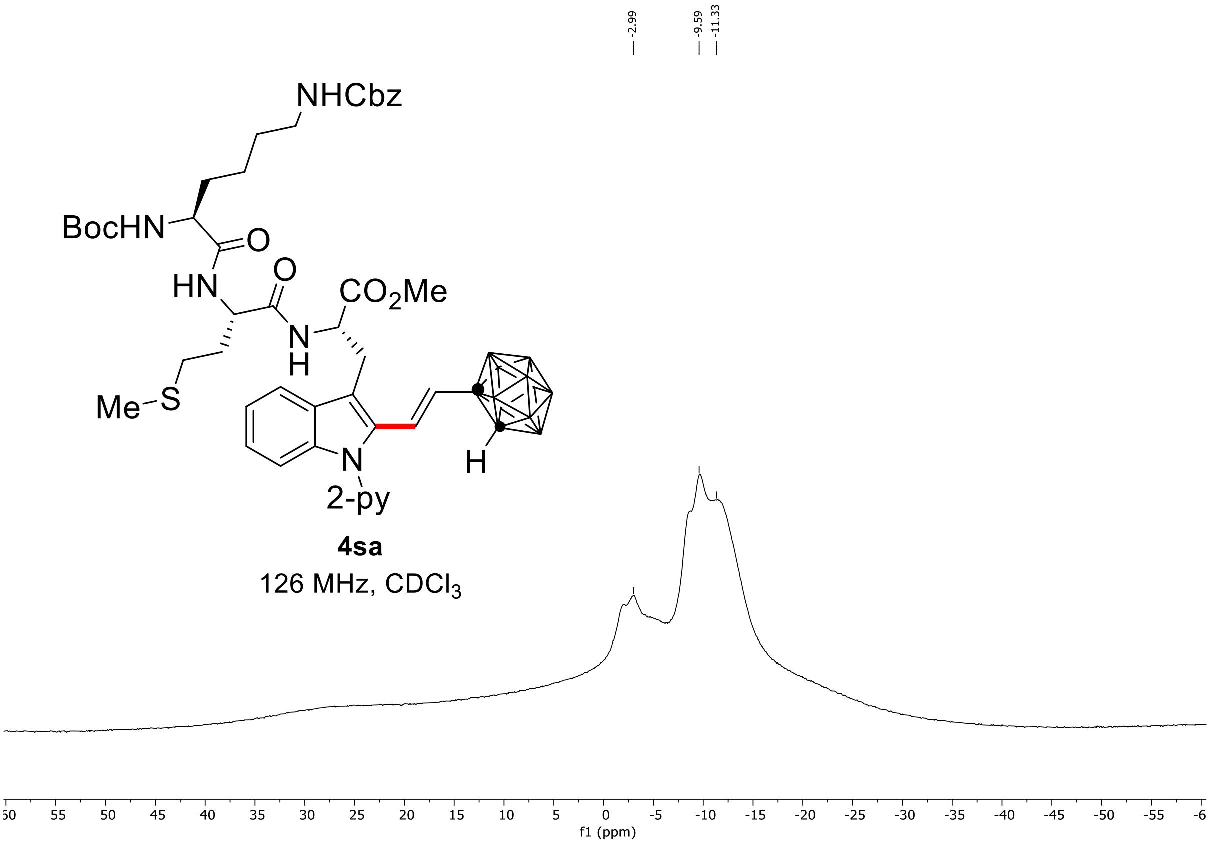
-2.99
-9.59
-11.33
NHCbz
BocHN
O
HN
O
CO2Me
N
H
Me-S
N
2-py
H
4sa
126 MHz, CDCl3
60 55 50 45 40 35 30 25 20 15 10 5 0 -5 -10 -15 -20 -25 -30 -35 -40 -45 -50 -55 -6
f1 (ppm)

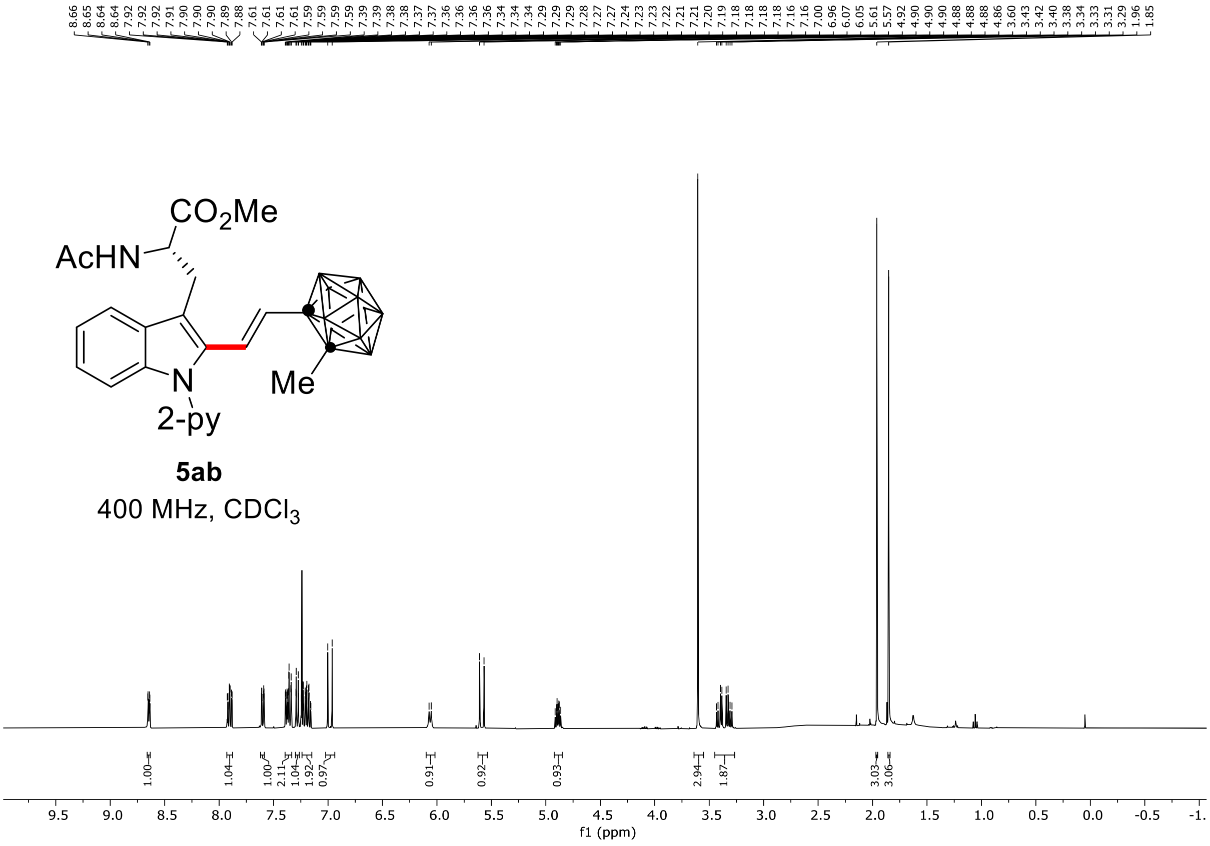
8.66 8.65 8.64 8.64 7.92 7.92 7.92 7.91 7.90 7.90 7.90 7.89 7.88 7.61 7.61 7.61 7.61 7.59 7.59 7.59 7.59 7.39 7.39 7.38 7.38 7.37 7.37 7.36 7.36 7.36 7.36 7.34 7.34 7.34 7.29 7.29 7.29 7.28 7.27 7.27 7.24 7.23 7.23 7.22 7.21 7.21 7.20 7.19 7.18 7.18 7.18 7.16 7.16 7.00 6.96 6.07 6.05 5.61 5.57 4.92 4.90 4.90 4.90 4.88 4.88 4.86 3.60 3.43 3.42 3.40 3.38 3.34 3.33 3.31 3.29 1.96 1.85
CO2Me
AcHN
N
2-py
Me
5ab
400 MHz, CDCl3
1.00 1.04 1.00 2.11 1.04 1.92 0.97 0.91 0.92 0.93 2.94 1.87 3.03 3.06
f1 (ppm)

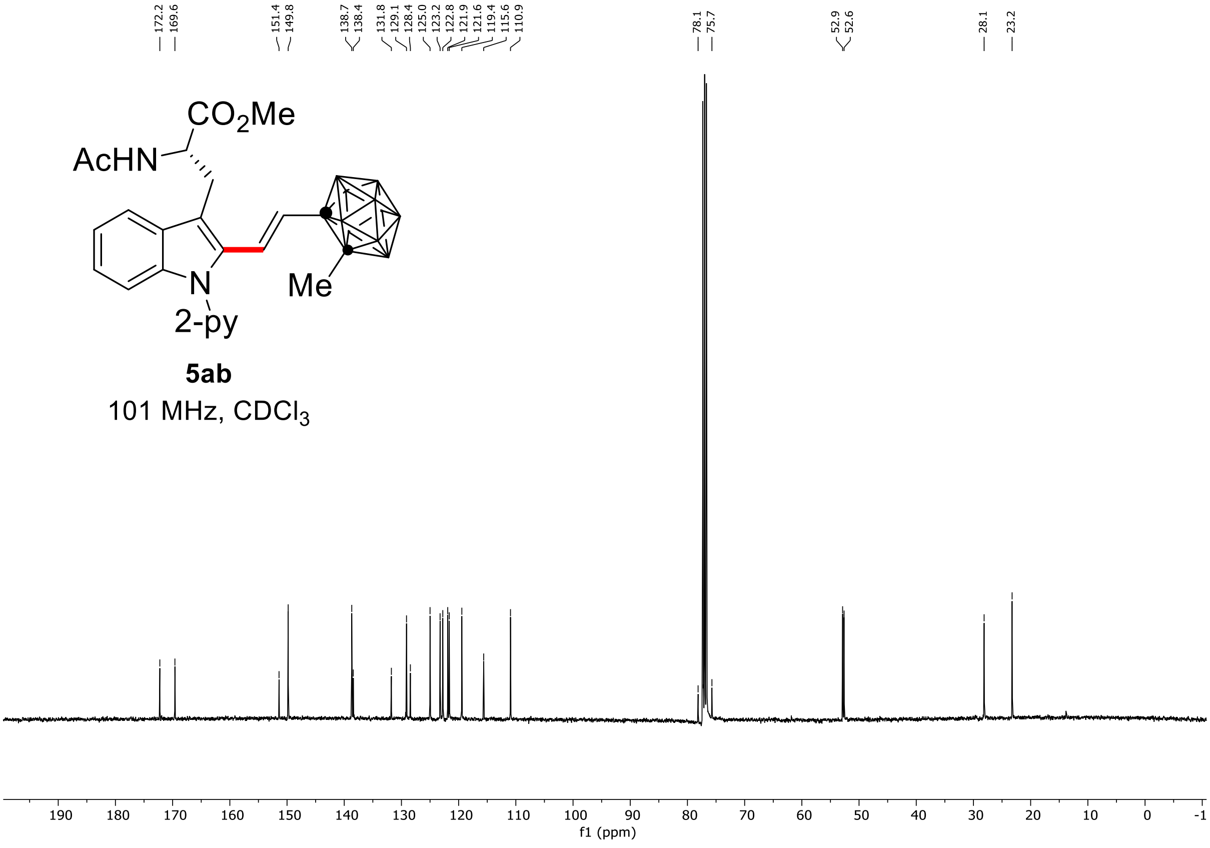
172.2 169.6 151.4 149.8 138.7 138.4 131.8 129.1 128.4 125.0 123.2 122.8 121.9 121.6 119.4 115.6 110.9 78.1 75.7 52.9 52.6 28.1 23.2
CO2Me
AcHN
N
2-py
Me
5ab
101 MHz, CDCl3
f1 (ppm)

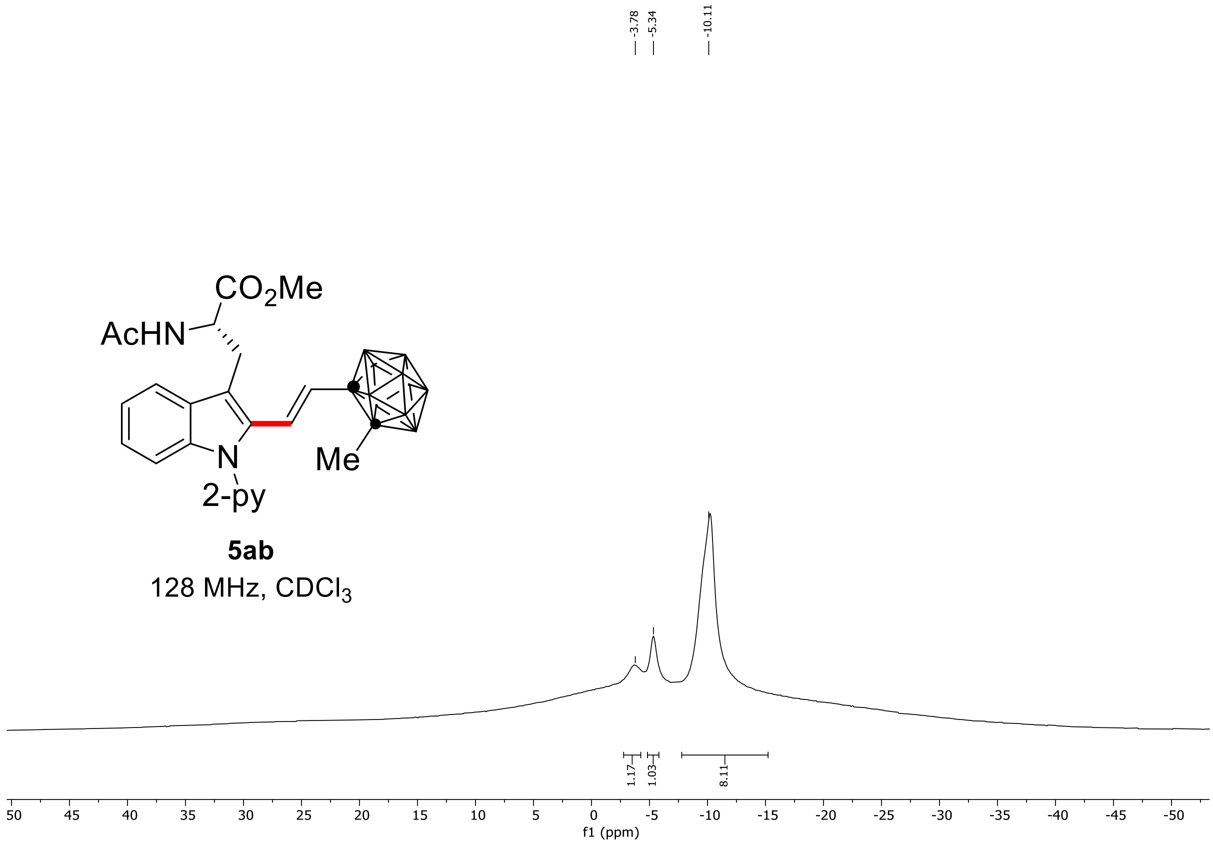

-3.78
-5.34
-10.11
CO2Me
AcHN
N
2-py
Me
5ab
128 MHz, CDCl3
1.17
1.03
8.11
50 45 40 35 30 25 20 15 10 5 0 -5 -10 -15 -20 -25 -30 -35 -40 -45 -50
f1 (ppm)

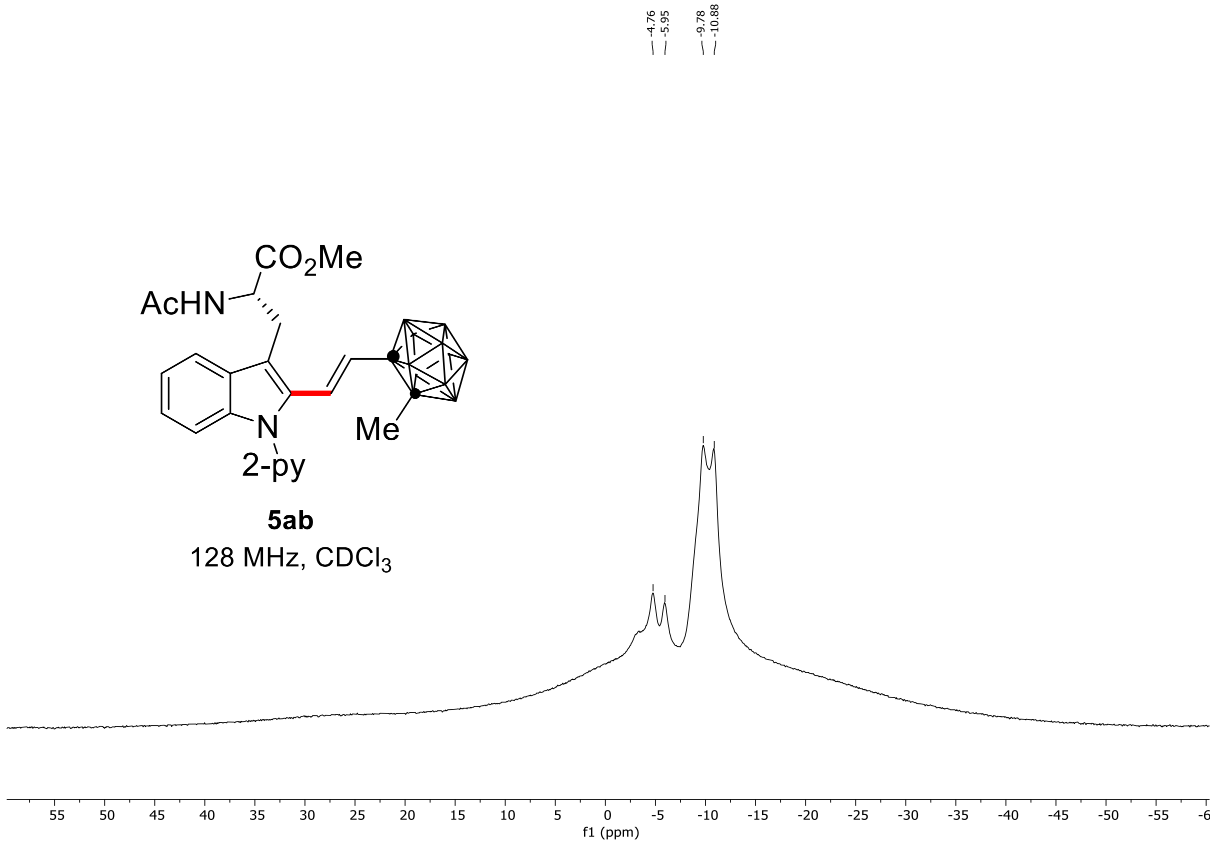

-4.76
-5.95
-9.78
-10.88
CO2Me
AcHN
N
2-py
Me
5ab
128 MHz, CDCl3
55 50 45 40 35 30 25 20 15 10 5 0 -5 -10 -15 -20 -25 -30 -35 -40 -45 -50 -55 -6
f1 (ppm)

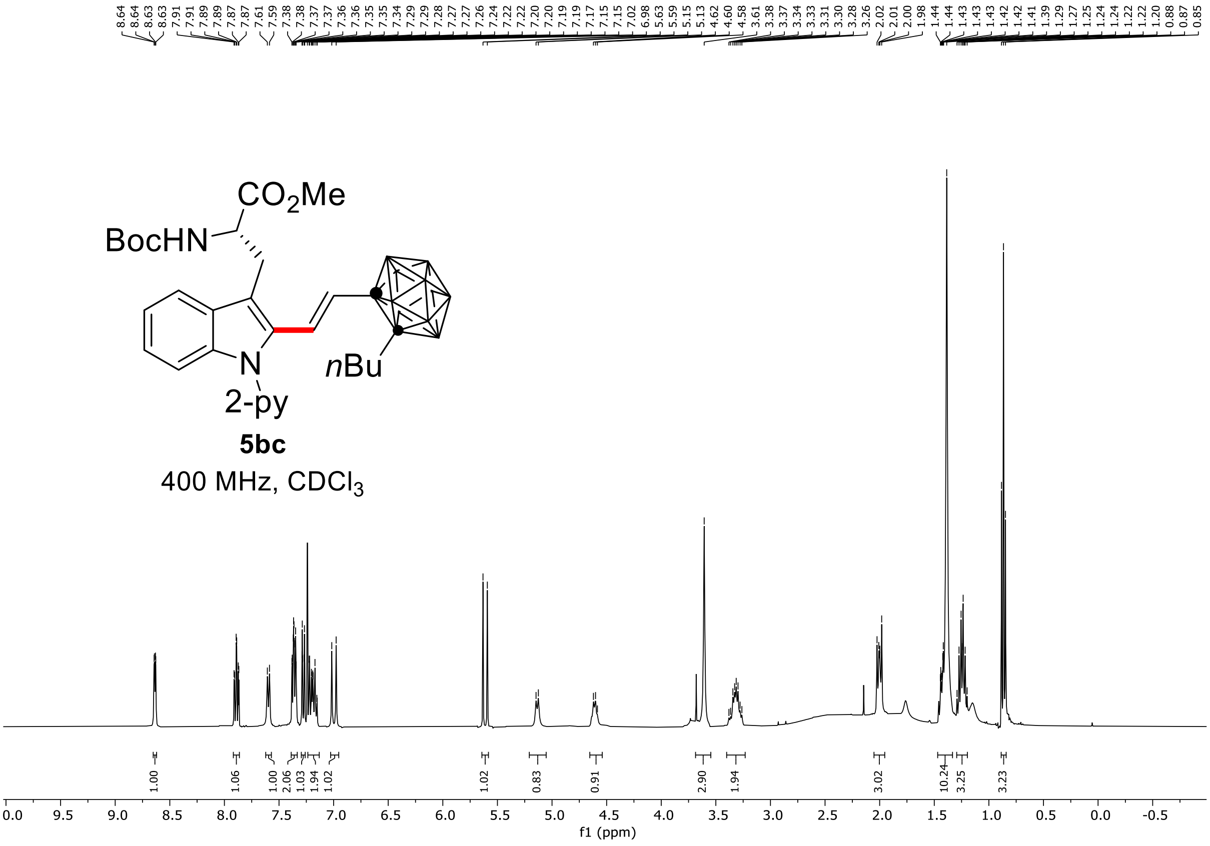
8.64 8.64 8.63 7.91 7.91 7.89 7.89 7.87 7.87 7.61 7.59 7.38 7.38 7.37 7.37 7.36 7.36 7.35 7.34 7.29 7.29 7.28 7.27 7.27 7.26 7.24 7.22 7.22 7.20 7.20 7.19 7.19 7.17 7.15 7.15 7.02 6.98 5.63 5.59 5.15 5.13 4.62 4.60 4.58 3.61 3.38 3.37 3.34 3.33 3.31 3.30 3.28 3.26 2.02 2.01 2.00 1.98 1.44 1.44 1.43 1.43 1.43 1.42 1.42 1.41 1.39 1.29 1.27 1.25 1.24 1.24 1.22 1.22 1.20 0.88 0.87 0.85
CO_2Me
BocHN
N
2-py
nBu
5bc
400 MHz, $CDCl_3$
1.00 1.06 1.00 2.06 1.03 1.94 1.02 1.02 0.83 0.91 2.90 1.94 3.02 10.24 3.25 3.23
f1 (ppm)

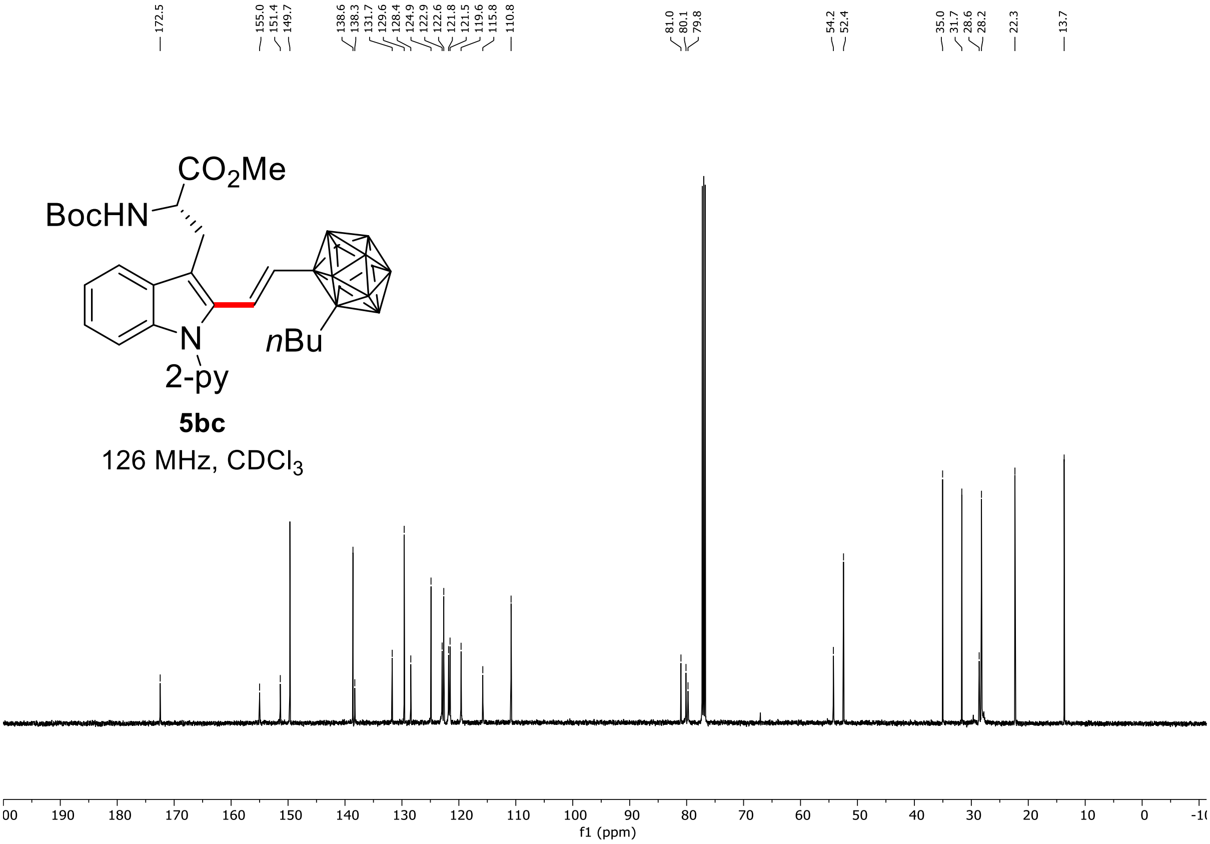
172.5 155.0 151.4 149.7 138.6 138.3 131.7 129.6 128.4 124.9 122.9 122.6 121.8 121.5 119.6 115.8 110.8 81.0 80.1 79.8 54.2 52.4 35.0 31.7 28.6 28.2 22.3 13.7
CO_2Me
BocHN
N
2-py
nBu
5bc
126 MHz, $CDCl_3$
f1 (ppm)

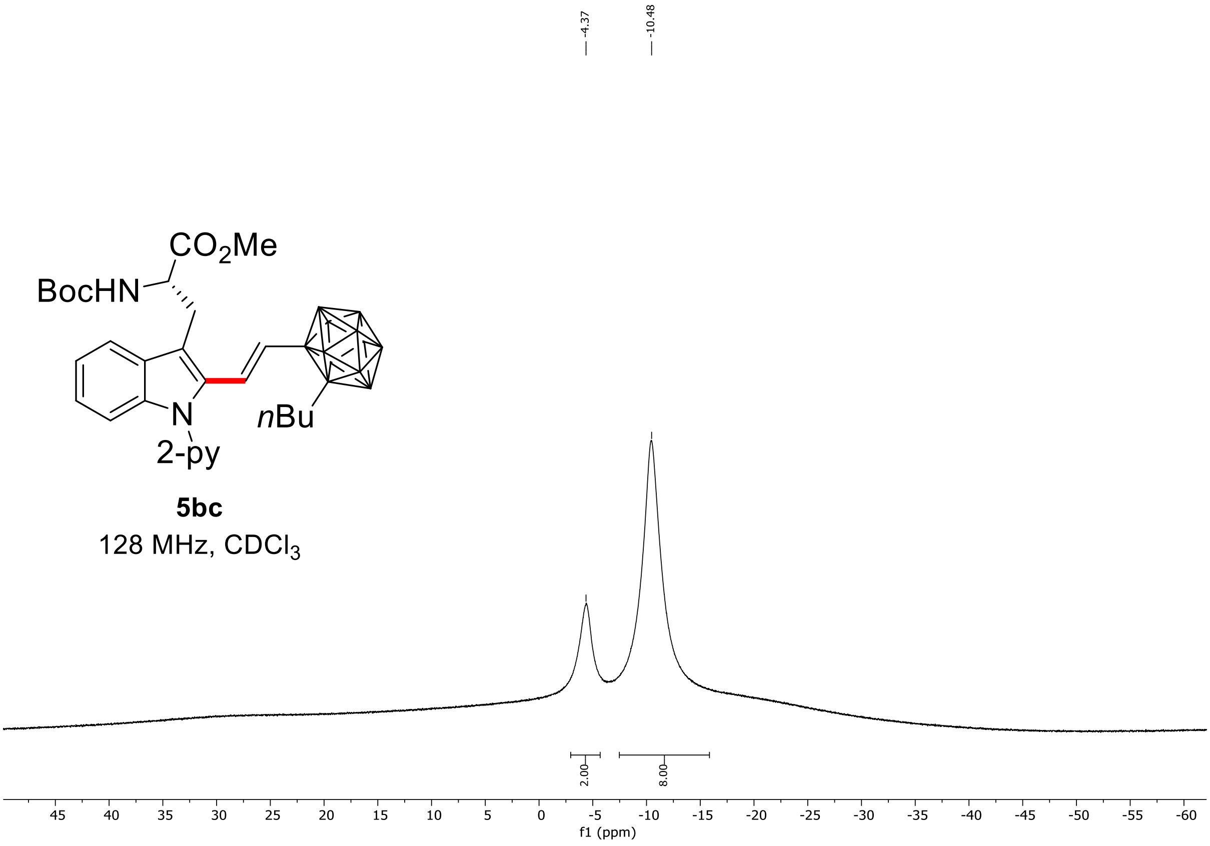

-4.37
-10.48
CO2Me
BocHN
N
2-py
nBu
5bc
128 MHz, CDCl3
2.00
8.00
45 40 35 30 25 20 15 10 5 0 -5 -10 -15 -20 -25 -30 -35 -40 -45 -50 -55 -60
f1 (ppm)

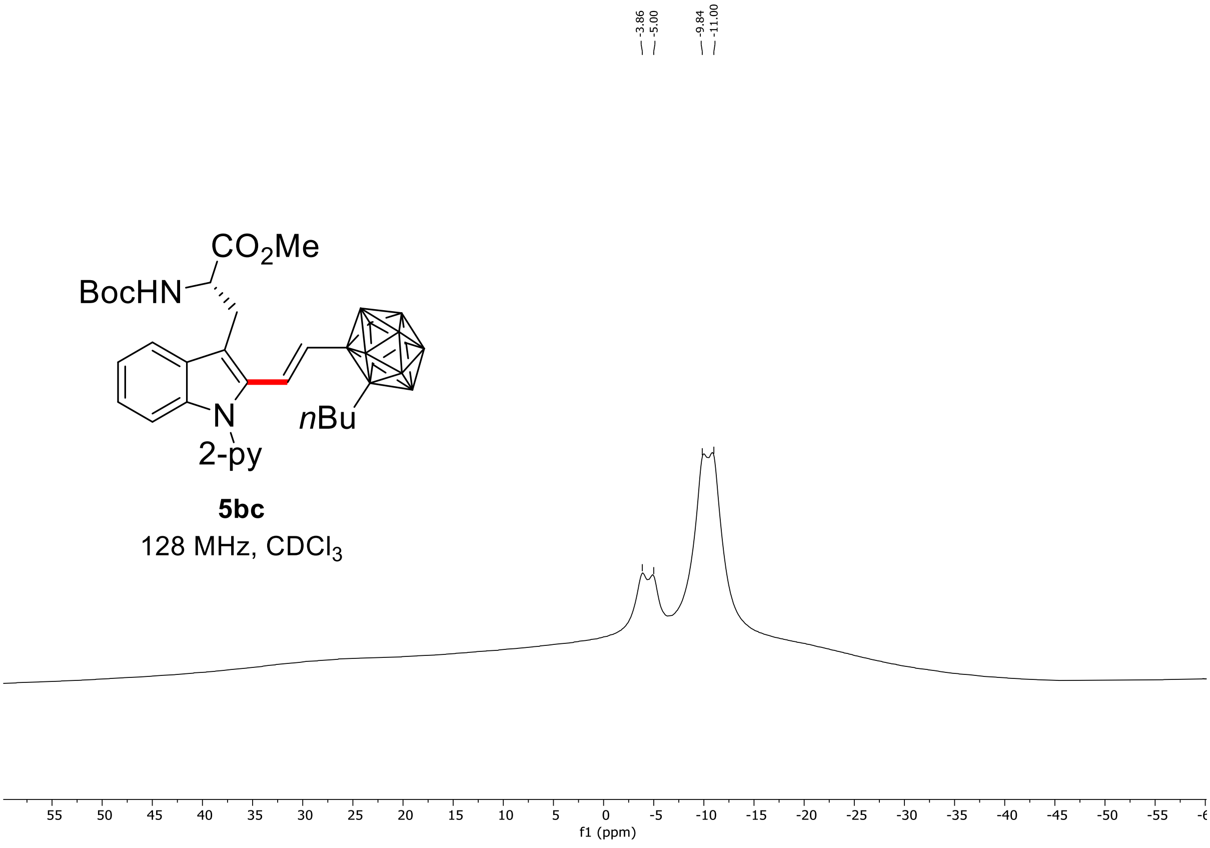

-3.86
-5.00
-9.84
-11.00
CO2Me
BocHN
N
2-py
nBu
5bc
128 MHz, CDCl3
55 50 45 40 35 30 25 20 15 10 5 0 -5 -10 -15 -20 -25 -30 -35 -40 -45 -50 -55
f1 (ppm)

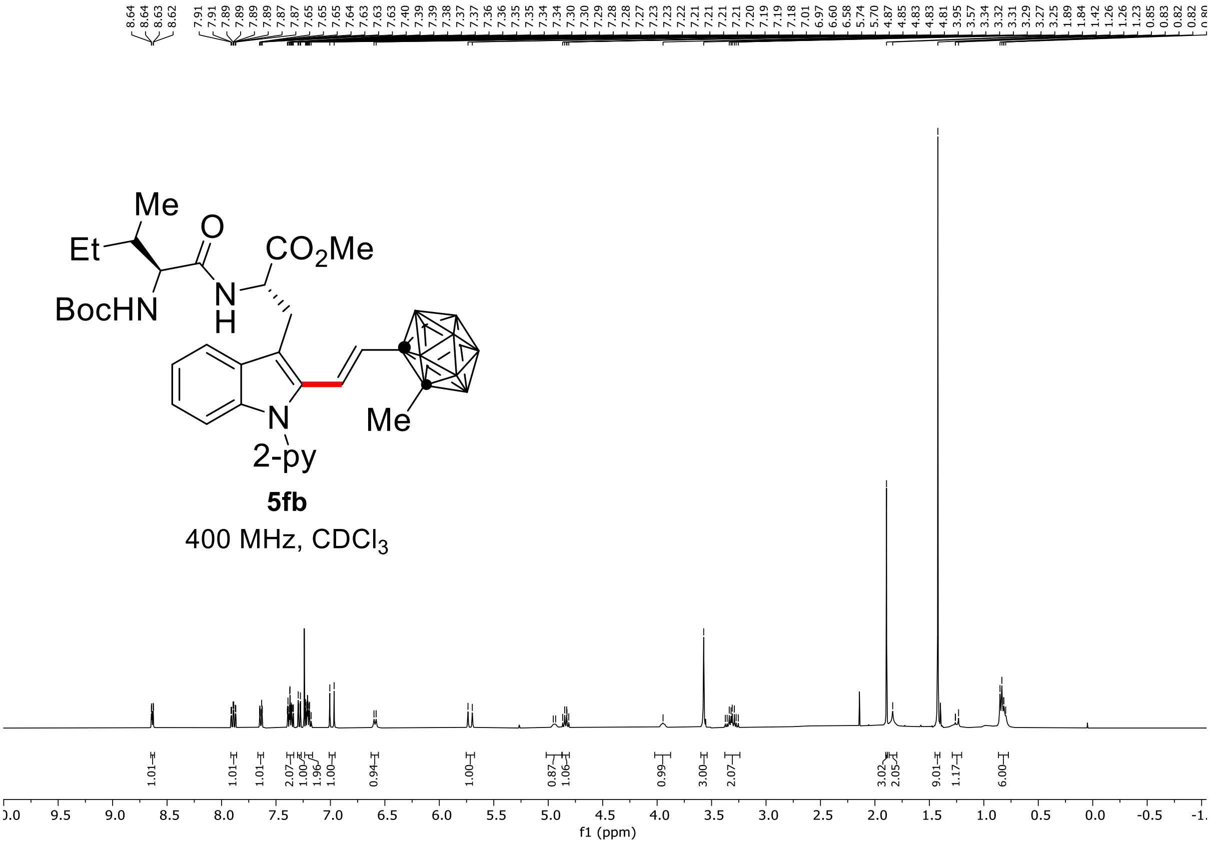
8.64 8.64 8.63 8.62 7.91 7.91 7.89 7.89 7.89 7.87 7.87 7.65 7.65 7.65 7.64 7.63 7.63 7.40 7.39 7.39 7.38 7.37 7.37 7.36 7.36 7.35 7.35 7.34 7.34 7.30 7.30 7.29 7.28 7.28 7.27 7.23 7.23 7.22 7.21 7.21 7.21 7.21 7.20 7.19 7.19 7.18 7.01 6.97 6.60 6.58 5.74 5.70 4.87 4.85 4.83 4.83 4.81 3.95 3.57 3.34 3.32 3.31 3.29 3.27 3.25 1.89 1.84 1.42 1.26 1.26 1.23 0.85 0.83 0.82 0.82
Me
O
Et
CO_2Me
BocHN
N
H
N
2-py
Me
5fb
400 MHz, $CDCl_3$
1.01 1.01 1.01 2.07 1.00 1.96 1.00 0.94 1.00 0.87 1.06 0.99 3.00 2.07 3.02 2.05 9.01 1.17 6.00
f1 (ppm)

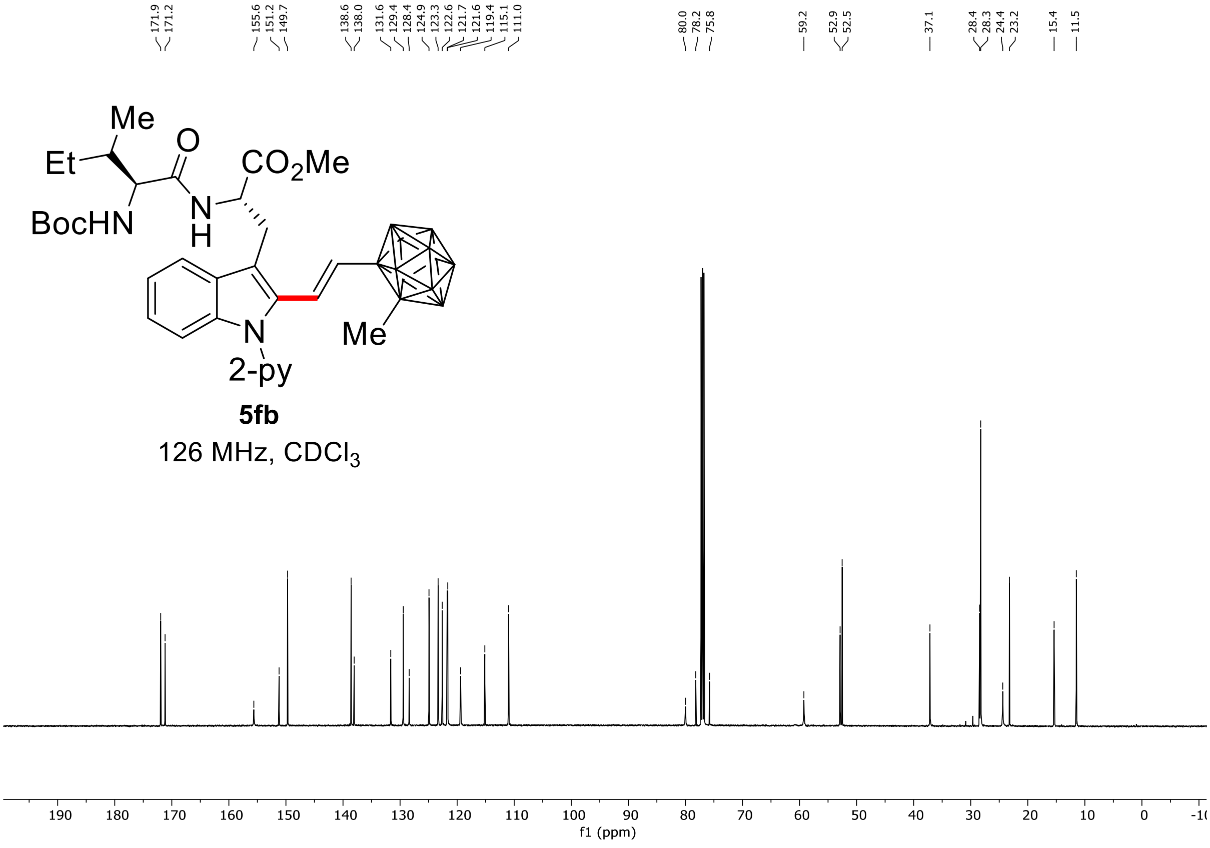
171.9 171.2 155.6 151.2 149.7 138.6 138.0 131.6 129.4 128.4 124.9 123.3 122.6 121.7 121.6 119.4 115.1 111.0 80.0 78.2 75.8 59.2 52.9 52.5 37.1 28.4 28.3 24.4 23.2 15.4 11.5
Me
O
Et
CO_2Me
BocHN
N
H
N
2-py
Me
5fb
126 MHz, $CDCl_3$
f1 (ppm)

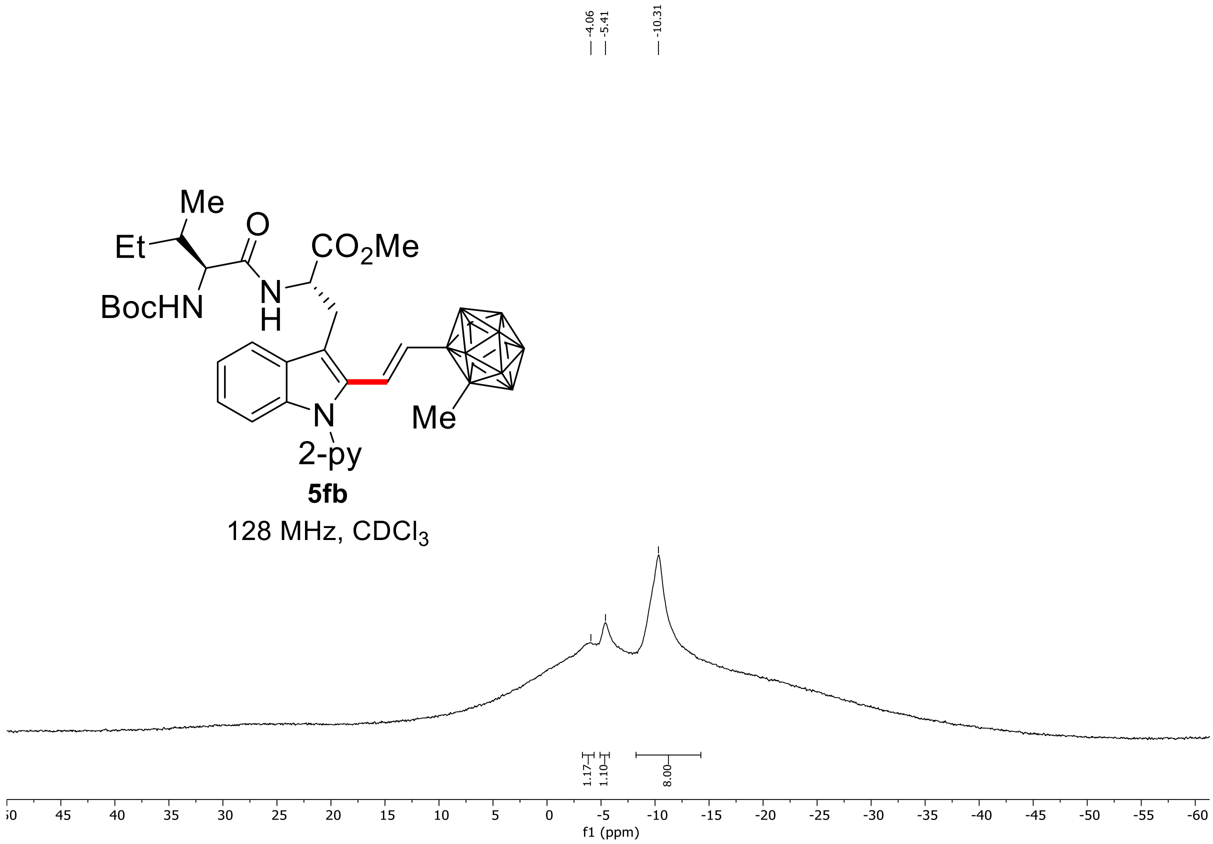

-4.06
-5.41
-10.31
Me
Et
O
CO2Me
BocHN
N
H
N
2-py
Me
5fb
128 MHz, CDCl3
1.17
1.10
8.00
f1 (ppm)

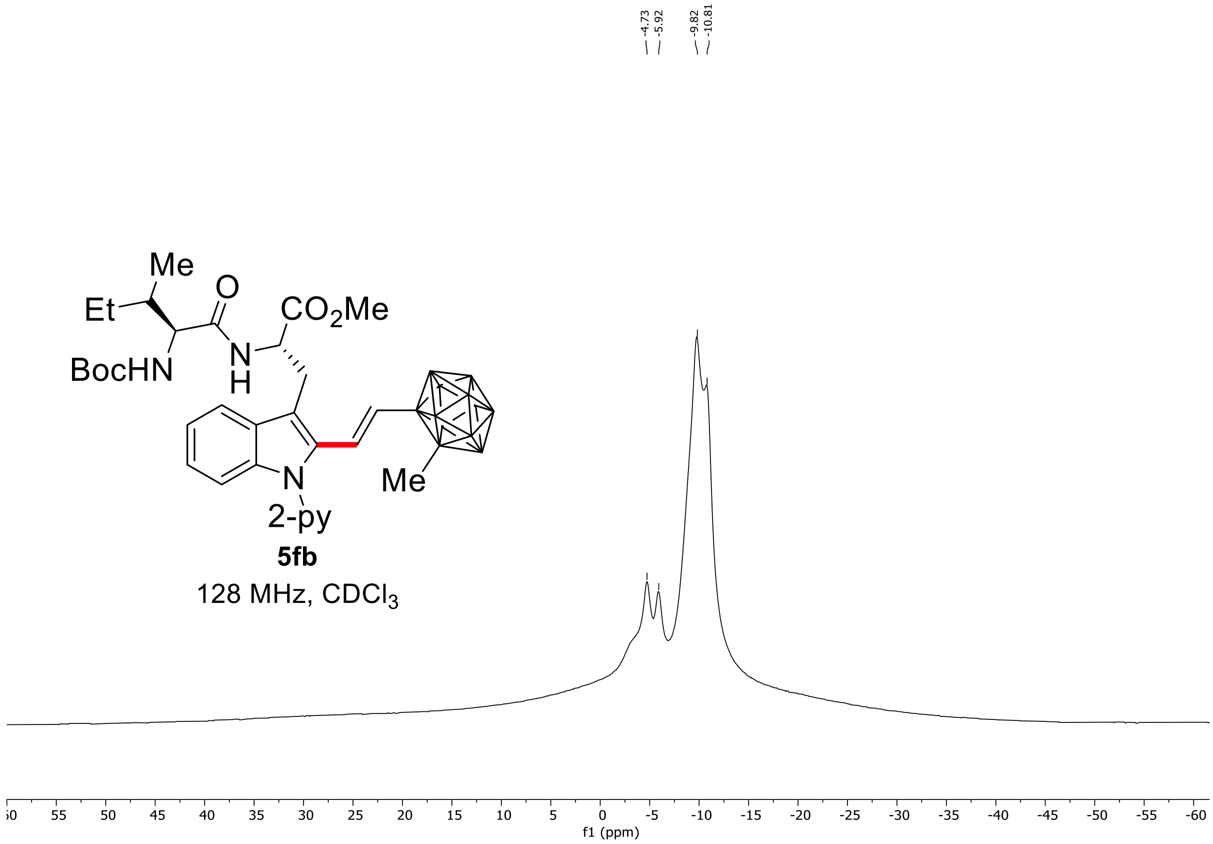

-4.73
-5.92
-9.82
-10.81
Me
Et
O
CO2Me
BocHN
N
H
N
2-py
Me
5fb
128 MHz, CDCl3
f1 (ppm)

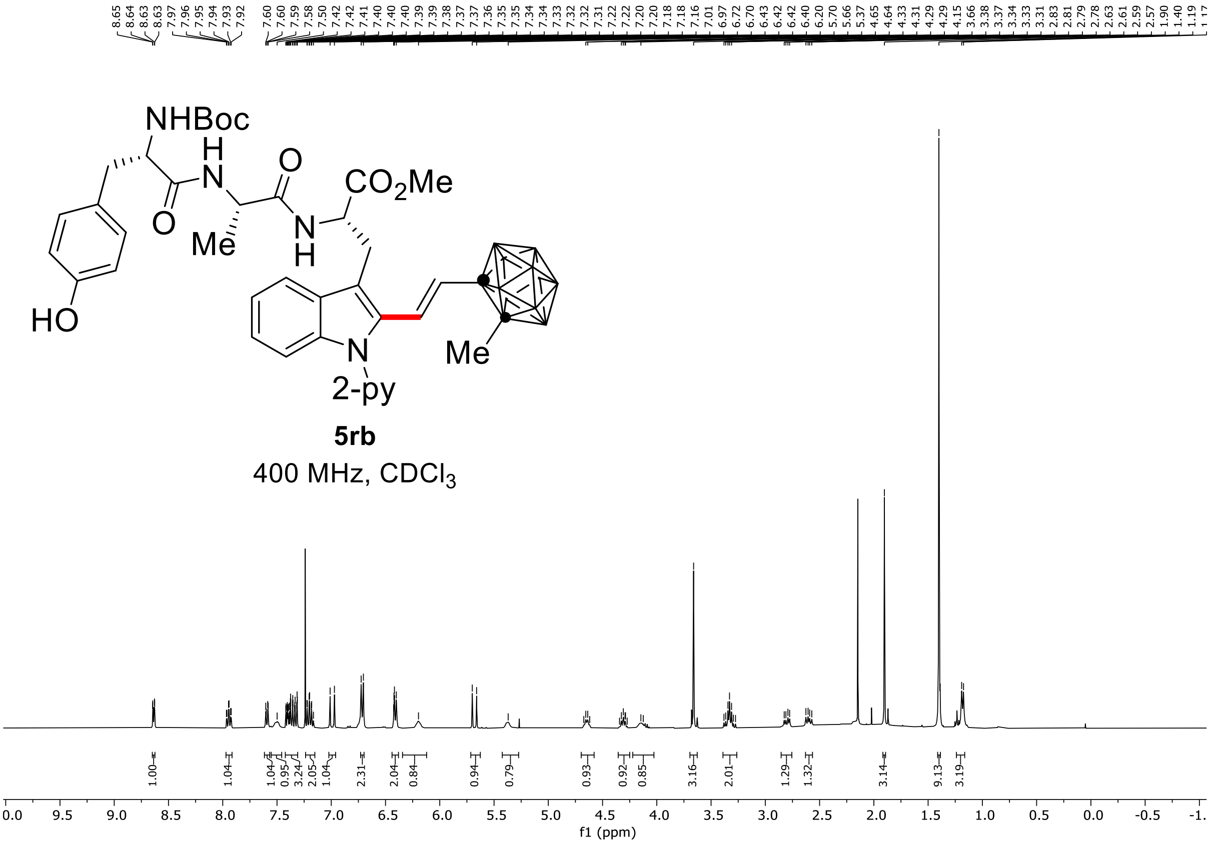
NHBoc
HO
Me
CO2Me
2-py
5rb
400 MHz, CDCl3
f1 (ppm)

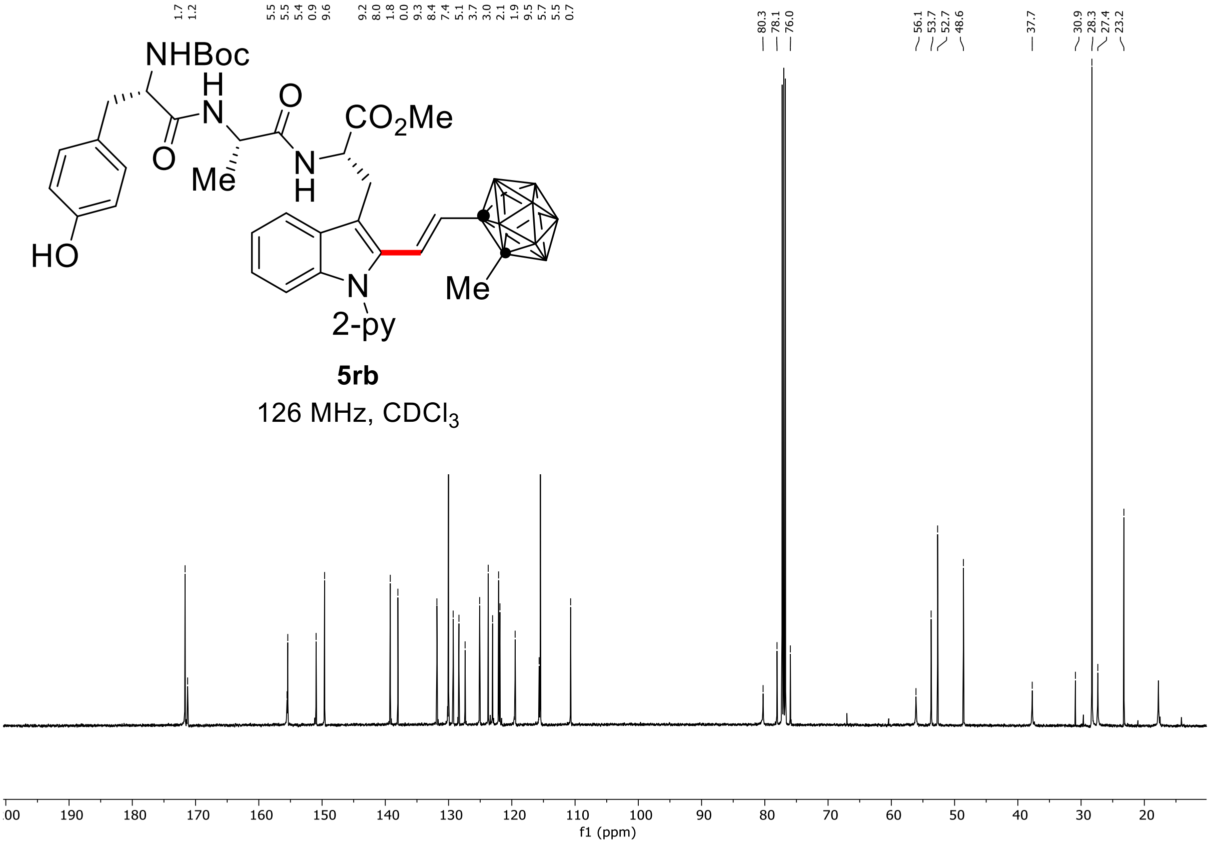
NHBoc
HO
Me
CO2Me
2-py
5rb
126 MHz, CDCl3
f1 (ppm)

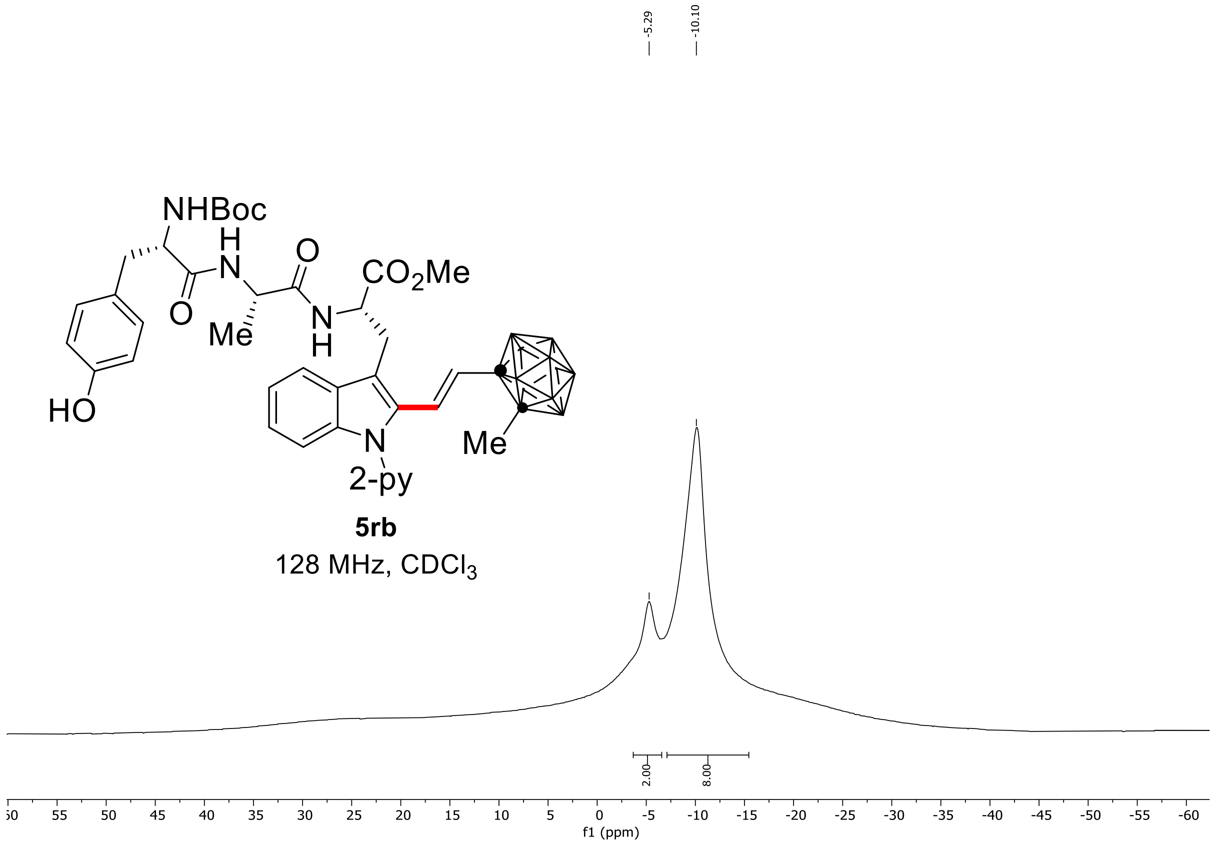

-5.29
-10.10
NHBoc
H
N
O
O
CO2Me
Me
N
H
HO
N
Me
2-py
5rb
128 MHz, CDCl3
2.00
8.00
f1 (ppm)

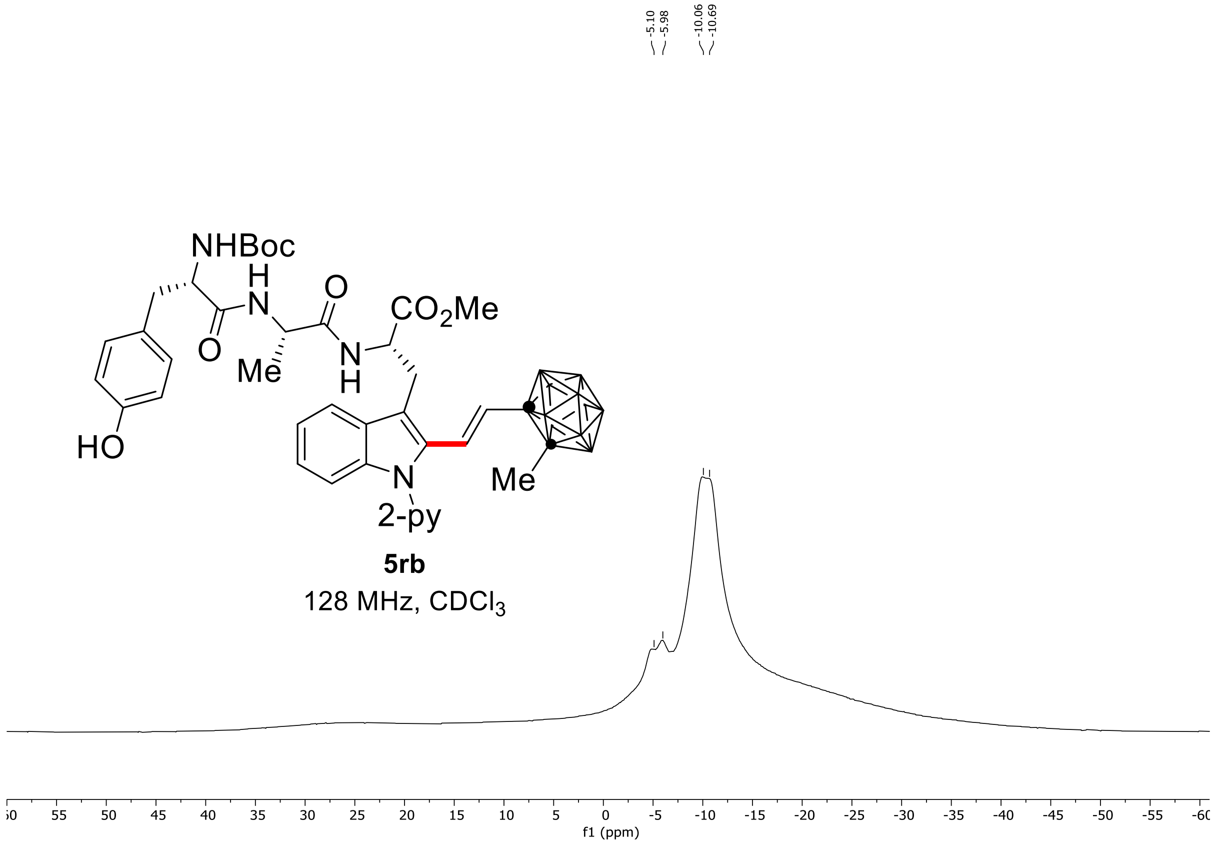

-5.10
-5.98
-10.06
-10.69
NHBoc
H
N
O
O
CO2Me
Me
N
H
HO
N
Me
2-py
5rb
128 MHz, CDCl3
f1 (ppm)

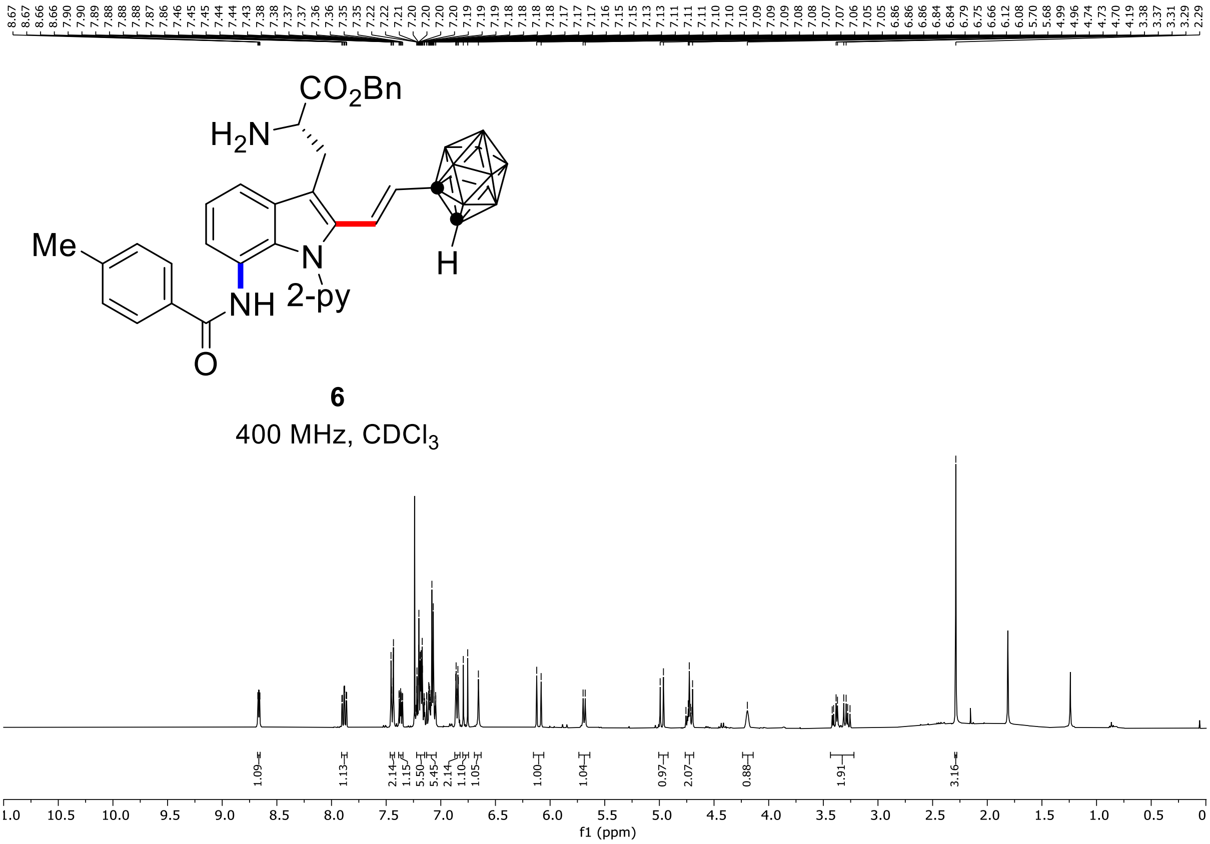
8.67 8.67 8.66 7.90 7.89 7.88 7.88 7.88 7.87 7.86 7.46 7.45 7.45 7.44 7.43 7.38 7.38 7.37 7.37 7.36 7.36 7.35 7.35 7.22 7.22 7.21 7.20 7.20 7.20 7.20 7.19 7.19 7.19 7.18 7.18 7.18 7.18 7.17 7.17 7.17 7.16 7.15 7.15 7.13 7.13 7.11 7.11 7.11 7.10 7.10 7.10 7.09 7.09 7.09 7.08 7.08 7.07 7.07 7.06 7.05 7.05 6.86 6.86 6.86 6.84 6.79 6.75 6.66 6.12 6.08 5.70 5.68 4.99 4.96 4.74 4.73 4.70 4.19 3.38 3.37 3.31 3.29 2.29
CO2Bn
H2N
Me
N
2-py
NH
O
H
6
400 MHz, CDCl3
1.09 1.13 2.14 1.15 5.50 5.45 2.14 1.10 1.05 1.00 1.04 0.97 2.07 0.88 1.91 3.16
f1 (ppm)

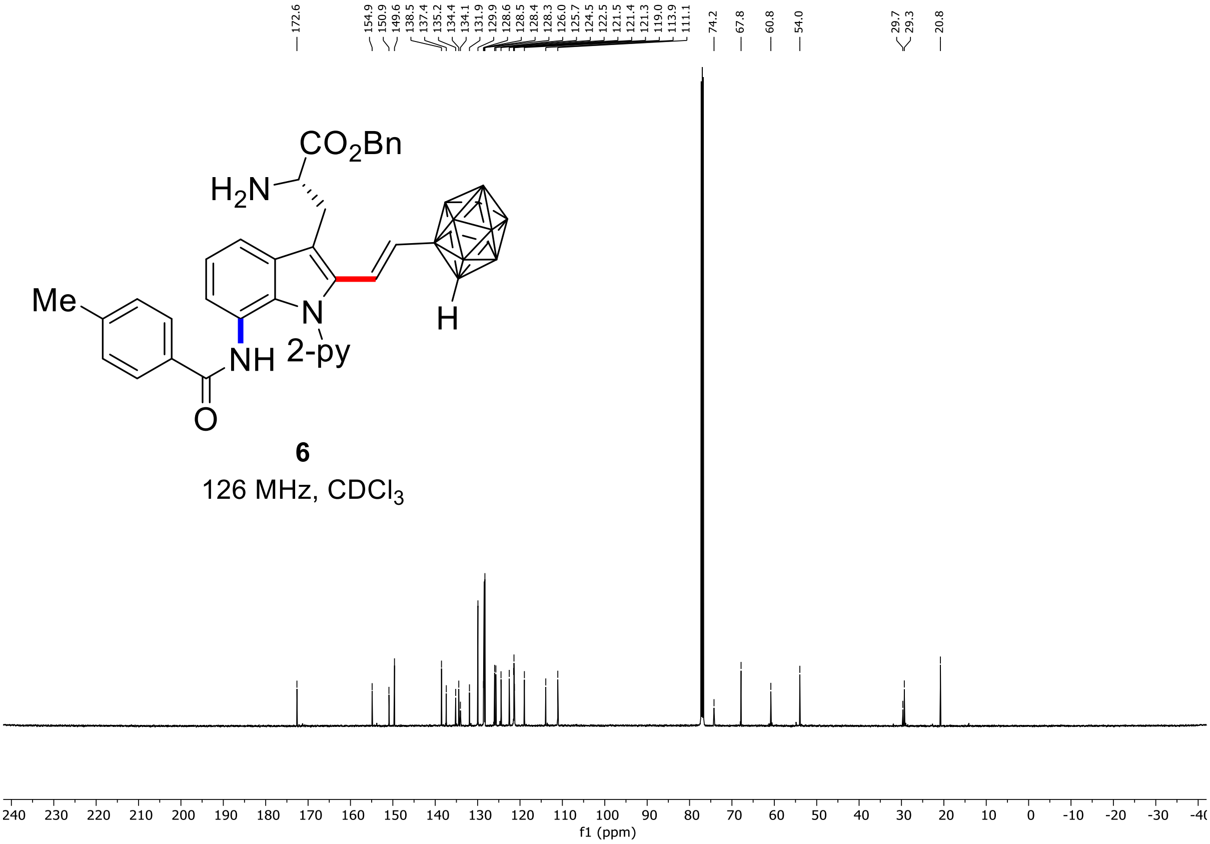
172.6 154.9 150.9 149.6 138.5 137.4 135.2 134.4 134.1 131.9 129.9 128.6 128.5 128.4 128.3 126.0 125.7 124.5 122.5 121.5 121.4 121.3 119.0 113.9 111.1 74.2 67.8 60.8 54.0 29.7 29.3 20.8
CO2Bn
H2N
Me
N
2-py
NH
O
H
6
126 MHz, CDCl3
f1 (ppm)

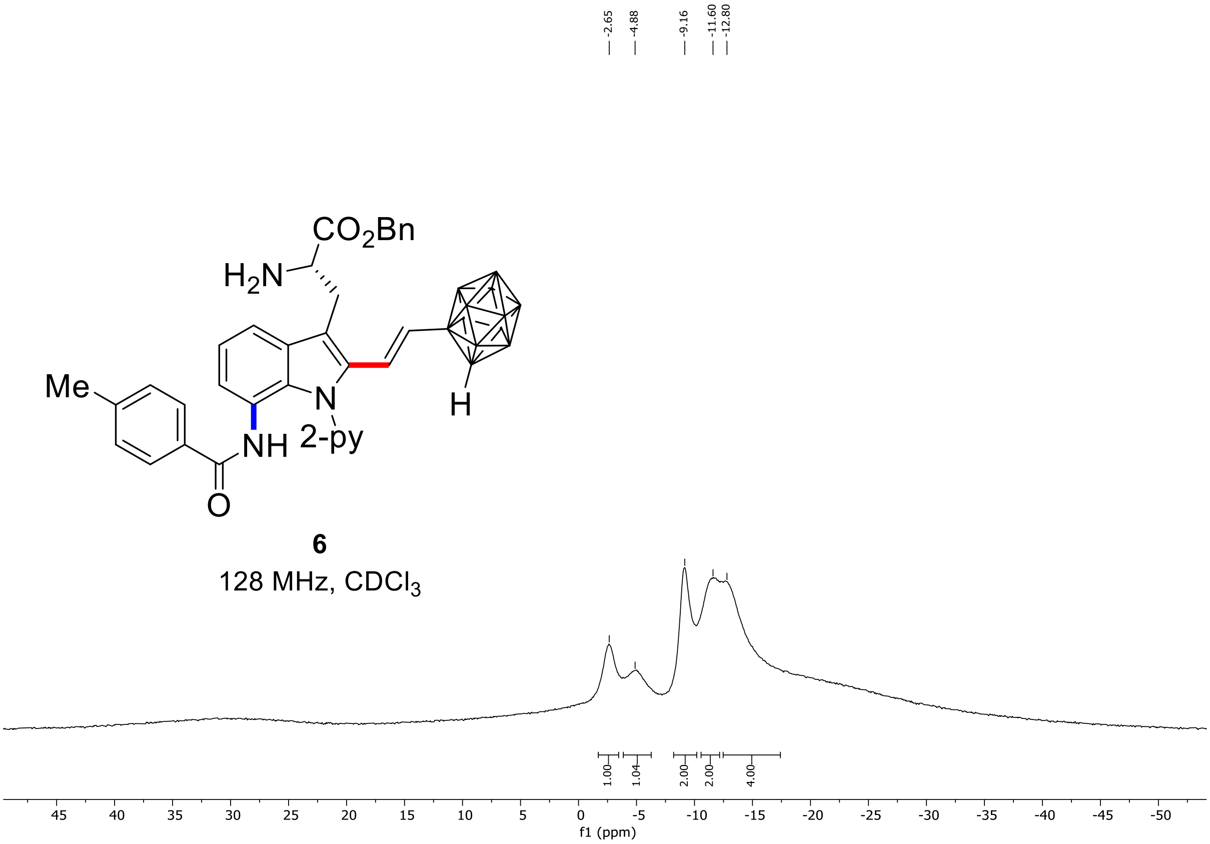
-2.65
-4.88
-9.16
-11.60
-12.80
CO2Bn
H2N
Me
NH
O
N
2-py
H
6
128 MHz, CDCl3
1.00
1.04
2.00
2.00
4.00
45 40 35 30 25 20 15 10 5 0 -5 -10 -15 -20 -25 -30 -35 -40 -45 -50
f1 (ppm)

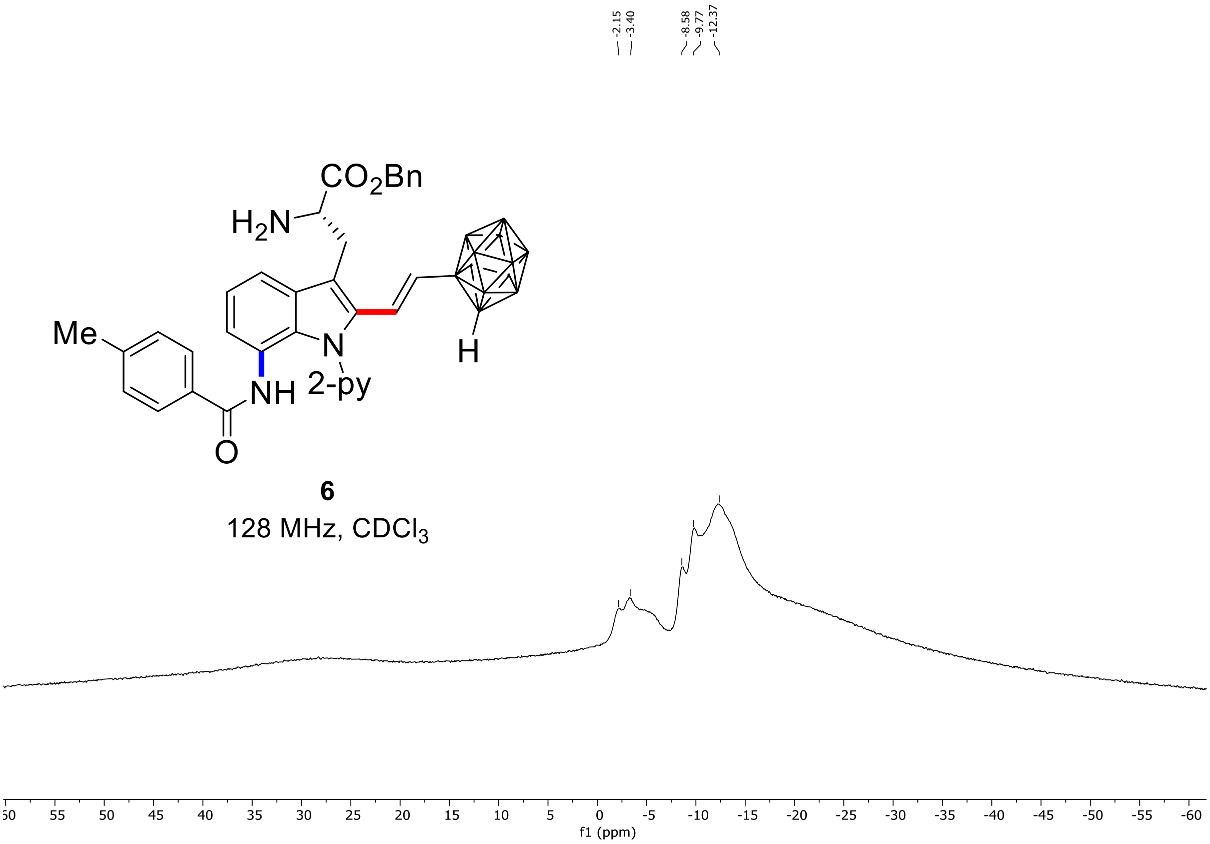
-2.15
-3.40
-8.58
-9.77
-12.37
CO2Bn
H2N
Me
NH
O
N
2-py
H
6
128 MHz, CDCl3
55 50 45 40 35 30 25 20 15 10 5 0 -5 -10 -15 -20 -25 -30 -35 -40 -45 -50 -55 -60
f1 (ppm)
